# Site Selective Boron Directed *Ortho* Benzylation of *N*-Aryl Amides: Access to Structurally Diversified Dibenzoazepines

**DOI:** 10.1021/acs.orglett.4c04196

**Published:** 2024-12-17

**Authors:** Ganesh H. Shinde, Hugo Castlind, Ganesh S. Ghotekar, Francoise M. Amombo Noa, Lars Öhrström, Henrik Sundén

**Affiliations:** † Department of Chemistry and Molecular Biology, University of Gothenburg, SE-41296 Gothenburg, Sweden; ‡ Department of Chemistry and Chemical Engineering, Chalmers University of Technology, SE-41296 Gothenburg, Sweden

## Abstract

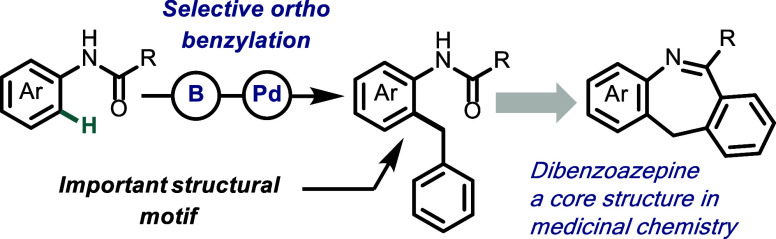

We present a highly selective protocol for the *ortho* benzylation of *N*-aryl amides. This method offers mild conditions, excellent site specificity, and scalability, enabling the synthesis of diarylmethane amides and dibenzoazepines. The protocol allows for one-pot diagonal dibenzylation of dianilides, creating valuable precursors for pharmaceutically active compounds and addressing limitations in current direct C–H activation methodologies.

## Introduction

**Figure 1:**
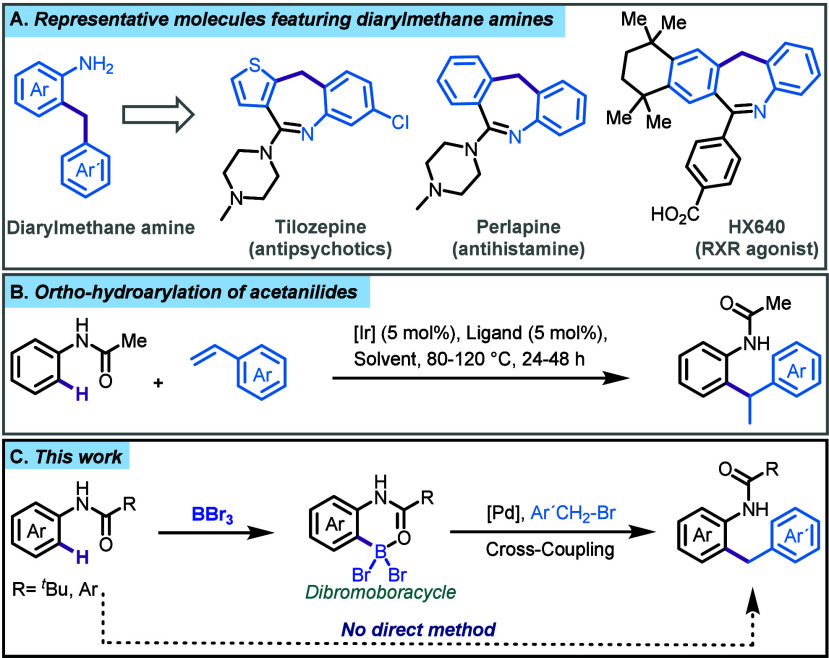
A) The diarylmethane amine component is a common structural feature in various pharmaceutically active dibenzoazepine derivatives. B) Direct method for *ortho*-hydroarylation of acetanilides. C) This work: Site selective boron directed *ortho* benzylation of *N*-aryl amides.

Diarylmethane amides and amines, particularly functionalized ones, represent a crucial structural motif serving as key components in therapeutic agents and bioactive molecules to play essential roles in organic synthesis.^1^ For instance, the diarylmethane amine component is found in various pharmaceutically active dibenzoazepine derivatives, such as tilozepine,^2^ perlapine,^3^ and HX640^4^ (Figure 1A). Traditionally, the synthesis of diarylmethane amides has been achieved by coupling benzylanilines with acyl chlorides under basic conditions.^5^ However, commercially available benzylanilines are scarce; therefore, the direct insertion of the benzyl group into an anilide is desirable, as this approach can lead to a diverse pool of substrates as both coupling partners can be varied. For instance, Bower and co-workers have shown that *N*-phenyl acetamides can undergoes an Ir-catalyzed hydroarylation with styrenes (Figure 1B).^6^ Despite these advances, a direct C–H activation strategy for the synthesis of diarylmethane amides from anilides remains elusive (Figure 1C). Given the lack of direct C–H benzylation methodologies for anilides, it becomes relevant to examine existing C–H activation strategies for benzylation using various directing groups.^7^ While C–H activation approaches have provided valuable tools for C–H benzylation, the methods reported so far heavily rely on the use of bidentate directing groups,^7b−7d^) which, although they improve selectivity, introduce additional synthetic steps for the introduction and removal of the directing group. Furthermore, the necessity for sensitive benzylating reagents^7e−7g^) in many of these reactions limits functional group tolerance and substrate scope. Additionally, these methods often require expensive catalysts,^7a−7c^) and harsh reaction conditions,^7a,7f^ further complicating the synthetic process and limiting practical applications.

Thus, developing a more efficient strategy to address these limitations would represent a significant advancement in the synthesis of structurally diverse diarylmethane amides.

Recently, boron tribromide (BBr_3_) has been effectively employed in metal-free strategies for the *ortho-*selective incorporation of boron into anilides.^8,9^ In our previous research, we demonstrated the selective formation and diverse reactivity of dibromoboracycles.^10^ Notably, we established that these boracycles can be utilized in Csp^2^–Csp^2^ cross-coupling reactions.^10d^ Building on this foundation and considering the limited precedent in the literature for direct benzylation of anilides, we hypothesized that the dibromoboracycle could serve as a starting point to facilitate Csp^2^–Csp^3^ coupling with benzyl bromides (Figure 1C) enabling the synthesis of synthetically valuable diarylmethane amides that were previously inaccessible through traditional methods.

### Results and Discussion

Preliminary studies involved exposing the dibromoboracycle derived from pivalamide **1a** and benzyl bromide to Suzuki-Miyaura cross-coupling conditions. At 70 °C in 1.5 mL of alkaline methanol, the benzylation product **3a** was obtained in 49% yield, along with a side product **4a** in 17% yield (entry 1, Table 1). This indicated competing reactivity between C(sp^2^)–C(sp^3^) and homo coupled C(sp^2^)–C(sp^2^) bond formation. Additionally, we observed a cross reactivity between methanol and benzyl bromide, further decreasing the efficiency of the transformation. However, the addition of water significantly improved the reaction outcome, yielding the desired product in excellent 89% yield with only 2% of **4a** forming (entry 2, Table 1). Increasing the loading of benzyl bromide to 1.5 equiv eliminated the formation of **4a**, resulting in an improved yield of product **3a** (91%, entry 3, Table 1). However, when 3 mol % of catalyst was used, the yield decreased, although the **3a** remained in trace amounts (entry 4 vs 3, Table 1). In the absence of both the catalyst and the base, the reaction failed to provide **3a** highlighting their crucial roles in the process (entries 6–7, Table 1). Benzyl chloride and benzyl alcohol were also evaluated as alternative benzylation reagents. Benzyl chloride proved effective, yielding the desired product in 80% along with the homocoupled byproduct **4a** (entry 8, Table 1). The increased formation of **4a** is likely due to the slower reaction rate of benzyl chloride compared to benzyl bromide, which allowed for greater homocoupling. In contrast, benzyl alcohol was completely ineffective under the reaction conditions, failing to produce the desired product (entry 9, Table 1).

**Table 1:**
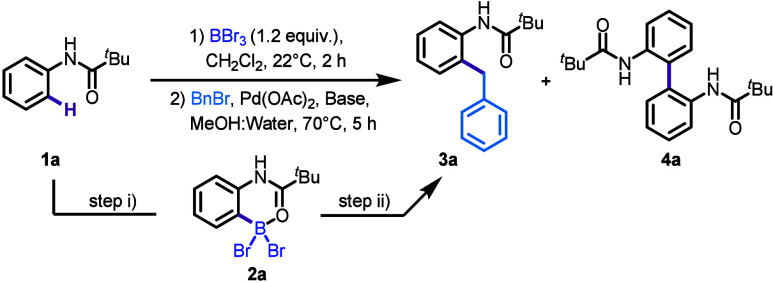
Reaction Optimization^a^

Entry	Catalyst (mol %)	Solvent	Yield^h^ (**3a**)	Yield^h^ (**4a**)
1	Pd(OAc)_2_ (5)	MeOH	49%	17%
2^b^	Pd(OAc)_2_ (5)	MeOH:Water	89%	2%
3	Pd(OAc)_2_ (5)	MeOH:Water	91%	trace
4	Pd(OAc)_2_ (3)	MeOH:Water	84%	trace
5^c^	Pd(OAc)_2_ (5)	MeOH:Water	88%	trace
6^d^	Pd(OAc)_2_ (5)	MeOH:Water	0%	0%
7	---	MeOH:Water	0%	0%
8^e^^,^^f^	Pd(OAc)_2_ (5)	MeOH:Water	80%	14%
9^g^^,^^f^	Pd(OAc)_2_ (5)	MeOH:Water	0%	trace

a Reaction conditions: Step (i) **1a** (0.15 mmol), BBr_3_ (0.18 mmol), in 0.5 mL anhydrous CH_2_Cl_2_ at 22 °C, 2 h; Step (ii) benzyl bromide (0.225 mmol), potassium carbonate (K_2_CO_3_, 0.45 mmol), Pd(OAc)_2_ (5 mol %), in 0.8 mL MeOH and 0.8 mL Water 70 °C for 5 h.

b Benzyl bromide (0.18 mmol).

c MeOH:Water (2:1 ratio).

d Without base.

e Benzyl chloride instead of Benzyl bromide.

f Step (ii) time 16 h.

g Benzyl alcohol instead of Benzyl bromide.

h Isolated yields.

**Scheme 1:**
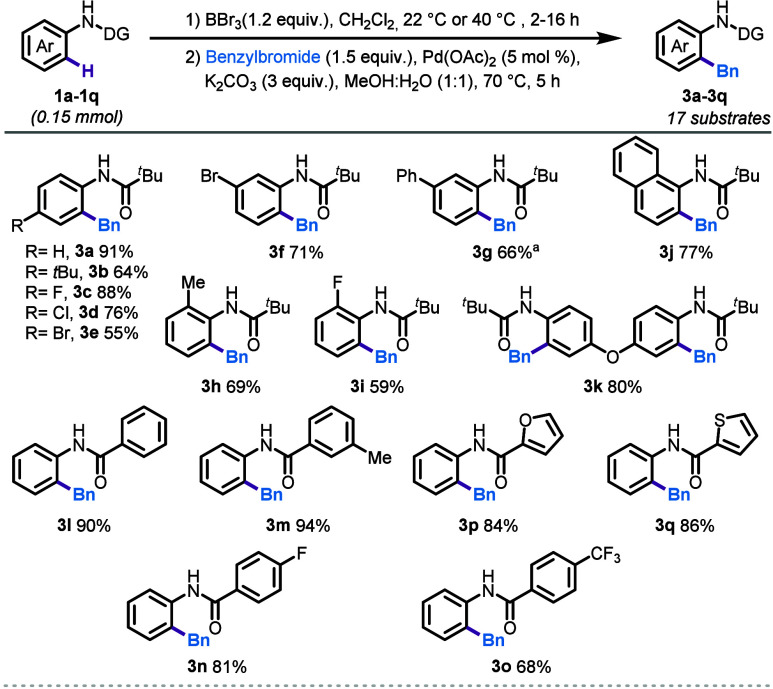
Reaction Scope Step 2 at 70 °C for 16 h The *N*-aryl pivalamide component: (**1a**–**1k**) step 1 at 22 °C, 2 h; *N*-phenyl benzanilides: (**1l**–**1q**) step 1 at 40 °C for 16 h.

Having optimized the conditions for the *ortho*-benzylation of anilides, the scope of the reaction was evaluated using a diverse set of substrates (Scheme 1). Initially, a series of pivalamides (**1a**–**1k**) were tested, revealing excellent tolerance for various functional groups. Both electron-donating (**1b**) and electron-withdrawing substituents (**1c**–**1e**) at the *para* position were well-tolerated, yielding the desired benzylation products in moderate to excellent yields (55–88%, Scheme 1). Substituents at the *meta* and *ortho* positions also afforded products in moderate to good yields (**1f**–**1i**, 59–71%). Notably, substrates with bromo substituents (**1e** and **1f**), which are typically reactive under palladium-catalyzed conditions, were well-tolerated, yielding the desired products in 55% and 71%, respectively. Extended aromatic compound **1j** also provides the desired product **3j** in 77% yield. Next, double benzylation was also achieved on substrate **1k**, yielding the product **3k** in 80% yield.

Next, we investigated benzanilides to explore site-selective benzylation on the aniline portion of the ring (**1l**–**1q**, Scheme 1). Various substituents on the phenyl ring, including methyl (**1m**), fluoro (**1n**), and trifluoromethyl groups (**1o**), were well accommodated under the optimized conditions, resulting in the desired benzylated products (**3l**–**3o**) with yields ranging from 68% to 94%. Additionally, heterocyclic anilides (**1p**–**1q**) demonstrated good reactivity, yielding the targeted benzylation products in 84% and 86%, respectively.

**Scheme 2:**
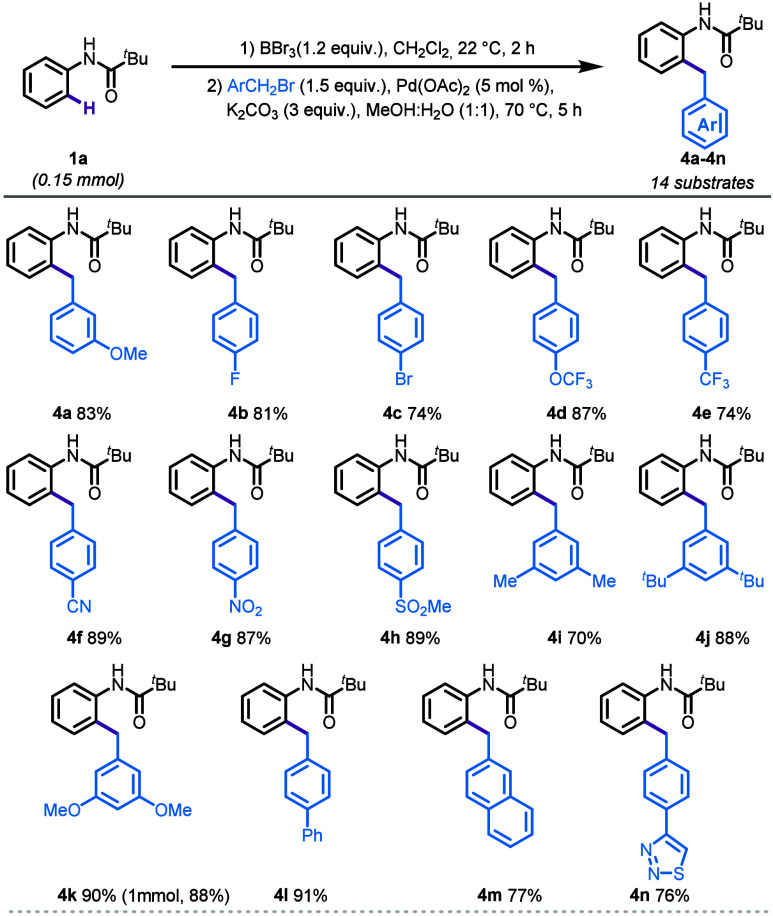
Reaction Scope: The Benzyl Component

After successfully installing benzyl groups on various anilides, we next investigated the scope of benzylation with *N*-phenylpivalamide (**1a**). Substrates bearing methoxy, halogen, and trifluoromethoxy (−OCF_3_) substituents on the benzyl ring were well-tolerated under similar conditions, yielding the desired products in 74–87% yield (**4a**-**4d**, Scheme 2). Furthermore, substrates with electron-withdrawing groups at the *para* position, such as trifluoromethyl (−CF_3_), cyano (−CN), nitro (−NO_2_), and methylsulfonyl (−SO_2_Me), also provided the desired benzylated products in good to excellent yields (74–89%, **4e**–**4h**, Scheme 2). This demonstrates the broad applicability of the benzylation protocol, showing excellent functional group compatibility and high efficiency across a range of electron-donating and electron-withdrawing substituents. Next, disubstituted and extended aromatic substrates also yielded the desired products in excellent yields (70–91%, **4i**–**4m**). A scale-up reaction performed on a 1 mmol scale for the synthesis of product **4k** demonstrated excellent yield, highlighting the robustness and practical applicability of our method. Notably, the use of benzyl bromide bearing a heterocyclic thiadiazole resulted in a 76% yield (**4n**), demonstrating the robustness of the reaction conditions without significantly impacting the overall efficiency.

**Scheme 3:**
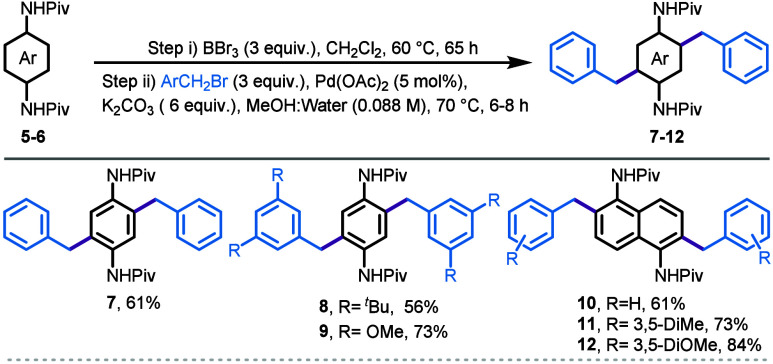
Diagonal Diarylation of Dianilides (**5** and **6**)

We were also interested in exploring whether it was possible to perform a one-pot dibenzylation on substrates, such as phenylenediamides, to introduce dibenzyl moieties in a diagonal orientation. As it turns out, the reaction is both general and completely selective, transforming dianilides **5** and **6** in combination with various benzyl bromides into the corresponding dibenzylated compounds (entries 7–12, Scheme 3). For instance, disubstituted benzyl bromides bearing *tert*-butyl and methoxy groups were well tolerated by the reaction providing compounds **8** and **9** in 56% and 73% yield, respectively with complete selectivity for the diagonal dibenzylated product (Scheme 3). Similarly, a naphthalene-derived dianilide **6** also underwent a smooth transformation, providing diagonal dibenzylation products in good to excellent yields (**10**–**12**, 61–84%). Diagonal dibenzylated compounds can potentially be used in synthesis of diagonal azepines.

**Scheme 4:**
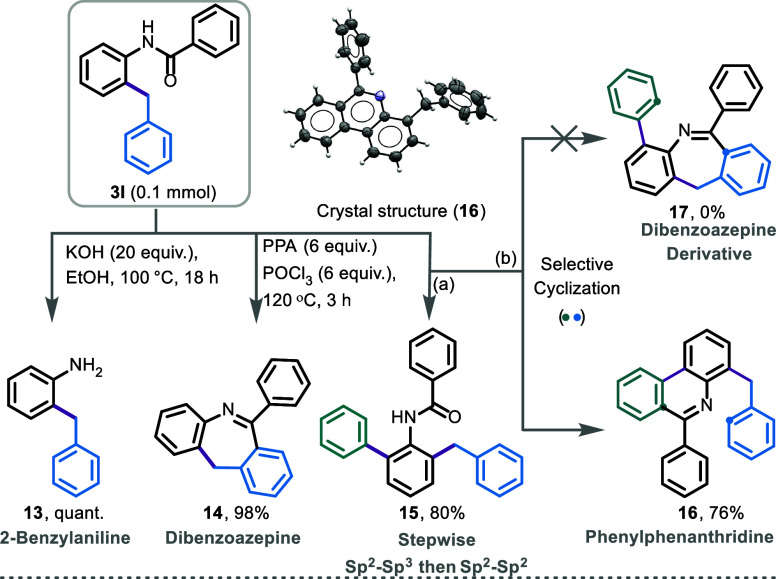
Postfunctionalization and Applications Condition (a): Refer to SI Section 3. Condition (b): PPA (10 equiv), POCl_3_ (10 equiv), 120 °C, 3.5 h.

The synthetic value of this protocol was further demonstrated by a range of transformations of compound **3l** including the synthesis of core structures found in biologically active compounds (Scheme 4). For example, amide **3l** can be converted to the corresponding 2-benzylaniline (**13**) in quantitative yield under basic conditions (Scheme 4). Furthermore, the benzyl group can undergo an acid-catalyzed intramolecular cyclization, leading to the pharmaceutically active dibenzoazepine (**14**) in 98% yield. Additionally, we synthesized difunctional benzanilide (**15**) in 80% yield under similar conditions using sequential Csp^2^–Csp^3^ and Csp^2^–Csp^2^ cross-coupling of a dibromoboracycle. Compound **15** is of particular interest, as it can be directly utilized to access selective phenanthridine derivatives. Notably, under acidic conditions, we exclusively observed the formation of a phenanthridine derivative (**16**) rather than the dibenzoazepine derivative (**17**).

Based on our previous report on Csp^2^–Csp^2^ coupling,^10d^ we propose a similar mechanism. It begins with the carbonyl-directed borylation of **1a**, leading to the formation of the dibromo boracycle (**2a**). Subsequent base-promoted ligand exchange on boron under basic conditions is followed by coupling with benzyl bromide, which proceeds through a Suzuki-Miyaura coupling (SMC)–type mechanism^11^ to form the desired product **3a**.

In conclusion, our research has successfully introduced a first highly efficient and versatile protocol for the *ortho* benzylation of *N*-aryl amides through the formation of a dibromo boracycle. Our methodology demonstrates excellent tolerance for various functional groups and allows for the construction of complex molecular architectures, including pharmaceutically relevant dibenzoazepines. The one-pot diagonal dibenzylation strategy further enhances the synthetic utility of our protocol, offering new avenues for the synthesis of valuable organic compounds. This work contributes to the advancement of Csp^2^–Csp^3^ coupling reactions and provides a practical solution for the synthesis of substituted 2-benzyl anilines and dibenzoazepines.

## Data Availability

The data underlying this study are available in the published article and its Supporting Information.

## Supporting Information Available

Experimental procedures, compound synthesis, and characterization (PDF)

## References

[ref1] aPeng X.; Sun Z.; Kuang P.; Li L.; Chen J.; Chen J. Copper-Catalyzed Selective Arylation of Nitriles with Cyclic Diaryl Iodonium Salts: Direct Access to Structurally Diversified Diarylmethane Amides with Potential Neuroprotective and Anticancer Activities. Org. Lett. 2020, 22, 5789–5795. 10.1021/acs.orglett.0c01829. 32677838

[ref2] Meigh J. P. K. Benzazepines and Their Group 15 Analogues. Sci. Synth. 2004, 17, 825–927.

[ref3] Warawa E. J.; Migler B. M.; Ohnmacht C. J.; Needles A. L.; Gatos G. C.; McLaren F. M.; Nelson C. L.; Kirkland K. M. Behavioral Approach to Nondyskinetic Dopamine Antagonists: Identification of Seroquel. J. Med. Chem. 2001, 44, 372–389. 10.1021/jm000242+. 11462978

[ref4] Umemiya H.; Fukasawa H.; Ebisawa M.; Eyrolles L.; Kawachi E.; Eisenmann G.; Gronemeyer H.; Hashimoto Y.; Shudo K.; Kagechika H. Regulation of Retinoidal Actions by Diazepinylbenzoic Acids. Retinoid Synergists Which Activate the RXR-RAR Heterodimers. J. Med. Chem. 1997, 40, 4222–4234. 10.1021/jm9704309. 9435893

[ref5] aSuffert J. Simple Direct Titration of Organolithium Reagents Using N-Pivaloyl-o-Toluidine and/or N-Pivaloyi-o-Benzylaniline. J. Org. Chem. 1989, 54, 509–510. 10.1021/jo00263a052.

[ref6] aCrisenza G. E. M.; Sokolova O. O.; Bower J. F. Branch-Selective Alkene Hydroarylation by Cooperative Destabilization: Iridium-Catalyzed Ortho-Alkylation of Acetanilides. Angew. Chemie -Int. Ed. 2015, 54, 14866–14870. 10.1002/anie.201506581. PMC4691333 26490739

[ref7] aAckermann L.; Novák P. Regioselective Ruthenium-Catalyzed Direct Benzylations of Arenes through C-H Bond Cleavages. Org. Lett. 2009, 11, 4966–4969. 10.1021/ol902115f. 19810689

[ref8] aIqbal S. A.; Cid J.; Procter R. J.; Uzelac M.; Yuan K.; Ingleson M. J. Acyl-Directed Ortho -Borylation of Anilines and C7 Borylation of Indoles Using Just BBr_3_. Angew. Chemie - Int. Ed. 2019, 58, 15381–15385. 10.1002/anie.201909786. PMC6856876 31461213

[ref9] aRej S.; Chatani N. Regioselective Transition-Metal-Free C(Sp^2^)–H Borylation: A Subject of Practical and Ongoing Interest in Synthetic Organic Chemistry. Angew. Chem., Int. Ed. 2022, 61, 202209539 10.1002/anie.202209539. 35945136

[ref10] aShinde G. H.; Sundén H. Boron-Mediated Regioselective Aromatic C-H Functionalization via an Aryl BF_2_ Complex. Chem. Eur. J. 2023, 29, e202203505 10.1002/chem.202203505. 36383388

[ref11] Review: Miyaura N.; Suzuki A. Palladium-Catalyzed Cross-Coupling Reactions of Organoboron Compounds. Chem. Rev. 1995, 95, 2457–2483. 10.1021/cr00039a007.

