# Supplementary material for: Site Selective Boron Directed Ortho Benzylation of N-Aryl Amides: Access to Structurally Diversified Dibenzoazepines
Source: Org Lett. 2024 Dec 17;27(1):207–11. doi: 10.1021/acs.orglett.4c04196 (PMC11731384; doi:10.1021/acs.orglett.4c04196)
Supplement: Supplementary file 1 — ol4c04196_si_001.pdf [file ol4c04196_si_001.pdf]

Site Selective Boron directed Ortho Benzylation of N-aryl amides: Access to Structurally Diversified Dibenzoazepines

Ganesh H. Shinde,[a] Hugo Castlind,[a] Ganesh S. Ghotekar,[a] Francoise M. Amombo Noa,[b] Lars Öhrström [b] and Henrik Sundén[a]*

[a]Department of Chemistry and Molecular Biology, University of Gothenburg, SE-412 96 Gothenburg, Sweden. orcid.org/0000-0001-6202-7557;

Email: henrik.sunden@chem.gu.se

[b]Department of Chemistry and Chemical Engineering, Chalmers University of Technology, SE-41296 Gothenburg, Sweden.

Supporting Information

Table of Contents

1. General information 3
2. Experimental section 4
3. Applications 25
4. Single crystal X-ray diffraction 29
5. References 31
6. NMR spectra of target compounds 32

1. General information

Experimental procedures, reagents, and glassware: All reactions were carried out in dry glassware under a nitrogen atmosphere using standard Schlenk techniques. 5 mL screw top V-Vial® (Sigma Aldrich, Product code-Z115150-12EA) was used for the final reactions. Heating block was used for the heating. Analytically grade methanol was purchased from VWR and anhydrous dichloromethane was purchased from Sigma Aldrich. Palladium acetate was purchased from Sigma Aldrich (product code-205869). All reagents were used as received from commercial suppliers unless otherwise stated. Reported yields of final compounds are calculated based on the amide starting material without consideration of dibromoboraneyl complex formation.

Chromatography: Reaction progress was monitored by thin-layer chromatography (TLC) performed on aluminum plates coated with silica gel 60 F254 (Art 5715, 0.25 mm). Chromatograms were visualized with UV light at 254 nm or by staining using potassium permanganate. Column chromatography was performed on automated column chromatography Biotage Isolera™ Spektra One with (Biotage® Sfär Silica-10g) and (Biotage® Sfär Silica HC-10g) columns. Purifications were usually done by gradient elution using binary mixtures of pentane and ethyl acetate and UV detection at 254 nm and 280 nm.

Melting Points (MP): Melting points performed on solids were recorded on a Büchi M560 and are uncorrected.

Mass Spectrometry: High-resolution mass spectrometry (HRMS) measurements were performed using an Agilent 6520 with electrospray ionization in positive mode. Additional HRMS were recorded using an Ionoptika J105 SIMS with GCIB-SIMS ionization in both positive and negative modes.

Infrared Spectroscopy: Infrared (IR) spectra were recorded on a Perkin Elmer Paragon 100 FTIR spectrophotometer, νmax in cm^{-1}. Samples were recorded as thin films of solids. Bands are characterized as broad (br), strong (s), medium (m), and weak (w).

NMR Spectroscopy: All ^{1}H-NMR, ^{13}C-NMR, and ^{19}F-NMR spectra were recorded using Varian AV-400 MHz and 600 MHz, 700 MHz and 800 MHz bruker spectrometers at 300K. ^{11}B NMR was recorded on a 500 MHz Bruker spectrometer. Chemical shifts are given in parts per million (ppm, δ), referenced to the solvent peak of $CDCl_3$, defined at δ = 7.26 ppm (^{1}H-NMR) and δ = 77.16 ppm (^{13}C-NMR); $(CD_3)SO$ defined at δ = 2.50 ppm (^{1}H-NMR), δ = 39.52 ppm (^{13}C-NMR); CD_3OD defined at δ = 3.31 ppm (^{1}H-NMR), δ = 49.00 ppm (^{13}C-NMR). Coupling constants are quoted in Hz (*J*). ^{1}H, ^{13}C, ^{19}F, and ^{11}B NMR splitting patterns are designated as singlet (s), doublet (d), triplet (t), quartet (q), bs (broad singlet) as they appeared in the spectrum. Splitting patterns that could not be interpreted or easily visualized are designated as multiplet (m). Pivalamide starting materials,[(1,2a)] and benzanilides[(2a)] was synthesized according to literature protocol**.**

2. Experimental section

2.1 General Procedure A for reaction optimization: Benzylation of pivalamides (3a-3k)

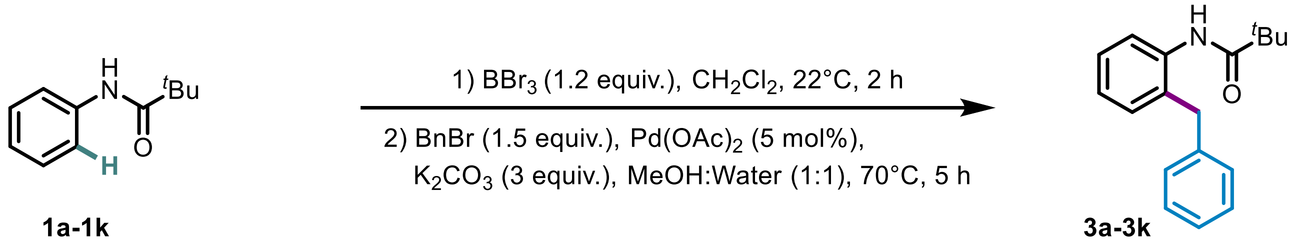

Step i) To a dry 5 mL, screw top V-Vial®, equipped with a rubber septum, stir bar, the amide derivative (0.15 mmol, 1 equiv.) in anhydrous CH_2Cl_2 (0.5 mL) under a nitrogen atmosphere was added dropwise BBr_3 (0.18 mmol, 1.2 equiv., 1M solution in CH_2Cl_2). After the complete addition of BBr_3, the reaction mixture was stirred at 22 ºC for 2 h after which the solvent was removed under reduced pressure.

Step ii) To the crude residue from step i) were added K_2CO_3 (0.45 mmol, 3 equiv.), 0.8 mL degassed methanol along with benzyl bromide (0.22 mmol, 1.5 equiv.) and 0.8 mL distilled water. Then palladium acetate (5 mol%, 0.05 equiv.) was added under nitrogen atmosphere and the reaction mixture was heated at 70 ºC for 5 h. The reaction was allowed to reach room temperature and diluted with 2 mL ethyl acetate and filtered through a pad of celite and sodium sulfate. The celite pad was washed with additional 15 mL of ethyl acetate and the filtrate was evaporated *in vacuo* to afford the crude product, which was purified using automated column chromatography (pentane/EtOAc).

2.2 Spectral data

N-(2-benzylphenyl)pivalamide (3a):[(2b)]

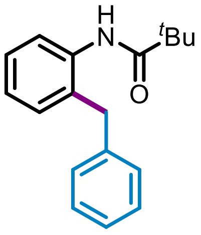

3a

Following the general procedure A using *N*-phenylpivalamide (26.59 mg, 0.15 mmol, 1 equiv.), BBr_3 (0.18 mmol, 1.2 equiv., 1M in CH_2Cl_2) at rt for 2 h and (bromomethyl)benzene (1.5 equiv., 0.22 mmol), K_2CO_3 (3 equiv., 0.45 mmol), $Pd(OAc)_2$ (0.05 equiv., 0.0075 mmol) stirred for 5 h at 70 °C. The crude product was purified by automated column chromatography (pentane/EtOAc, 8:2) and the desired product was obtained as a white solid (36.50 mg, 91%); **Rf:** 0.43 (hexane/EtOAc, 80:20); **^{1}H NMR (400 MHz, $CDCl_3$) δ=** 7.99 (dd, *J* = 8.1, 1.3 Hz, 1H), 7.34−7.29 (m, 3H), 7.26−7.22 (m, 2H), 7.16−7.12 (m, 3H), 7.03 (bs, 1H), 4.02 (s, 2H), 1.06 (s, 9H); **^{13}C{^{1}H} NMR (101 MHz, $CDCl_3$) δ=** 176.6, 138.8, 136.3, 131.3, 130.3, 129.1, 128.3, 127.8, 127.1, 124.9, 123.5, 39.6, 38.7, 27.5.

***N*-(2-Benzyl-4-(tert-butyl)phenyl)pivalamide (3b):**

Following the general procedure A using *N*-(4-(tert-butyl)phenyl)pivalamide (35 mg, 0.15 mmol, 1 equiv.) BBr_3 (0.18 mmol, 1.2 equiv., 1M in CH_2Cl_2) at rt for 2 h and benzyl bromide (1.5 equiv., 0.22 mmol), K_2CO_3 (3 equiv., 0.45 mmol), $Pd(OAc)_2$ (0.05 equiv., 0.0075 mmol) stirred for 5 h at 70 °C. The crude product was purified by automated column chromatography (pentane/EtOAc, 8:2) and the desired product was obtained as a white solid (31 mg, 64%); **Rf:** 0.5 (hexane/EtOAc, 80:20); **M.P**: 131-133 ºC; **^{1}H NMR (800 MHz, $CDCl_3$) δ** = 7.86 (d, *J* = 8.5 Hz, 1H), 7.33 – 7.29 (m, 3H), 7.25 (d, *J* = 2.4 Hz, 1H), 7.23 (t, *J* = 7.4 Hz, 1H), 7.15 – 7.12 (m, 2H), 6.94 (bs, 1H), 4.01 (s, 2H), 1.32 (s, 9H), 1.04 (s, 9H); **^{13}C{^{1}H} NMR (201 MHz, $CDCl_3$) δ=** 176.6, 147.8, 139.1, 133.7, 130.0, 129.1, 128.3, 128.3, 127.0, 124.7, 123.3, 39.5, 39.2, 34.5, 31.6, 27.5; **HRMS (ESI) (m/z):** calculated for $[M+H]^+$ $C_{22}H_{30}NO$ 324.2321; found 324.2327.

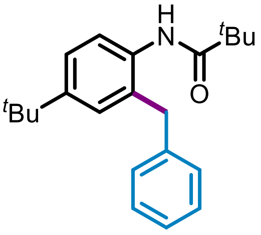

3b

***N*-(2-Benzyl-4-fluorophenyl)pivalamide (3c):**

Following the general procedure A using *N*-(4-fluorophenyl)pivalamide (29.3 mg, 0.15 mmol, 1 equiv.) BBr_3 (0.18 mmol, 1.2 equiv., 1M in CH_2Cl_2) at rt for 2 h and benzyl bromide (1.5 equiv., 0.22 mmol), K_2CO_3 (3 equiv., 0.45 mmol), $Pd(OAc)_2$ (0.05 equiv., 0.0075 mmol) stirred for 5 h at 70 °C. The crude product was purified by automated column chromatography (pentane/EtOAc, 8:2) and the desired product was obtained as a white solid (38 mg, 88%); **Rf:** 0.44 (hexane/EtOAc, 80:20); **Mp:** 114-116 ºC; **^{1}H NMR (600 MHz, $CDCl_3$) δ =** 7.84 – 7.79 (m, 1H), 7.35 – 7.29 (m, 2H), 7.25 (td, *J* = 6.6, 4.0 Hz, 1H), 7.12 (d, *J* = 7.5 Hz, 2H), 6.98 (t, *J* = 8.7 Hz, 1H), 6.93 (dd, *J* = 7.8, 4.5 Hz, 2H), 3.96 (s, 2H), 1.08 (s, 9H); **^{13}C{^{1}H} NMR (151 MHz, $CDCl_3$) δ=** 176.8, 159.9 (d, *J* = 244.1 Hz), 138.2, 133.7 (d, *J* = 7.3 Hz), 132.2, 129.2, 128.4, 127.2, 125.9 (d, *J* = 8.3 Hz), 117.7 (d, *J* = 22.7 Hz), 114.2 (d, *J* = 21.8 Hz), 39.5, 38.6, 27.5; **^{19}F NMR (564 MHz, $CDCl_3$) δ=** -117.62; **IR (neat, cm^{-1}) =** 3310 (br), 2962 (br), 2929 (br), 1651 (s), 1494 (s), 1453 (m), 1418 (m), 1366 (w), 1262 (w), 1211 (s), 1146 (m), 961 (m), 867 (w), 808 (m), 720 (s), 696 (s), 525 (s); **HRMS (ESI) (m/z):** calculated for $[M+H]^+$ $C_{18}H_{21}FNO$ 286.1601; found 286.1606.

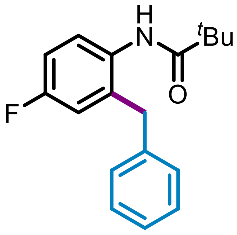

3c

***N* -(2-Benzyl-4-chlorophenyl)pivalamide (3d):**[(3)]

Following the general procedure A using *N*-(4-chlorophenyl)pivalamide (31.8 mg, 0.15 mmol, 1 equiv.) BBr_3 (0.18 mmol, 1.2 equiv., 1M in CH_2Cl_2) at rt for 2 h and benzyl bromide (1.5 equiv., 0.22 mmol), K_2CO_3 (3 equiv., 0.45 mmol), $Pd(OAc)_2$ (0.05 equiv., 0.0075 mmol) stirred for 5 h at 70 °C. The crude product was purified by automated column chromatography (pentane/EtOAc, 8:2) and the desired product was obtained as a white solid (35 mg, 76%); **Rf:** 0.57 (hexane/EtOAc, 80:20); **^{1}H NMR (600 MHz, $CDCl_3$) δ =**

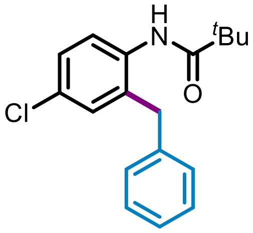

3d

7.95 (d, *J* = 8.7 Hz, 1H), 7.36 – 7.30 (m, 2H), 7.27 (dd, *J* = 8.4, 2.5 Hz, 2H), 7.23 (d, *J* = 2.8 Hz, 1H), 7.13 (d, *J* = 7.5 Hz, 2H), 6.98 (bs, 1H), 3.97 (s, 2H), 1.05 (s, 9H); **^{13}C{^{1}H} NMR (151 MHz, $CDCl_3$) δ=** 176.7, 137.9, 135.0, 132.2, 130.9, 129.8, 129.3, 128.3, 127.8, 127.4, 124.7, 39.7, 38.5, 27.4; **IR (neat, cm^{-1}) =** 3312 (br), 2962 (br), 2928 (br), 1652 (s), 1505 (s), 1480 (s), 1453 (m), 1401 (m), 1367 (w), 1299 (w), 1178 (m), 1112 (w), 923 (w), 906 (w), 808 (m), 724 (m), 697 (m), 671 (m).

N **-(2-Benzyl-4-Bromophenyl)pivalamide (3e):**

Following the general procedure A using *N*-(4-bromophenyl)pivalamide (38.4 mg, 0.15 mmol, 1 equiv.) BBr_3 (0.18 mmol, 1.2 equiv., 1M in CH_2Cl_2) at rt for 2 h and benzyl bromide (1.5 equiv., 0.22 mmol),

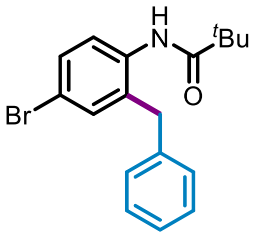

3e

K_2CO_3 (3 equiv., 0.45 mmol), $Pd(OAc)_2$ (0.05 equiv., 0.0075 mmol) stirred for 5 h at 70 °C. The crude product was purified by automated column chromatography (pentane/EtOAc, 8:2) and the desired product was obtained as a white solid (29 mg, 55%); **Rf:** 0.57 (hexane/EtOAc, 80:20); **Mp:** 139-141 °C; **^{1}H NMR (600 MHz, $CDCl_3$) δ =** 7.92 (d, *J* = 8.6 Hz, 1H), 7.43 (dd, *J* = 8.7, 2.5 Hz, 1H), 7.40 (d, *J* = 2.5 Hz, 1H), 7.34 (t, *J* = 7.7 Hz, 2H), 7.28 (d, *J* = 7.2 Hz, 1H), 7.14 (d, *J* = 7.2 Hz, 2H), 7.00 (s, 1H), 3.98 (s, 2H), 1.05 (s, 9H); **^{13}C{^{1}H} NMR (151 MHz, $CDCl_3$) δ=** 176.7, 138.0, 136.1, 133.8, 132.4, 130.7, 129.3, 128.3, 127.4, 124.9, 117.6, 39.7, 38.5, 27.54; **HRMS (ESI) (m/z):** calculated for $[M+H]^+$ $C_{18}H_{21}BrNO$ 346.0801; found 346.0806.

***N*-(2-Benzyl-5-bromophenyl)pivalamide (3f):**

Following the general procedure A using *N*-(3-bromophenyl)pivalamide (38.4 mg, 0.15 mmol, 1 equiv.) BBr_3 (0.18 mmol, 1.2 equiv., 1M in CH_2Cl_2) at rt for 2 h and benzyl bromide (1.5 equiv., 0.22 mmol),

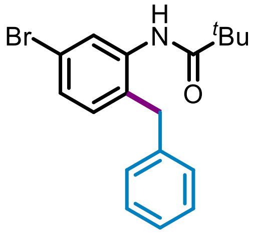

3f

K_2CO_3 (3 equiv., 0.45 mmol), $Pd(OAc)_2$ (0.05 equiv., 0.0075 mmol) stirred for 5 h at 70 °C. The crude product was purified by automated column chromatography (pentane/EtOAc, 8:2) and the desired product was obtained as a white solid (37 mg, 71%); **Rf:** 0.62 (hexane/EtOAc, 80:20); **Mp:** 111-113 °C; **^{1}H NMR (600 MHz, $CDCl_3$) δ=** 8.30 (d, *J* = 2.1 Hz, 1H), 7.32 (dd, *J* = 8.3, 6.9 Hz, 2H), 7.28 – 7.23 (m, 2H), 7.13 – 7.08 (m, 3H), 7.03 (bs, 1H), 3.97 (s, 2H), 1.03 (s, 9H); **^{13}C{^{1}H} NMR (151 MHz, $CDCl_3$) δ=** 176.7, 137.9, 137.6, 132.4, 129.3, 128.7, 128.3, 127.6, 127.3, 125.9, 121.3, 39.7, 38.3, 27.4; **IR (neat, cm^{-1}) =** 3311 (br), 2955 (m), 2922 (s), 2852 (m), 1654 (s), 1576 (m), 1506 (s), 1495 (s), 1478 (s), 1453 (s), 1406 (s), 1244 (w), 1158 (s), 1111 (w), 1074 (w), 1029 (w), 938 (w), 896 (w), 732 (s), 696 (s); **HRMS (ESI) (m/z):** calculated for $[M+H]^+$ $C_{18}H_{21}BrNO$ 346.0801; found 346.0806.

***N*-(4-Benzyl-[1,1'-biphenyl]-3-yl)pivalamide (3g):**

Following the general procedure A using *N*-([1,1'-biphenyl]-3-yl)pivalamide (38.0 mg, 0.15 mmol, 1 equiv.) BBr_3 (0.18 mmol, 1.2 equiv., 1M in CH_2Cl_2) at rt for 2 h and benzyl bromide (1.5 equiv., 0.22 mmol), K_2CO_3 (3 equiv., 0.45 mmol), $Pd(OAc)_2$ (0.05 equiv., 0.0075 mmol) stirred for 16 h at 70 °C. The crude product was purified by automated column chromatography (pentane/EtOAc, 8:2) and the desired product was obtained as a white solid (34 mg, 66%); **Rf:** 0.43 (hexane/EtOAc, 80:20); **Mp:** 124-126 ºC; **^{1}H NMR (800 MHz, $CDCl_3$) δ** = 8.32 (d, *J* = 1.9 Hz, 1H), 7.67 – 7.62 (m, 2H), 7.41 (t, *J* = 7.7 Hz, 2H), 7.37 (dd, *J* = 7.8, 1.9 Hz, 1H), 7.34 – 7.31 (m, 3H), 7.30 (d, *J* = 7.7 Hz, 1H), 7.28 – 7.23 (m, 1H), 7.18 (d, *J* = 8.1 Hz, 2H), 7.10 (bs, 1H), 4.05 (s, 2H), 1.07 (s, 9H); **$^{13}C\{^{1}H\}$ NMR (201 MHz, $CDCl_3$) δ=** 176.8, 140.9, 140.7, 138.7, 136.7, 131.6, 129.2, 129.1, 128.8, 128.4, 127.4, 127.3, 127.1, 123.3, 122.1, 39.7, 38.5, 27.5; **IR (neat, cm^{-1}) =** 3331 (br), 2966 (br), 1647 (s), 1502 (s), 1487 (s), 1451 (m), 1363 (w), 1205 (w), 1179 (w), 1166 (w), 1075 (w), 1029 (w), 947 (w), 887 (m), 843 (m), 762 (s), 725 (s), 695 (s), 609 (m); **HRMS (ESI) (m/z):** calculated for $[M+H]^+$ $C_{24}H_{26}NO$ 344.2008; found 344.2015.

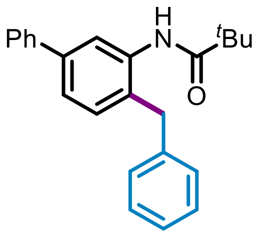

3g

***N*-(2-Benzyl-6-methylphenyl)pivalamide (3h):**

Following the general procedure A using *N*-(o-tolyl)pivalamide (28.7 mg, 0.15 mmol, 1 equiv.) BBr_3 (0.18 mmol, 1.2 equiv., 1M in CH_2Cl_2) at rt for 2 h and benzyl bromide (1.5 equiv., 0.22 mmol), K_2CO_3 (3 equiv., 0.45 mmol), $Pd(OAc)_2$ (0.05 equiv., 0.0075 mmol) stirred for 5 h at 70 °C. The crude product was purified by automated column chromatography (pentane/EtOAc, 8:2) and the desired product was obtained as a white solid (29.0 mg, 69%); **Rf:** 0.38 (hexane/EtOAc, 80:20); **Mp:** 132-134 ºC; **^{1}H NMR (800 MHz, $CDCl_3$) δ =** 7.29 (t, *J* = 7.5 Hz, 2H), 7.21 (t, *J* = 7.4 Hz, 1H), 7.16 (q, *J* = 4.5 Hz, 2H), 7.10 (d, *J* = 7.6 Hz, 2H), 7.07 (dd, *J* = 6.3, 2.8 Hz, 1H), 6.71 (s, 1H), 3.94 (s, 2H), 2.19 (s, 3H), 1.20 (s, 9H); **$^{13}C\{^{1}H\}$ NMR (201 MHz, $CDCl_3$) δ** = 176.5, 140.4, 137.4, 136.5, 134.3, 129.3, 128.8, 128.7, 128.5, 127.4, 126.4, 39.3, 38.7, 27.7, 18.5; **IR (neat, cm^{-1}) =** 3344 (s), 2953 (br), 2922 (br), 1651 (s), 1496 (s), 1472 (s), 1389 (w), 1357 (w), 1261 (w), 1218 (m), 1167 (m), 1032 (w), 927 (w), 772 (s), 744 (s), 727 (s), 702 (s), 619 (s); **HRMS (ESI) (m/z):** calculated for $[M+H]^+$ $C_{19}H_{24}NO$ 282.1852; found 282.1857.

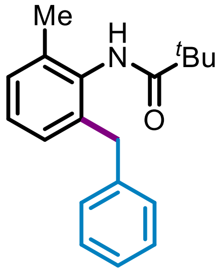

3h

***N*-(2-Benzyl-6-fluorophenyl)pivalamide (3i):**

Following the general procedure A using *N*-(2-fluorophenyl)pivalamide (29.3 mg, 0.15 mmol, 1 equiv.) BBr_3 (0.18 mmol, 1.2 equiv., 1M in CH_2Cl_2) at rt for 2 h and benzyl bromide (1.5 equiv., 0.22 mmol), K_2CO_3 (3 equiv., 0.45 mmol), $Pd(OAc)_2$ (0.05 equiv., 0.0075 mmol) stirred for 5 h at 70 °C. The crude product was purified by automated column

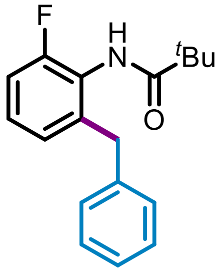

3i

chromatography (pentane/EtOAc, 8:2) and the desired product was obtained as a white solid (25.0 mg, 59%); **Rf:** 0.50 (hexane/EtOAc, 80:20); **Mp:** 139-141 °C; **^{1}H NMR (600 MHz, CDCl$_3$) δ =** 7.29 (t, *J* = 7.6 Hz, 2H), 7.22 (t, *J* = 7.5 Hz, 1H), 7.20 – 7.16 (m, 1H), 7.11 (dd, *J* = 7.3, 1.6 Hz, 2H), 7.02 (ddd, *J* = 9.6, 8.3, 1.3 Hz, 1H), 6.99 (d, *J* = 7.7 Hz, 1H), 6.71 (bs, 1H), 3.97 (s, 2H), 1.23 (s, 9H); **^{13}C{^{1}H} NMR (151 MHz, CDCl$_3$) δ** = 177.2, 158.2 (d, *J* = 248.2 Hz), 140.1, 139.8, 128.8, 128.7, 127.9 (d, *J* = 8.6 Hz), 126.6, 126.1 (d, *J* = 3.2 Hz), 123.68 (d, *J* = 13.1 Hz), 114.29 (d, *J* = 20.9 Hz) 39.4, 38.2, 27.7; **^{19}F NMR (564 MHz, CDCl$_3$) δ**= -120.70; **IR (neat, cm^{-1}) =** 3231 (br), 2955 (m), 2921 (s), 2851 (m), 1648 (s), 1521 (s), 1468 (s), 1457 (s), 1403 (w), 1370 (w), 1294 (w), 1266 (m), 1222 (m), 1179 (m), 1075 (w), 1028 (w), 974 (m), 939 (m), 770 (s), 717 (s), 709 (s); **HRMS (ESI) (m/z):** calculated for [M+H]$^+$ $C_{18}H_{21}FNO$ 286.1601; found 286.1606.

***N*-(2-Benzylnaphthalen-1-yl)pivalamide (3j):**

Following the general procedure A using *N*-(naphthalen-1-yl)pivalamide (34.0 mg, 0.15 mmol, 1 equiv.) BBr$_3$ (0.18 mmol, 1.2 equiv., 1M in CH_2Cl_2) at rt for 2 h and benzyl bromide (1.5 equiv., 0.22 mmol), K_2CO_3 (3 equiv., 0.45 mmol), Pd(OAc)$_2$ (0.05 equiv., 0.0075 mmol) stirred for 16 h at 70 °C. The crude product was purified by automated column chromatography (pentane/EtOAc, 8:2) and the desired product was obtained as a white solid (37 mg, 77%); **Rf:** 0.34 (hexane/EtOAc, 80:20); **Mp:** 172-174 °C; **^{1}H NMR (600 MHz, CDCl$_3$) δ** = 7.84 (d, J = 8.1 Hz, 1H), 7.77 (d, *J* = 8.5 Hz, 2H), 7.51 (ddd, *J* = 8.3, 6.8, 1.4 Hz, 1H), 7.47 (t, *J* = 7.4 Hz, 1H), 7.34 (d, *J* = 8.4 Hz, 1H), 7.29 (t, *J* = 7.6 Hz, 2H), 7.22 (t, *J* = 7.4 Hz, 1H), 7.18 – 7.11 (m, 3H), 4.11 (s, 2H), 1.34 (s, 9H); **^{13}C{^{1}H} NMR (151 MHz, CDCl$_3$) δ =** 177.5, 140.4, 135.6, 133.3, 131.1, 130.7, 128.8, 128.7, 128.6, 128.3, 127.9, 126.8, 126.4, 125.7, 122.6, 39.5, 38.4, 27.8; **IR (neat, cm^{-1}) =** 3283 (br), 2957 (br), 2924 (br), 2868 (br), 1647 (m), 1495 (s), 1399 (w), 1385 (w), 1367 (w), 1196 (w), 1170 (w), 1029 (w), 933 (w), 801 (w), 759 (w), 740 (w), 710 (w), 697 (m), 661 (w); **HRMS (ESI) (m/z):** calculated for [M+H]$^+$ $C_{22}H_{24}NO$ 318.1852 found 318.1857.

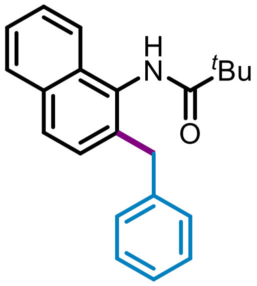

3j

***N,N'*-(oxybis(2-Benzyl-4,1-phenylene))bis(2,2-dimethylpropanamide)) (3k):**

Following the general procedure A using *N,N'*-(oxybis(4,1-phenylene))bis(2,2-dimethylpropanamide) (55.3 mg, 0.15 mmol, 1 equiv.) BBr$_3$ (0.36 mmol, 2.4 equiv., 1M in CH_2Cl_2) at rt for 2 h and benzyl bromide (3.0 equiv., 0.45 mmol), K_2CO_3 (6 equiv., 0.9 mmol), Pd(OAc)$_2$ (0.10 equiv., 0.015 mmol) stirred for 5 h at 70 °C. The crude product was purified by automated column chromatography (pentane/EtOAc, 8:2) and the desired product was obtained as a white solid (66 mg, 80%); **Rf:** 0.46 (hexane/EtOAc, 60:40); **Mp:** 191-193 °C; **^{1}H NMR (800 MHz, CDCl$_3$) δ** = 7.83 (d, *J* =

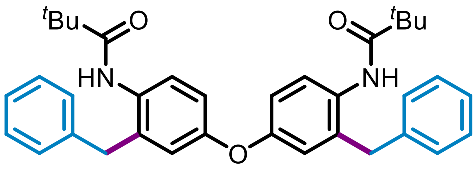

3k

9.6 Hz, 2H), 7.31 (t, J = 7.6 Hz, 4H), 7.24 (d, J = 7.5 Hz, 2H), 7.14 (d, J = 6.9 Hz, 4H), 6.97 – 6.93 (m, 6H), 3.96 (s, 4H), 1.08 (s, 18H); **$^{13}C\{^1H\}$ NMR (201 MHz, $CDCl_3$) δ=** 176.8, 154.3, 138.5, 133.3, 131.6, 129.2, 128.3, 127.1, 125.6, 121.5, 117.6, 39.5, 38.7, 27.5; **IR (neat, cm^{-1}) =** 3423 (br), 3234 (br), 2957 (br), 1682 (s), 1647 (s), 1513 (s), 1493 (s), 1477 (s), 1424 (s), 1281 (m), 1214 (s), 1183 (m), 1158 (m), 1028 (w), 982 (m), 881 (w), 868 (w), 826 (m), 805 (m), 737 (s), 725 (s), 696 (s); **HRMS (ESI) (m/z):** calculated for $[M+H]^+$ $C_{36}H_{41}N_2O_3$ 549.3111 found 549.3121.

2.3 General Procedure B for reaction optimization:Benzylation of benzanilides and urea (3l-3q)

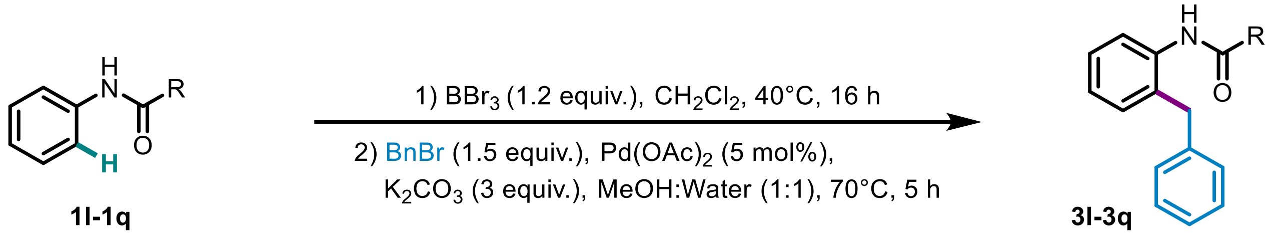

Step i) To a dry 5 mL, screw top V-Vial®, equipped with a rubber septum, stir bar, the amide derivative (0.15 mmol, 1 equiv.) in anhydrous CH_2Cl_2 (0.5 mL) under a nitrogen atmosphere was added dropwise BBr_3 (0.18 mmol, 1.2 equiv., 1M solution in CH_2Cl_2). After the complete addition of BBr_3, the reaction mixture was stirred at 40 ºC for 16 h after which the solvent was removed under reduced pressure.

Step ii) To the crude residue from step i) were added K_2CO_3 (0.45 mmol, 3 equiv.), 0.8 mL degassed methanol along with benzyl bromide (0.22 mmol, 1.5 equiv.) and 0.8 mL distilled water. Then palladium acetate (5 mol%, 0.05 equiv.) was added under nitrogen atmosphere and the reaction mixture was heated at 70 ºC for 5 h. The reaction was allowed to reach room temperature and diluted with 2 mL ethyl acetate and filtered through a pad of celite and sodium sulfate. The celite pad was washed with additional 15 mL of ethyl acetate and the filtrate was evaporated *in vacuo* to afford the crude product, which was purified using automated column chromatography (pentane/EtOAc).

2.4 Spectral data

***N*-(2-benzylphenyl)benzamide (3l):**[5]

Following the general procedure B using *N*-phenylbenzamide (29.6 mg, 0.15 mmol, 1 equiv.) BBr_3 (0.18 mmol, 1.2 equiv., 1M in CH_2Cl_2) at 40 °C for 16 h and benzyl bromide (1.5 equiv., 0.22 mmol), K_2CO_3 (3 equiv., 0.045 mmol), $Pd(OAc)_2$ (0.05 equiv., 0.0075 mmol) stirred for 5 h at 70 °C. The crude product was purified by automated column chromatography (pentane/EtOAc, 8:2) and the desired product was obtained as a white solid (39 mg, 90%); **Rf:** 0.45 (hexane/EtOAc, 80:20); **^1H NMR (600 MHz, $CDCl_3$) δ** = 8.09 (d, J = 8.0 Hz, 1H), 7.62 (s, 1H), 7.51 – 7.47 (m, 1H), 7.47 – 7.41 (m, 2H), 7.38 – 7.33 (m, 5H), 7.33 – 7.28 (m, 2H), 7.23 – 7.17 (m, 3H), 4.08 (s, 2H); **$^{13}C\{^1H\}$ NMR (151 MHz, $CDCl_3$) δ=** 165.4, 138.9, 136.3, 134.7, 131.8, 131.3,

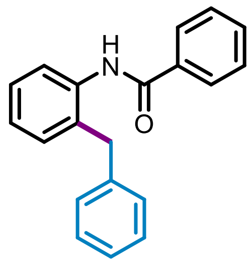

3l

131.0, 129.3, 128.7, 128.6, 127.9, 127.2, 127.0, 125.3, 123.5, 39.0.

***N*-(2-Benzylphenyl)-3-methylbenzamide (3m):**

Following the general procedure B using 3-methyl-*N*-phenylbenzamide (31.7 mg, 0.15 mmol, 1 equiv.) BBr_3 (0.18 mmol, 1.2 equiv., 1M in CH_2Cl_2) at 40 °C for 16 h and benzyl bromide (1.5 equiv., 0.22 mmol), K_2CO_3 (3 equiv., 0.045 mmol), $Pd(OAc)_2$ (0.05 equiv., 0.0075 mmol) stirred for 5 h at 70 °C. The crude product was purified by automated column chromatography (pentane/EtOAc, 8:2) and the desired product was obtained as a white solid (42 mg, 94%); **Rf:** 0.42 (hexane/EtOAc, 80:20); **Mp:** 106-108 ºC; **^{1}H NMR (600 MHz, $CDCl_3$) δ** = 8.11 (d, *J* = 8.1 Hz, 1H), 7.60 (bs, 1H), 7.39 – 7.35 (m, 3H), 7.34 – 7.29 (m, 3H), 7.27 (t, *J* = 1.1 Hz, 2H), 7.23 – 7.19 (m, 4H), 4.09 (s, 2H), 2.35 (s, 3H); **$^{13}C\{^1H\}$ NMR (151 MHz, $CDCl_3$) δ =** 165.5, 138.9, 138.5, 136.4, 134.7, 132.6, 131.3, 130.8, 129.3, 128.6 (2C), 128.0, 127.5, 127.2, 125.2, 124.2, 123.5, 39.0, 21.4; **IR (neat, cm^{-1}) =** 3283 (br), 2953 (br), 2922 (br), 2853 (br), 1646 (s), 1586 (s), 1516 (s), 1493 (s), 1449 (s), 1303 (s), 1268 (m), 1246 (w), 1195 (w), 1074 (w), 1029 (w), 732 (s), 697 (s), 668 (m); **HRMS (ToF-SIMS) (m/z):** calculated for $[M+H]^+$ $C_{21}H_{20}NO$ 302.1539; found 302.1540.

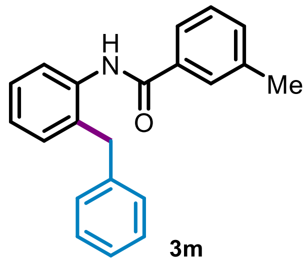

3m

***N*-(2-Benzylphenyl)-4-fluorobenzamide (3n):**

Following the general procedure B using 4-Fluoro-*N*-phenylbenzamide (32.3 mg, 0.15 mmol, 1 equiv.) BBr_3 (0.18 mmol, 1.2 equiv., 1M in CH_2Cl_2) at 40 °C for 16 h and benzyl bromide (1.5 equiv., 0.22 mmol), K_2CO_3 (3 equiv., 0.045 mmol), $Pd(OAc)_2$ (0.05 equiv., 0.0075 mmol) stirred for 5 h at 70 °C. The crude product was purified by automated column chromatography (pentane/EtOAc, 8:2) and the desired product was obtained as a white solid (37 mg, 81%); **Rf:** 0.47 (hexane/EtOAc, 80:20); **Mp:** 129-131 ºC; **^{1}H NMR (600 MHz, $CDCl_3$) δ =** 8.05 (d, *J* = 8.0 Hz, 1H), 7.52 (bs, 1H), 7.39 (dd, *J* = 8.6, 5.3 Hz, 2H), 7.37 – 7.33 (m, 3H), 7.32 (t, *J* = 7.7 Hz, 2H), 7.21 (d, *J* = 7.5 Hz, 1H), 7.19 (d, *J* = 7.7 Hz, 2H), 7.02 (t, *J* = 8.6 Hz, 2H), 4.07 (s, 2H); **$^{13}C\{^1H\}$ NMR (151 MHz, $CDCl_3$) δ =** 164.9 (d, *J* = 252.45 Hz), 164.3, 138.9, 136.2, 131.4, 130.9 (d, *J* = 7.2 Hz), 129.4, 129.4, 129.3, 128.6, 128.0, 127.2, 125.4, 123.5, 115.7 (d, *J* = 21.9 Hz), 39.1; **^{19}F NMR (564 MHz, CDCl3) δ** = -107.69; **IR (neat, cm^{-1}) =** 3276 (br), 3062 (br), 3027 (br), 2954 (br), 2924 (br), 2853 (br), 1645 (s), 1602 (s), 1525 (m), 1501 (s), 1450 (s), 1313 (w), 1293 (w), 1232 (s), 1159 (m), 848 (m), 761 (m), 750 (m), 733 (m), 698 (m); **HRMS (ToF-SIMS) (m/z):** calculated for $[M+H]^+$ $C_{20}H_{17}FNO$ 306.1288; found 306.1290.

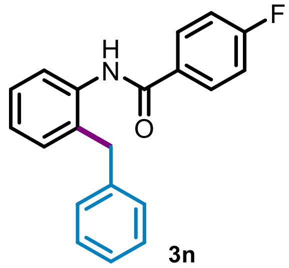

3n

***N*-(2-Benzylphenyl)-4-(trifluoromethyl)benzamide (3o):**

Following the general procedure B using *N*-phenyl-4-(trifluoromethyl)benzamide (39.8 mg, 0.15 mmol, 1 equiv.) BBr_3 (0.18 mmol, 1.2 equiv., 1M in CH_2Cl_2) at 40 °C for 16 h and benzyl bromide (1.5 equiv., 0.22 mmol), K_2CO_3 (3 equiv., 0.045 mmol), $Pd(OAc)_2$ (0.05 equiv., 0.0075 mmol) stirred for 5 h at 70 °C. The crude product was purified by automated column chromatography (pentane/EtOAc, 8:2) and the desired product was obtained as a white solid (36 mg, 68%); **Rf:** 0.43 (hexane/EtOAc, 80:20); **Mp:** 175-177 °C; **^{1}H NMR (600 MHz, $CDCl_3$) δ** = 8.08 (d, *J* = 8.1 Hz, 1H), 7.61 (d, *J* = 8.2 Hz, 2H), 7.59 (bs, 1H), 7.47 (d, *J* = 8.0 Hz, 2H), 7.39 – 7.31 (m, 5H), 7.23 (t, *J* = 7.5 Hz, 1H), 7.19 (d, *J* = 7.4 Hz, 2H), 4.08 (s, 2H); **^{13}C{^{1}H} NMR (151 MHz, $CDCl_3$) δ =** 164.0, 138.8, 138.0, 136.0, 133.5 (q, *J* = 32.5 Hz), 131.5, 130.9, 129.4, 128.6, 128.1, 127.5, 127.4, 125.7, 125.4 (q, *J* = 4.1 Hz), 123.7 (d, *J* = 272.5 Hz), 122.8, 39.2; **^{19}F NMR (564 MHz, $CDCl_3$) δ** -62.98; **IR (neat, cm^{-1}) =** 3218 (br), 3028 (br), 1638 (s), 1536 (s), 1495 (w), 1333 (s), 1309 (m), 1158 (m), 1128 (s), 1069 (s), 1018 (w), 919 (w), 855 (m), 758 (s), 733 (s), 667 (m); **HRMS (ToF-SIMS) (m/z):** calculated for $[M]^+$ $C_{21}H_{16}F_3NO$ 355.1184; found 355.1185.

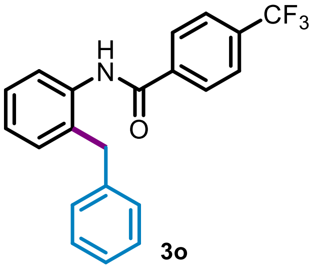

3o

***N*-(2-Benzylphenyl)furan-2-carboxamide (3p):**

Following the general procedure B using *N*-phenylfuran-2-carboxamide (28.1 mg, 0.15 mmol, 1 equiv.) BBr_3 (0.18 mmol, 1.2 equiv., 1M in CH_2Cl_2) at 40 °C for 16 h and benzyl bromide (1.5 equiv., 0.22 mmol), K_2CO_3 (3 equiv., 0.045 mmol), $Pd(OAc)_2$ (0.05 equiv., 0.0075 mmol) stirred for 5 h at 70 °C. The crude product was purified by automated column chromatography (pentane/EtOAc, 8:2) and the desired product was obtained as a white solid (35 mg, 84%); **Rf:** 0.26 (hexane/EtOAc, 80:20); **Mp:** 111-113 °C; **^{1}H NMR (600 MHz, $CDCl_3$) δ** = 8.05 (dd, *J* = 8.1, 1.3 Hz, 1H), 7.89 (bs, 1H), 7.41 (d, *J* = 2.4 Hz, 1H), 7.36 – 7.30 (m, 3H), 7.28 (d, *J* = 7.6 Hz, 1H), 7.26 – 7.21 (m, 3H), 7.18 (td, *J* = 7.5, 1.3 Hz, 1H), 7.12 (d, *J* = 3.5 Hz, 1H), 6.51 (dd, *J* = 3.5, 1.7 Hz, 1H), 4.06 (s, 2H); **^{13}C{^{1}H} NMR (151 MHz, $CDCl_3$) δ=** 156.2, 148.0, 144.2, 138.9, 135.5, 131.5, 131.0, 129.0, 128.8, 127.8, 127.0, 125.4, 123.5, 115.1, 112.6, 38.7; **IR (neat, cm^{-1}) =** 3396 (br), 2922 (br), 1677 (s), 1582 (s), 1523 (s), 1450 (s), 1309 (s), 1262 (m), 1160 (m), 1070 (w), 1013 (m), 931 (w), 760 (s), 738 (s), 700 (s), 624 (m), 585 (s); **HRMS (ESI) (m/z):** calculated for $[M+H]^+$ $C_{18}H_{16}NO_2$ 278.1175; found 278.118.

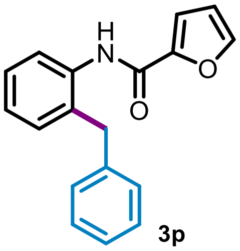

3p

***N*-(2-benzylphenyl)thiophene-2-carboxamide (3q):**

Following the general procedure B using *N*-phenylthiophene-2-carboxamide (30.5 mg, 0.15 mmol, 1 equiv.) BBr_3 (0.18 mmol, 1.2 equiv., 1M in CH_2Cl_2) at 40 °C for 16 h and benzyl bromide (1.5 equiv., 0.22 mmol), K_2CO_3 (3 equiv., 0.045 mmol), $Pd(OAc)_2$ (0.05 equiv., 0.0075 mmol) stirred for 5 h at 70 °C. The crude product was purified by automated column chromatography (pentane/EtOAc, 8:2) and the desired product was obtained as a white solid (38 mg, 86%); **Rf:** 0.23 (hexane/EtOAc, 80:20); **Mp:** 99-101 ºC; **^{1}H NMR (600 MHz, $CDCl_3$) δ** = 8.01 (d, *J* = 8.1 Hz, 1H), 7.48 (dd, *J* = 4.8, 1.3 Hz, 1H), 7.42 (bs, 1H), 7.38 – 7.34 (m, 3H), 7.33 – 7.28 (m, 2H), 7.23 – 7.18 (m, 3H), 7.02 – 6.98 (m, 2H), 4.08 (s, 2H); **^{13}C{^{1}H} NMR (151 MHz, $CDCl_3$) δ =** 159.8, 139.5, 138.8, 136.0, 131.3, 131.1, 130.8, 129.3, 128.6, 128.2, 128.0, 127.8, 127.3, 125.4, 123.8, 39.0; **IR (neat, cm^{-1}) =** 3321 (br), 2922 (br), 1628 (s), 1585 (w), 1532 (m), 1513 (m), 1451 (m), 1417 (w), 1354 (w), 1302 (m), 1272 (m), 1247 (w), 1108 (w), 749 (s), 722 (s), 699 (s), 592 (m); **HRMS (ToF-SIMS) (m/z):** calculated for $[M+H]^+$ $C_{18}H_{16}NOS$ 294.0947; found 294.0947.

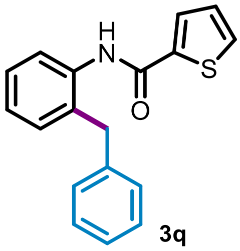

3q

2.5 General Procedure C for reaction optimization: Scope of Benzylation (4a-4n)

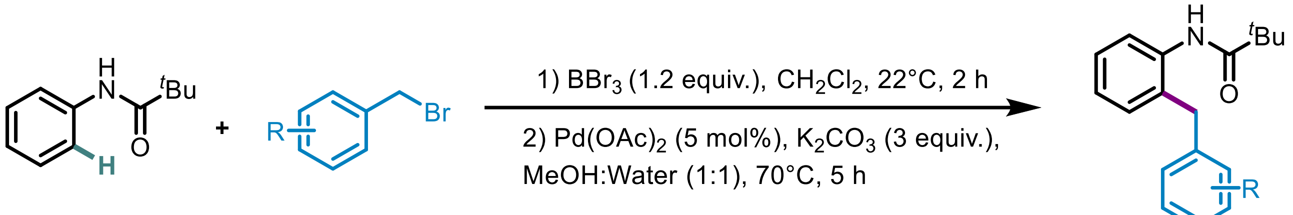

Step i) To a dry 5 mL, screw top V-Vial®, equipped with a rubber septum, stir bar, the *N*-phenylpivalamide (26.6 mg, 0.15 mmol, 1 equiv.) in anhydrous CH_2Cl_2 (0.5 mL) under a nitrogen atmosphere was added dropwise BBr_3 (0.18 mmol, 1.2 equiv., 1M solution in CH_2Cl_2). After the complete addition of BBr_3, the reaction mixture was stirred at 22 ºC for 2 h after which the solvent was removed under reduced pressure.

Step ii) To the crude residue from step i) were added K_2CO_3 (0.45 mmol, 3 equiv.), 0.8 mL degassed methanol along with substituted benzyl bromide (0.22 mmol, 1.5 equiv.) and 0.8 mL distilled water. Then palladium acetate (5 mol%, 0.05 equiv.) was added under nitrogen atmosphere and the reaction mixture was heated at 70 ºC for 5 h. The reaction was allowed to reach room temperature and diluted with 2 mL ethyl acetate and filtered through a pad of celite and sodium sulfate. The celite pad was washed with additional 15 mL of ethyl acetate and the filtrate was evaporated *in vacuo* to afford the crude product, which was purified using automated column chromatography (pentane/EtOAc).

2.6 Spectral data

_N_-(2-(3-methoxybenzyl)phenyl)pivalamide (4a):

Following the general procedure C using *N*-phenylpivalamide (26.6 mg, 0.15 mmol, 1 equiv.) BBr_3 (0.18 mmol, 1.2 equiv., 1M in CH_2Cl_2) at rt for 2 h and 3-Methoxybenzyl bromide (1.5 equiv., 0.22 mmol), K_2CO_3 (3 equiv., 0.45 mmol), $Pd(OAc)_2$ (0.05 equiv., 0.0075 mmol) stirred for 5 h at 70 °C. The crude product was purified by automated column chromatography (pentane/EtOAc, 8:2) and the desired product was obtained as a white solid (37 mg, 83%); **Rf:** 0.41 (hexane/EtOAc, 80:20); **Mp:** 93-95 ºC; **^{1}H NMR (600 MHz, $CDCl_3$) δ =** 7.96 (d, J = 8.1 Hz, 1H), 7.30 (td, J = 7.8, 1.6 Hz, 1H), 7.25 – 7.21 (m, 2H), 7.13 (td, J = 7.5, 1.3 Hz, 1H), 7.06 (s, 1H), 6.78 (dd, J = 8.2, 2.6 Hz, 1H), 6.73 (d, J = 7.6 Hz, 1H), 6.67 (t, J = 2.0 Hz, 1H), 3.98 (s, 2H), 3.75 (s, 3H), 1.09 (s, 9H); **^{13}C{^{1}H} NMR (151 MHz, $CDCl_3$) δ=** 176.7, 160.3, 140.5, 136.4, 131.2, 130.4, 130.1, 127.8, 124.9, 123.6, 120.7, 114.2, 112.2, 55.3, 39.6, 38.7, 27.5; **IR (neat, cm^{-1}) =** 3352 (br), 2932 (br), 1657 (s), 1592 (s), 1513 (s), 1487 (m), 1449 (s), 1397 (w), 1293 (w), 1262 (s), 1164 (s), 1033 (s), 936 (w), 924 (w), 870 (m), 801 (m), 773 (s), 761 (s), 747 (s), 716 (m), 697 (s), 631 (m), 563 (m); **HRMS (ToF-SIMS) (m/z):** calculated for $[M]^+$ $C_{19}H_{23}NO_2$ 297.1729; found 297.1728.

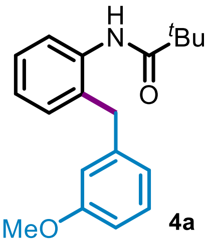

4a

N-(2-(4-Fluorobenzyl)phenyl)pivalamide (4b):

Following the general procedure C using *N*-phenylpivalamide (26.6 mg, 0.15 mmol, 1 equiv.) BBr_3 (0.18 mmol, 1.2 equiv., 1M in CH_2Cl_2) at rt for 2 h and 4-Fluorobenzyl bromide (1.5 equiv., 0.22 mmol), K_2CO_3 (3 equiv., 0.45 mmol), $Pd(Ac)_2$ (0.05 equiv., 0.0075 mmol) stirred for 5 h at 70 °C. The crude product was purified by automated column chromatography (pentane/EtOAc, 8:2) and the desired product was obtained as a white solid (35 mg, 81%); **Rf:** 0.41 (Hexane/EtOAc, 80:20); **Mp:** 112-114 °C; **^{1}H NMR (600 MHz, $CDCl_3$) δ=** 7.94 (dd, *J* = 8.6, 2.5 Hz, 1H), 7.34 – 7.28 (m, 1H), 7.21 (d, *J* = 7.5 Hz, 1H), 7.14 (dt, *J* = 8.1, 3.4 Hz, 1H), 7.12 – 7.07 (m, 2H), 7.05 – 6.96 (m, 3H), 3.97 (s, 2H), 1.10 (s, 9H); **^{13}C NMR (151 MHz, $CDCl_3$) δ=** 176.6, 161.9 (d, *J* = 245.5 Hz), 136.2, 134.5 (d, *J* = 3.2 Hz), 131.1, 130.5, 129.8 (d, *J* = 7.8 Hz), 128.0, 125.1, 123.8, 115.9 (d, *J* = 21.3 Hz), 39.6, 37.8, 27.5; **^{19}F NMR (471 MHz, $CDCl_3$) δ=** -115.89; **IR (neat, cm^{-1}) =** 3321 (br), 2959 (br), 2921 (br), 2852 (br), 1648 (s), 1506 (s), 1450 (s), 1216 (s), 1157 (m), 827 (w), 812 (m), 770 (s), 750 (s), 620 (m); **HRMS (ToF-SIMS) (m/z):** calculated for $[M+H]^+$ $C_{18}H_{21}FNO$ 286.1601; found 286.1602.

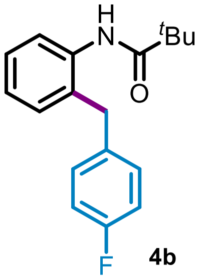

4b

N-(2-(4-Bromobenzyl)phenyl)pivalamide (4c): [(3)]

Following the general procedure C using *N*-phenylpivalamide (26.6 mg, 0.15 mmol, 1 equiv.) BBr_3 (0.18 mmol, 1.2 equiv., 1M in CH_2Cl_2) at rt for 2 h and 4-Bromobenzyl bromide (1.5 equiv., 0.22 mmol), K_2CO_3 (3 equiv., 0.45 mmol), $Pd(OAc)_2$ (0.05 equiv., 0.0075 mmol) stirred for 5 h at 70 °C. The crude product was purified by automated column chromatography (pentane/EtOAc, 8:2) and the desired product was obtained as a white solid (38.5 mg, 74%); **Rf:** 0.39 (hexane/EtOAc, 80:20); **^{1}H NMR (600 MHz, $CDCl_3$) δ=** 7.88 (d, *J* = 8.1 Hz, 1H), 7.43 (d, *J* = 8.4 Hz, 2H), 7.30 (td, *J* = 7.7, 1.7 Hz, 1H), 7.19 (dd, *J* = 7.6, 1.7 Hz, 1H), 7.14 (td, *J* = 7.4, 1.3 Hz, 1H), 7.00 (d, *J* = 8.2 Hz, 2H), 6.99 (s, 1H), 3.94 (s, 2H), 1.11 (s, 9H); **^{13}C{^{1}H} NMR (151 MHz, $CDCl_3$) δ=** 176.7, 138.0, 136.1, 132.1, 131.1, 130.6, 130.1, 128.0, 125.3, 124.1, 120.7, 39.6, 37.9, 27.5; **IR (neat, cm^{-1}) =** 3315 (br), 2961 (br), 2920 (br), 1647 (s), 1583 (w), 1507 (s), 1486 (s), 1451 (m), 1363 (w), 1173 (w), 1071 (w), 1011 (m), 797 (w), 749 (s), 624 (w); **HRMS (ToF-SIMS) (m/z):** calculated for $[M+H]^+$ $C_{18}H_{21}BrNO$ 346.0801; found 346.0807.

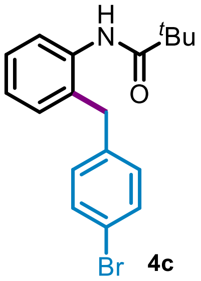

4c

N-(2-(4-(Trifluoromethoxy)benzyl)phenyl)pivalamide (4d):

Following the general procedure C using *N*-phenylpivalamide (26.6 mg, 0.15 mmol, 1 equiv.) BBr_3 (0.18 mmol, 1.2 equiv., 1M in CH_2Cl_2) at rt for 2 h and 4- trifluoromethoxy benzyl bromide (1.5 equiv., 0.22 mmol), K_2CO_3 (3 equiv., 0.45 mmol), $Pd(OAc)_2$ (0.05 equiv., 0.0075 mmol) stirred for 5 h at 70 °C. The crude product was purified by automated column chromatography (pentane/EtOAc, 8:2) and the desired product was

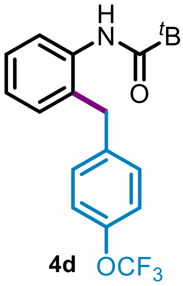

4d

obtained as a white solid (46 mg, 87%); **Rf:** 0.37 (hexane/EtOAc, 80:20); **Mp:** 107-109 ºC; **^{1}H NMR (600 MHz, $CDCl_3$) δ**= 7.91 (d, *J* = 8.1 Hz, 1H), 7.31 (t, *J* = 7.8 Hz, 1H), 7.21 (dd, *J* = 7.6, 1.7 Hz, 1H), 7.18 – 7.14 (m, 5H), 6.97 (bs, 1H), 4.01 (s, 2H), 1.08 (s, 9H); **^{13}C{^{1}H} NMR (151 MHz, $CDCl_3$) δ =** 176.7, 148.2, 137.8, 136.2, 131.2, 130.4, 129.7, 128.1, 125.3, 124.0, 121.7, 120.6 (q, *J* = 257.1 Hz), 39.6, 37.9, 27.4; **^{19}F NMR (564 MHz, $CDCl_3$) δ** = -58.06; **IR (neat, cm^{-1}) =** 3315 (br), 2967 (br), 2922 (br), 1647 (s), 1508 (s), 1483 (w), 1452 (w), 1437 (w), 1258 (s), 1223 (s), 1169 (s), 1159 (s), 751 (m), 661 (w), 622 (w); **HRMS (ESI) (m/z):** calculated for $[M+H]^+$ $C_{19}H_{21}F_3NO_2$ 352.1518; found 352.1524.

***N*-(2-(4-(Trifluoromethyl)benzyl)phenyl)pivalamide (4e):**

Following the general procedure C using *N*-phenylpivalamide (26.6 mg, 0.15 mmol, 1 equiv.) BBr_3 (0.18 mmol, 1.2 equiv., 1M in CH_2Cl_2) at rt for 2 h and 4- trifluoromethyl benzyl bromide (1.5 equiv., 0.22 mmol), K_2CO_3 (3 equiv., 0.45 mmol), $Pd(OAc)_2$ (0.05 equiv., 0.0075 mmol) stirred for 5 h at 70 °C. The crude product was purified by automated column chromatography (pentane/EtOAc, 8:2) and the desired product was obtained as a white solid (37.3 mg, 74%); **Rf:** 0.37 (hexane/EtOAc, 80:20); **Mp:** 82-84 ºC; **^{1}H NMR (600 MHz, $CDCl_3$) δ=** 7.85 (d, *J* = 9.0 Hz, 1H), 7.57 (d, *J* = 7.9 Hz, 2H), 7.32 (t, *J* = 7.5 Hz, 1H), 7.25 (d, *J* = 8.3 Hz, 2H), 7.21 (d, *J* = 7.6 Hz, 1H), 7.19 – 7.12 (m, 1H), 6.96 (bs, 1H), 4.06 (s, 2H), 1.10 (s, 9H); **^{13}C{^{1}H} NMR (151 MHz, $CDCl_3$) δ=** 176.7, 143.4, 136.0, 131.1, 130.7, 129.3 (q, *J* = 32.3 Hz), 128.8, 128.1, 125.9 (d, *J* = 4.3 Hz), 125.6, 124.5, 123.8 (q, *J* = 271.7 Hz), 39.6, 38.2, 27.5; **IR (neat, cm^{-1}) =** 3318 (br), 2955 (br), 2922 (s), 2853 (m), 1652 (s), 1510 (s), 1449 (s), 1325 (s), 1298 (w), 1162 (s), 1122 (s), 1067 (s), 1018 (w), 748 (s), 726 (m), 697 (w); **^{19}F NMR (564 MHz, $CDCl_3$) δ**= -62.51; **IR (neat, cm^{-1}) =** 3319 (br), 2956 (br), 2922 (s), 2853 (br), 1653 (s), 1587 (w), 1511 (s), 1450 (s), 1325 (s), 1163 (s), 1123 (s), 1067 (s), 1019 (w), 748 (s), 727 (m), 697 (w); **HRMS (ToF-SIMS) (m/z):** calculated for $[M+H]^+$ $C_{19}H_{21}F_3NO$ 336.1569; found 336.1570.

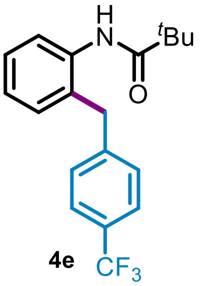

4e

***N*-(2-(4-cyanobenzyl)phenyl)pivalamide (4f):**

Following the general procedure C using *N*-phenylpivalamide (26.6 mg, 0.15 mmol, 1 equiv.) BBr_3 (0.18 mmol, 1.2 equiv., 1M in CH_2Cl_2) at rt for 2 h and 4-cyanobenzyl bromide (1.5 equiv., 0.22 mmol), K_2CO_3 (3 equiv., 0.45 mmol), $Pd(OAc)_2$ (0.05 equiv., 0.0075 mmol) stirred for 5 h at 70 °C. The crude product was purified by automated column chromatography (pentane/EtOAc, 8:2) and the desired product was obtained as a white solid (39 mg, 89%); **Rf:** 0.37 (hexane/EtOAc, 80:20); **M.P**: 176-178 ºC; **^{1}H NMR (600 MHz, $CDCl_3$) δ =** 7.76 (d, *J* = 8.0 Hz, 1H), 7.58 (d, *J* = 8.3 Hz, 2H), 7.31 (dd, *J* = 8.2, 5.2 Hz, 1H), 7.23 (d, *J* = 8.1 Hz, 2H), 7.18 – 7.15 (m, 2H), 6.95 (bs, 1H), 4.04 (s, 2H), 1.12 (s, 9H); **^{13}C{^{1}H} NMR (151 MHz, $CDCl_3$) δ=** 176.7, 145.0, 135.9, 132.7, 131.1, 130.8, 129.3, 128.2, 125.9, 125.0, 118.8, 110.7, 39.5, 38.4, 27.6; **IR (neat, cm^{-1}) =** 3313 (br), 2963 (br),

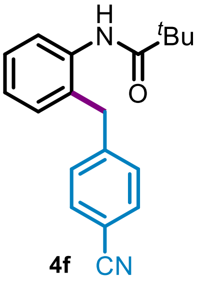

4f

2228 (m), 1646 (s), 1609 (w), 1583 (w), 1502 (s), 1449 (s), 1295 (w), 1250 (w), 1171 (m), 921 (m), 829 (w), 814 (m), 759 (s), 746 (m), 620 (m); **HRMS (ESI) (m/z):** calculated for $[M+H]^+$ $C_{19}H_{21}N_2O$ 293.1648; found 293.1652.

***N*-(2-(4-nitrobenzyl)phenyl)pivalamide (4g):**

Following the general procedure C using *N*-phenylpivalamide (26.6 mg, 0.15 mmol, 1 equiv.) BBr_3 (0.18 mmol, 1.2 equiv., 1M in CH_2Cl_2) at rt for 2 h and 1-(bromomethyl)-4-nitrobenzene (1.5 equiv., 0.22 mmol), K_2CO_3 (3 equiv., 0.45 mmol), $Pd(OAc)_2$ (0.05 equiv., 0.0075 mmol) stirred for 5 h at 70 °C. The crude product was purified by automated column chromatography (pentane/EtOAc, 85:15) and the desired product was obtained as a white solid (41 mg, 87%); **Rf:** 0.33 (hexane/EtOAc, 80:20); **M.P**: 196-198 ºC; **^{1}H NMR (600 MHz, $CDCl_3$) δ** =8.15 (d, *J* = 8.7 Hz, 2H), 7.73 (d, *J* = 8.7 Hz, 1H), 7.32 (ddd, *J* = 8.9, 6.3, 2.7 Hz, 1H), 7.29 (d, *J* = 9.1 Hz, 2H), 7.21 – 7.16 (m, 2H), 6.96 (bs, 1H), 4.08 (s, 2H), 1.14 (s, 9H); **$^{13}C\{^1H\}$ NMR (151 MHz, $CDCl_3$) δ=** 176.8, 147.2, 146.9, 135.9, 131.1, 131.1, 129.3, 128.3, 126.1, 125.2, 124.1, 39.6, 38.1, 27.6; **IR (neat, cm^{-1})** = 3324 (br), 2963 (br), 1648 (s), 1596 (w), 1506 (s), 1449 (m), 1435 (m), 1347 (s), 1172 (w), 1112 (w), 924 (m), 858 (m), 840 (m), 754 (s), 733 (m), 617 (m); **HRMS (ESI) (m/z):** calculated for $[M+H]^+$ $C_{18}H_{21}N_2O_3$ 313.1546; found 313.1551.

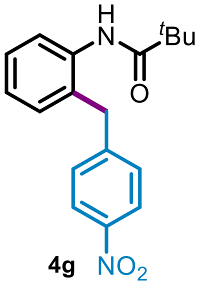

***N*-(2-(4-(Methylsulfonyl)benzyl)phenyl)pivalamide (4h):**

Following the general procedure C using *N*-phenylpivalamide (26.6 mg, 0.15 mmol, 1 equiv.) BBr_3 (0.18 mmol, 1.2 equiv., 1M in CH_2Cl_2) at rt for 2 h and 4- methylsulfonyl benzyl bromide (1.5 equiv., 0.22 mmol), K_2CO_3 (3 equiv., 0.45 mmol), $Pd(OAc)_2$ (0.05 equiv., 0.0075 mmol) stirred for 5 h at 70 °C. The crude product was purified by automated column chromatography (pentane/EtOAc, 8:2) and the desired product was obtained as a white solid (46 mg, 89%); **Rf:** 0.31 (hexane/EtOAc, 80:20); **Mp:** 153 – 155 ºC; **^{1}H NMR (600 MHz, $CDCl_3$) δ** = 7.84 (d, *J* = 8.4 Hz, 2H), 7.71 (d, *J* = 8.0 Hz, 1H), 7.31 (d, *J* = 8.0 Hz, 2H), 7.29 (dd, *J* = 5.1, 3.1 Hz, 1H), 7.17 – 7.16 (m, 2H), 7.01 (bs, 1H), 4.06 (s, 2H), 3.00 (s, 3H), 1.11 (s, 9H); **$^{13}C\{^1H\}$ NMR (151 MHz, $CDCl_3$) δ=** 176.8, 146.1, 139.0, 135.8, 131.3, 131.0, 129.5, 128.2, 127.9, 125.9, 125.2, 44.6, 39.5, 38.1, 27.5; **IR (neat, cm^{-1})** = 3321 (br), 2958 (br), 2924 (br), 2867 (br), 1658 (m), 1596 (w), 1508 (m), 1490 (m), 1449 (m), 1408 (w), 1301 (s), 1145 (s), 1089 (m), 956 (w), 751 (s), 653 (w), 596 (w), 529 (m); **HRMS (ESI) (m/z):** calculated for $[M+H]^+$ $C_{19}H_{24}NO_3S$ 346.1471; found 346.1476.

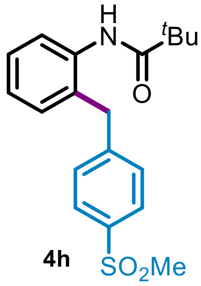

***N*-(2-(3,5-Dimethylbenzyl)phenyl)pivalamide (4i):**

Following the general procedure C using *N*-phenylpivalamide (26.6 mg, 0.15 mmol, 1 equiv.) BBr_3 (0.18 mmol, 1.2 equiv., 1M in CH_2Cl_2) at rt for 2 h and 3,5-dimethylbenzyl bromide (1.5 equiv., 0.22 mmol), K_2CO_3 (3 equiv., 0.45 mmol), $Pd(OAc)_2$ (0.05 equiv., 0.0075 mmol) stirred for 5 h at 70 °C. The crude product was purified by automated column chromatography (pentane/EtOAc, 8:2) and the desired product was obtained as a white solid (30.9 mg, 70%); **Rf:** 0.61 (hexane/EtOAc, 80:20); **Mp:** 136-138 ºC; **^{1}H NMR (600 MHz, $CDCl_3$) δ** = 8.01 (d, *J* = 8.2 Hz, 1H), 7.30 (td, *J* = 7.8, 1.6 Hz, 1H), 7.25 (d, *J* = 7.5 Hz, 1H), 7.13 (td, *J* = 7.5, 1.3 Hz, 1H), 7.09 (bs, 1H), 6.88 (s, 1H), 6.76 (s, 2H), 3.93 (s, 2H), 2.26 (s, 6H), 1.08 (s, 9H); **^{13}C{^{1}H} NMR (151 MHz, $CDCl_3$) δ=** 176.7, 138.7, 138.7, 136.5, 131.2, 130.5, 128.7, 127.7, 126.2, 124.7, 123.3, 39.6, 38.9, 27.4, 21.3; **IR (neat, cm^{-1}) =** 3256 (br), 2961 (br), 2907 (br), 2867 (br), 1648 (s), 1601 (m), 1518 (s), 1486 (s), 1398 (w), 1365 (w), 1303 (m), 1268 (w), 1239 (w), 1227 (w), 1187 (m), 1173 (w), 931 (w), 847 (m), 750 (s), 702 (w); **HRMS (ESI) (m/z):** calculated for $[M+H]^+$ $C_{20}H_{26}NO$ 296.2008; found 296.2014.

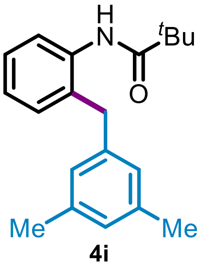

4i

***N*-(2-(3,5-Di-tert-butylbenzyl)phenyl)pivalamide (4j):**

Following the general procedure C using *N*-phenylpivalamide (26.6 mg, 0.15 mmol, 1 equiv.) BBr_3 (0.18 mmol, 1.2 equiv., 1M in CH_2Cl_2) at rt for 2 h and 3,5-di-tert-butylbenzyl bromide (1.5 equiv., 0.22 mmol), K_2CO_3 (3 equiv., 0.45 mmol), $Pd(OAc)_2$ (0.05 equiv., 0.0075 mmol) stirred for 5 h at 70 °C. The crude product was purified by automated column chromatography (pentane/EtOAc, 8:2) and the desired product was obtained as a white solid (50 mg, 88%); **Rf:** 0.43 (hexane/EtOAc, 90:10); **Mp:** 104-106 ºC; **^{1}H NMR (600 MHz, $CDCl_3$) δ =** 8.06 (d, *J* = 8.1 Hz, 1H), 7.34 – 7.29 (m, 2H), 7.25 (d, *J* = 7.7 Hz, 1H), 7.13 (td, *J* = 7.4, 1.3 Hz, 1H), 7.05 (bs, 1H), 6.98 (s, 2H), 4.03 (s, 2H), 1.27 (s, 18H), 0.99 (s, 9H); **^{13}C{^{1}H} NMR (151 MHz, $CDCl_3$) δ=** 176.5, 151.8, 137.6, 136.5, 131.3, 130.0, 127.7, 124.6, 122.9, 122.4, 121.1, 39.6, 39.4, 35.0, 31.5, 27.4; **IR (neat, cm^{-1}) =** 3415 (br), 3336 (br), 2954 (m), 2904 (br), 2867 (br), 1675 (m), 1596 (m), 1587 (m), 1517 (s), 1476 (s), 1448 (s), 1393 (w), 1362 (m), 1293 (m), 1247 (s), 1160 (m), 1045 (w), 935 (w), 921 (w), 858 (w), 759 (m), 748 (s), 713 (s), 668 (w), 565 (w); **HRMS (ESI) (m/z):** calculated for $[M+H]^+$ $C_{26}H_{38}NO$ 380.2947; found 380.2956.

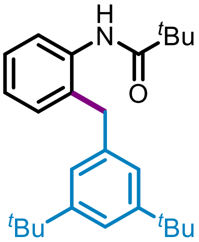

4j

***N*-(2-(3,5-dimethoxybenzyl)phenyl)pivalamide (4k):**

Following the general procedure C using *N*-phenylpivalamide (26.6 mg, 0.15 mmol, 1 equiv.) BBr_3 (0.18 mmol, 1.2 equiv., 1M in CH_2Cl_2) at rt for 2 h and 3,5-dimethoxybenzyl bromide (1.5 equiv., 0.22 mmol), K_2CO_3 (3 equiv., 0.45 mmol), $Pd(OAc)_2$ (0.05 equiv., 0.0075 mmol) stirred for 5 h at 70 °C. The crude product was purified by automated column chromatography (pentane/EtOAc, 8:2) and the desired product

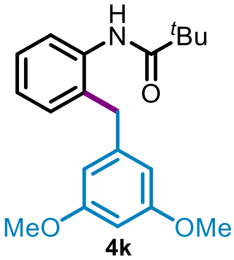

4k

was obtained as a white solid (44 mg, 90%); Scale up reaction condition (1mmol): Step i) *N*-phenylpivalamide (177.24 mg, 1 mmol, 1 equiv.) BBr_3 (1.2 mL, 1.2 mmol, 1.2 equiv., 1M in CH_2Cl_2) in 3.5 mL anhydrous CH_2Cl_2, at rt for 2 h. Step ii) 3,5-dimethoxybenzyl bromide (346.62 mg, 1.5 mmol, 1.5 equiv.,), K_2CO_3 (414.61 mg, 3 mmol, 3 equiv.), $Pd(OAc)_2$ (11.23 mg, 0.05 mmol, 0.05 equiv.) in 5 mL MeOH and 5 mL distilled water, stirred for 5 h at 70 °C (287 mg, 88%); **Rf:** 0.37 (hexane/EtOAc, 80:20); **Mp:** 71-73 °C; **^{1}H NMR (600 MHz, $CDCl_3$) δ =** 7.93 (d, *J* = 8.1 Hz, 1H), 7.30 – 7.27 (m, 1H), 7.22 (dd, *J* = 7.5, 1.6 Hz, 1H), 7.12 (t, *J* = 7.5 Hz, 1H), 7.10 (bs, 1H), 6.34 (t, *J* = 2.3 Hz, 1H), 6.29 (d, *J* = 2.3 Hz, 2H), 3.93 (s, 2H), 3.73 (s, 6H), 1.11 (s, 9H); **^{13}C{^{1}H} NMR (151 MHz, $CDCl_3$) δ=** 176.7, 161.4, 141.3, 136.4, 131.1, 130.4, 127.8, 124.9, 123.7, 106.5, 98.7, 55.4, 39.6, 39.0, 27.5; **IR (neat, cm^{-1}) =** 3326 (br), 2958 (br), 2933 (br), 1669 (m), 1593 (s), 1512 (m), 1449 (s), 1429 (m), 1259 (w), 1204 (s), 1154 (s), 1064 (m), 925 (w), 833 (w), 751 (m), 691 (w), 662 (w); **HRMS (ESI) (m/z):** calculated for $[M+H]^+$ $C_{20}H_{26}NO_3$ 328.1907; found 328.1913.

***N*-(2-([1,1'-biphenyl]-4-ylmethyl)phenyl)pivalamide (4l):**

Following the general procedure C using *N*-phenylpivalamide (26.6 mg, 0.15 mmol, 1 equiv.) BBr_3 (0.18 mmol, 1.2 equiv., 1M in CH_2Cl_2) at rt for 2 h and 4-phenylbenzyl bromide (1.5 equiv., 0.22 mmol), K_2CO_3 (3 equiv., 0.45 mmol), $Pd(OAc)_2$ (0.05 equiv., 0.0075 mmol) stirred for 5 h at 70 °C. The crude product was purified by automated column chromatography (pentane/EtOAc, 8:2) and the desired product was obtained as a white solid (46.8 mg, 91%); **Rf:** 0.47 (hexane/EtOAc, 80:20); **Mp:** 94-96 °C; **^{1}H NMR (600 MHz, $CDCl_3$) δ=** 8.00 (d, *J* = 8.1 Hz, 1H), 7.57 (t, *J* = 7.7 Hz, 4H), 7.45 (t, *J* = 7.4 Hz, 2H), 7.38 – 7.32 (m, 2H), 7.29 (d, *J* = 7.5 Hz, 1H), 7.23 (d, *J* = 7.9 Hz, 2H), 7.17 (td, *J* = 7.5, 1.3 Hz, 1H), 7.10 (bs, 1H), 4.07 (s, 2H), 1.10 (s, 9H); **^{13}C{^{1}H} NMR (151 MHz, $CDCl_3$) δ=** 176.7, 140.7, 140.0, 137.9, 136.3, 131.2, 130.5, 128.9, 128.8, 127.9, 127.8, 127.5, 127.1, 125.0, 123.7, 39.6, 38.4, 27.5; **IR (neat, cm^{-1}) =** 3318 (br), 2959 (br), 2905 (br), 2868 (br), 1653 (m), 1586 (w), 1507 (s), 1486 (s), 1447 (s), 1365 (w), 1301 (w), 1292 (w), 1245 (w), 1225 (w), 1158 (m), 1007 (w), 913 (w), 833 (w), 759 (s), 696 (s), 612 (w), 563 (w); **HRMS (ESI) (m/z):** calculated for $[M+H]^+$ $C_{24}H_{26}NO$ 344.2008; found 344.2014.

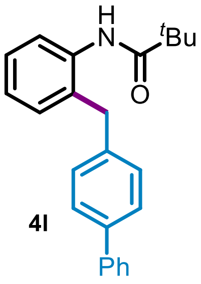

4l

***N*-(2-(Naphthalen-2-ylmethyl)phenyl)pivalamide (4m):**

Following the general procedure C using *N*-phenylpivalamide (26.6 mg, 0.15 mmol, 1 equiv.) BBr_3 (0.18 mmol, 1.2 equiv., 1M in CH_2Cl_2) at rt for 2 h and 2-(bromomethyl)naphthalene (1.5 equiv., 0.22 mmol), K_2CO_3 (3 equiv., 0.45 mmol), $Pd(OAc)_2$ (0.05 equiv., 0.0075 mmol) stirred for 5 h at 70 °C. The crude product was purified by automated column chromatography (pentane/EtOAc, 8:2) and the desired product was obtained as a white solid (37 mg, 77%); **Rf:** 0.55 (hexane/EtOAc, 80:20); **Mp:** 96-98 °C; **^{1}H NMR (600 MHz, $CDCl_3$) δ**

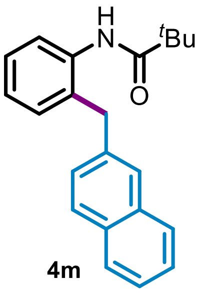

4m

= 7.98 (d, *J* = 8.1 Hz, 1H), 7.81 (dd, *J* = 8.9, 6.4 Hz, 2H), 7.75 – 7.72 (m, 1H), 7.55 (d, *J* = 2.0 Hz, 1H), 7.49 – 7.44 (m, 2H), 7.34 (td, *J* = 7.8, 1.6 Hz, 1H), 7.30 (s, 2H), 7.18 (td, *J* = 7.5, 1.3 Hz, 1H), 7.14 (bs, 1H), 4.17 (s, 2H), 1.03 (s, 9H); **^{13}C{^{1}H} NMR (151 MHz, $CDCl_3$) δ =** 176.7, 136.4, 136.4, 133.7, 132.5, 131.3, 130.6, 128.9, 127.9, 127.8, 127.6, 126.8, 126.6, 126.5, 125.9, 125.1, 123.8, 39.6, 38.9, 27.5; **IR (neat, cm^{-1}) =** 3409 (br), 2963 (br), 1669 (s), 1586 (m), 1515(s), 1441 (s), 1369 (w), 1301 (m), 1282 (w), 1247 (s), 1159 (s), 1046 (w), 953 (w), 917 (w), 853 (w),816 (s), 761 (s), 751 (s), 736 (s); **HRMS (ToF-SIMS) (m/z):** calculated for [M-H] $C_{22}H_{22}NO$ 316.1706; found 316.1702.

***N*-(2-(4-(1,2,3-thiadiazol-4-yl)benzyl)phenyl)pivalamide (4n):**

Following the general procedure C using *N*-phenylpivalamide (26.6 mg, 0.15 mmol, 1 equiv.) BBr_3 (0.18 mmol, 1.2 equiv., 1M in CH_2Cl_2) at rt for 2 h and 4-(4-(bromomethyl)phenyl)-1,2,3-thiadiazole (1.5 equiv., 0.22 mmol), K_2CO_3 (3 equiv., 0.45 mmol), $Pd(OAc)_2$ (0.05 equiv., 0.0075 mmol) stirred for 5 h at 70 °C. The crude product was purified by automated column chromatography (pentane/EtOAc, 8:2) and the desired product was obtained as a white solid (40 mg, 76%); **Rf:** 0.31 (hexane/EtOAc, 80:20); **Mp:** 163-165 ºC; **^{1}H NMR (600 MHz, $CDCl_3$) δ=** 8.64 (s, 1H), 8.00 (d, *J* = 8.3 Hz, 2H), 7.93 (d, *J* = 8.1 Hz, 1H), 7.33 (t, *J* = 7.7 Hz, 1H), 7.30 – 7.27 (m, 3H), 7.17 (td, *J* = 7.5, 1.3 Hz, 1H), 7.09 (bs, 1H), 4.08 (s, 2H), 1.12 (s, 9H); **^{13}C{^{1}H} NMR (151 MHz, $CDCl_3$) δ=** 176.7, 162.5, 140.4, 136.2, 131.2, 130.6, 130.1, 129.6, 129.2, 128.0, 127.9, 125.3, 124.1, 39.6, 38.3, 27.5; **IR (neat, cm^{-1}) =** 3322 (br), 2957 (m), 2921 (s), 2851 (m), 1662 (s), 1586 (w), 1509 (s), 1480 (s), 1464 (s), 1450 (s), 1298 (w), 1247 (w), 1225 (w), 1185 (m), 1164 (w), 934 (m), 892 (w), 750 (s); **HRMS (ToF-SIMS) (m/z):** calculated for $[M+H]^+$ $C_{20}H_{22}N_3OS$ 352.1478; found 352.1477.

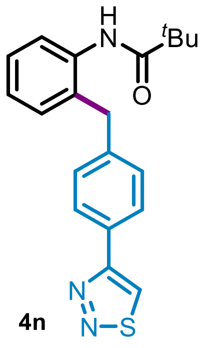

4n

2.7 General Procedure D for reaction optimization: Scope of Digonal Di-Benzylation (7-12)

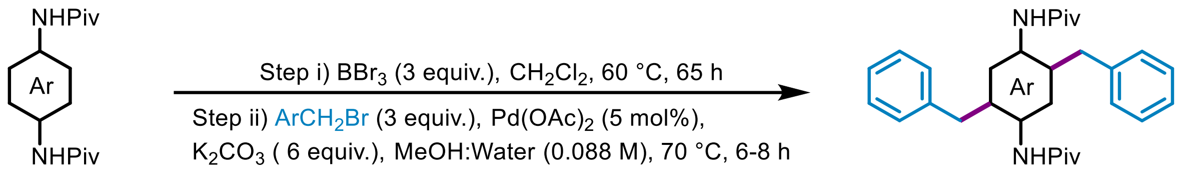

5-6
(0.15 mmol)

7-12

Step i) To a dry 5 mL, screw top V-Vial®, equipped with a rubber septum, stir bar, the amide derivative (5-6) (0.15 mmol, 1 equiv.) in anhydrous CH_2Cl_2 (0.5 mL) under a nitrogen atmosphere was added dropwise BBr_3 (0.45 mL, 0.45 mmol, 3 equiv., 1M solution in CH_2Cl_2). After the complete addition of BBr_3, the reaction mixture was stirred at 60 ºC for 65 h after which the solvent was removed under reduced pressure.

Step ii) To the crude residue from step i) were added K_2CO_3 (0.9 mmol, 6 equiv.), 1 mL degassed methanol along with substituted benzyl bromide (0.3 mmol, 3 equiv.) and 0.7 mL distilled water. Then palladium acetate (5 mol%, 0.05 equiv.) was added under nitrogen atmosphere and the reaction mixture was heated at 70 ºC for 8 h. The reaction was allowed to reach room temperature and diluted with 2 mL ethyl acetate and filtered through a pad of celite and sodium sulfate. The celite pad was washed with additional 15 mL of ethyl acetate and the filtrate was evaporated *in vacuo* to afford the crude product, which was purified using automated column chromatography (pentane/EtOAc).

2.8 Spectral data

N,N'-(2,5-dibenzyl-1,4-phenylene)bis(2,2-dimethylpropanamide) (7):

Following the general procedure D using *N,N'*-(1,4-phenylene)bis(2,2-dimethylpropanamide) (41.5 mg, 0.15 mmol, 1 equiv.) BBr_3 (0.45 mmol, 3.0 equiv., 1M in CH_2Cl_2) at 60°C for 65 h and benzyl bromide

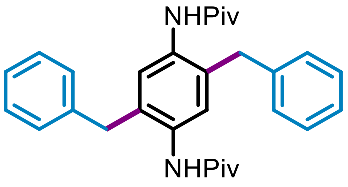

7

(3 equiv., 0.45 mmol), K_2CO_3 (124.37 mg, 6 equiv., 0.6 mmol), $Pd(OAc)_2$ (1.68 mg, 0.05 equiv., 0.0075 mmol) stirred for 6 h at 70 °C. The crude product was purified by automated column chromatography (pentane/EtOAc, 8:2) and the desired product was obtained as a white solid (42 mg, 61%); **Rf:** 0.57 (hexane/EtOAc, 60:40); **Mp:** 264-266 ºC; **^{1}H NMR (600 MHz, CDCl$_3$) δ** = 8.02 (s, 2H), 7.31 (t, *J* = 7.5 Hz, 4H), 7.23 (t, *J* = 7.6 Hz, 2H), 7.19 (d, *J* = 8.4 Hz, 4H), 7.01 (bs, 2H), 4.02 (s, 4H), 1.05 (s, 18H); **^{13}C{^{1}H} NMR (151 MHz, CDCl$_3$) δ=** 176.7, 138.7, 132.9, 129.5, 129.1, 128.4, 127.1, 126.0, 39.6, 38.8, 27.5; **IR (neat, cm^{-1})** = 3429 (br), 2958 (br), 1664 (s), 1531 (s), 1490 (w), 1438 (m), 1412 (s), 1312 (m), 1212 (m), 1172 (m), 1156 (m), 1029 (w), 949 (w), 929 (w), 868 (w), 724 (s), 694 (s), 566 (s); **HRMS (ESI) (m/z):** calculated for $[M+H]^+$ $C_{30}H_{37}N_2O_2$ 457.2849; found 457.2858.

_N,N'_-(2,5-bis(3,5-di-tert-butylbenzyl)-1,4-phenylene)bis(2,2-dimethylpropanamide) (8):

Following the general procedure D using _N,N'_-(1,4-phenylene)bis(2,2-dimethylpropanamide) (41.5 mg, 0.15 mmol, 1 equiv.) BBr_3 (0.45 mmol, 3.0 equiv., 1M in CH_2Cl_2) at 60°C for 65 h and 1-(bromomethyl)-3,5-di-tert-butylbenzene (3 equiv., 0.45 mmol), K_2CO_3 (124.37 mg, 6 equiv., 0.6 mmol), $Pd(OAc)_2$ (1.68 mg, 0.05 equiv., 0.0075 mmol) stirred for 8 h at 70 °C. The crude product was purified by automated column chromatography (pentane/EtOAc, 85:15) and the desired product was obtained as a white solid (57 mg, 56%); **Rf:** 0.66 (hexane/EtOAc, 80:20); **Mp:** 194-196 ºC; **^{1}H NMR (600 MHz, CDCl$_3$) δ** = 8.14 (s, 2H), 7.30 (t, J = 1.8 Hz, 2H), 7.00 (d, J = 1.7 Hz, 6H), 4.05 (s, 4H), 1.27 (s, 36H), 0.94 (s, 18H); **^{13}C{^{1}H} NMR (151 MHz, CDCl$_3$) δ=** 176.5, 151.7, 137.7, 132.9, 128.8, 125.3, 122.3, 121.1, 39.6, 34.9, 31.6, 27.4; **IR (neat, cm^{-1}) =** 3389 (br), 2953 (br), 1664 (s), 1598 (w), 1526 (s), 1478 (m), 1409 (m), 1362 (m), 1248 (w), 1207 (m), 1159 (m), 868 (m), 713 (m), 672 (w), 577 (m); **HRMS (ESI) (m/z):** calculated for $[M+H]^+$ $C_{46}H_{69}N_2O_2$ 681.5353; found 681.5364.

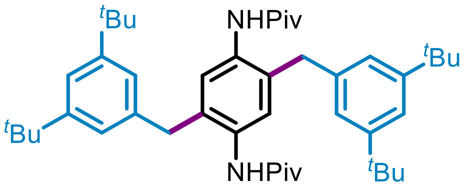

8

_N,N'_-(2,5-bis(3,5-dimethoxybenzyl)-1,4-phenylene)bis(2,2-dimethylpropanamide) (9):

Following the general procedure D using _N,N'_-(1,4-phenylene)bis(2,2-dimethylpropanamide) (41.5 mg, 0.15 mmol, 1 equiv.) BBr_3 (0.45 mmol, 3.0 equiv., 1M in CH_2Cl_2) at 60°C for 65 h and 1-(bromomethyl)-3,5-dimethoxybenzene (3 equiv., 0.45 mmol), K_2CO_3 (124.37 mg, 6 equiv., 0.6 mmol), $Pd(OAc)_2$ (1.68 mg, 0.05 equiv., 0.0075 mmol) stirred for 6 h at 70 °C. The crude product was purified by automated column chromatography (pentane/EtOAc, 70:30) and the desired product was obtained as a white solid (63 mg, 73%); **Rf:** 0.25 (hexane/EtOAc, 70:30); **Mp:** 200-202 ºC; **^{1}H NMR (600 MHz, CDCl$_3$) δ** = 8.00 (s, 2H), 7.08 (bs, 2H), 6.32 (s, 6H), 3.94 (s, 4H), 3.73 (s, 12H), 1.09 (s, 18H); **^{13}C{^{1}H} NMR (151 MHz, CDCl$_3$) δ=** 176.7, 161.5, 141.2, 132.9, 129.2, 125.9, 106.5, 98.8, 55.5, 39.7, 39.1, 27.5; **IR (neat, cm^{-1}) =** 3432 (br), 2962 (br), 1662 (s), 1593 (s), 1530 (s), 1462 (m), 1430 (s), 1409 (s), 1309 (w), 1201 (s), 1151 (s), 1061 (s), 843 (m), 831 (m), 689 (m), 543 (m); **HRMS (ESI) (m/z):** calculated for $[M+H]^+$ $C_{34}H_{45}N_2O_6$ 577.3272; found 577.3279.

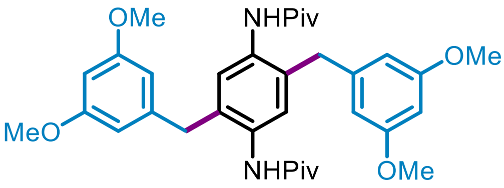

9

_N,N'_-(2,6-dibenzylnaphthalene-1,5-diyl)bis(2,2-dimethylpropanamide) (10):

Following the general procedure D using _N,N'_-(naphthalene-1,5-diyl)bis(2,2-dimethylpropanamide) (48.96 mg, 0.15 mmol, 1 equiv.) BBr_3 (0.45 mmol, 3.0 equiv., 1M in CH_2Cl_2) at 60°C for 65 h and benzyl bromide (3 equiv., 0.45 mmol), K_2CO_3 (124.37 mg, 6 equiv., 0.6 mmol), $Pd(OAc)_2$ (1.68 mg, 0.05 equiv., 0.0075 mmol) stirred for 8 h at 70 °C. The crude product was purified by automated column

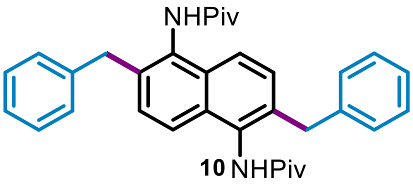

10

chromatography (pentane/EtOAc, 70:30) and the desired product was obtained as a white solid (46.36 mg, 61%); **Rf:** 0.61 (hexane/EtOAc, 60:40); **Mp:** 274-276 ºC; **^{1}H NMR (600 MHz, $CDCl_3$) δ** 7.69 (d, *J* = 8.7 Hz, 2H), 7.34 (d, *J* = 8.7 Hz, 2H), 7.29 – 7.26 (m, 4H), 7.20 (t, *J* = 7.4 Hz, 2H), 7.12 (d, *J* = 7.6 Hz, 6H), 4.09 (s, 4H), 1.33 (s, 18H); **^{13}C{^{1}H} NMR (151 MHz, $CDCl_3$) δ=** 177.6, 140.2, 135.5, 131.1, 131.0, 129.4, 128.8, 126.5, 122.6, 39.5, 38.3, 27.9; **IR (neat, cm^{-1}) =** 3432 (br), 2962 (br), 1662 (s), 1593 (s), 1530 (s), 1462 (m), 1430 (s), 1409 (s), 1309 (w), 1201 (s), 1151 (s), 1061 (s), 843 (m), 831 (m), 689 (m), 543 (m); **HRMS (ESI) (m/z):** calculated for $[M+H]^+$ $C_{34}H_{39}N_2O_2$ 507.3006; found 507.3019.

***N,N'*-(2,6-bis(3,5-dimethylbenzyl)naphthalene-1,5-diyl)bis(2,2-dimethylpropanamide) (11):**

Following the general procedure D using *N,N'*-(naphthalene-1,5-diyl)bis(2,2-dimethylpropanamide) (48.96 mg, 0.15 mmol, 1 equiv.) BBr_3 (0.45 mmol, 3.0 equiv., 1M in CH_2Cl_2) at 60°C for 65 h and 1-(bromomethyl)-3,5-dimethylbenzene (89.58 mg, 3 equiv., 0.45 mmol), K_2CO_3 (124.37 mg, 6 equiv., 0.6 mmol), $Pd(OAc)_2$ (1.68 mg, 0.05 equiv., 0.0075 mmol) stirred for 8 h at 70 °C. The crude product was purified by automated column chromatography (pentane/EtOAc, 80:20) and the desired product was obtained as a white solid (61.5 mg, 73%); **Rf:** 0.15 (hexane/EtOAc, 80:20); **Mp:** 258-260 ºC; **^{1}H NMR (600 MHz, $CDCl_3$) δ=** 7.69 (d, *J* = 8.7 Hz, 2H), 7.36 (d, *J* = 8.7 Hz, 2H), 7.07 (bs, 2H), 6.84 (s, 2H), 6.74 (s, 4H), 4.01 (s, 4H), 2.25 (s, 12H), 1.35 (s, 18H); **^{13}C{^{1}H} NMR (151 MHz, $CDCl_3$) δ=** 177.5, 140.0, 138.4, 135.5, 131.1, 130.9, 129.4, 128.1, 126.7, 122.6, 39.5, 38.4, 27.9, 21.4; **HRMS (ESI) (m/z):** calculated for $[M+H]^+$ $C_{38}H_{47}N_2O_2$ 563.3632; found 563.3643.

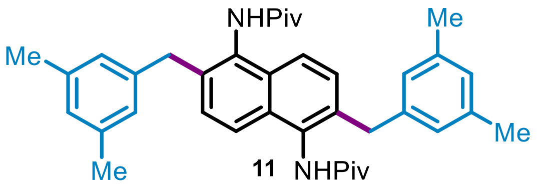

***N,N'*-(2,6-bis(3,5-dimethoxybenzyl)naphthalene-1,5-diyl)bis(2,2-dimethylpropanamide) (12):**

Following the general procedure D using *N,N'*-(naphthalene-1,5-diyl)bis(2,2-dimethylpropanamide) (41.5 mg, 0.15 mmol, 1 equiv.) BBr_3 (0.45 mmol, 3.0 equiv., 1M in CH_2Cl_2) at 60°C for 65 h and 1-(bromomethyl)-3,5-dimethoxybenzene (103.98 mg, 3 equiv., 0.45 mmol), K_2CO_3 (124.37 mg, 6 equiv., 0.6 mmol), $Pd(OAc)_2$ (1.68 mg, 0.05 equiv., 0.0075 mmol) stirred for 8 h at 70 °C. The crude product was purified by automated column chromatography (pentane/EtOAc, 75:25) and the desired product was obtained as a white solid (79 mg, 84%); **Rf:** 0.14 (hexane/EtOAc, 70:30); **Mp:** 226-228 ºC; **^{1}H NMR (600 MHz, $CDCl_3$) δ=** 7.68 (d, *J* = 8.6 Hz, 2H), 7.33 (d, *J* = 8.8 Hz, 2H), 7.15 (bs, 2H), 6.33 – 6.27 (m, 6H), 4.01 (s, 4H), 3.72 (s, 12H), 1.35 (s, 18H); **^{13}C{^{1}H} NMR (151 MHz, $CDCl_3$) δ=** 177.5, 161.2, 142.5, 135.1, 131.1, 131.0, 129.2, 122.7, 106.9, 98.5, 55.5, 39.6, 38.6, 27.9; **HRMS (ESI) (m/z):** calculated for $[M+H]^+$ $C_{38}H_{47}N_2O_6$ 627.3428; found 627.3441.

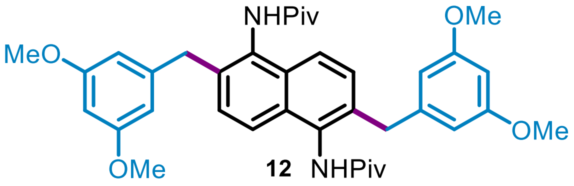

2.9 Confirmation of dibromoboracycle intermediate (2a)[(1, 2a)]

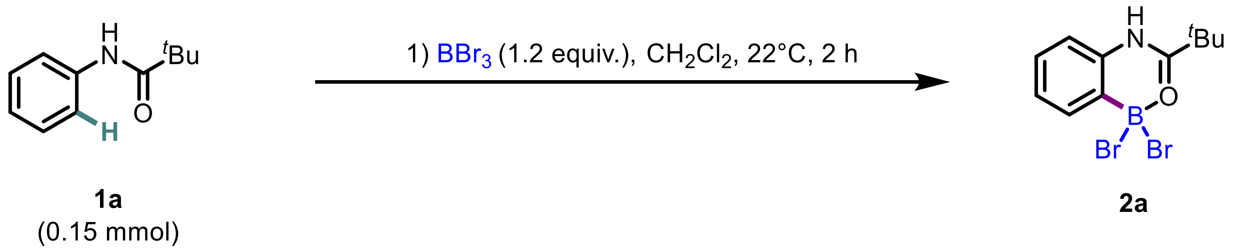

Step i) To a dry 5 mL, screw top V-Vial®, equipped with a rubber septum, stir bar, the *N*-phenylpivalamide (26.59 mg, 0.15 mmol, 1 equiv.) in anhydrous CH_2Cl_2 (0.5 mL) under a nitrogen atmosphere was added dropwise BBr_3 (180 µl, 0.18 mmol, 1.2 equiv., 1M solution in CH_2Cl_2). After the complete addition of BBr_3, the reaction mixture was stirred at 22 °C for 2 h after which the solvent was removed under reduced pressure. The crude solid was analyzed by NMR.

Spectral data: ^{1}H NMR (800 MHz, $CDCl_3$) δ= 8.89 (bs, 1H), 7.84 (d, *J* = 7.4 Hz, 1H), 7.45 (t, *J* = 7.5 Hz, 1H), 7.34 (t, *J* = 7.7 Hz, 1H), 7.01 (dt, *J* = 7.9, 3.6 Hz, 1H), 1.49 (s, 9H); **^{13}C{^{1}H} NMR (201 MHz, $CDCl_3$) δ=** 176.9, 134.2, 130.9, 129.9, 129.6, 116.1, 38.8, 26.5.

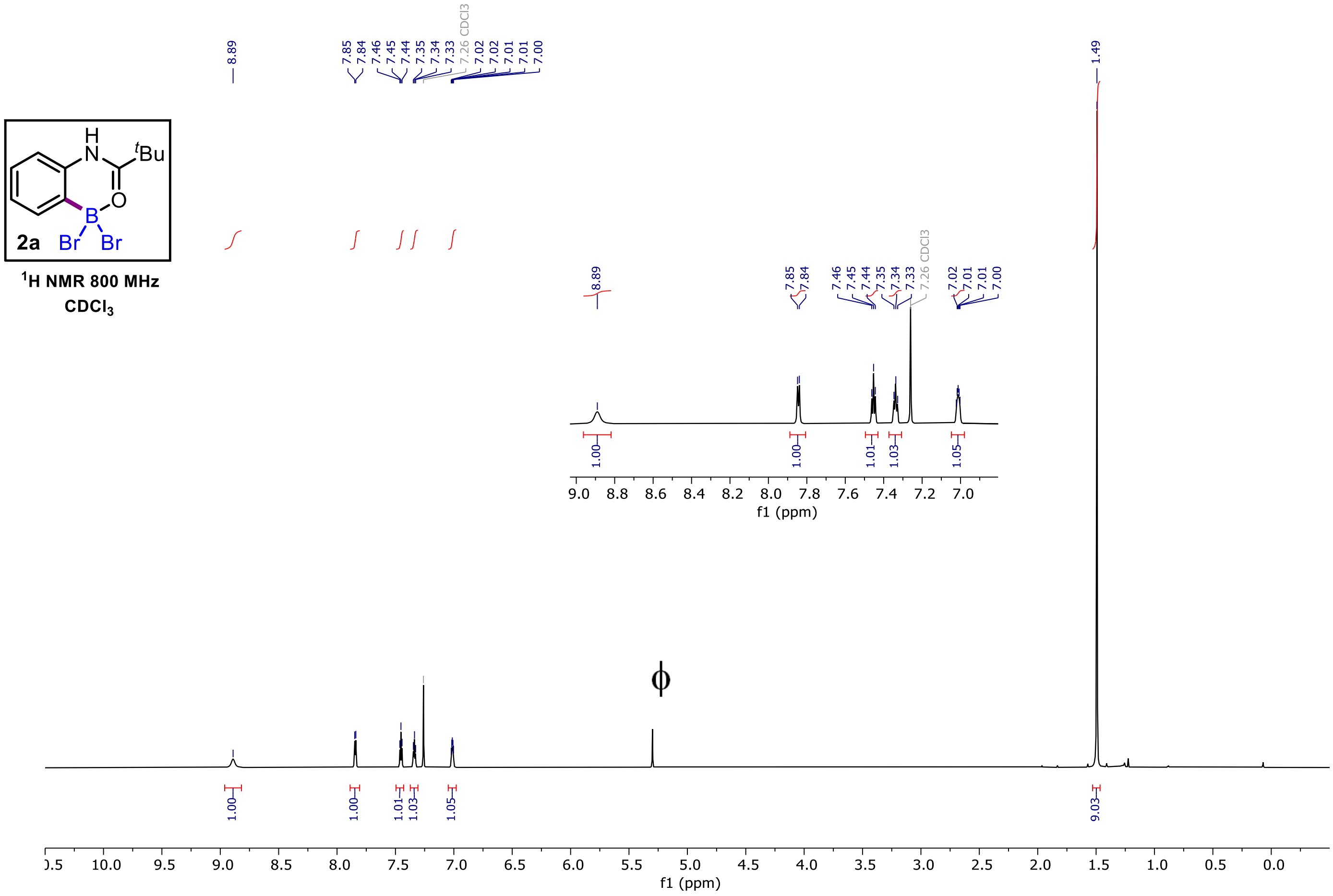

ϕ= Residual CH_2Cl_2

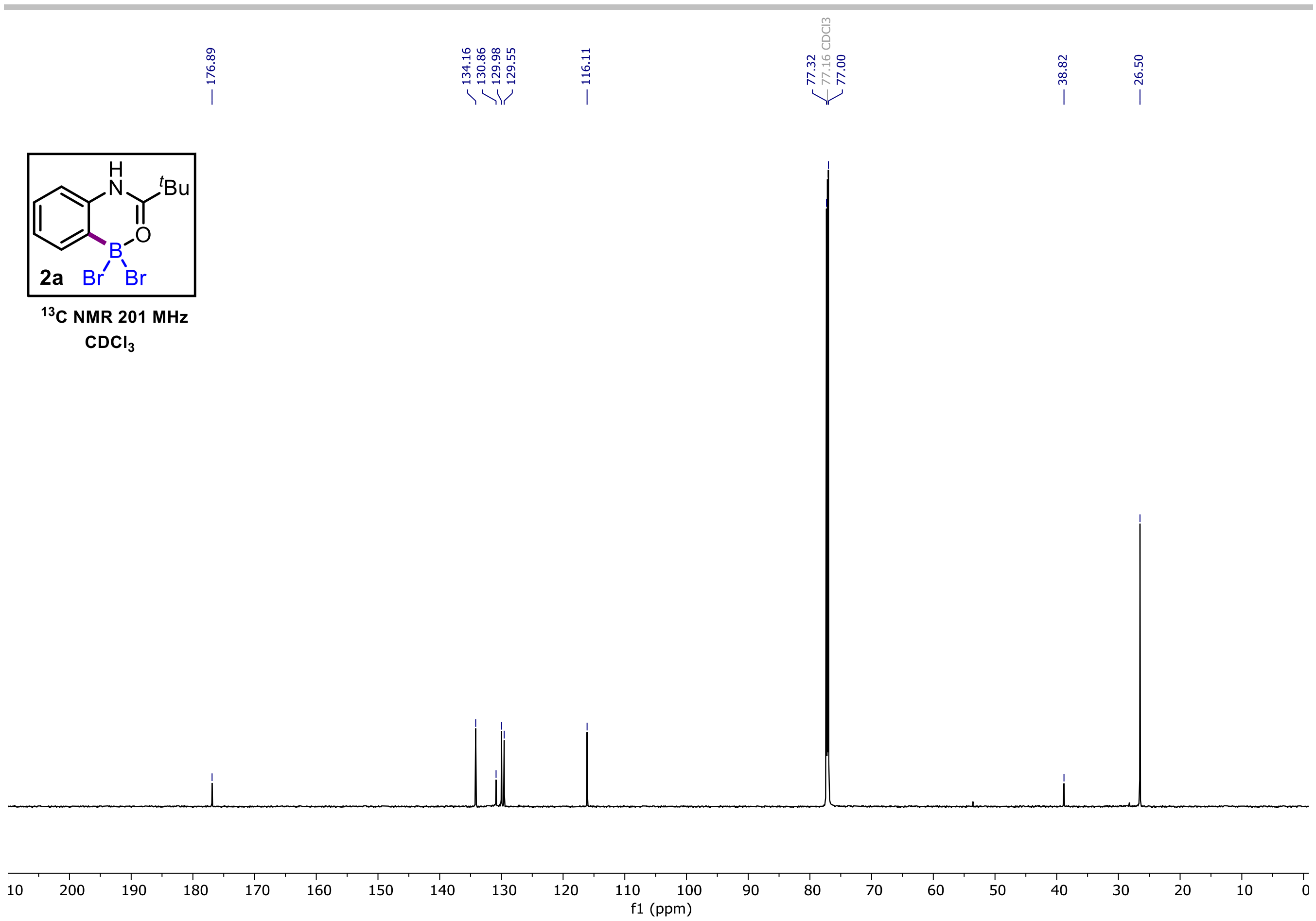
176.89
134.16
130.86
129.98
129.55
116.11
77.32
77.16 CDCl3
77.00
38.82
26.50
H
N
tBu
O
B
Br
Br
2a
13C NMR 201 MHz
CDCl3
10
200
190
180
170
160
150
140
130
120
110
100
90
80
70
60
50
40
30
20
10
0
f1 (ppm)

3. Applications

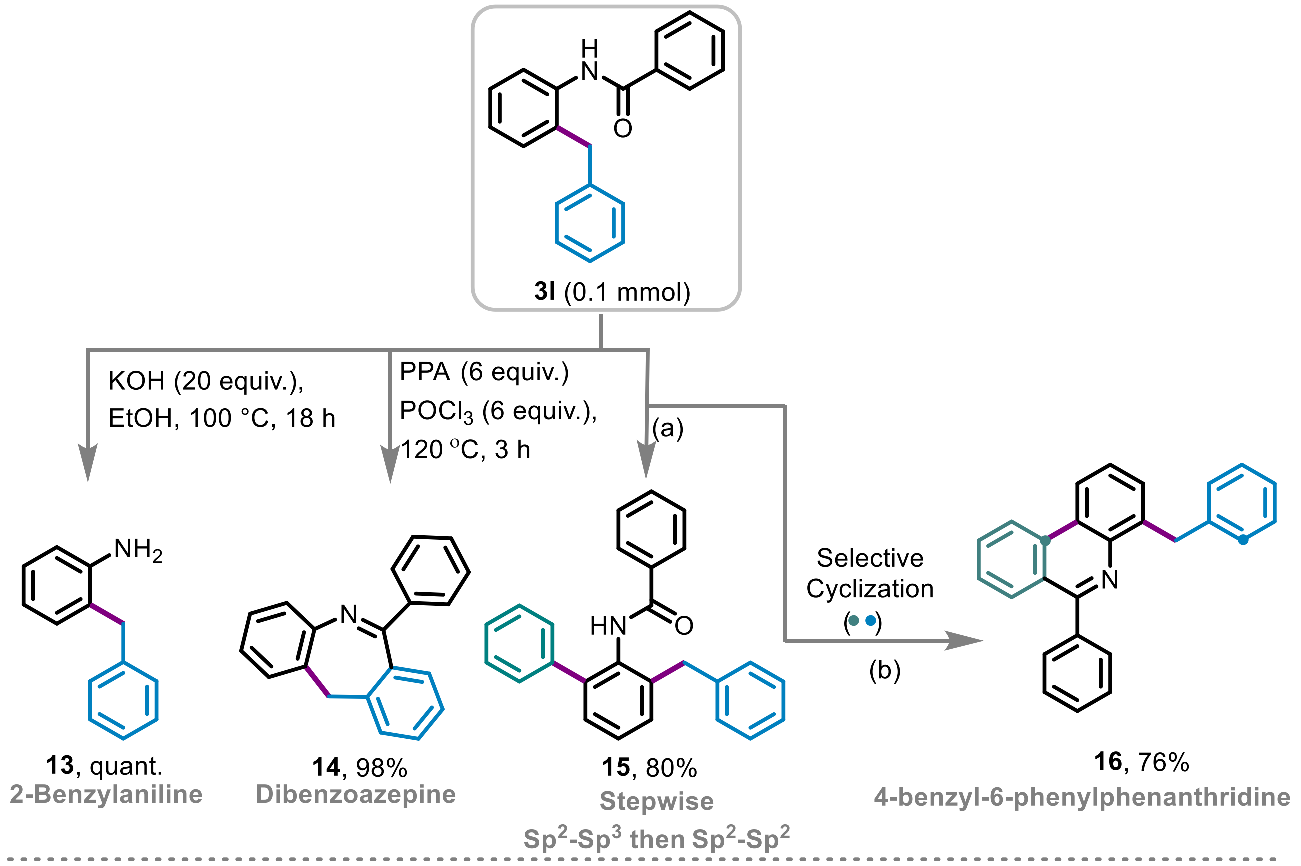

3.1 Synthesis of 2-benzylaniline:Hydrolysis of amide (13)

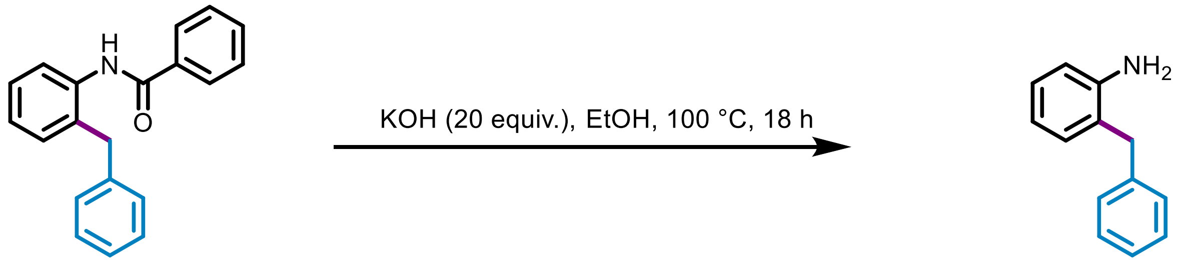

3l (0.1 mmol) **13,** quant

To a dry 1 mL, screw top V-Vial®, equipped with a stir bar, *N*-(2-benzylphenyl)benzamide (**3l**) (28.74 mg, 0.1 mmol, 1 equiv.), was dissolved in 0.5 mL EtOH. To this, KOH (110 mg, 2 mmol, 20 equiv.) was added and the reaction mixture was refluxed on preheated stirrer at 100 ºC for 18 h. The reaction mixture was allowed to reach room temperature and transfered into 25 mL beaker. The vial was washed with 10 mL EtOAc and 5 mL distilled water to transfer the complete mixture into the beaker. The solution was extracted with additional 5 mL EtOAc. The combined organic phases were washed with brine, dried using anhydrous sodium sulfate, and concentrated under *vacuo* to afford the 2-benzylaniline **7** in quantitative yield.

Spectral data 2-benzylaniline (13):[(4)] **^{1}H NMR (600 MHz, CDCl$_3$) δ=** 7.31 (t, *J* = 7.5 Hz, 2H), 7.24 (d, *J* = 7.3 Hz, 1H), 7.21 (d, *J* = 8.4 Hz, 2H), 7.12 (td, *J* = 7.6, 1.6 Hz, 1H), 7.08 (dd, *J* = 7.5, 1.6 Hz, 1H), 6.79 (td, *J* = 7.4, 1.2 Hz, 1H), 6.70 (dd, *J* = 7.9, 1.2 Hz, 1H), 3.93 (s, 2H), 3.51 (bs, 2H); **^{13}C{^{1}H} NMR (151 MHz, CDCl$_3$) δ=** 144.8, 139.5, 131.0, 128.8, 128.6, 127.8, 126.5, 125.2, 118.9, 116.0, 38.2.

3.2 Synthesis of 6-phenyl-11*H*-dibenzo[*b*,*e*]azepine (14)

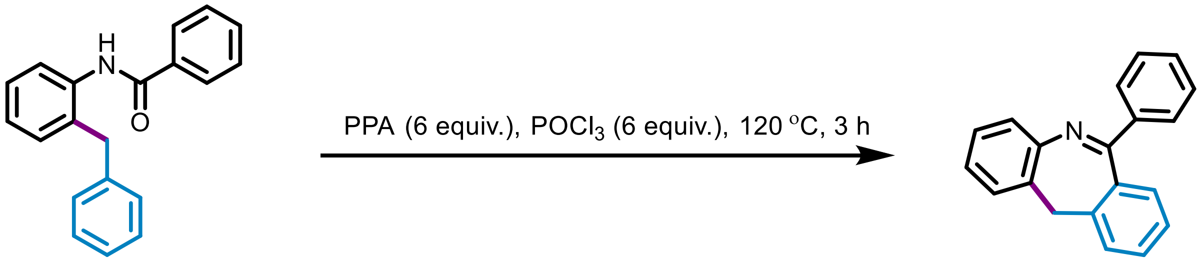

To a dry 1 mL, screw top V-Vial®, equipped with a stir bar, *N*-(2-benzylphenyl)benzamide (**3l**) (28.74 mg, 0.1 mmol, 1 equiv.), was added in a mixture of polyphosphoric acid (58 mg, 0.6 mmol, 6 equiv.) and phosphorus oxychloride (56 µl, 0.6 mmol, 6 equiv.). The reaction mixture was heated on preheated stirrer at 120 ºC for 3 h. The reaction mixture was allowed to reach room temperature and transfered in 25 mL beaker containing ice water. The vial was washed with 10 mL CH_2Cl_2 and 2 mL distilled water to transfer the complete mixture into the beaker. The vial and aqueous layer was neutralise with ammonia solution (25%) and extracted with additional 5 mL CH_2Cl_2. The combined organic phases were washed with brine, dried using anhydrous sodium sulfate, and concentrated under *vacuo* to afford the 6-phenyl-11*H*-dibenzo[*b*,*e*]azepine **14** in 98% yield.

Spectral data 6-phenyl-11*H*-dibenzo[*b*,*e*]azepine (14):[(5)] Beige color solid; **^{1}H NMR (600 MHz, CDCl$_3$) δ=** 7.86 – 7.84 (m, 2H), 7.50 – 7.38 (m, 5H), 7.34 (dd, *J* = 7.7, 1.3 Hz, 1H), 7.28 – 7.21 (m, 3H), 7.19 (td, *J* = 7.5, 1.3 Hz, 1H), 7.13 (td, *J* = 7.4, 1.3 Hz, 1H), 3.70 (dd, *J* = 33.0, 12.6 Hz, 2H); **^{13}C{^{1}H} NMR (151 MHz, CDCl$_3$) δ=** 167.1, 145.8, 143.8, 141.2, 133.0, 131.7, 131.2, 130.3, 130.0, 129.9, 128.3, 127.1 (2C), 126.6, 126.2, 125.8, 125.7, 39.4.

3.3 Synthesis of *N*-(3-benzyl-[1,1'-biphenyl]-2-yl)benzamide: Arylation (15)

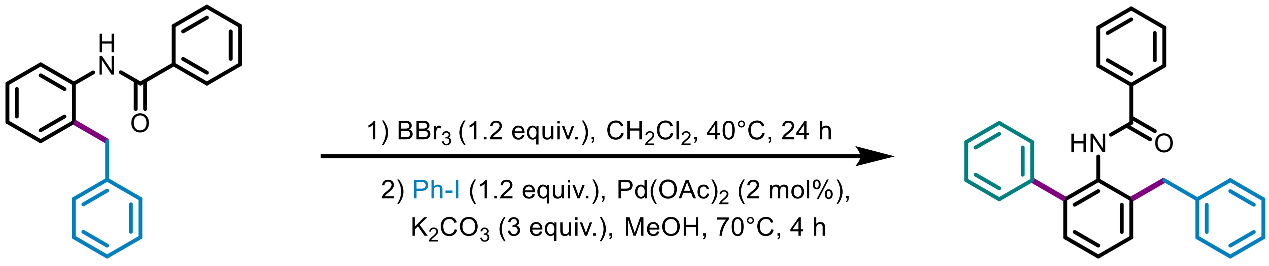

Step i) To a dry 5 mL, screw top V-Vial®, equipped with a rubber septum, stir bar, *N*-(2-benzylphenyl)benzamide (**3l**) (86.21 mg, 0.3 mmol, 1 equiv.) in anhydrous CH_2Cl_2 (1 mL) under a

nitrogen atmosphere was added dropwise BBr_3 (0.36 mL 0.18 mmol, 1.2 equiv., 1M solution in CH_2Cl_2). After the complete addition of BBr_3, the reaction mixture was stirred at 40 °C for 24 h after which the solvent was removed under reduced pressure.

Step ii) To the crude residue from step i) were added K_2CO_3 (125 mg, 0.9 mmol, 3 equiv.) and palladium acetate (1.35 mg, 2 mol%, 0.02 equiv.) under nitrogen atmosphere. To this mixture, 2.5 mL degassed methanol was added along with iodobenzene (73.45 mg, 0.36 mmol, 1.2 equiv.) and the reaction mixture was heated at 70 °C for 4 h. The reaction was allowed to reach room temperature and diluted with 2 mL ethyl acetate and filtered through a pad of celite. The celite was washed with additional 15 mL of ethyl acetate and the filtrate was evaporated *in vacuo* to afford the crude product, which was purified using automated column chromatography (pentane/EtOAc, 85:15).

Spectral data: Off white solid; **Rf:** 0.24 (hexane/EtOAc, 90:10); **M.P.**= 160-162 °C; **^{1}H NMR (600 MHz, $CDCl_3$) δ=** 7.49 (d, *J* = 7.2 Hz, 2H), 7.46 (td, *J* = 7.8, 1.1 Hz, 1H), 7.39 – 7.22 (m, 12H), 7.19 (t, *J* = 7.3 Hz, 1H), 7.13 (d, *J* = 8.5 Hz, 2H), 7.06 (bs, 1H), 4.10 (s, 2H); **^{13}C{^{1}H} NMR (151 MHz, $CDCl_3$) δ=** 167.0, 140.6, 140.6, 139.8, 139.6, 134.5, 132.6, 131.8, 130.4, 129.1, 129.0, 128.9, 128.7, 128.6, 128.5, 127.9, 127.6, 127.3, 126.3, 38.9; **IR (neat, cm^{-1})** = 3224 (br), 2954 (br), 2852 (br), 1639 (s), 1566 (s), 1547 (s), 1514 (m), 1503 (s), 1485 (s), 1452 (m), 1306 (s), 1296 (s), 1025 (m), 759 (s), 691 (s); **HRMS (ESI) (m/z):** calculated for $[M+H]^+$ $C_{26}H_{22}NO$ 364.1695; found 364.1702.

3.4 Synthesis of 4-benzyl-6-phenylphenanthridine (16): Selective cyclization

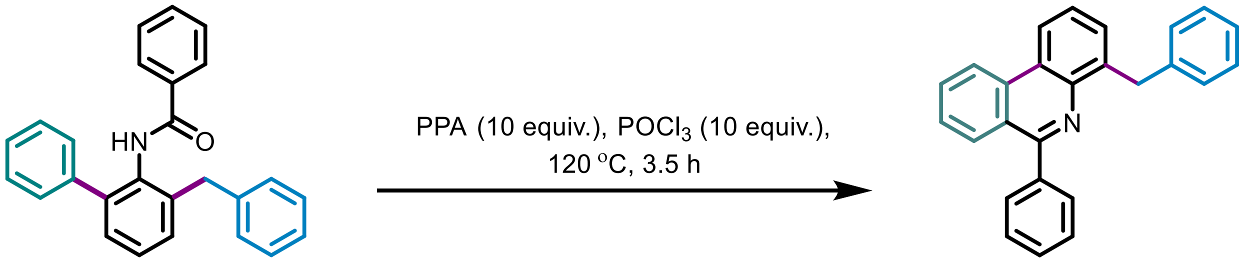

15 (0.07 mmol) **16**, 76%

To a dry 1 mL, screw top V-Vial®, equipped with a stir bar, *N*-(3-benzyl-[1,1'-biphenyl]-2-yl)benzamide (**15**) (25.44 mg, 0.07 mmol, 1 equiv.), was added in a mixture of polyphosphoric acid (68 mg, 0.7 mmol, 10 equiv.) and phosphorus oxychloride (33 µl, 0.7 mmol, 10 equiv.). The reaction mixture was heated on preheated stirrer at 120 °C for 3.5 h. The reaction mixture was allowed to reach room temperature and transfered in 25 mL beaker containing ice water. The vial was washed with 10 mL CH_2Cl_2 and 2 mL distilled water to transfer the complete mixture into the beaker. The vial and aqueous layer was neutralise with ammonia solution (25%) and extracted with additional 5 mL CH_2Cl_2. The combined organic phases were washed with brine, dried using anhydrous sodium sulfate, and concentrated under *vacuo* to afford the 4-benzyl-6-phenylphenanthridine (16) in 76% yield.

Spectral data: Beige color solid; **Rf:** 0.72 (hexane/EtOAc,80:20); **^{1}H NMR (600 MHz, $CDCl_3$) δ=** 8.71 (d, *J* = 8.3 Hz, 1H), 8.51 (dd, *J* = 7.7, 1.9 Hz, 1H), 8.22 (dd, *J* = 8.3, 1.3 Hz, 1H), 7.84 (ddd, *J* = 8.3, 6.9, 1.3 Hz, 1H), 7.82 – 7.77 (m, 2H), 7.65 – 7.54 (m, 6H), 7.38 (d, *J* = 7.9 Hz, 2H), 7.28 (t, *J* = 7.7 Hz, 2H), 7.19 (t, *J* = 7.4 Hz, 1H), 4.78 (s, 2H); **^{13}C{^{1}H} NMR (151 MHz, $CDCl_3$) δ=** 159.5, 142.3, 142.1, 141.3, 140.4, 134.0, 130.4, 130.3, 129.6, 129.4, 128.7, 128.3, 128.3 (2C), 127.0, 126.8, 125.8, 124.9, 123.8, 122.6, 120.4, 37.6; **HRMS (ESI) (m/z):** calculated for $[M+H]^+$ $C_{26}H_{20}N$ 346.1590; found 346.1598.

4. Single crystal X-ray diffraction

Recrystallization of compound **16** from a 1:1 (v/v) mixture of ethyl acetate and hexane at room temperature yielded single crystals suitable for X-ray diffraction analysis.

A suitable single crystal of **16** data was collected on a Rigaku XtaL AB Synergy-DW diffractor equipped with a HyPix-Arc 150° detector using CuKα radition at λ = 1.54184 Å. The data diffraction was obtained and processed with CrysAlisPro software.[6,7] for the structure and the refinements were established by full-matrix least squares with SHELX-2018/3[8] using Olex2[9] software. Mercury[10] software was utilized as graphical interface. Gaussian absorption correction was applied for absorption effects.

Table S2. Crystal data and structure refinement for coumpound **16**.

Code	**16**
Structural formula	$C_{26}H_{19}N$
Molecular mass (g mol^{-1})	345.42
Data collection temp. (K)	293 (2)
Crystal system	Monoclinic
Space group	$P2_1/n$
A (Å)	5.66793 (8)
B (Å)	14.5463 (3)
C (Å)	22.3274 (4)
α (°)	90
β (°)	92.7216 (14)
γ (°)	90
Volume (Å^3)	1838.76 (6)
Z	4
Dc, calc density (cm^3)	1.248
μ (mm^{-1})	0.549
F(000)	728.0
Crystal size (mm^3)	0.448 × 0.184 × 0.124
Radiation	Cu Kα (λ = 1.54184)
2θ range for data collection (°)	7.256 to 150.876
Index ranges	$-6 \leq h \leq 4$, $-18 \leq k \leq 17$, $-28 \leq l \leq 27$

Reflections collected	16668
Independent reflections	3552 [R_{int} = 0.0302, R_{sigma} = 0.0279]
Data/restraints/parameters	3552/0/245
Goodness-of-fit on F^2	1.098
Final R indexes [I>=2σ (I)]	R_1 = 0.0425, wR_2 = 0.1154
Final R indexes [all data]	R_1 = 0.0555, wR_2 = 0.1407
Largest diff. peak/hole / e Å-3	0.20; -0.20
CCDC no.	2400705

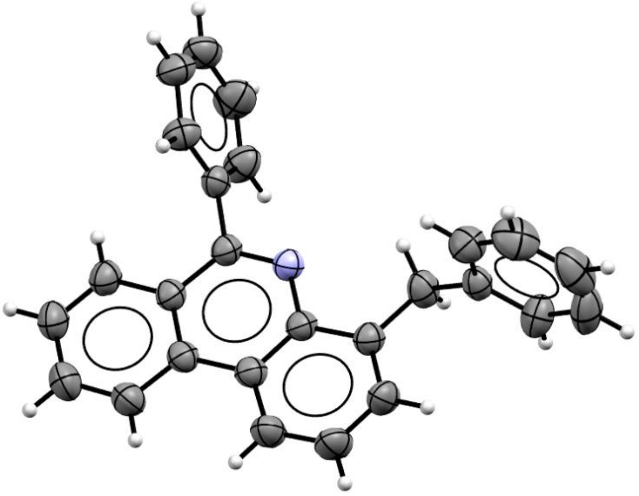

Figure 1. ORTEP of compound **16**: C (grey), N (purple) and H (white); Ellipsoid contour probability level (50%)

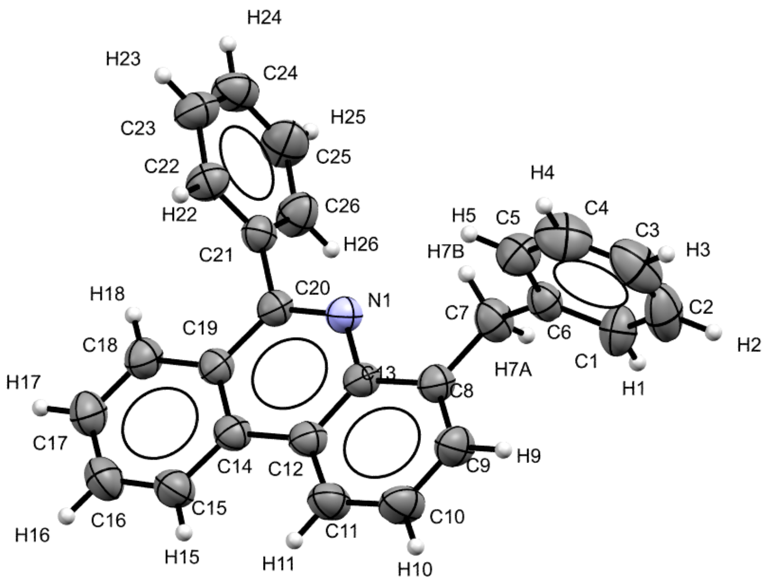

5. References

1) a) Lv, J.; Chen, X.; Xue, X. S.; Zhao, B.; Liang, Y.; Wang, M.; Jin, L.; Yuan, Y.; Han, Y.; Zhao, Y.; Lu, Y.; Zhao, J.; Sun, W. Y.; Houk, K. N.; Shi, Z. Metal-Free Directed Sp2-C–H Borylation. *Nature* **2019**, 575, 336–340; b) Lv, J.; Zhao, B.; Yuan, Y.; Han, Y.; Shi, Z. Boron-Mediated Directed Aromatic C–H Hydroxylation. *Nat. Commun.* **2020**, *11*, 1316;

2) a) Shinde, G. H.; Ghotekar, G. S.; Amombo Noa, F. M.; Öhrström, L.; Norrby, P. O.; Sundén, H. Regioselective Ortho Halogenation of N-Aryl Amides and Ureas via Oxidative Halodeboronation: Harnessing Boron Reactivity for Efficient C-Halogen Bond Installation. *Chem. Sci.* **2023**, *14*, 13429–13436; b) Suffert, J. Simple direct titration of organolithium reagents using N-pivaloyl-o-toluidine and/or N-pivaloyl-o-benzylaniline. *J. Org. Chem*. **1989**, *54*, 509– 510.

3) Kang, K. H.; Do, J.; Park, Y. S. Asymmetric Synthesis of Trans-2,3-Disubstituted Indoline Derivatives. *J. Org. Chem.* **2012**, *77*, 808–812.

4) Manolikakes, G.; Schade, M. A.; Hernandez, C. M.; Mayr, H.; Knochel, P. Negishi Cross-Couplings of Unsaturated Halides Bearing Relatively Acidic Hydrogen Atoms with Organozinc Reagents. *Org. Lett.* **2008**, *10* , 2765–2768.

5) Balakrishna, B.; Bauzá, A.; Frontera, A.; Vidal-Ferran, A. Asymmetric Hydrogenation of Seven-Membered C=N-Containing Heterocycles and Rationalization of the Enantioselectivity. *Chem. - A Eur. J.* **2016**, *22*, 10607–10613.

6) Crysalis CCD; Oxford Diffraction Ltd: Abingdon, Oxfordshire, UK, 2005.

7) Crysalis RED; Oxford Diffraction Ltd: Abingdon, Oxfordshire, UK, 2005.

8) Sheldrick, G. M. Crystal structure refinement with SHELXL. *Acta Crystallogr., Sect. C: Struct. Chem.* **2015**, *71*, 3– 8.

9) Dolomanov, O. V.; Bourhis, L. J.; Gildea, R. J.; Howard, J. A. K.; Puschmann, H. OLEX2: a complete structure solution, refinement and analysis program. *J. Appl. Crystallogr*. **2009**, *42*, 339– 341.

10) Macrae, C. F.; Bruno, I. J.; Chisholm, J. A.; Edgington, P. R.; McCabe, P.; Pidcock, E.; Rodriguez-Monge, L.; Taylor, R.; Van De Streek, J.; Wood, P. A. *J. Appl. Crystallogr*. **2008**, *41*, 466-470.

6. NMR spectra of target compounds

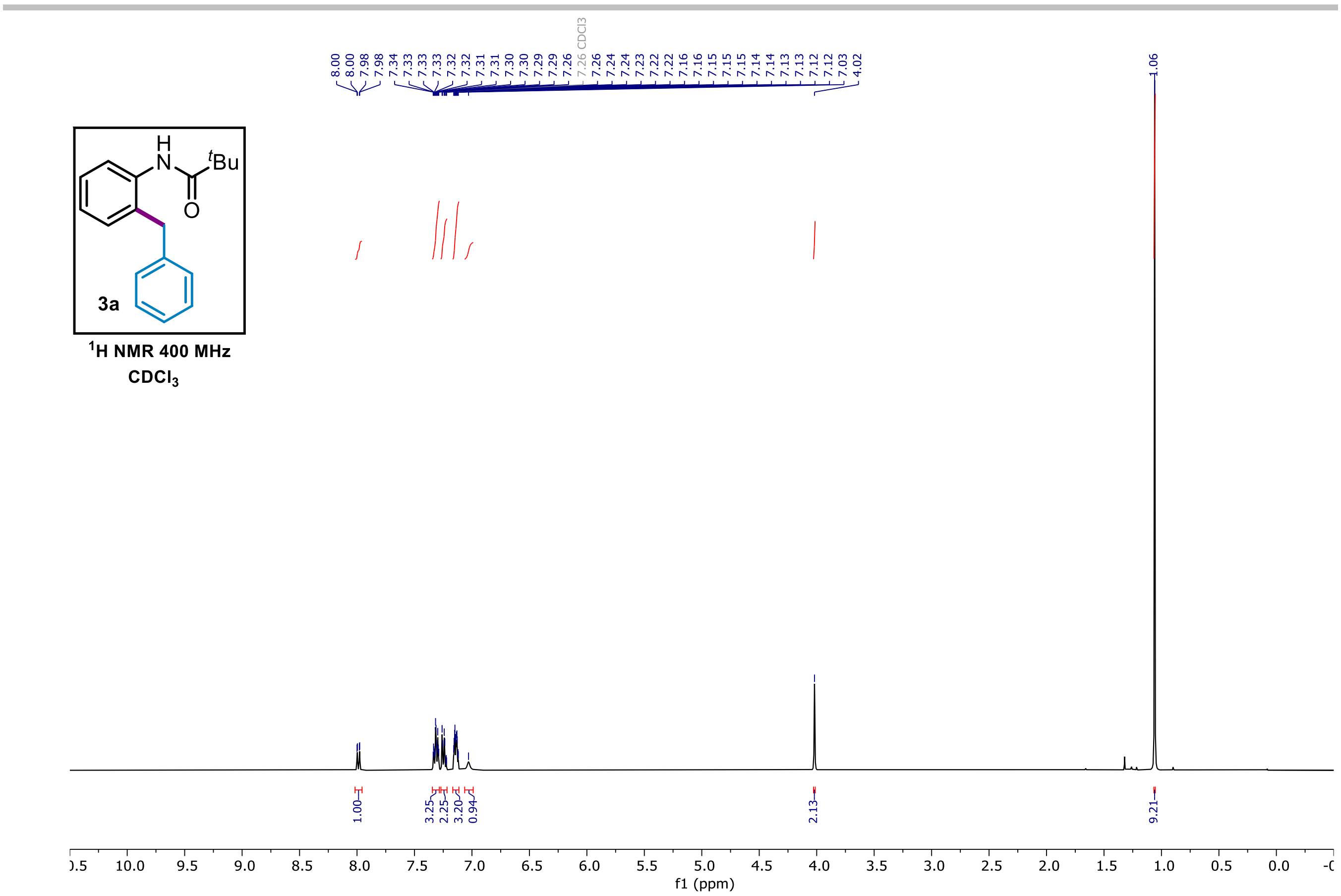

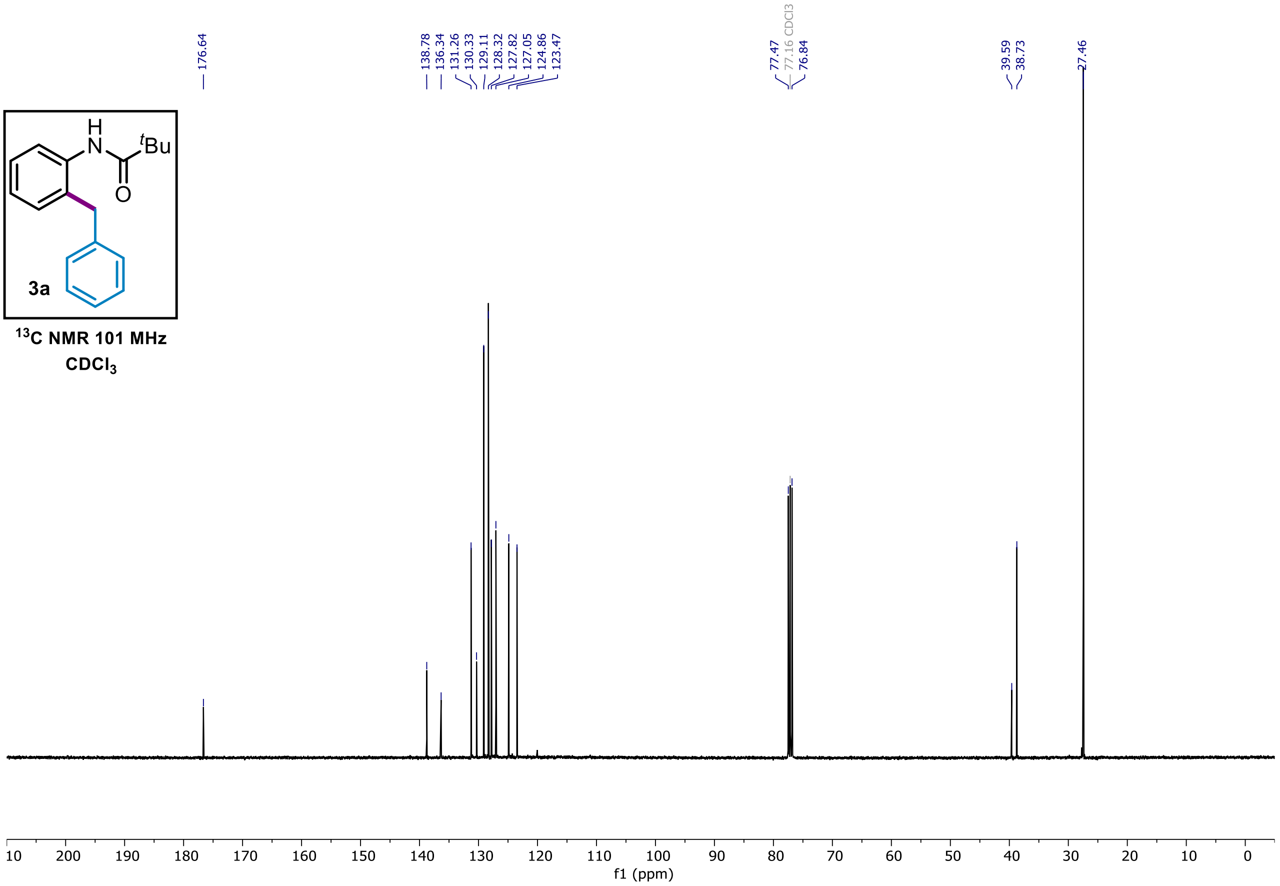

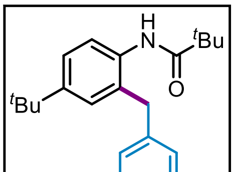

3b

^{1}H NMR 800 MHz
$CDCl_3$

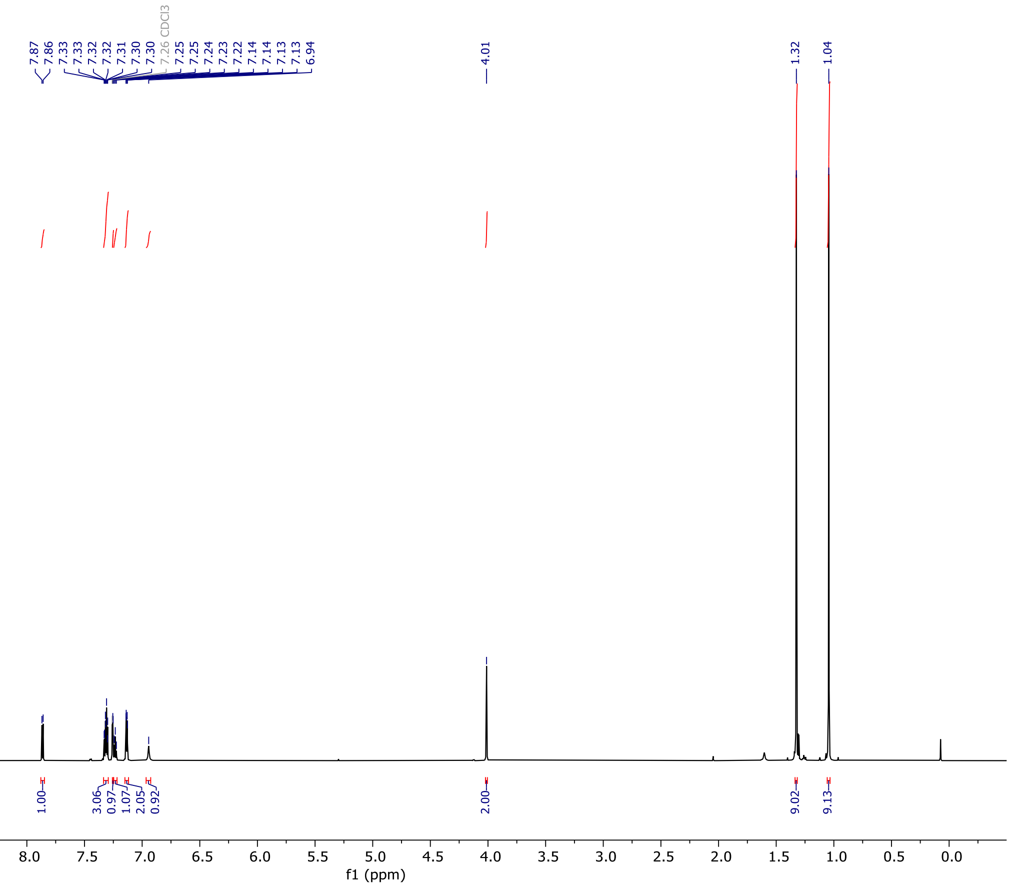

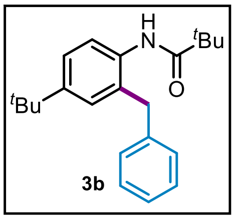

3b

^{13}C NMR 201 MHz
$CDCl_3$

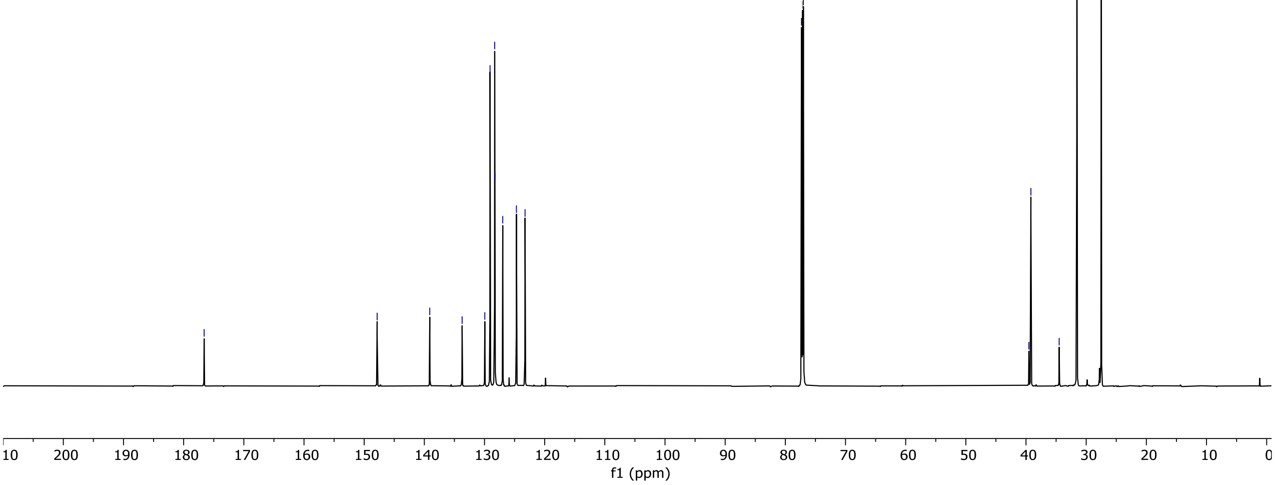

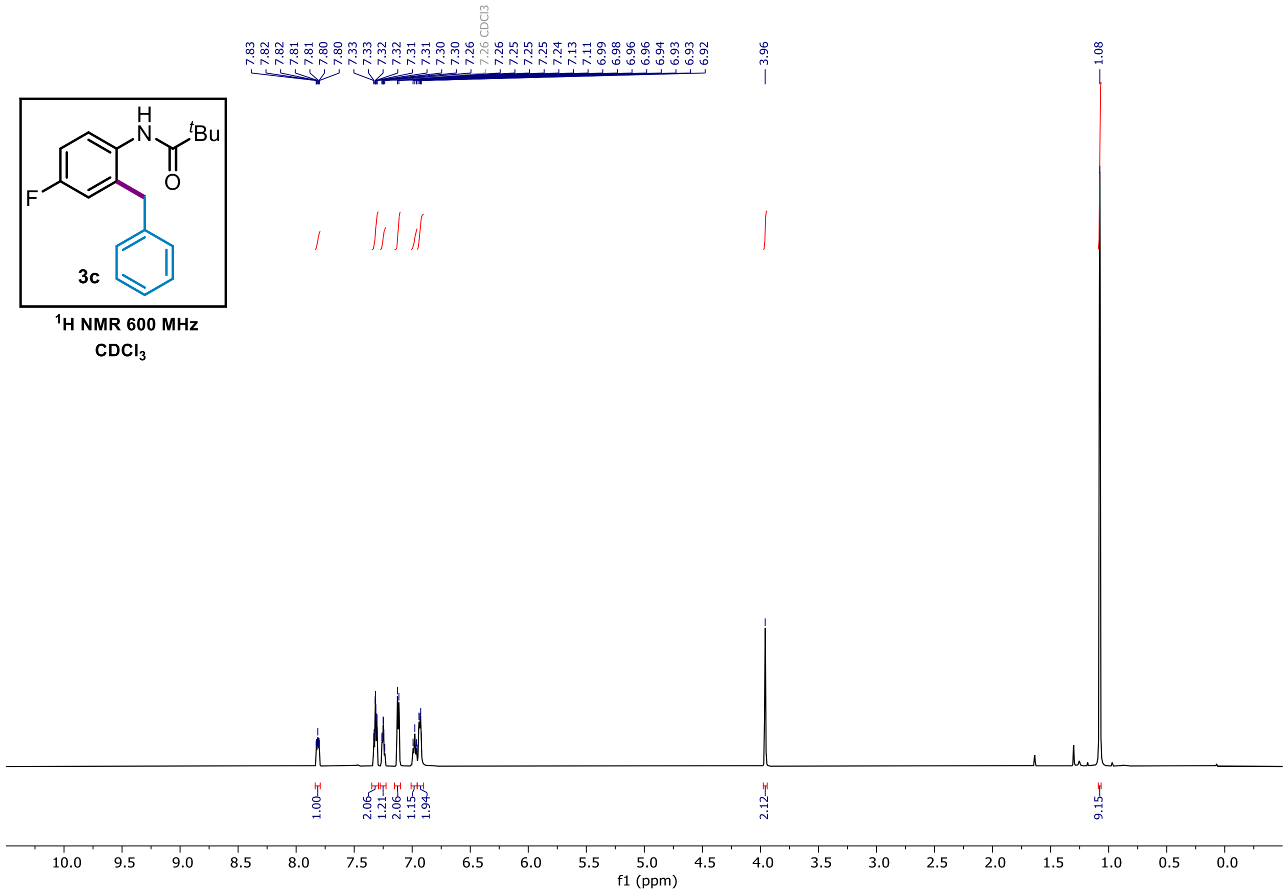
3c
1H NMR 600 MHz
CDCl3
f1 (ppm)

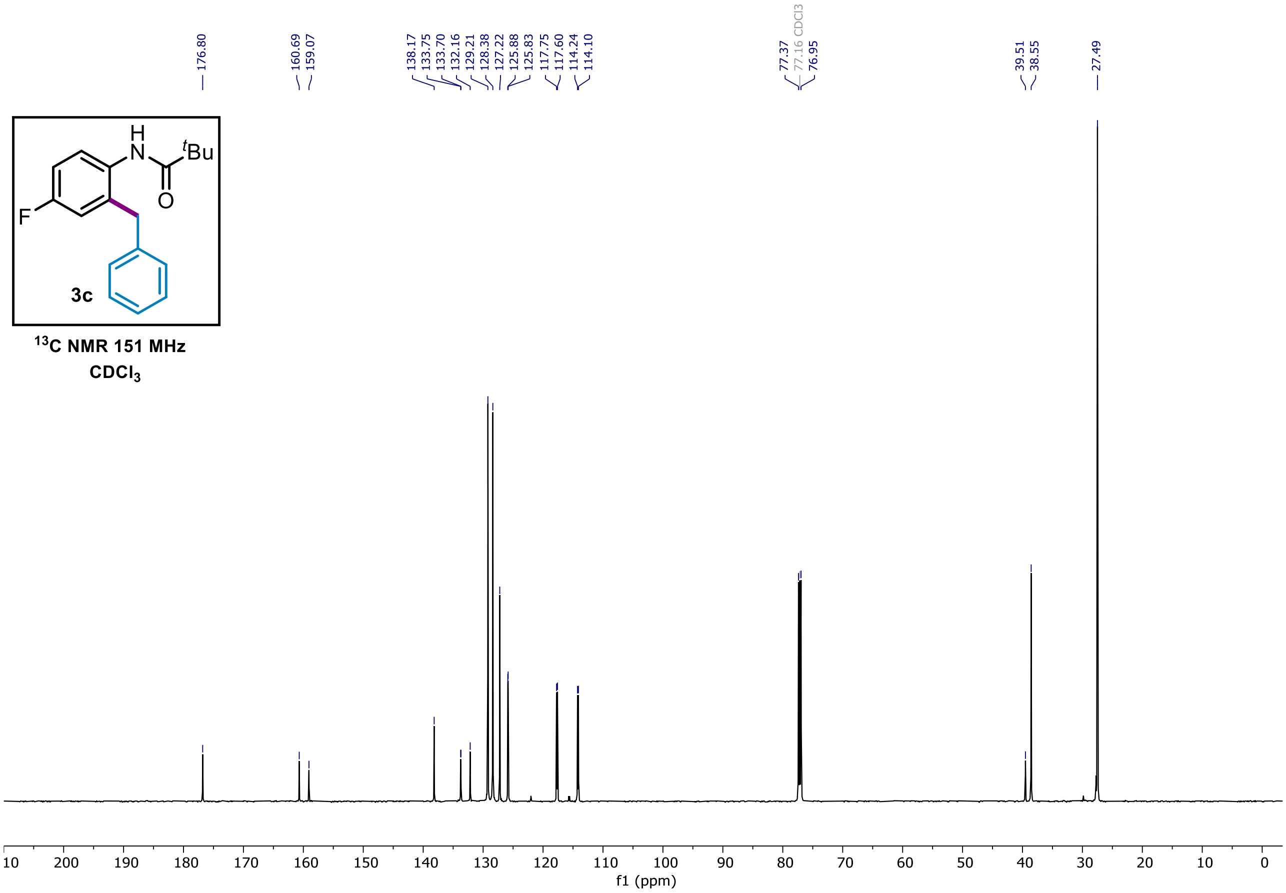
3c
13C NMR 151 MHz
CDCl3
f1 (ppm)

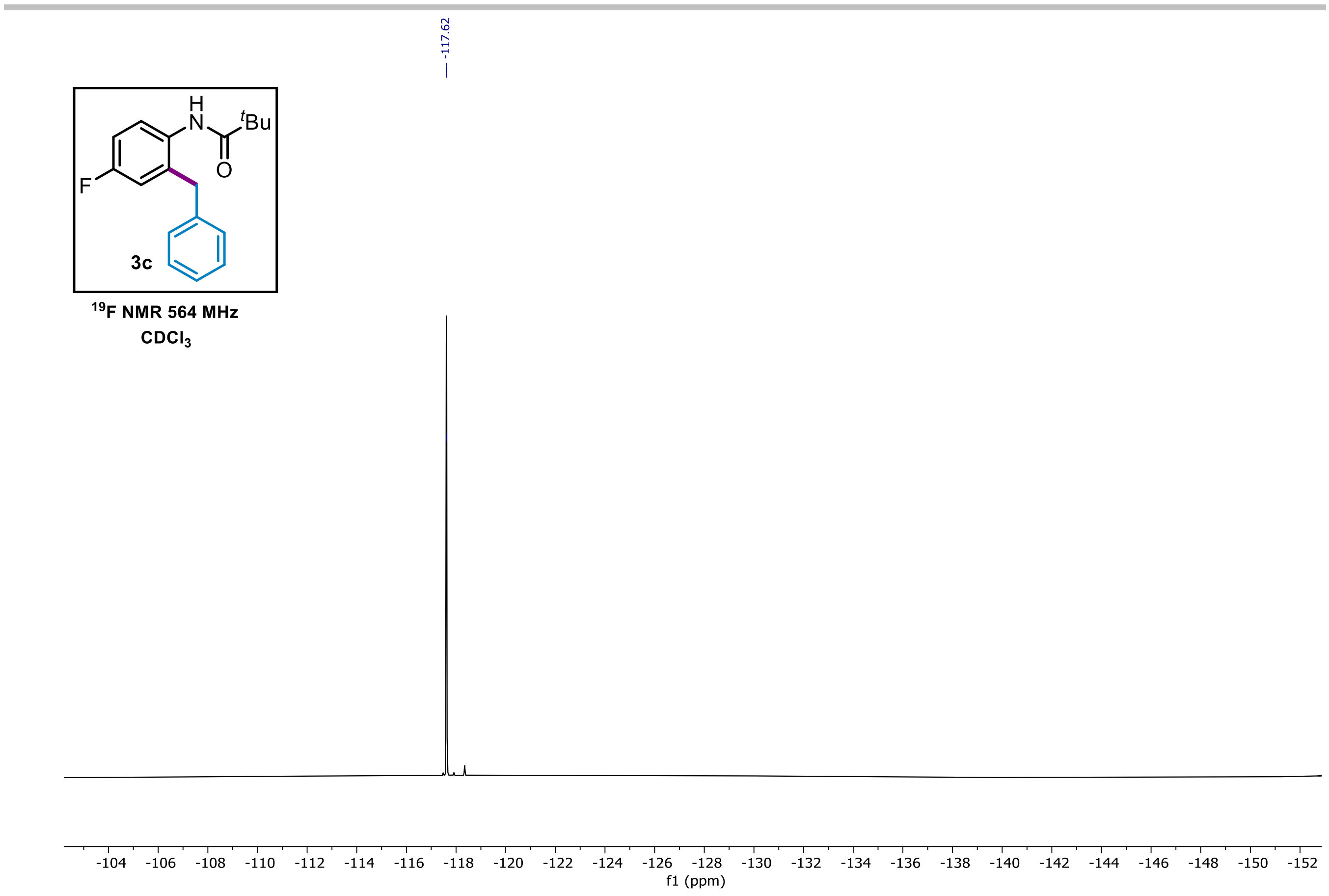

-117.62
H
N
tBu
O
F
3c
19F NMR 564 MHz
CDCl3
f1 (ppm)

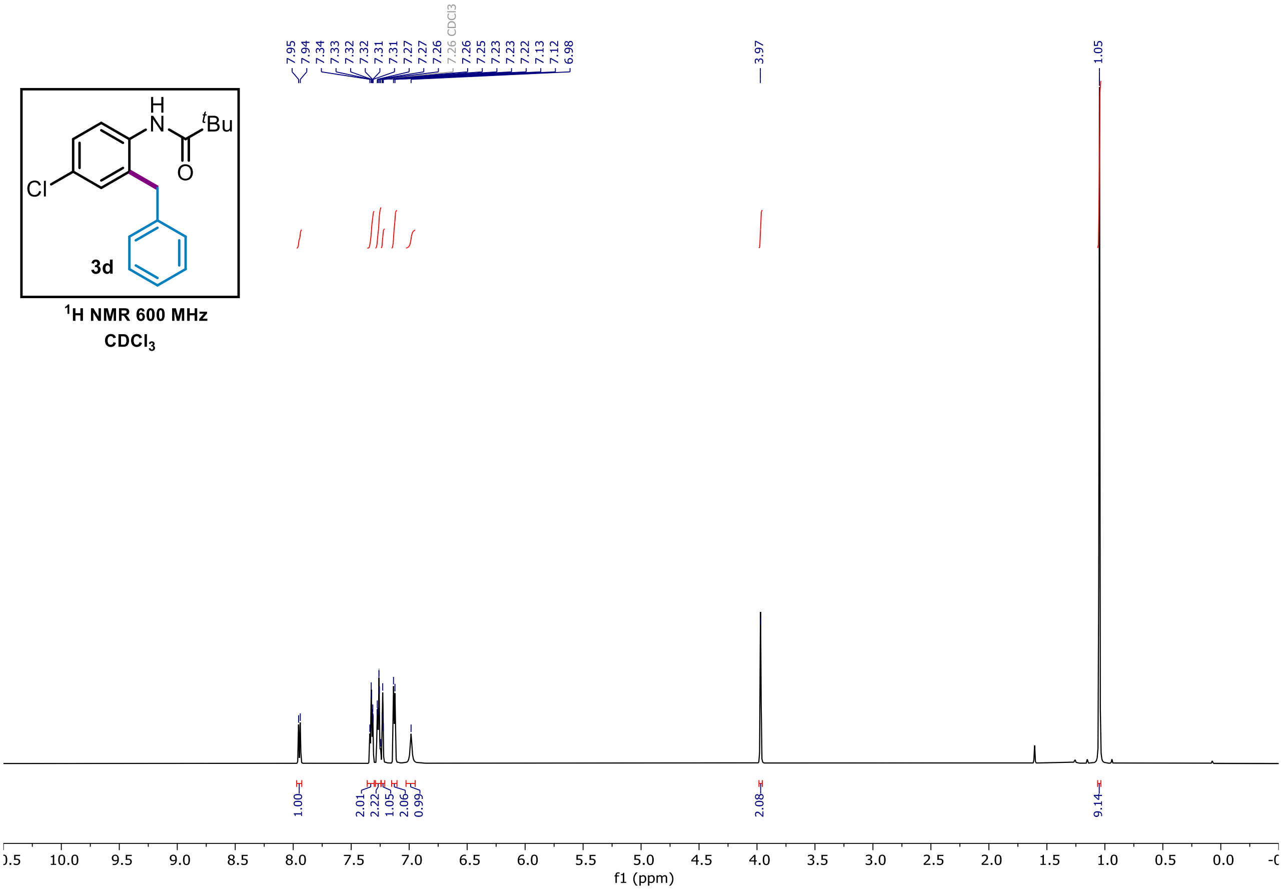

7.95
7.94
7.34
7.33
7.32
7.32
7.31
7.31
7.27
7.26
7.26 CDCl3
7.26
7.25
7.23
7.23
7.22
7.13
7.12
6.98
3.97
1.05
H
N
tBu
O
Cl
3d
1H NMR 600 MHz
CDCl3
1.00
2.01
2.22
1.05
2.06
0.99
2.08
9.14
f1 (ppm)

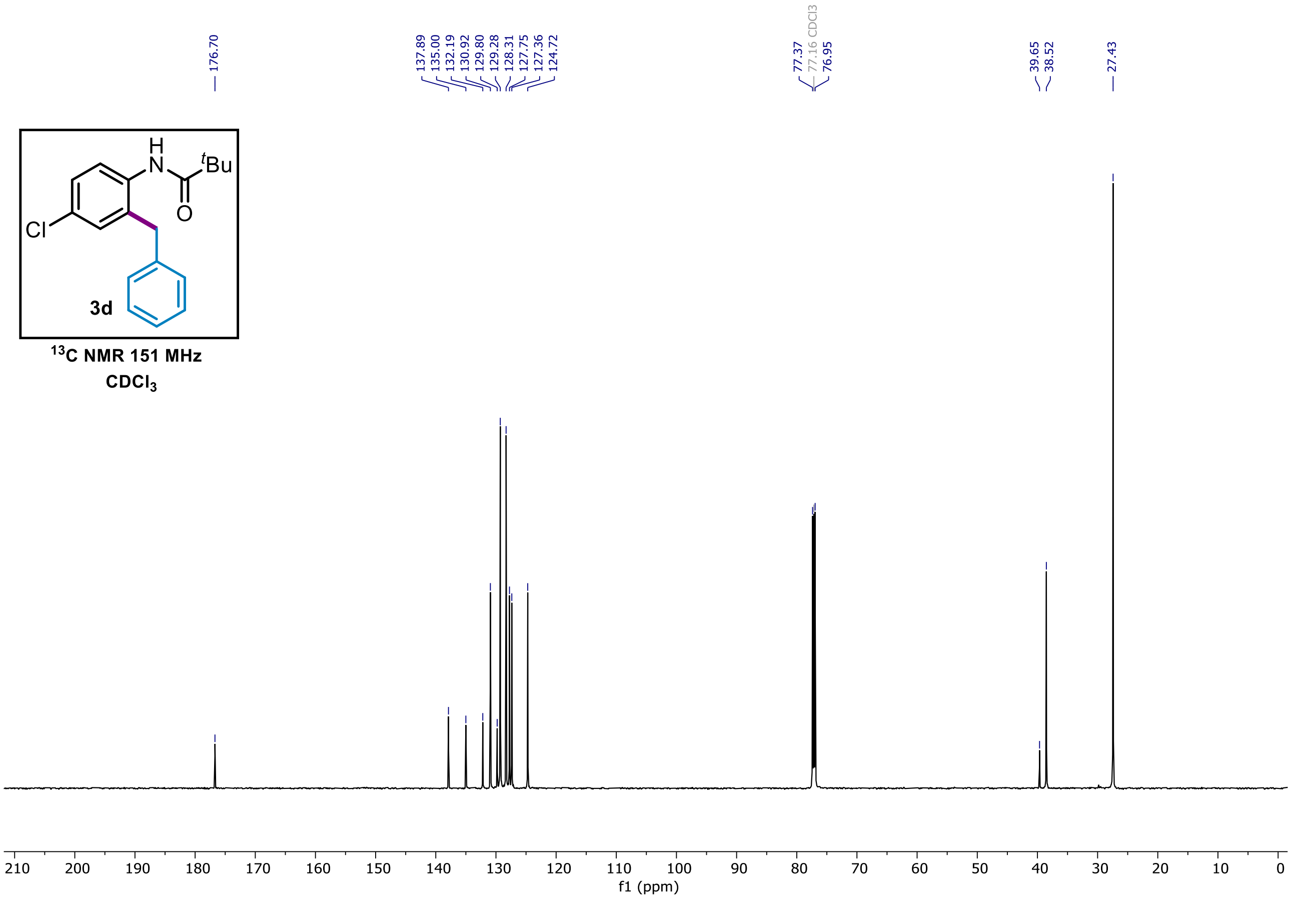

^{13}C NMR 151 MHz
$CDCl_3$

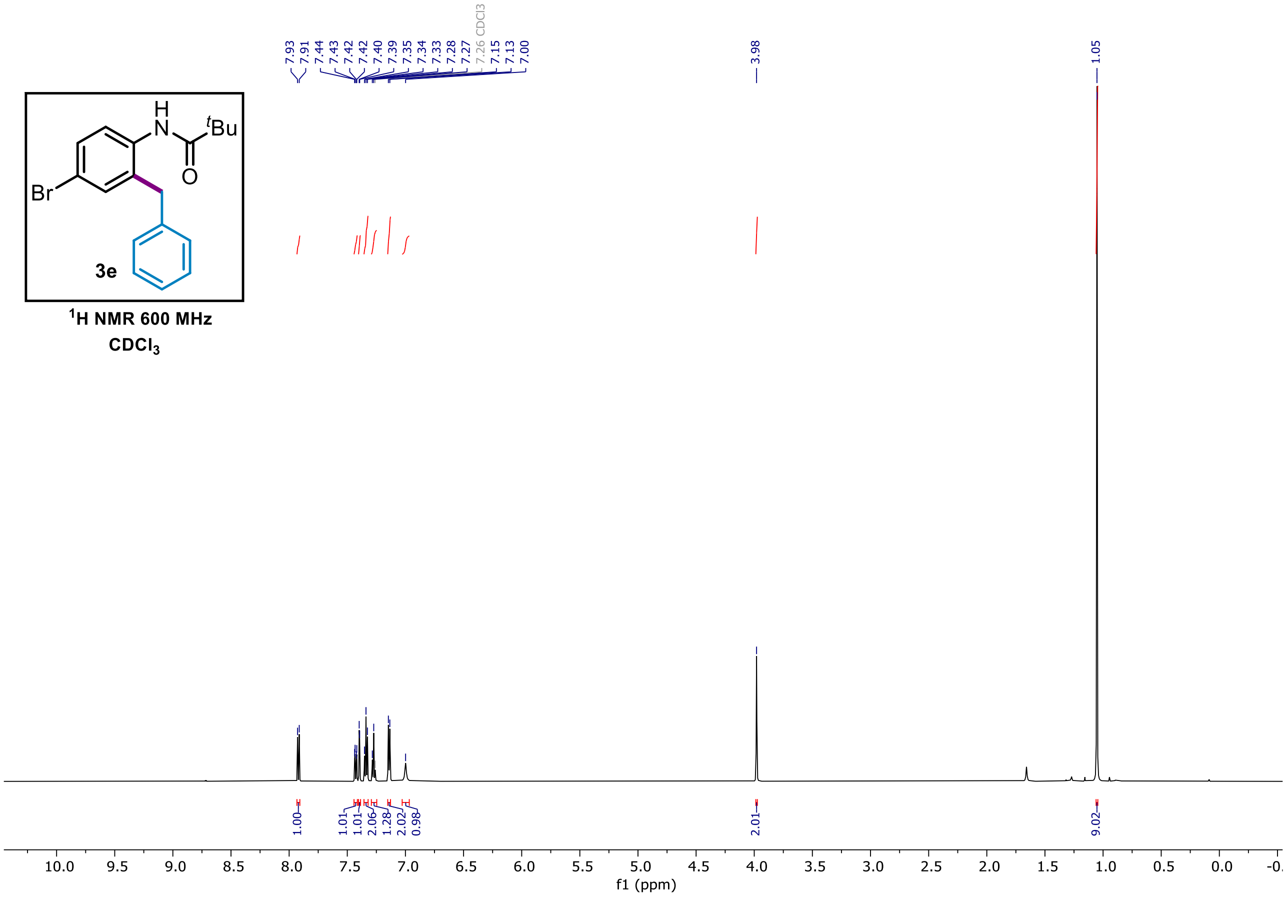

^{1}H NMR 600 MHz
$CDCl_3$

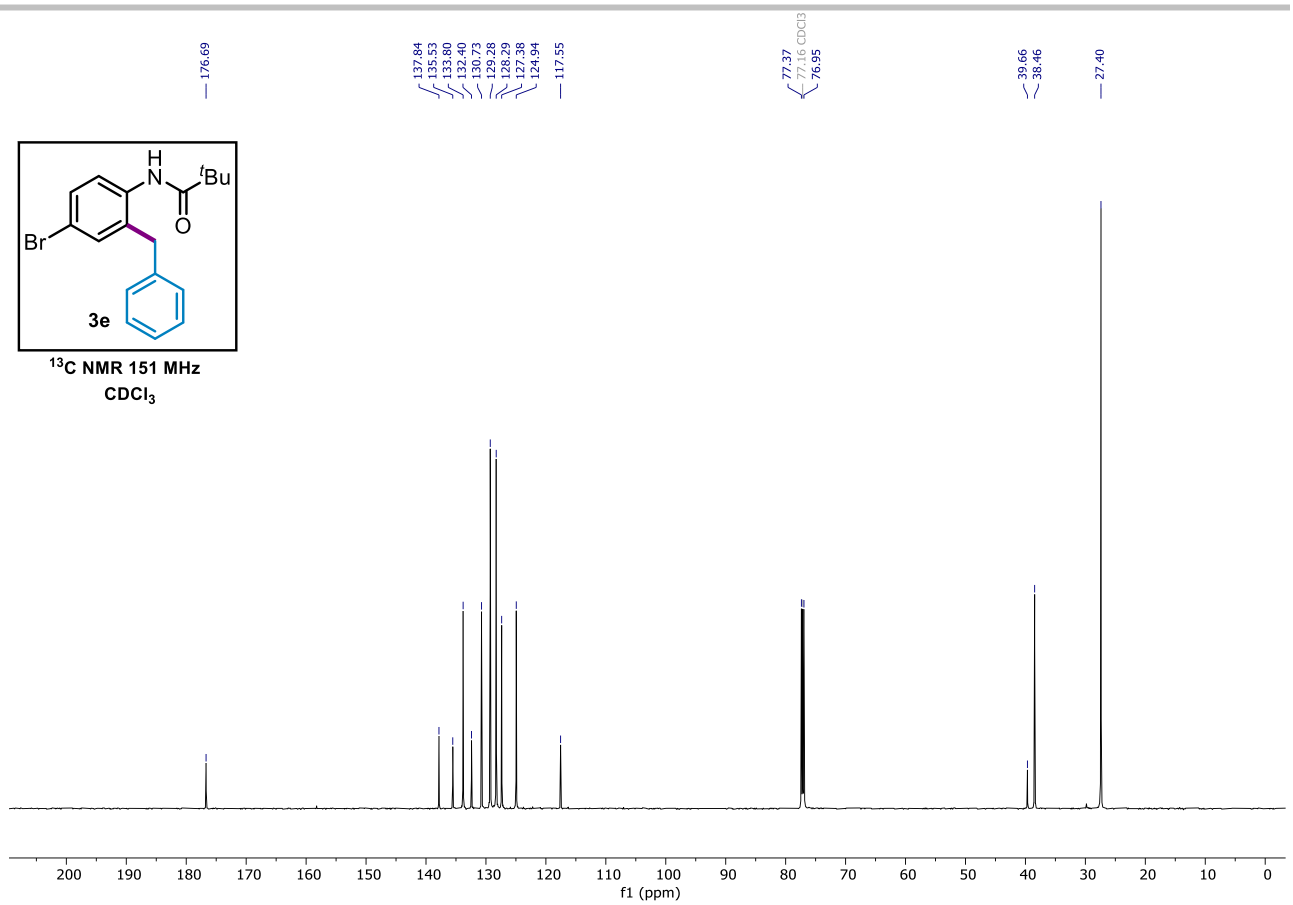
176.69
137.84
135.53
133.80
132.40
130.73
129.28
128.29
127.38
124.94
117.55
77.37
77.16 CDCl3
76.95
39.66
38.46
27.40
3e
13C NMR 151 MHz
CDCl3
f1 (ppm)

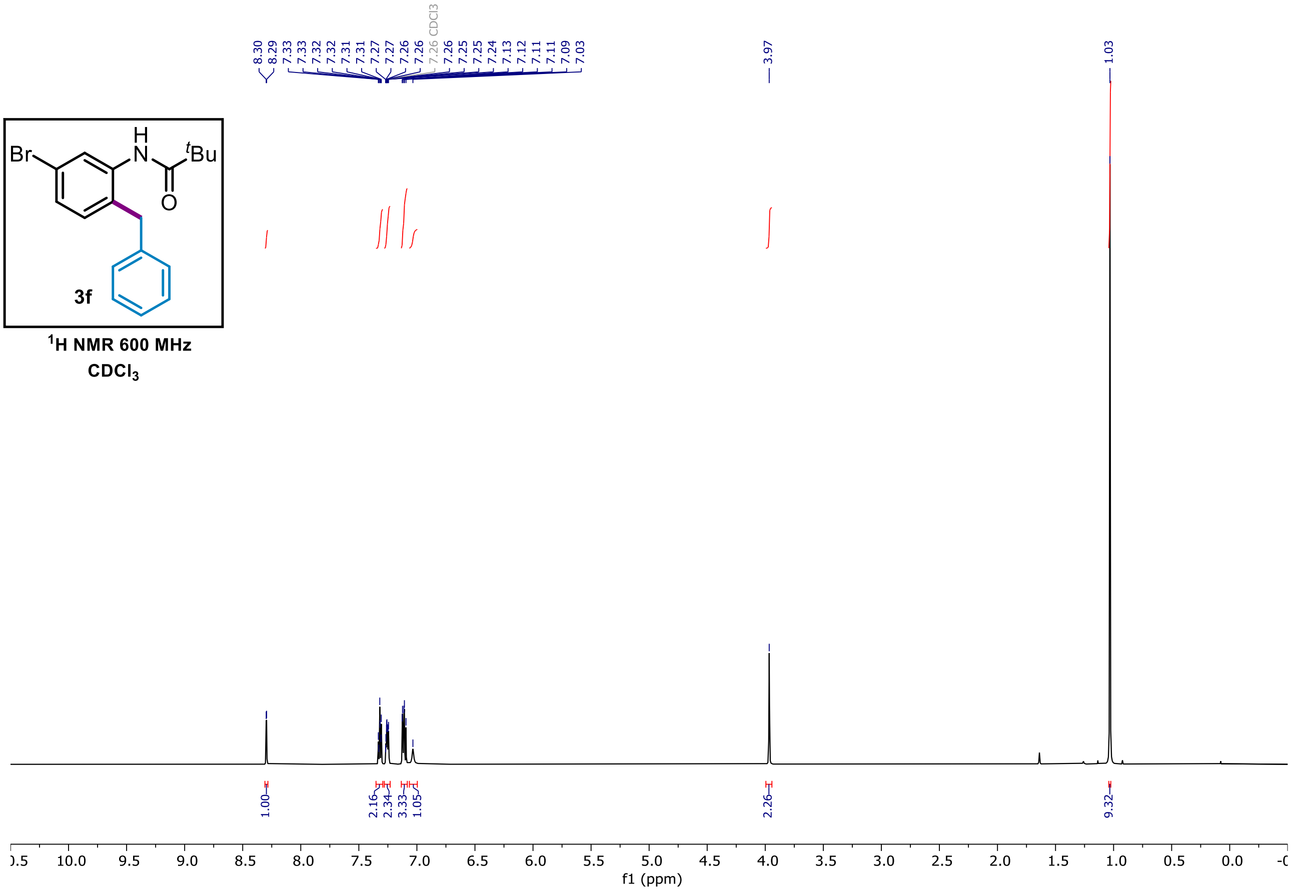
8.30
8.29
7.33
7.33
7.32
7.32
7.31
7.31
7.27
7.27
7.26
7.26 CDCl3
7.26
7.25
7.25
7.24
7.13
7.12
7.11
7.11
7.09
7.03
3.97
1.03
3f
1H NMR 600 MHz
CDCl3
1.00
2.16
2.34
3.33
1.05
2.26
9.32
f1 (ppm)

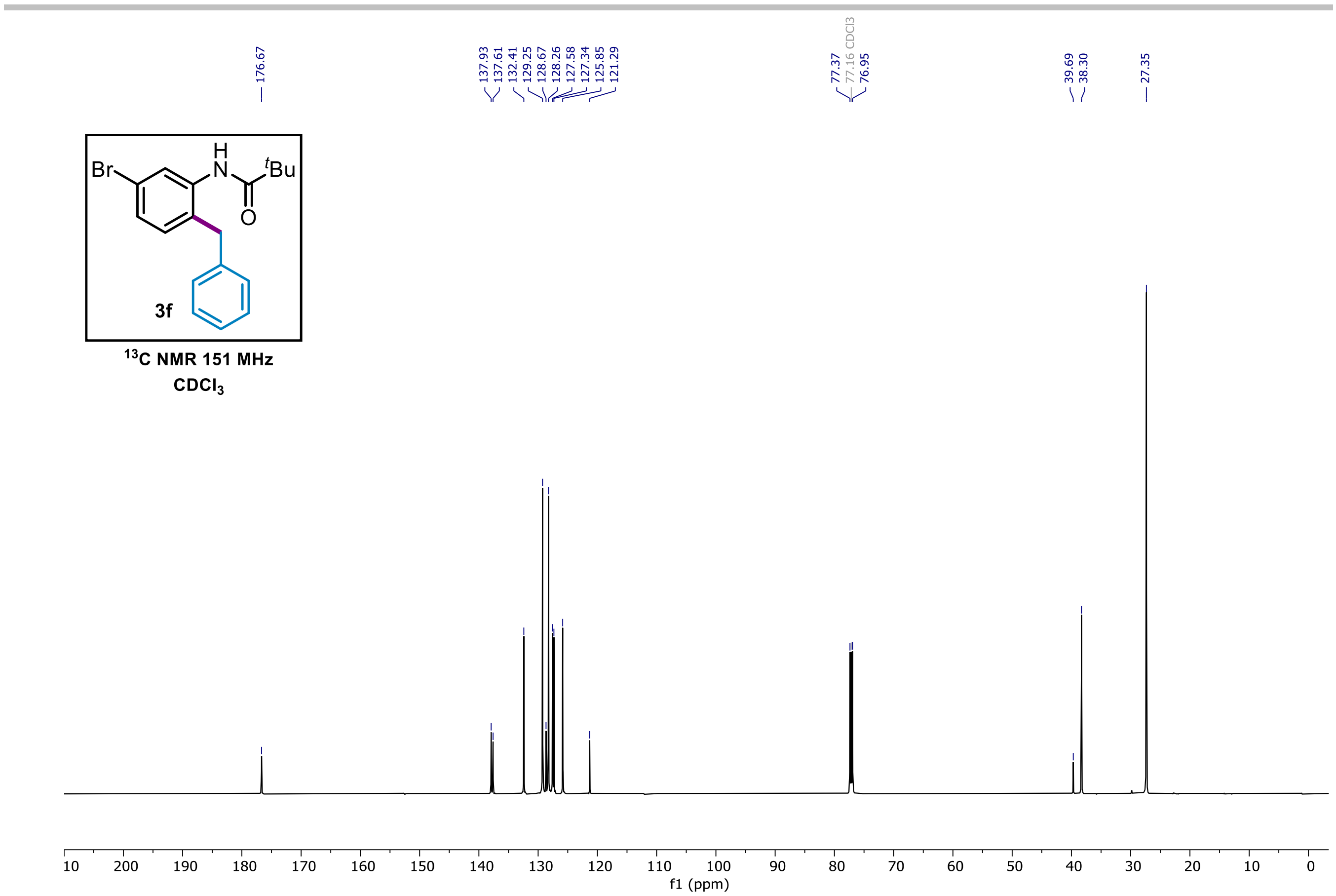
176.67
137.93
137.61
132.41
129.25
128.67
128.26
127.58
127.34
125.85
121.29
77.37
77.16 CDCl3
76.95
39.69
38.30
27.35
Br
H
N
tBu
O
3f
^{13}C NMR 151 MHz
$CDCl_3$
f1 (ppm)

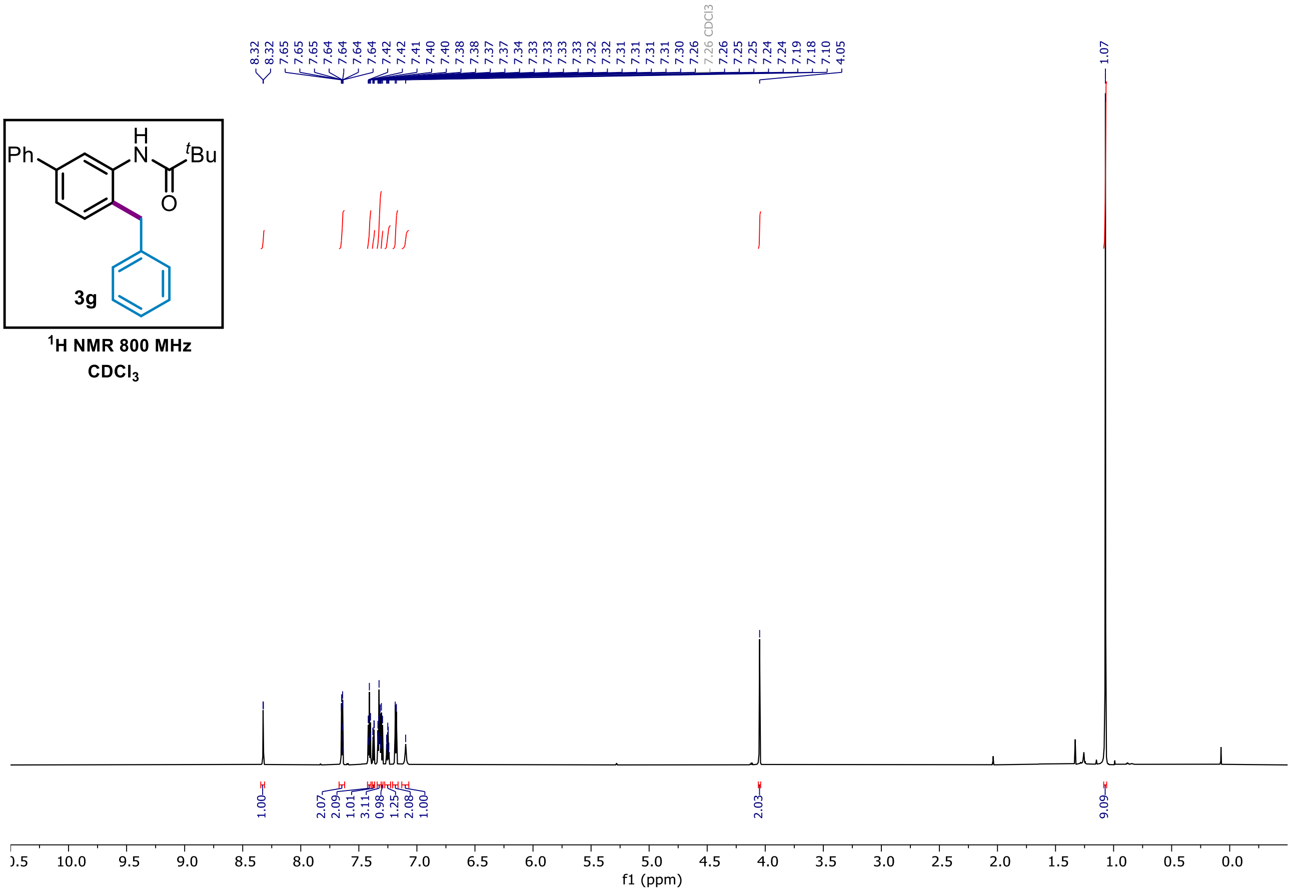
8.32
8.32
7.65
7.65
7.64
7.64
7.64
7.42
7.42
7.41
7.40
7.40
7.38
7.38
7.37
7.37
7.34
7.33
7.33
7.33
7.32
7.32
7.31
7.31
7.31
7.31
7.30
7.26
7.26 CDCl3
7.25
7.25
7.24
7.24
7.19
7.18
7.10
4.05
1.07
Ph
H
N
tBu
O
3g
^{1}H NMR 800 MHz
$CDCl_3$
1.00
2.07
2.09
1.01
3.11
0.98
1.25
2.08
1.00
2.03
9.09
f1 (ppm)

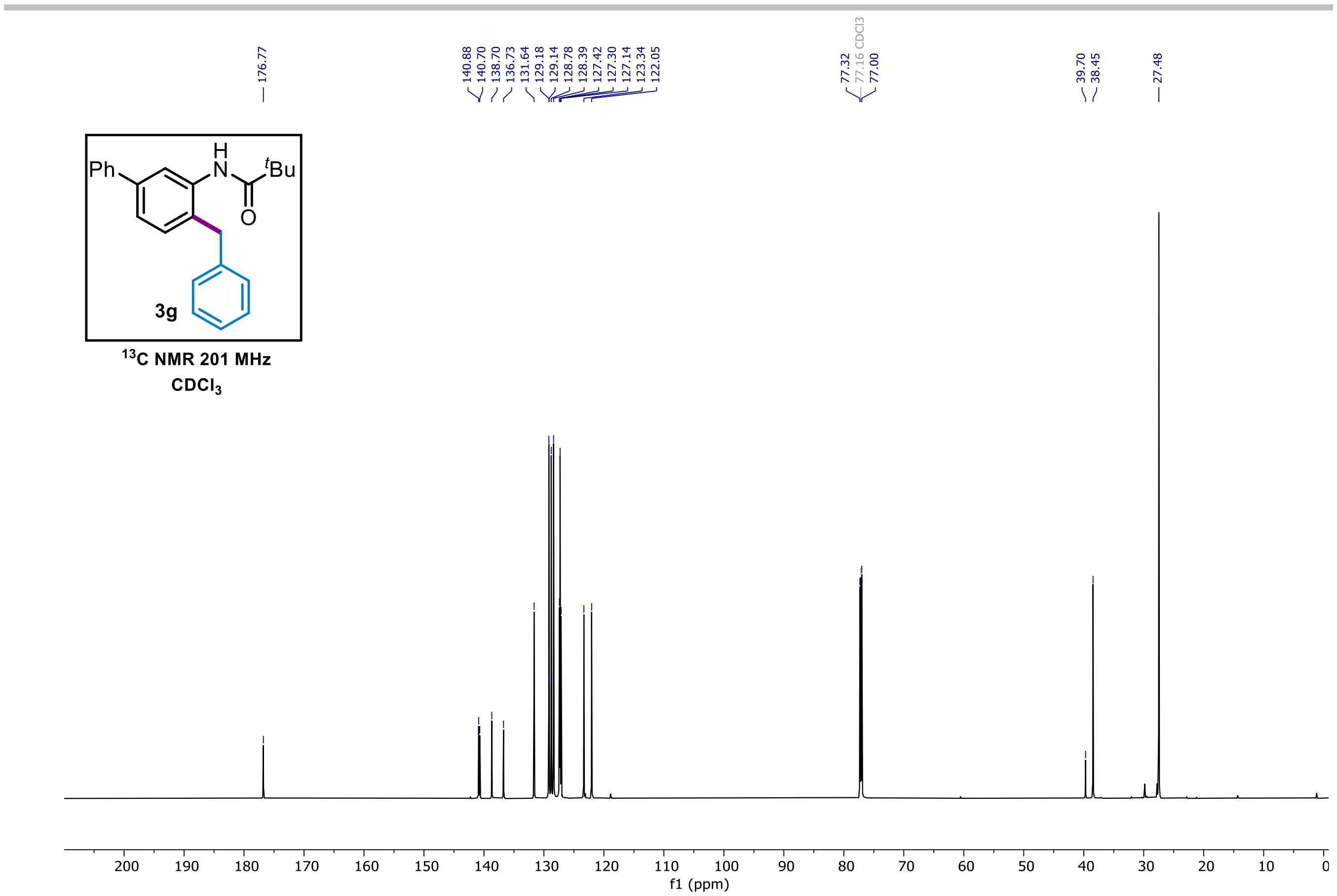

176.77
140.88
140.70
138.70
136.73
131.64
129.18
129.14
128.78
128.39
127.42
127.30
127.14
123.34
122.05
77.32
77.16 CDCl3
77.00
39.70
38.45
27.48
Ph
H
N
tBu
O
3g
13C NMR 201 MHz
CDCl3
f1 (ppm)

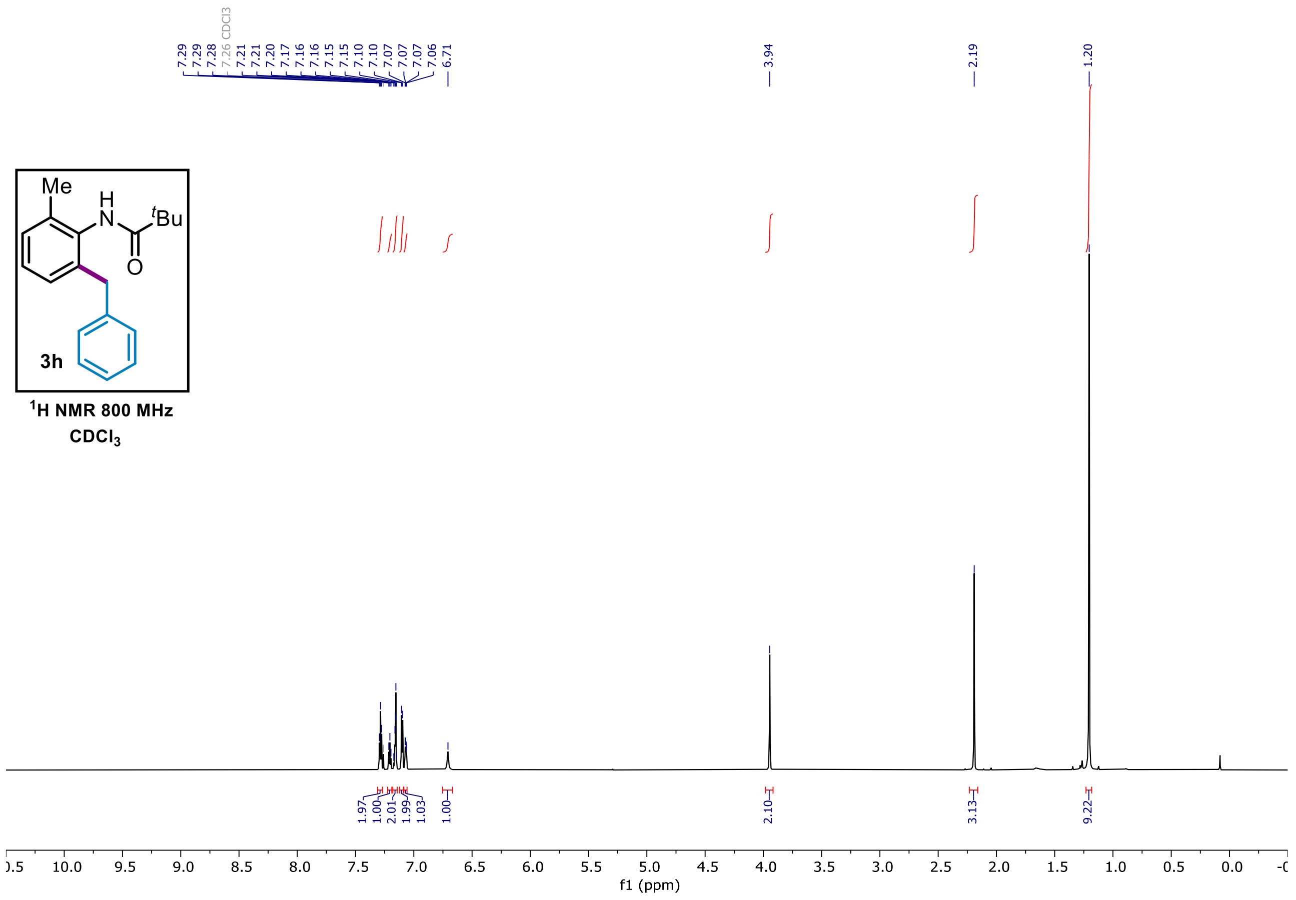

7.29
7.29
7.28
7.26 CDCl3
7.21
7.21
7.20
7.17
7.16
7.16
7.15
7.15
7.10
7.10
7.07
7.07
7.07
7.06
6.71
3.94
2.19
1.20
Me
H
N
tBu
O
3h
1H NMR 800 MHz
CDCl3
1.97
1.00
2.01
1.99
1.03
1.00
2.10
3.13
9.22
f1 (ppm)

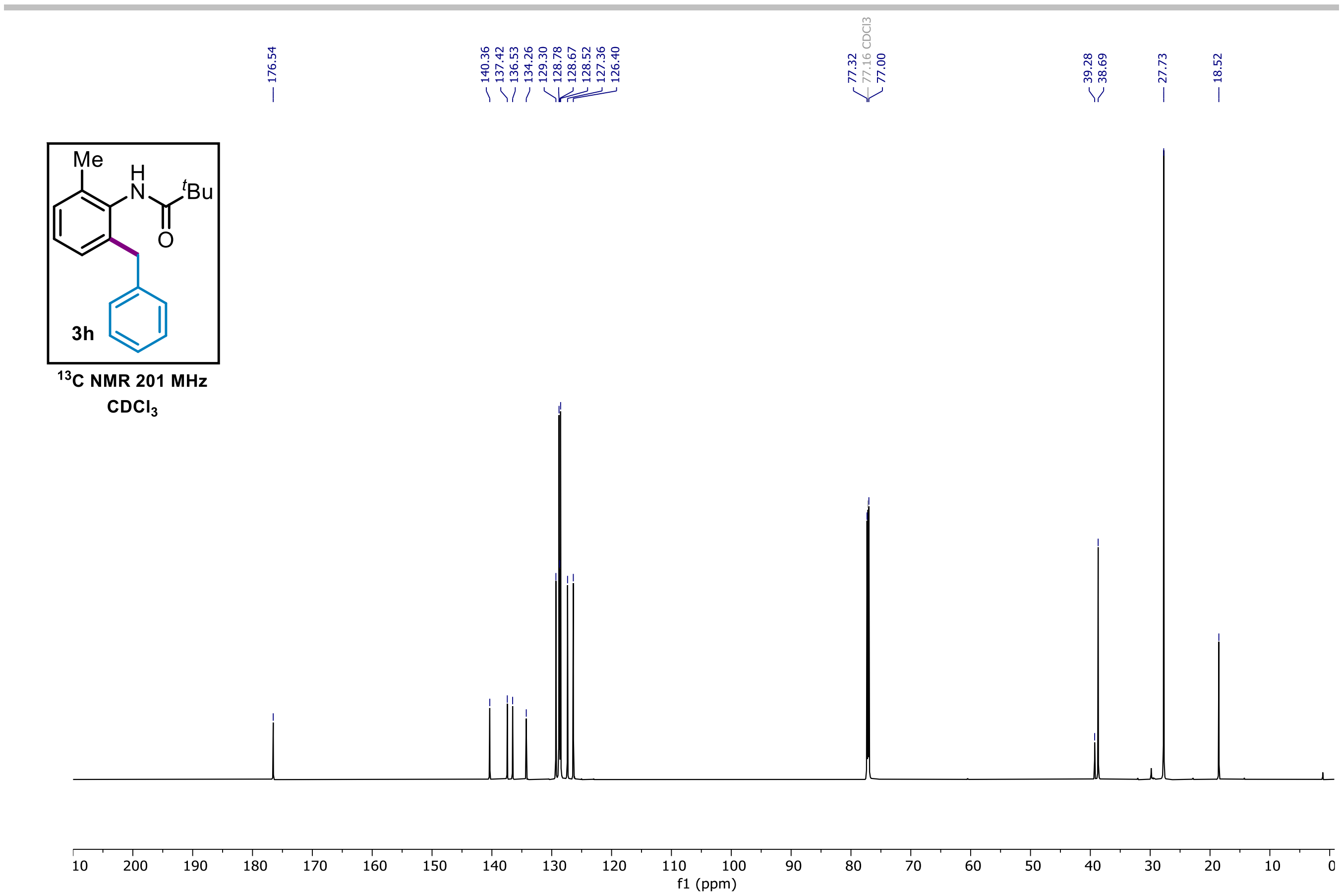

176.54
140.36
137.42
136.53
134.26
129.30
128.78
128.67
128.52
127.36
126.40
77.32
77.16 CDCl3
77.00
39.28
38.69
27.73
18.52
Me
H
N
tBu
O
3h
^{13}C NMR 201 MHz
$CDCl_3$
f1 (ppm)

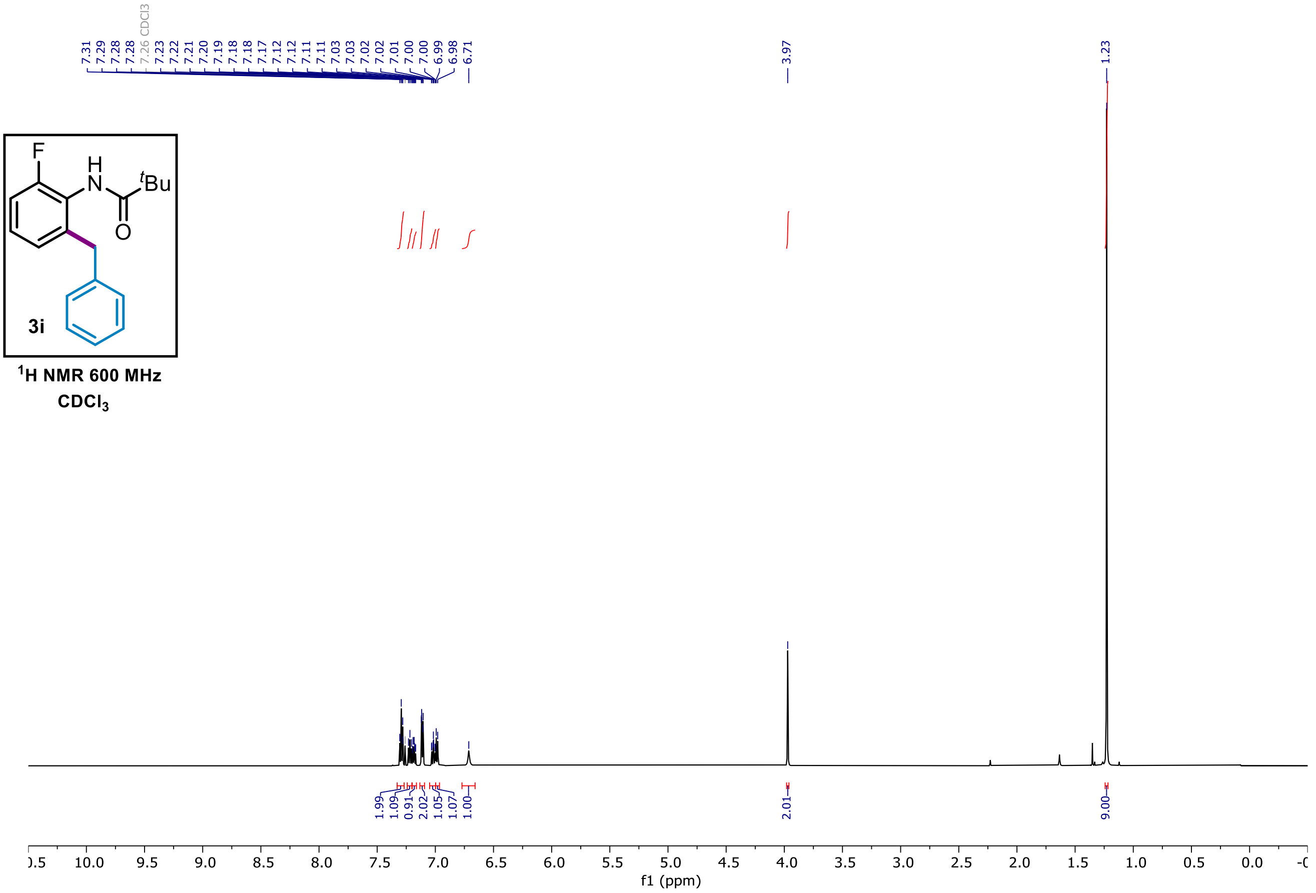

7.31
7.29
7.28
7.28
7.26 CDCl3
7.23
7.22
7.21
7.20
7.19
7.18
7.17
7.12
7.12
7.11
7.03
7.03
7.02
7.01
7.00
7.00
6.99
6.98
6.71
3.97
1.23
F
H
N
tBu
O
3i
^{1}H NMR 600 MHz
$CDCl_3$
1.99
1.09
0.91
2.02
1.05
1.07
1.00
2.01
9.00
f1 (ppm)

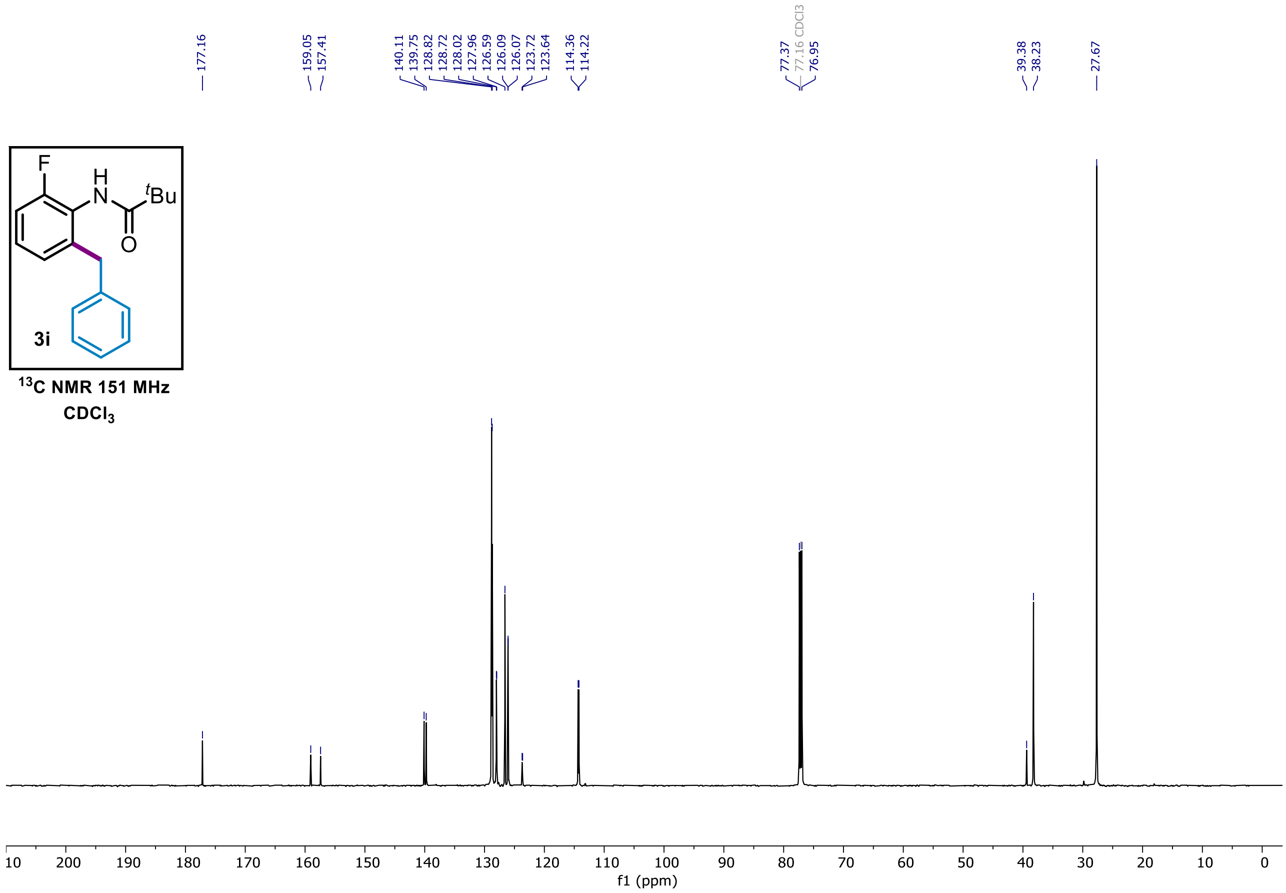
3i
^{13}C NMR 151 MHz
$CDCl_3$
177.16
159.05
157.41
140.11
139.75
128.82
128.72
128.02
127.96
126.59
126.09
126.07
123.72
123.64
114.36
114.22
77.37
77.16 CDCl3
76.95
39.38
38.23
27.67
f1 (ppm)

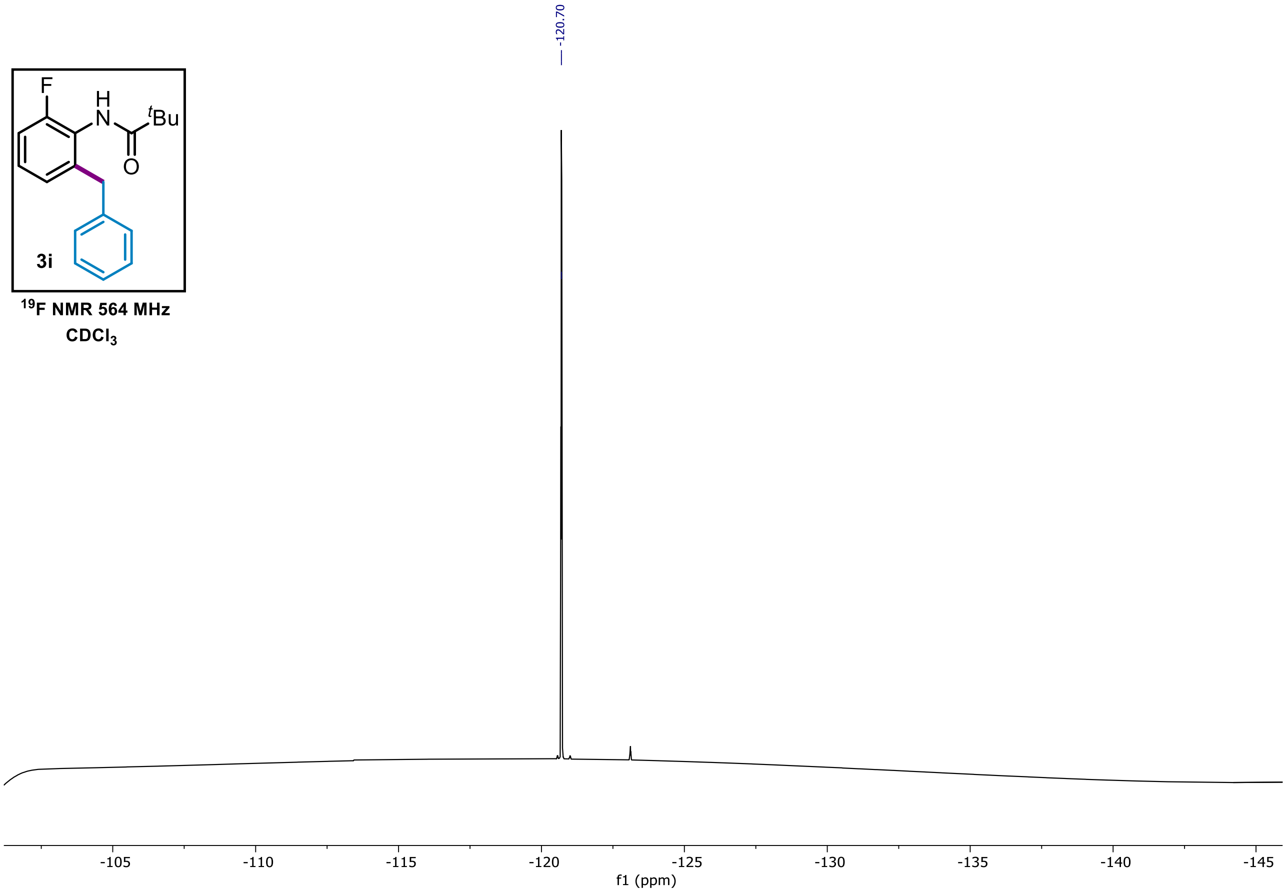
3i
^{19}F NMR 564 MHz
$CDCl_3$
-120.70
f1 (ppm)

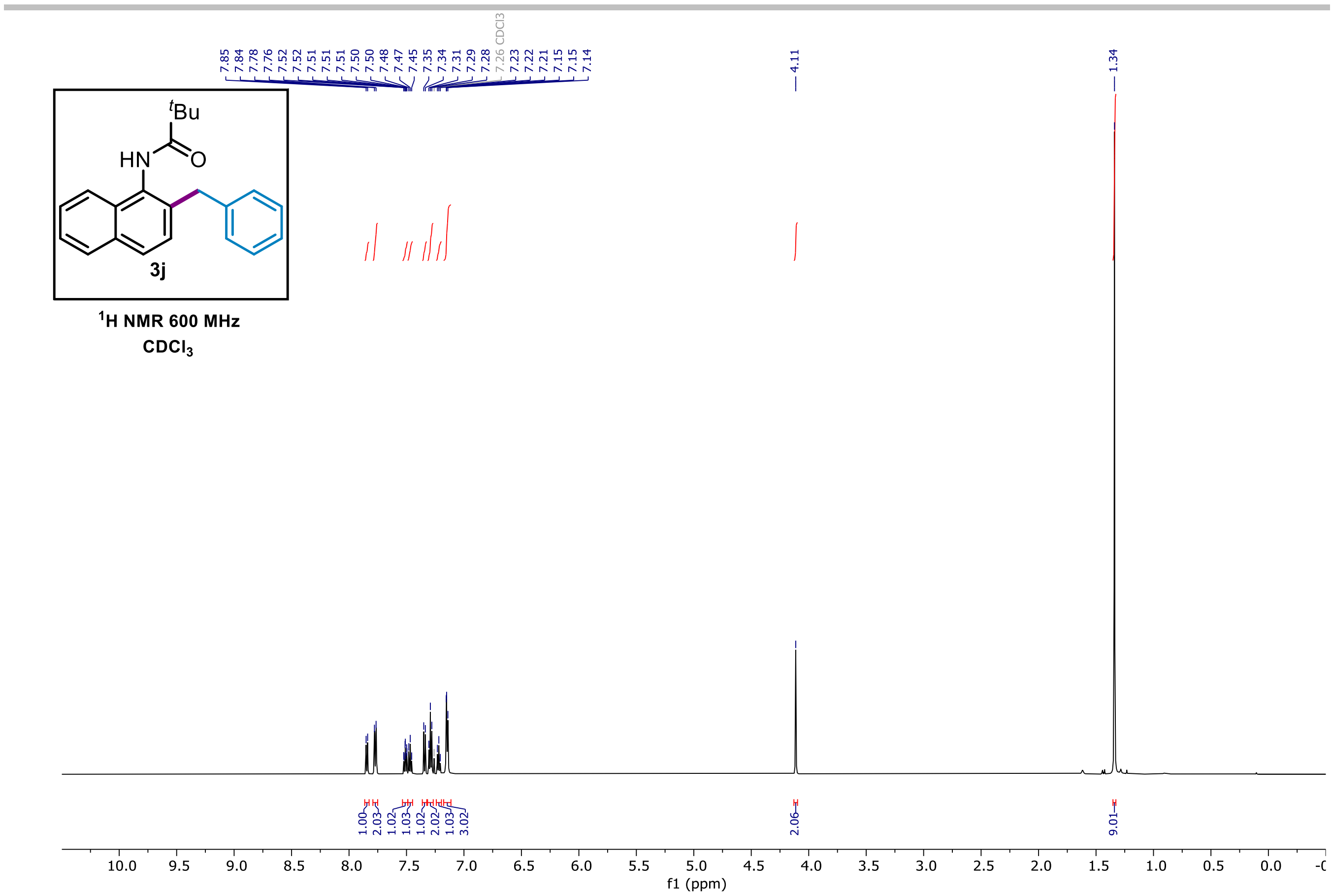

tBu
HN
O
3j
1H NMR 600 MHz
CDCl3
7.85 7.84 7.78 7.76 7.52 7.52 7.51 7.51 7.51 7.50 7.50 7.48 7.47 7.45 7.35 7.34 7.31 7.29 7.28 7.26 CDCl3 7.23 7.22 7.21 7.15 7.15 7.14
4.11
1.34
1.00 2.03 1.02 1.03 1.02 2.02 1.03 3.02
2.06
9.01
10.0 9.5 9.0 8.5 8.0 7.5 7.0 6.5 6.0 5.5 5.0 4.5 4.0 3.5 3.0 2.5 2.0 1.5 1.0 0.5 0.0
f1 (ppm)

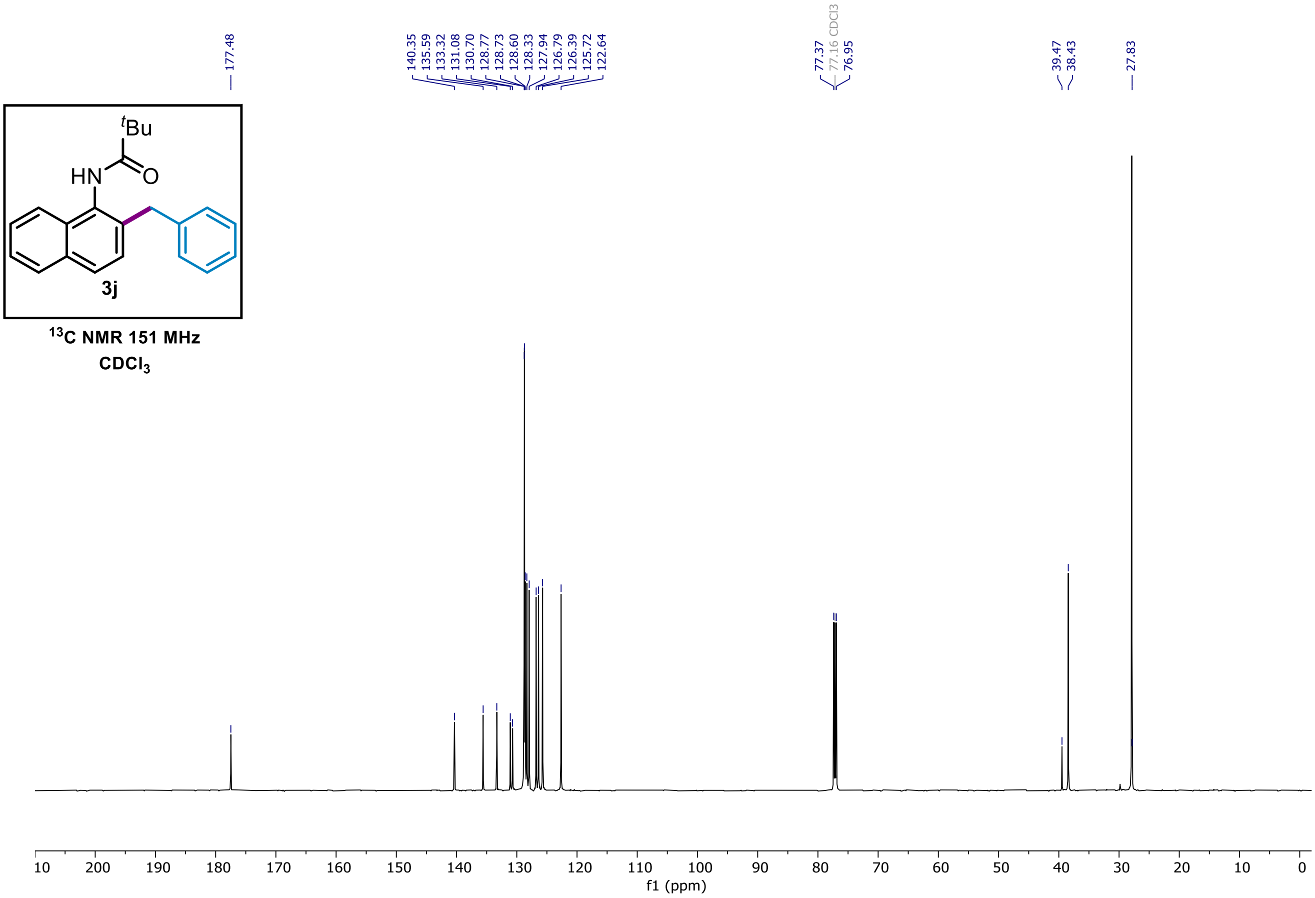

tBu
HN
O
3j
13C NMR 151 MHz
CDCl3
177.48
140.35 135.59 133.32 131.08 130.70 128.77 128.73 128.60 128.33 127.94 126.79 126.39 125.72 122.64
77.37 77.16 CDCl3 76.95
39.47 38.43
27.83
200 190 180 170 160 150 140 130 120 110 100 90 80 70 60 50 40 30 20 10 0
f1 (ppm)

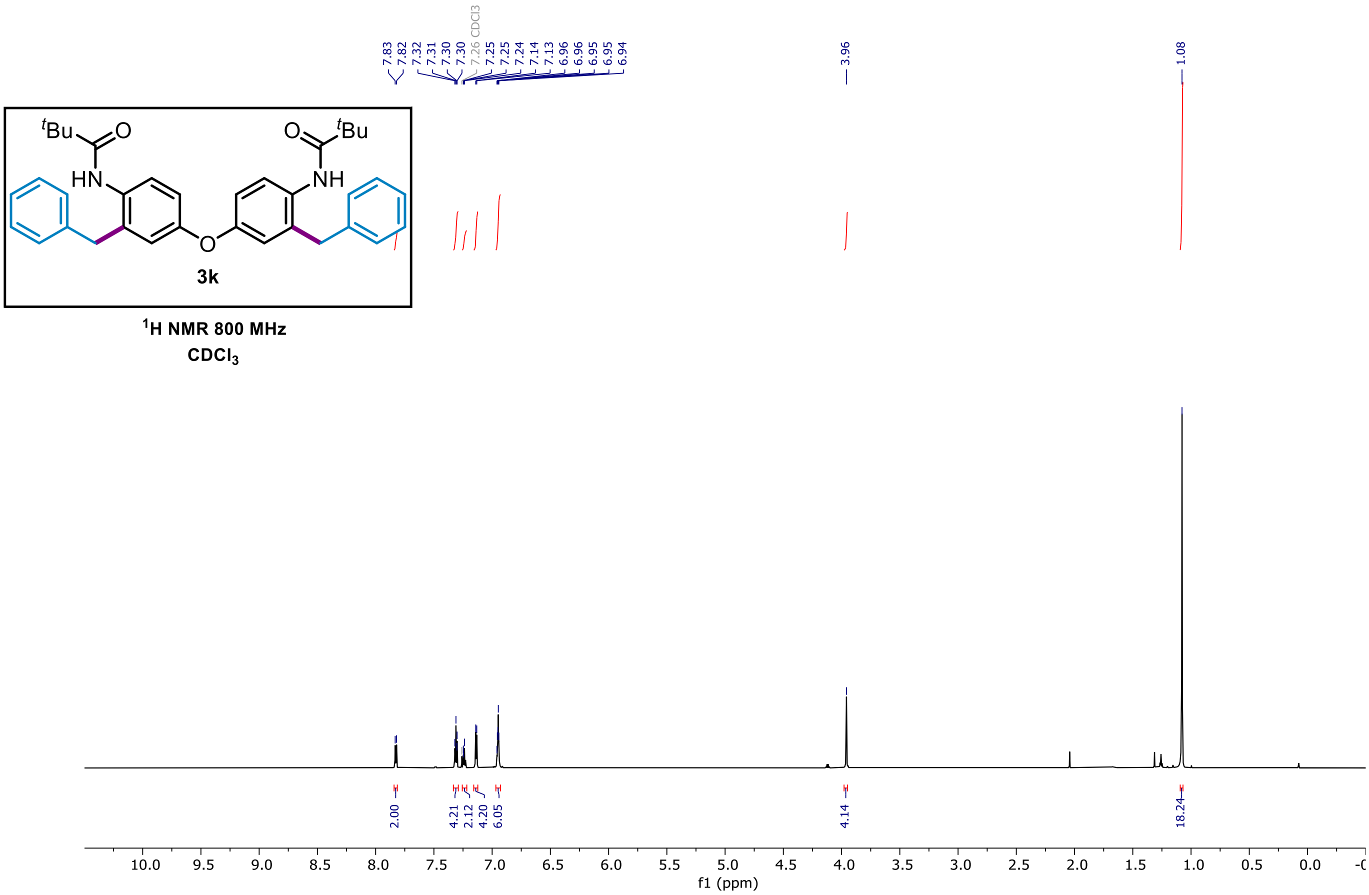

3k
^{1}H NMR 800 MHz
$CDCl_3$
f1 (ppm)

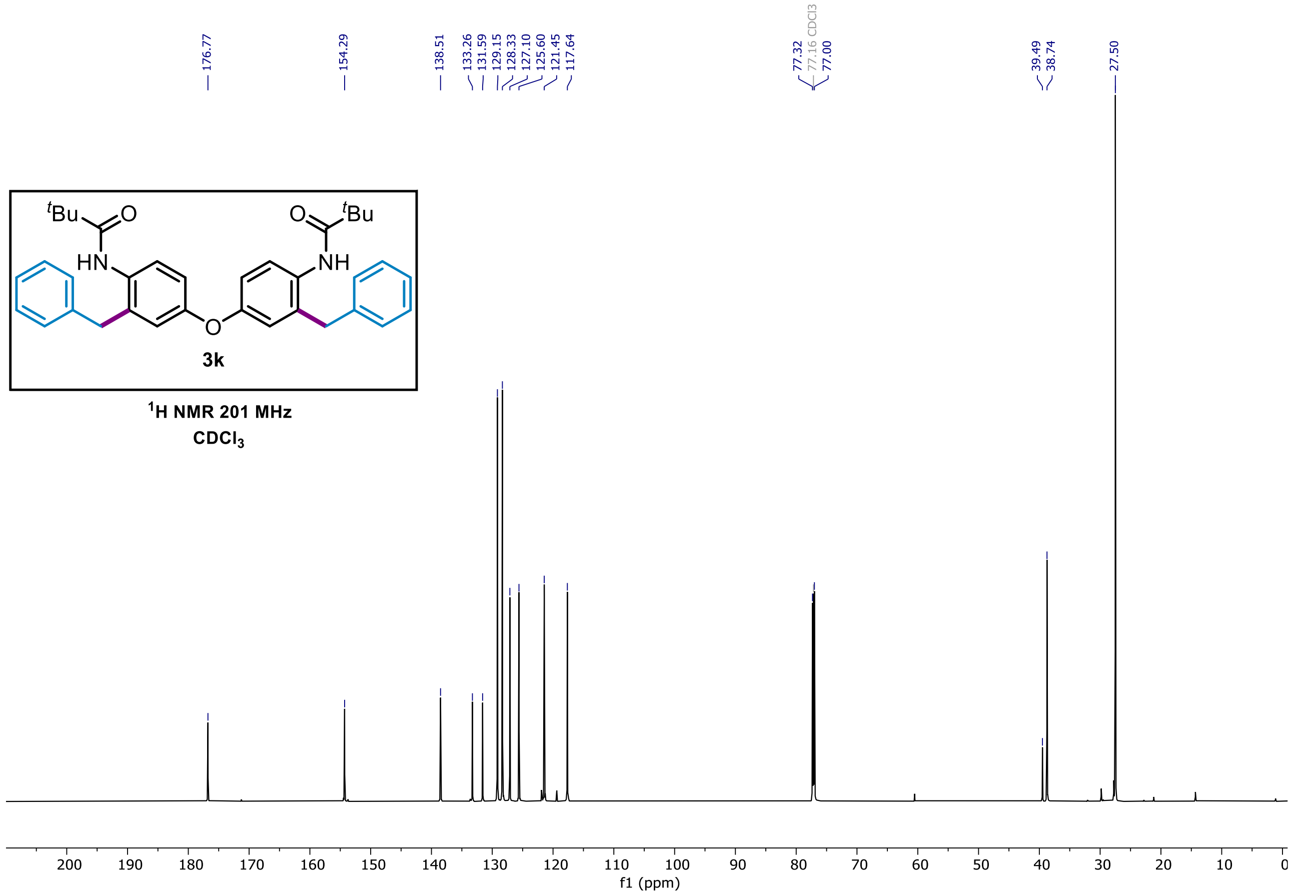

3k
^{1}H NMR 201 MHz
$CDCl_3$
f1 (ppm)

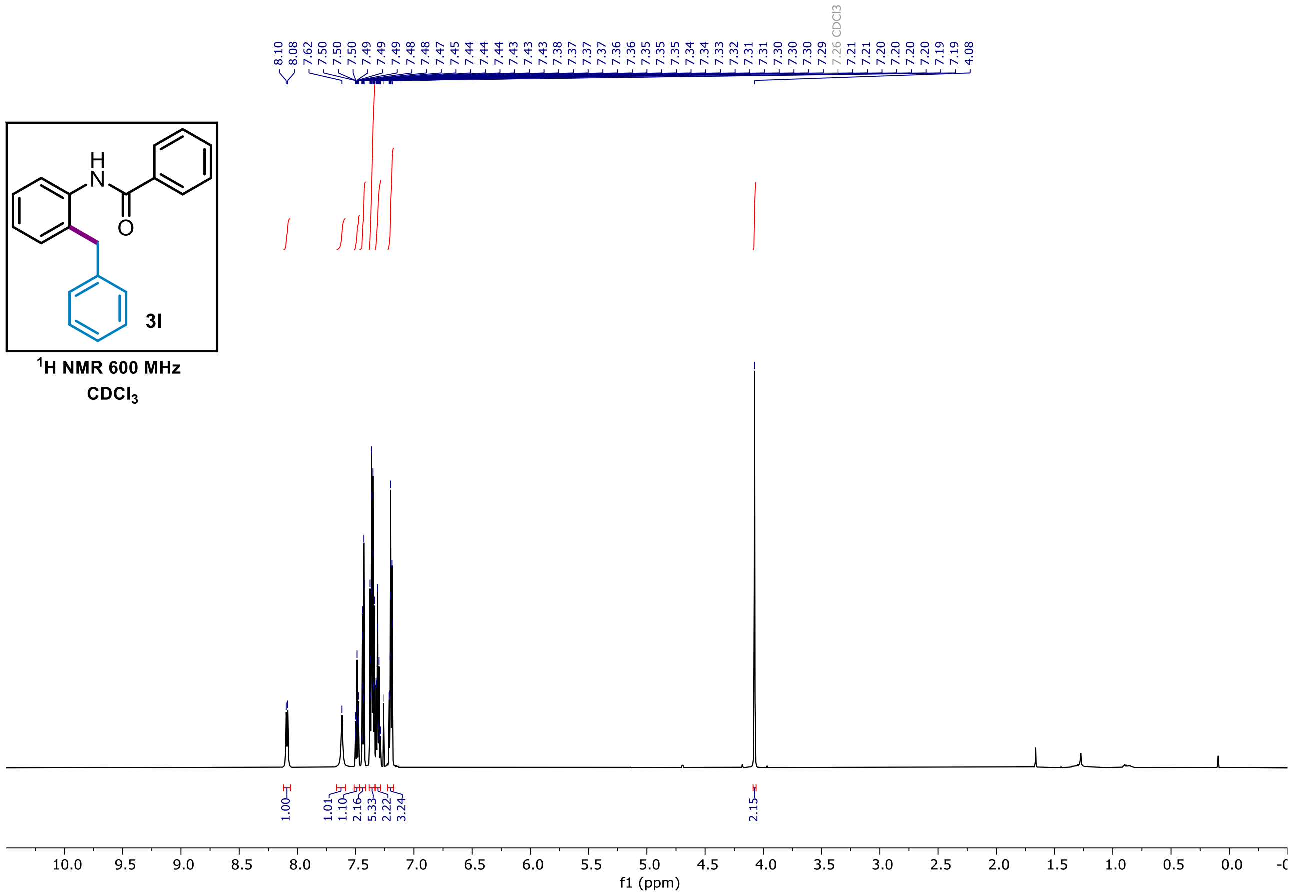

^{1}H NMR 600 MHz

$CDCl_3$

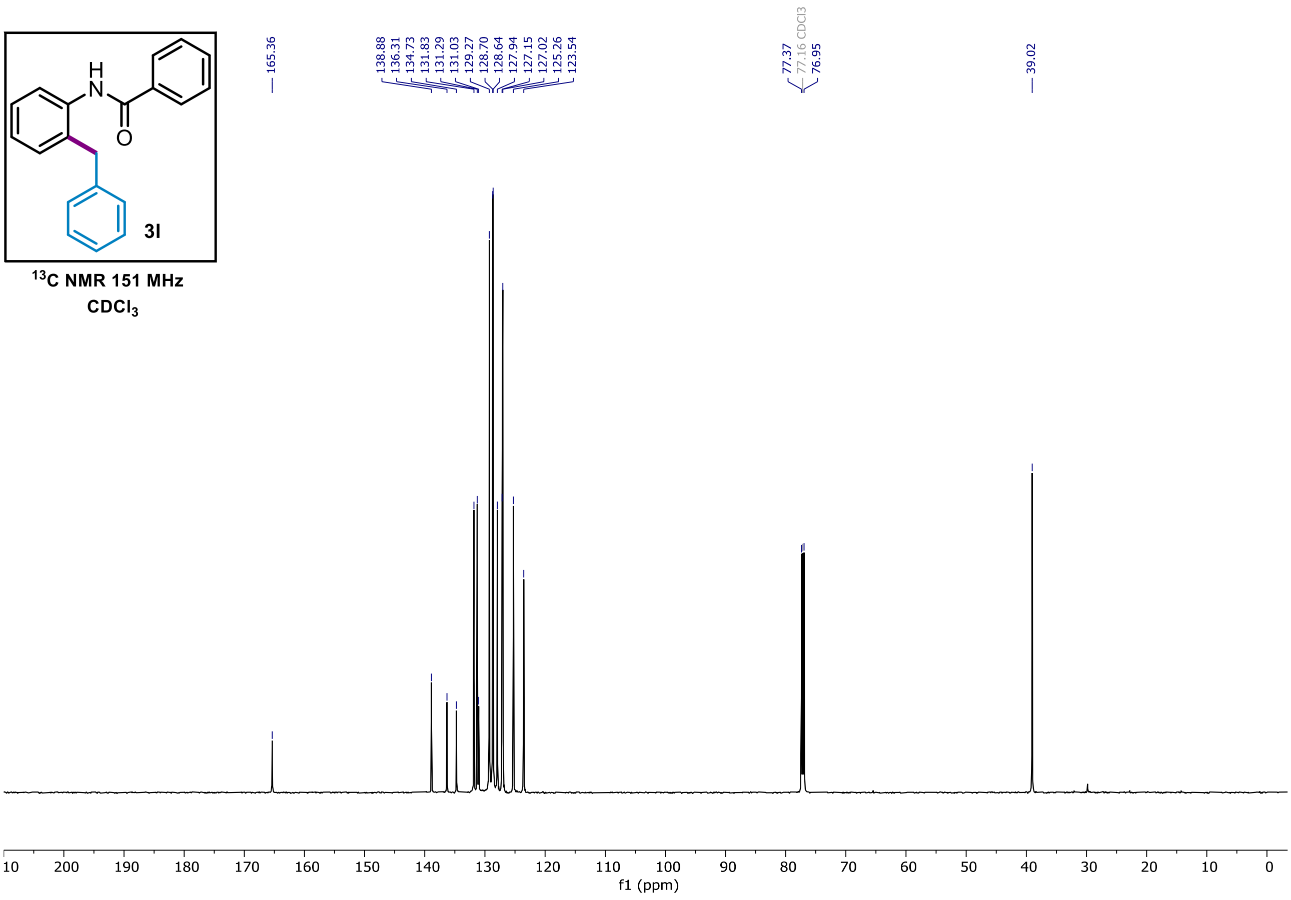

^{13}C NMR 151 MHz

$CDCl_3$

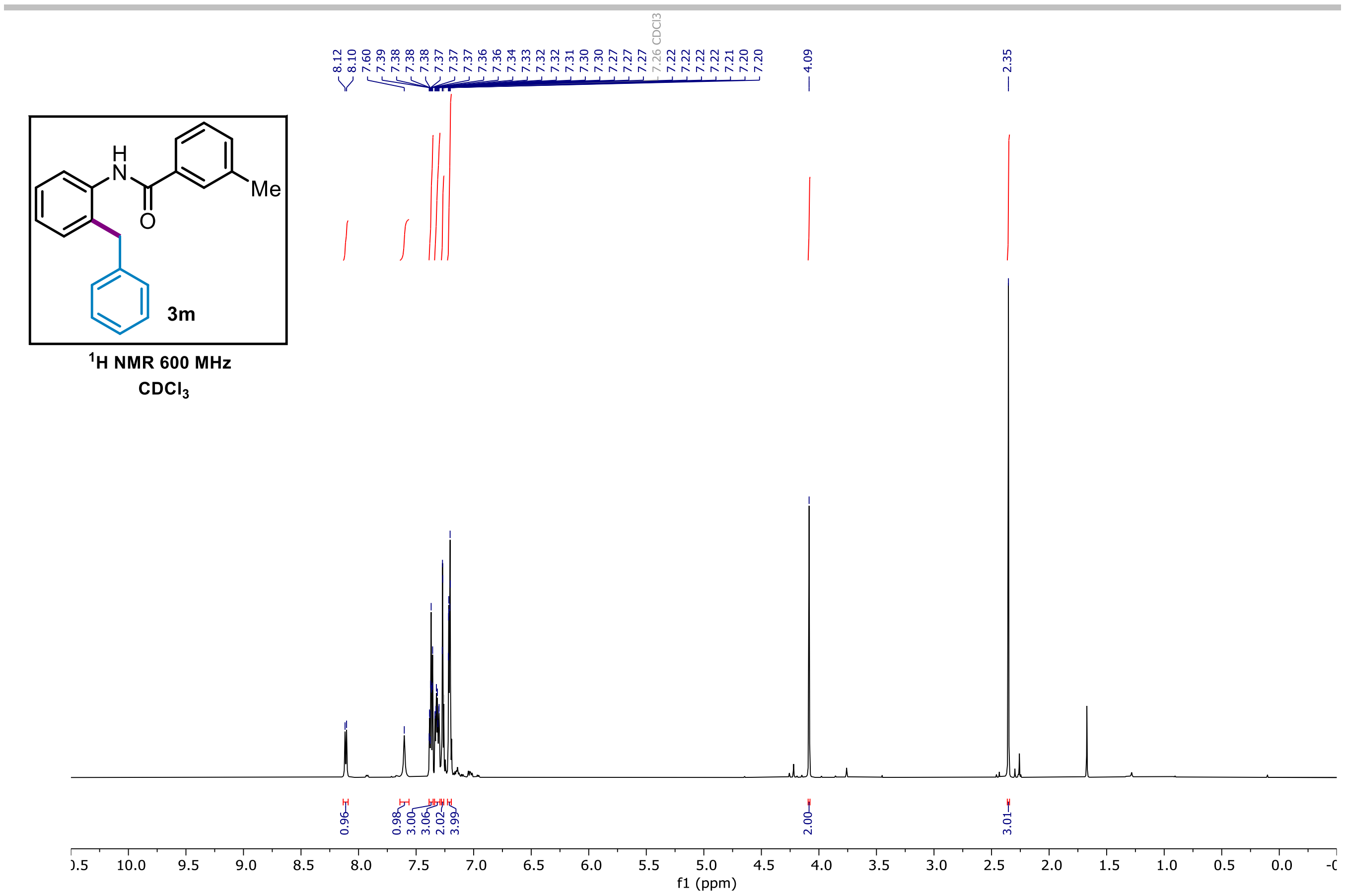
3m
1H NMR 600 MHz
CDCl3
f1 (ppm)

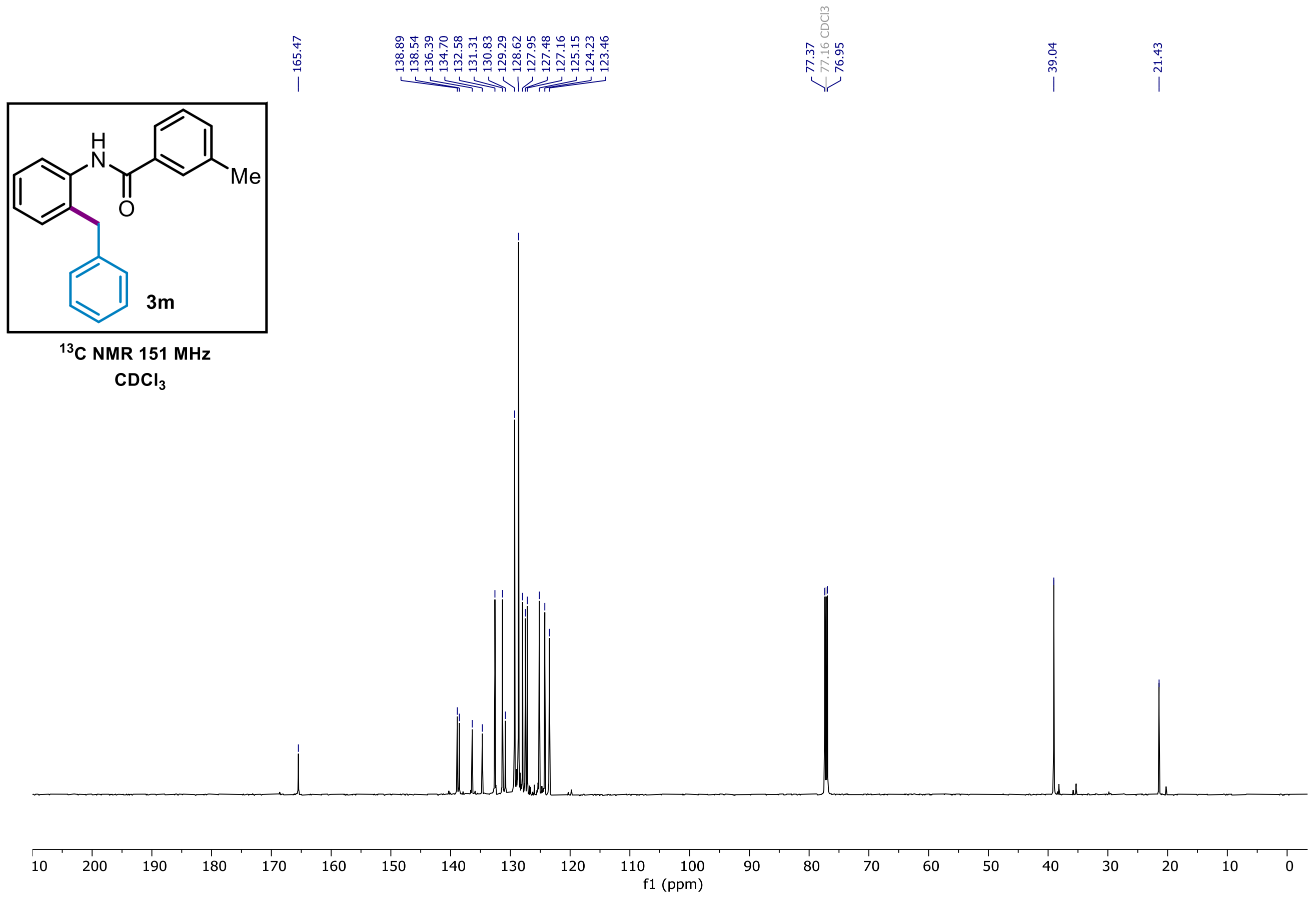
3m
13C NMR 151 MHz
CDCl3
f1 (ppm)

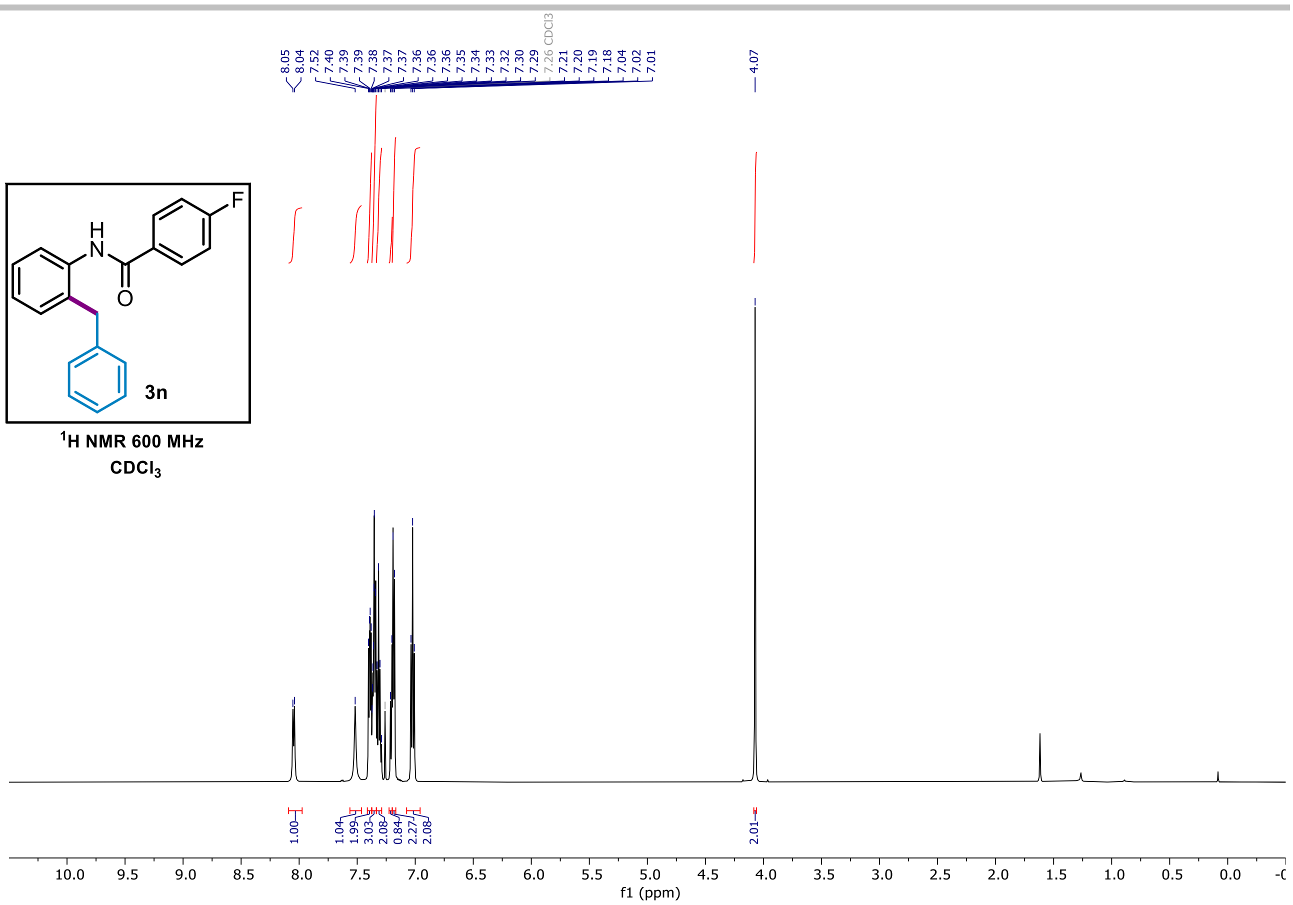
3n
¹H NMR 600 MHz
CDCl3
8.05
8.04
7.52
7.40
7.39
7.39
7.38
7.37
7.37
7.36
7.36
7.35
7.34
7.33
7.32
7.30
7.29
7.26 CDCl3
7.21
7.20
7.19
7.18
7.04
7.02
7.01
4.07
1.00
1.04
1.99
3.03
2.08
0.84
2.27
2.08
2.01
f1 (ppm)

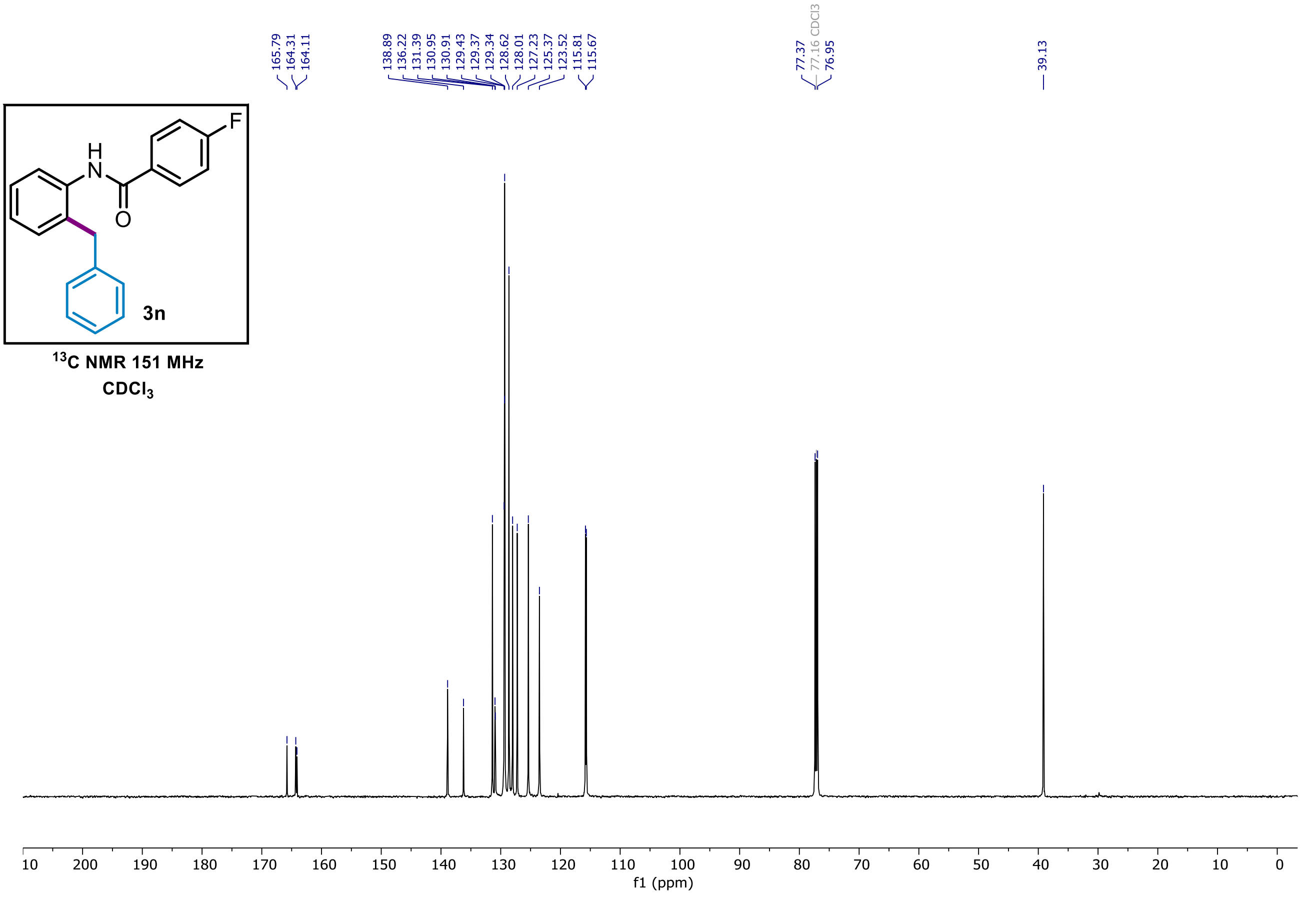
3n
¹³C NMR 151 MHz
CDCl3
165.79
164.31
164.11
138.89
136.22
131.39
130.95
130.91
129.43
129.37
129.34
128.62
128.01
127.23
125.37
123.52
115.81
115.67
77.37
77.16 CDCl3
76.95
39.13
f1 (ppm)

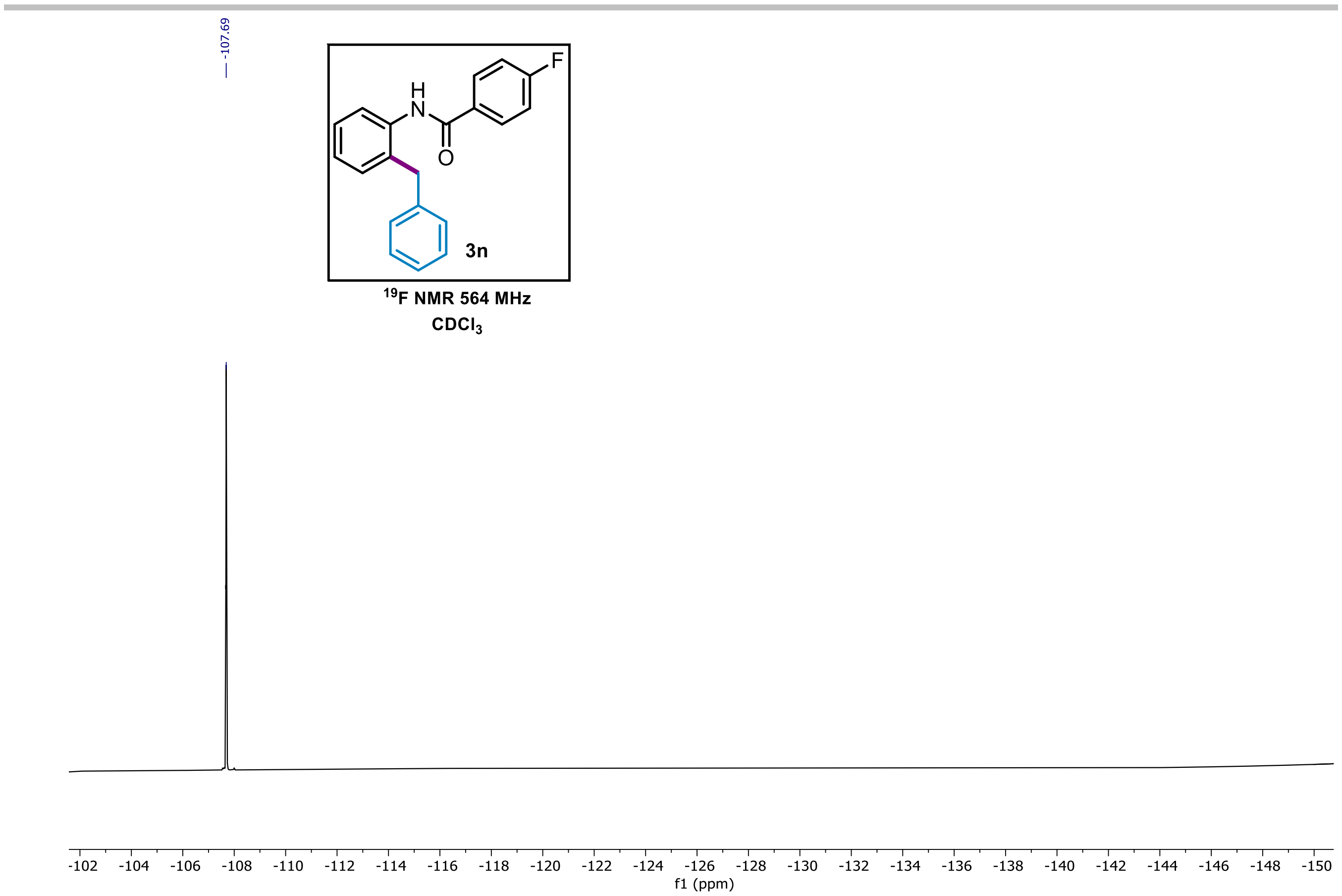
-107.69
F
H
N
O
3n
19F NMR 564 MHz
CDCl3
-102 -104 -106 -108 -110 -112 -114 -116 -118 -120 -122 -124 -126 -128 -130 -132 -134 -136 -138 -140 -142 -144 -146 -148 -150
f1 (ppm)

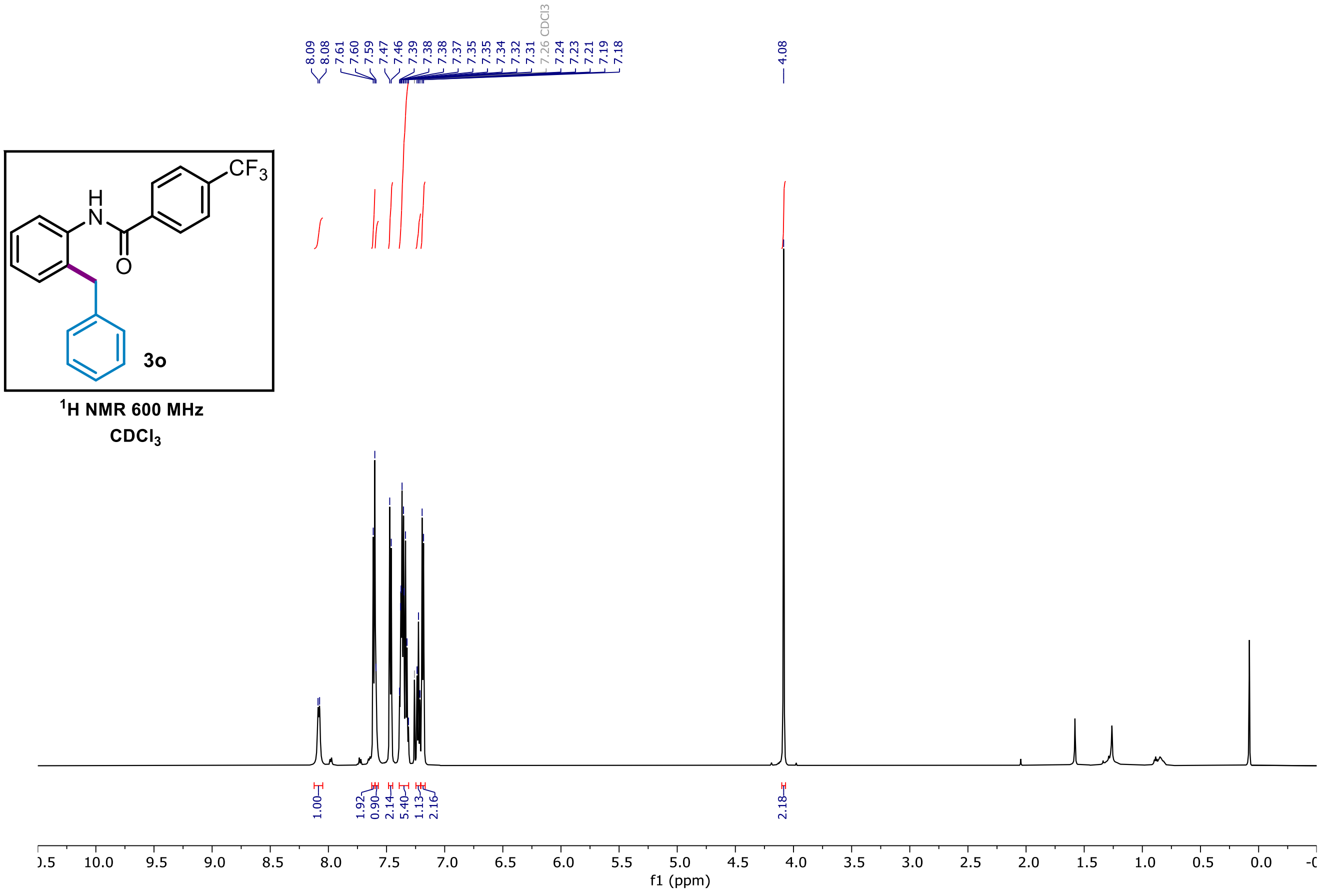
8.09 8.08 7.61 7.60 7.59 7.47 7.46 7.39 7.38 7.38 7.37 7.35 7.35 7.34 7.32 7.31 7.26 CDCl3 7.24 7.23 7.21 7.19 7.18
4.08
CF3
H
N
O
3o
1H NMR 600 MHz
CDCl3
1.00
1.92 0.90 2.14 5.40 1.13 2.16
2.18
10.0 9.5 9.0 8.5 8.0 7.5 7.0 6.5 6.0 5.5 5.0 4.5 4.0 3.5 3.0 2.5 2.0 1.5 1.0 0.5 0.0
f1 (ppm)

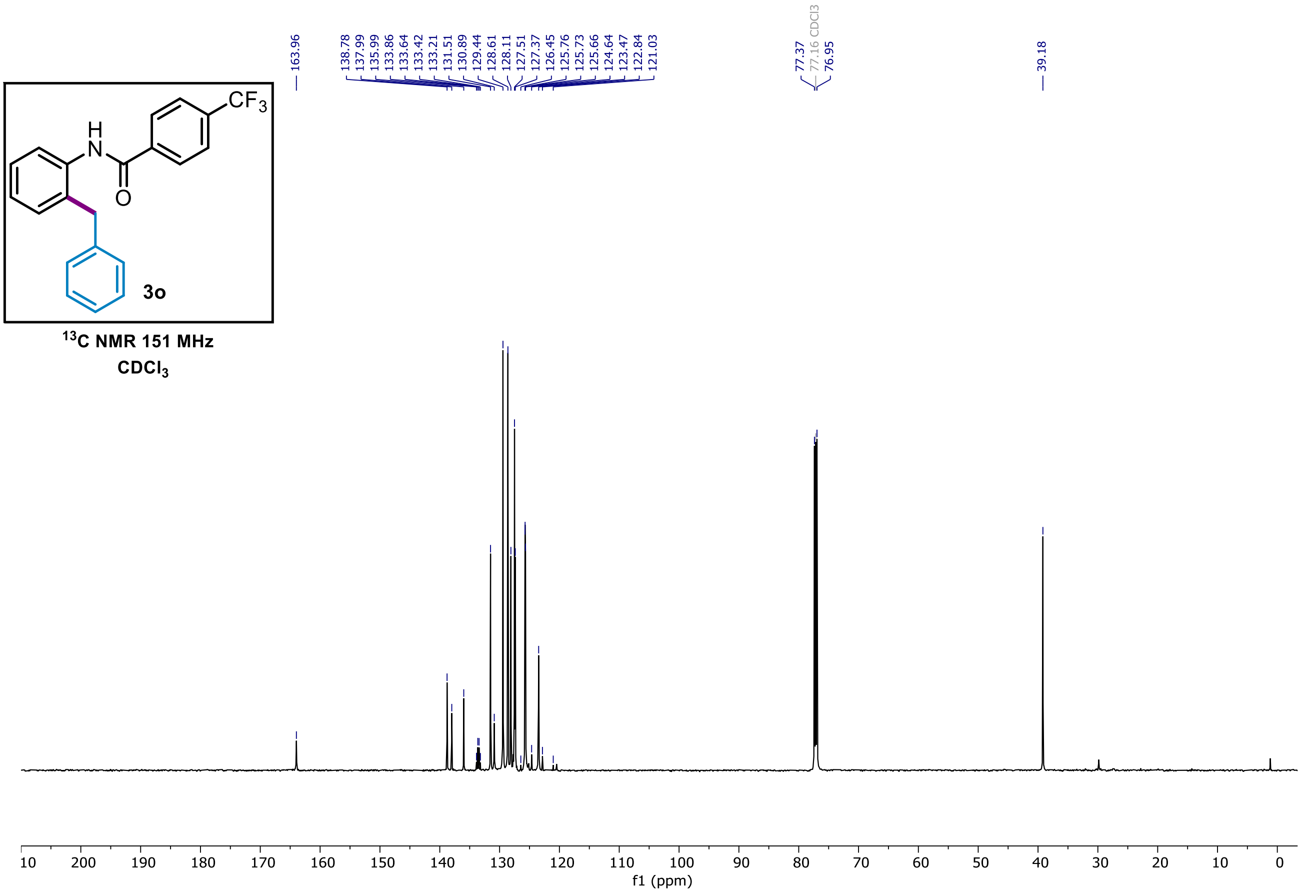

163.96
138.78
137.99
135.99
133.86
133.64
133.42
133.21
131.51
130.89
129.44
128.61
128.11
127.51
127.37
126.45
125.76
125.73
125.66
124.64
123.47
122.84
121.03
77.37
77.16 CDCl3
76.95
39.18
CF3
H
N
O
3o
13C NMR 151 MHz
CDCl3
f1 (ppm)

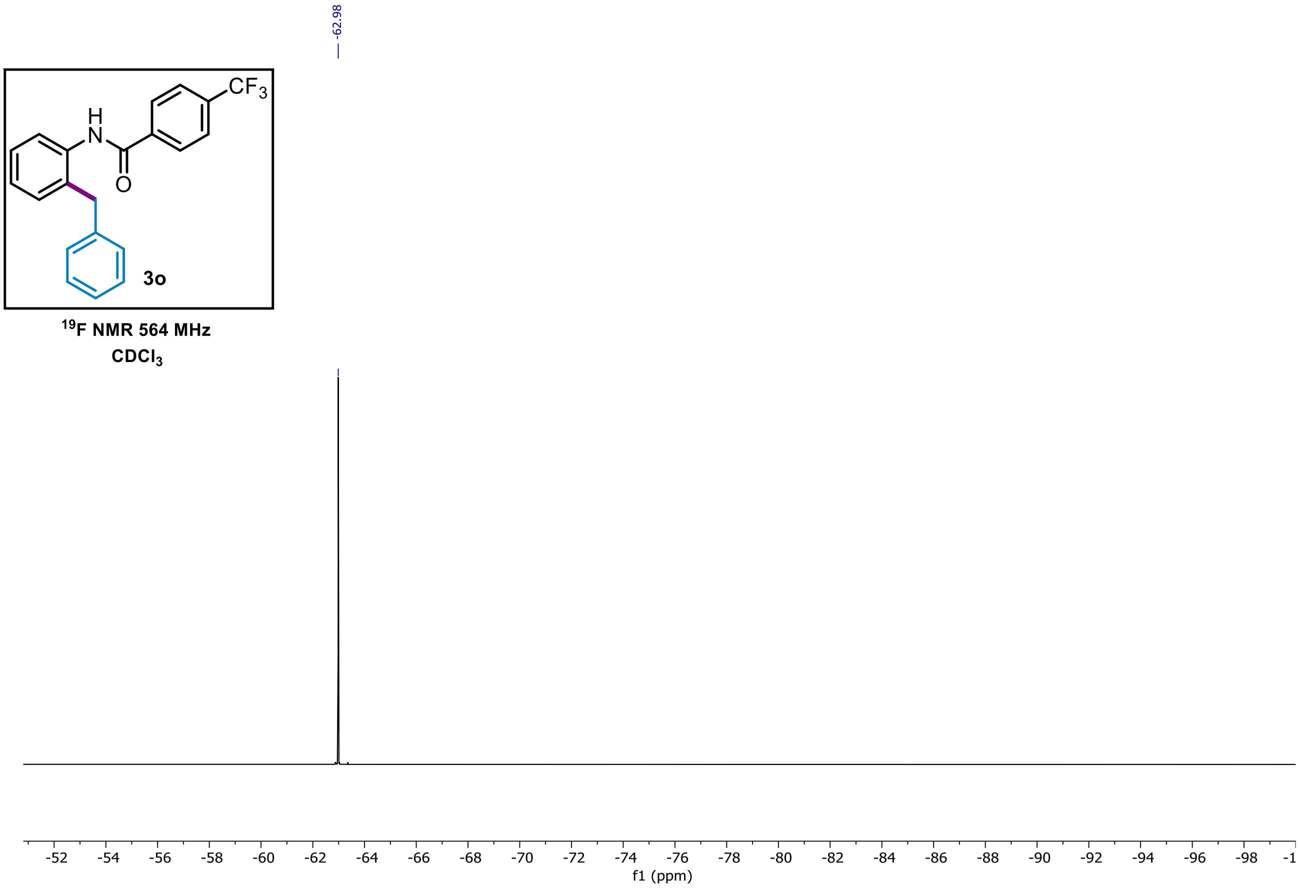

-62.98
CF3
H
N
O
3o
19F NMR 564 MHz
CDCl3
f1 (ppm)

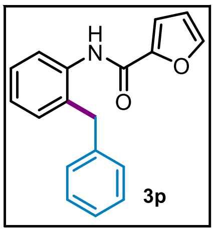

^{1}H NMR 600 MHz
$CDCl_3$

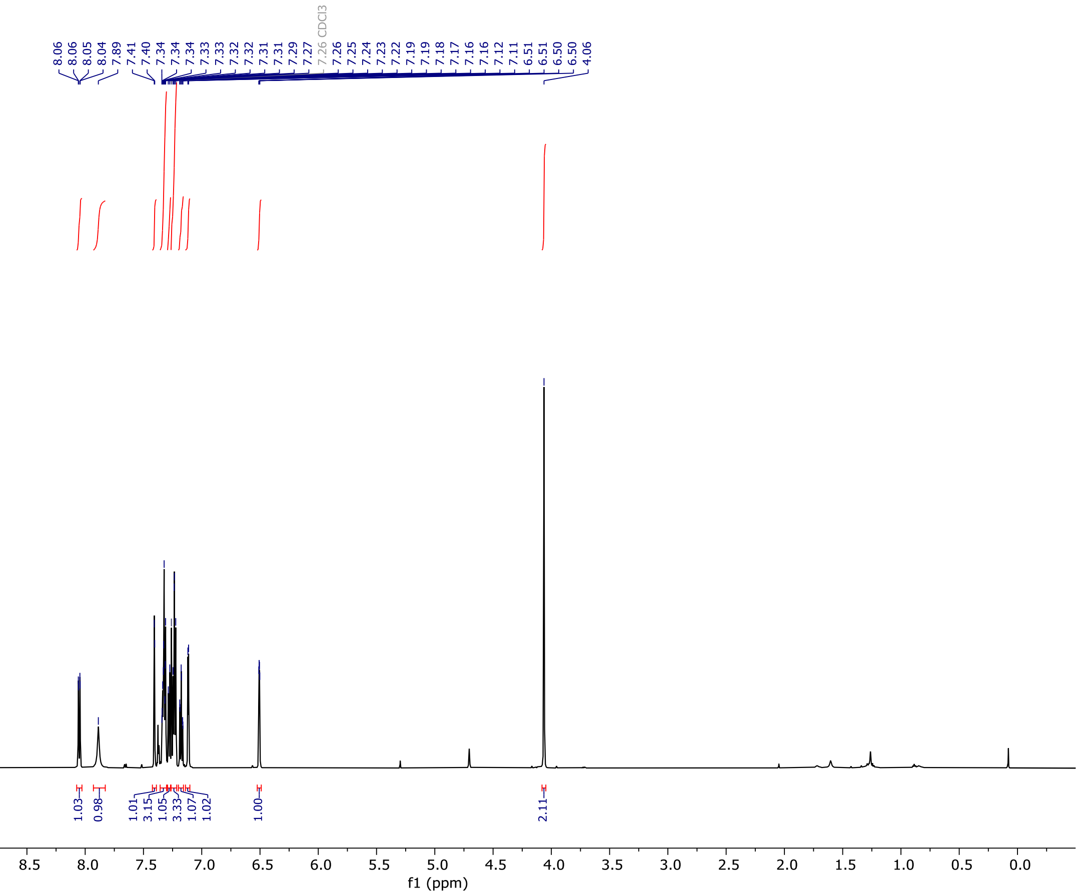

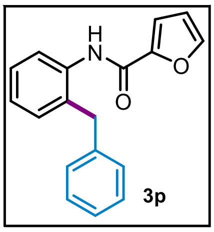

^{13}C NMR 151 MHz
$CDCl_3$

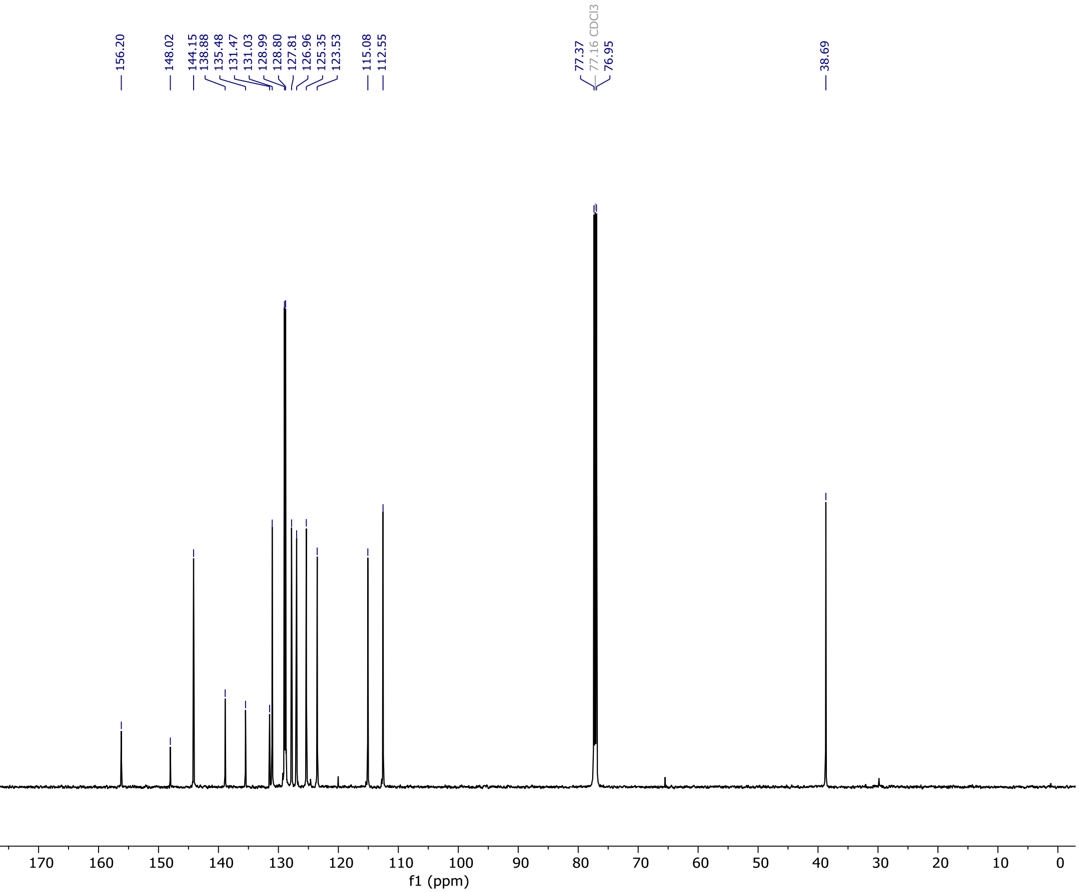

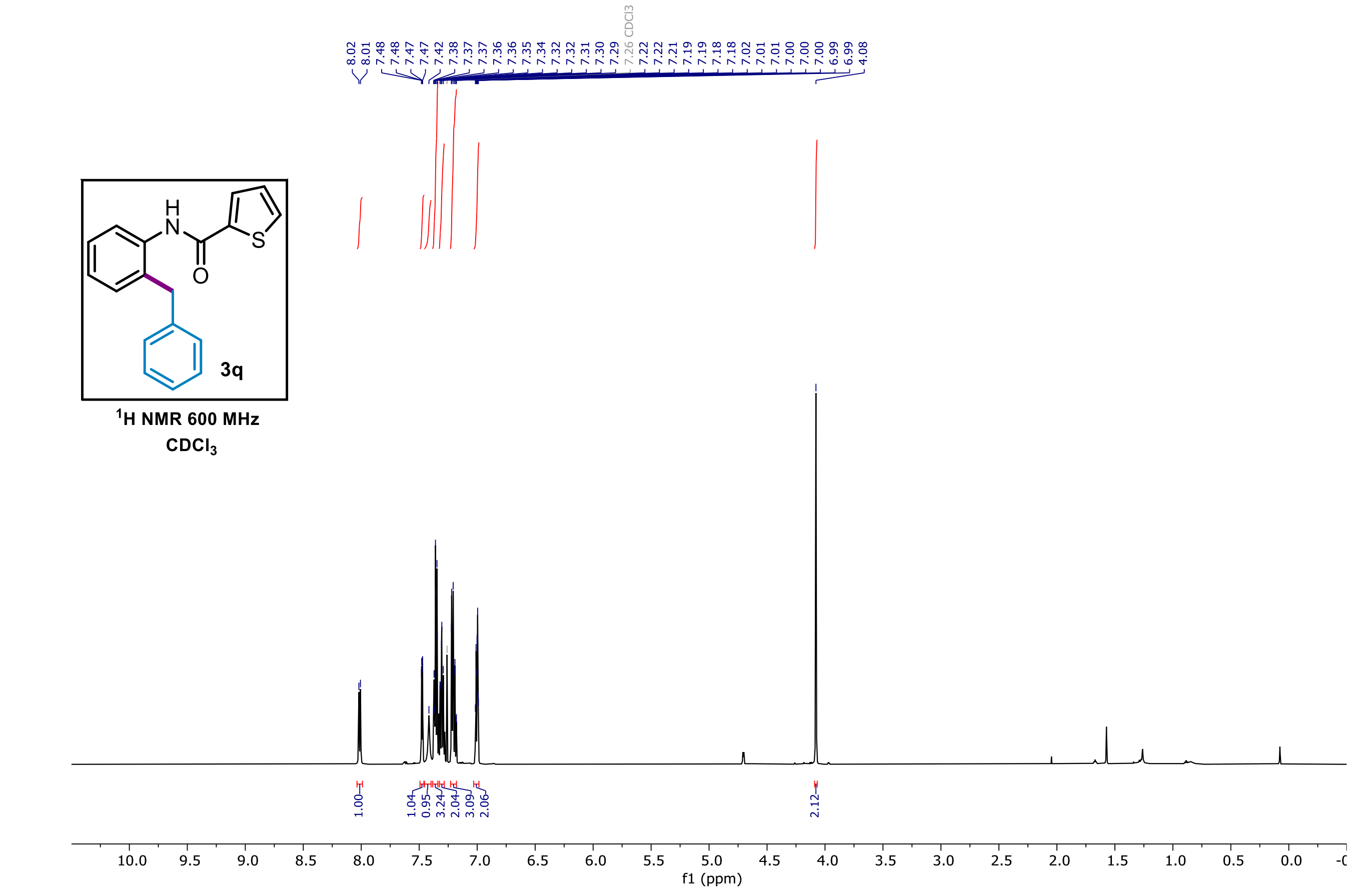

^{1}H NMR 600 MHz
$CDCl_3$

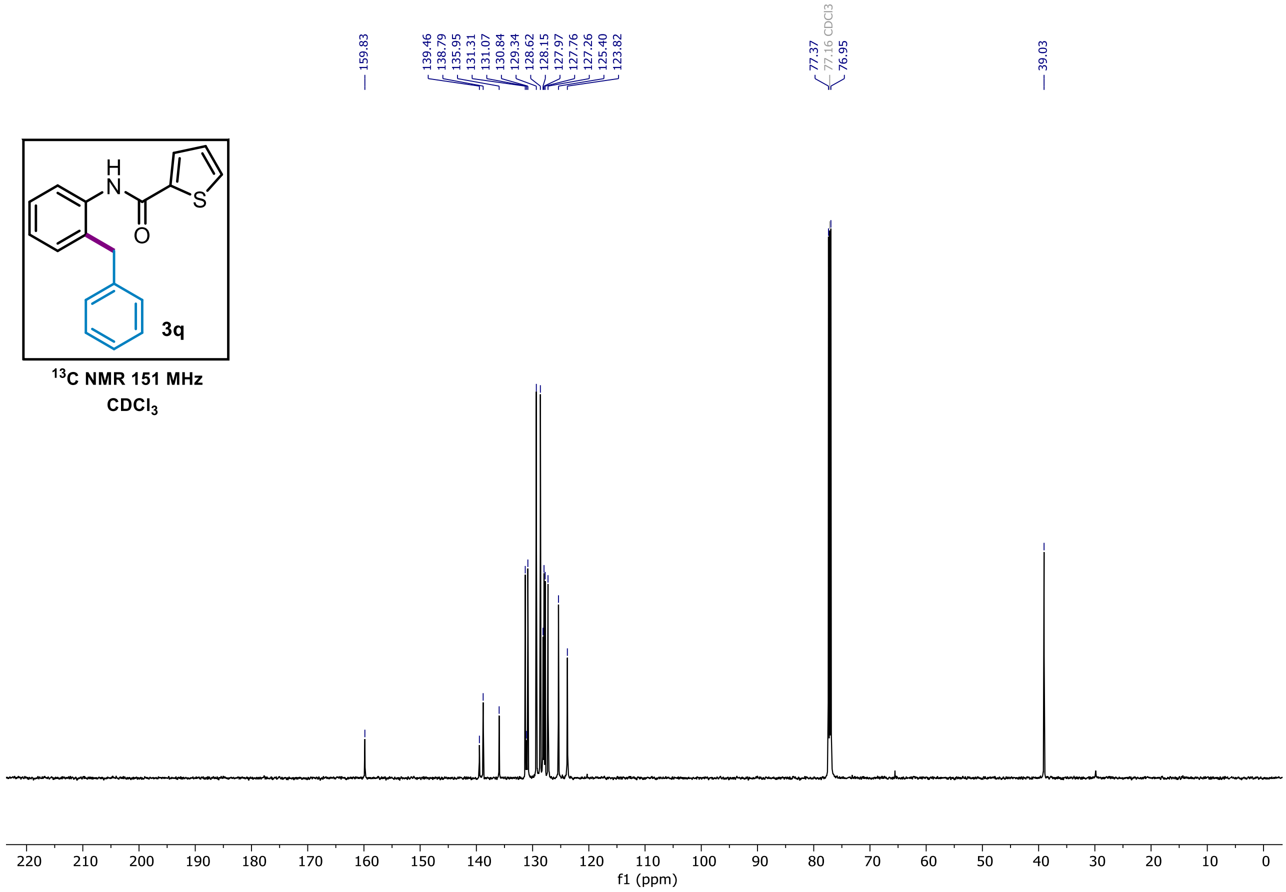

^{13}C NMR 151 MHz
$CDCl_3$

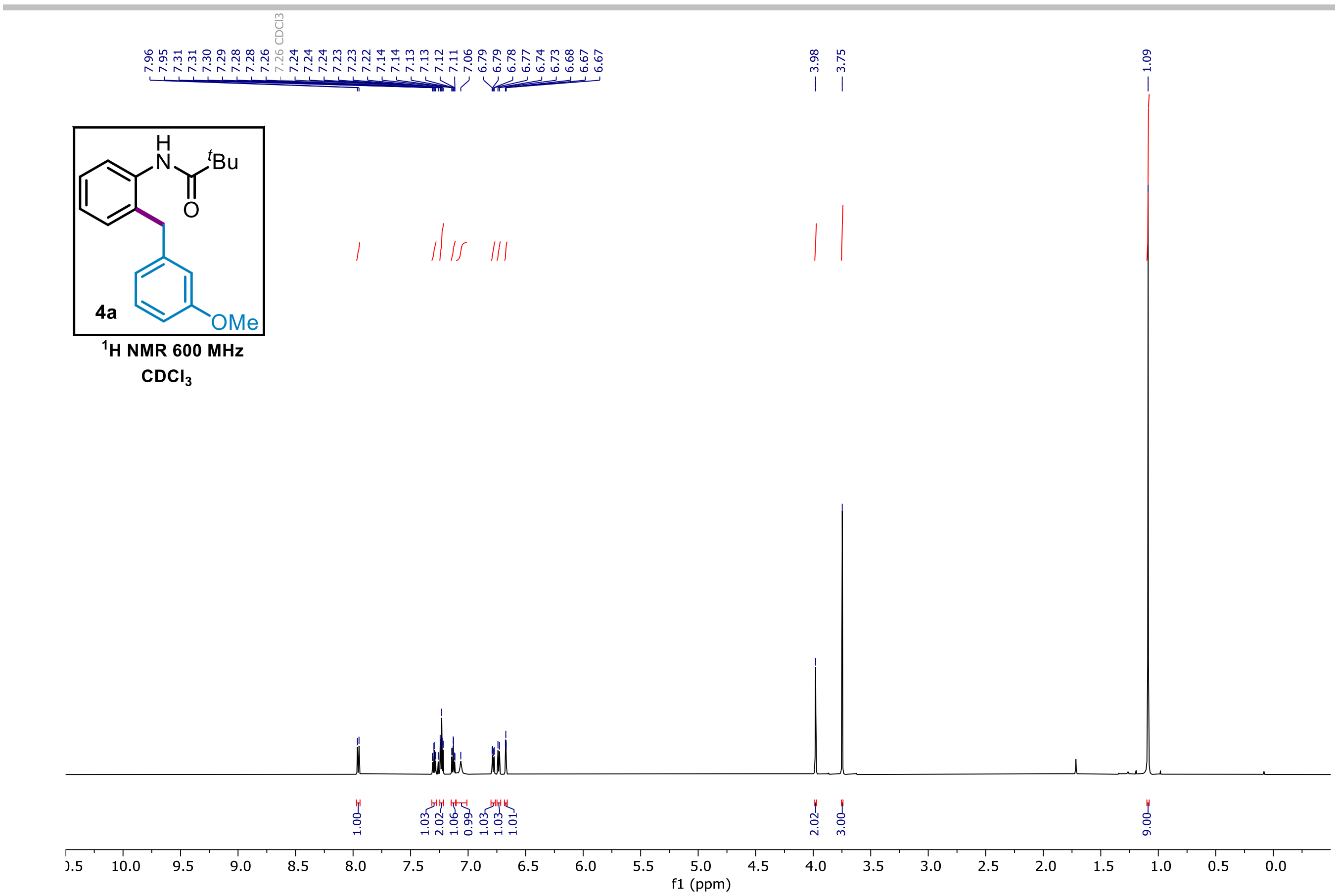
4a
1H NMR 600 MHz
CDCl3
f1 (ppm)

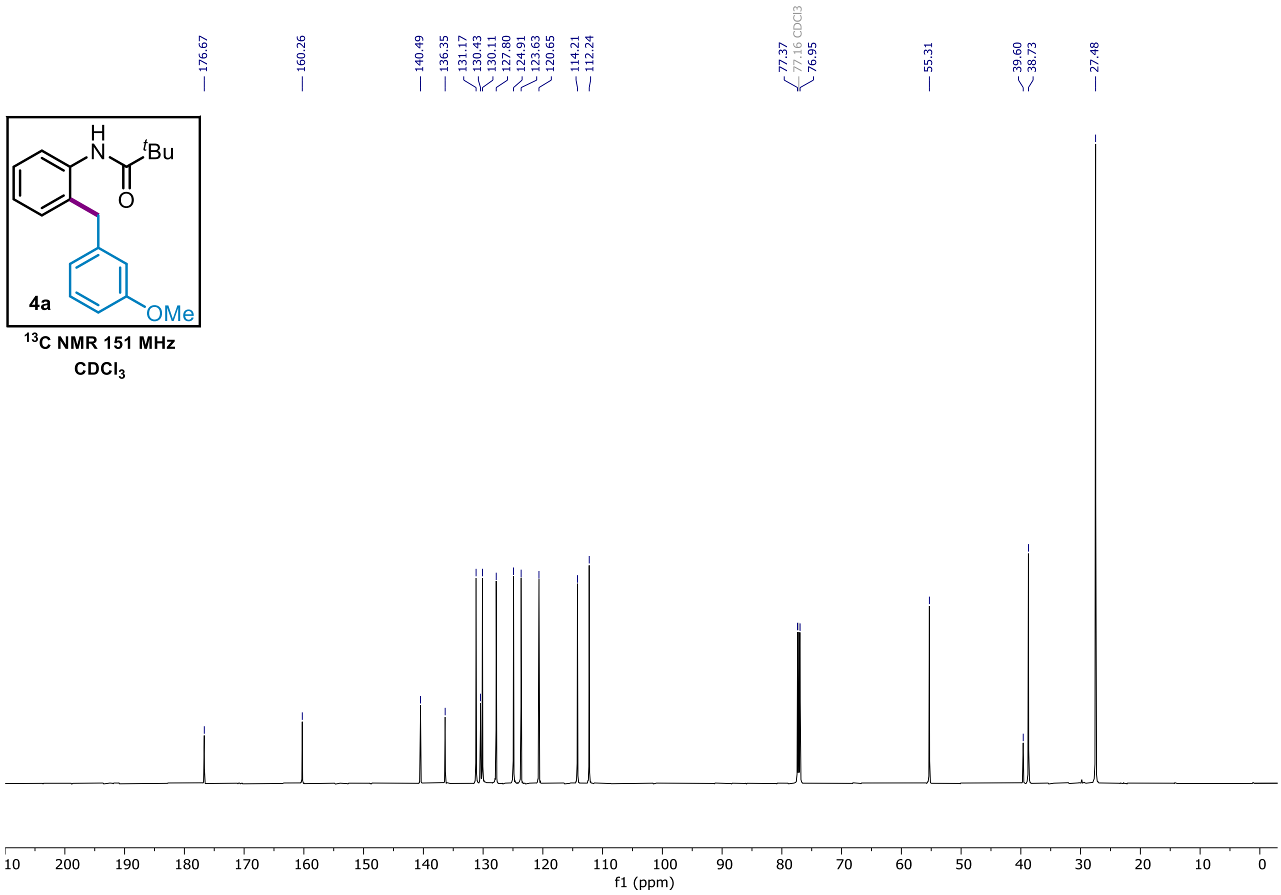
4a
13C NMR 151 MHz
CDCl3
f1 (ppm)

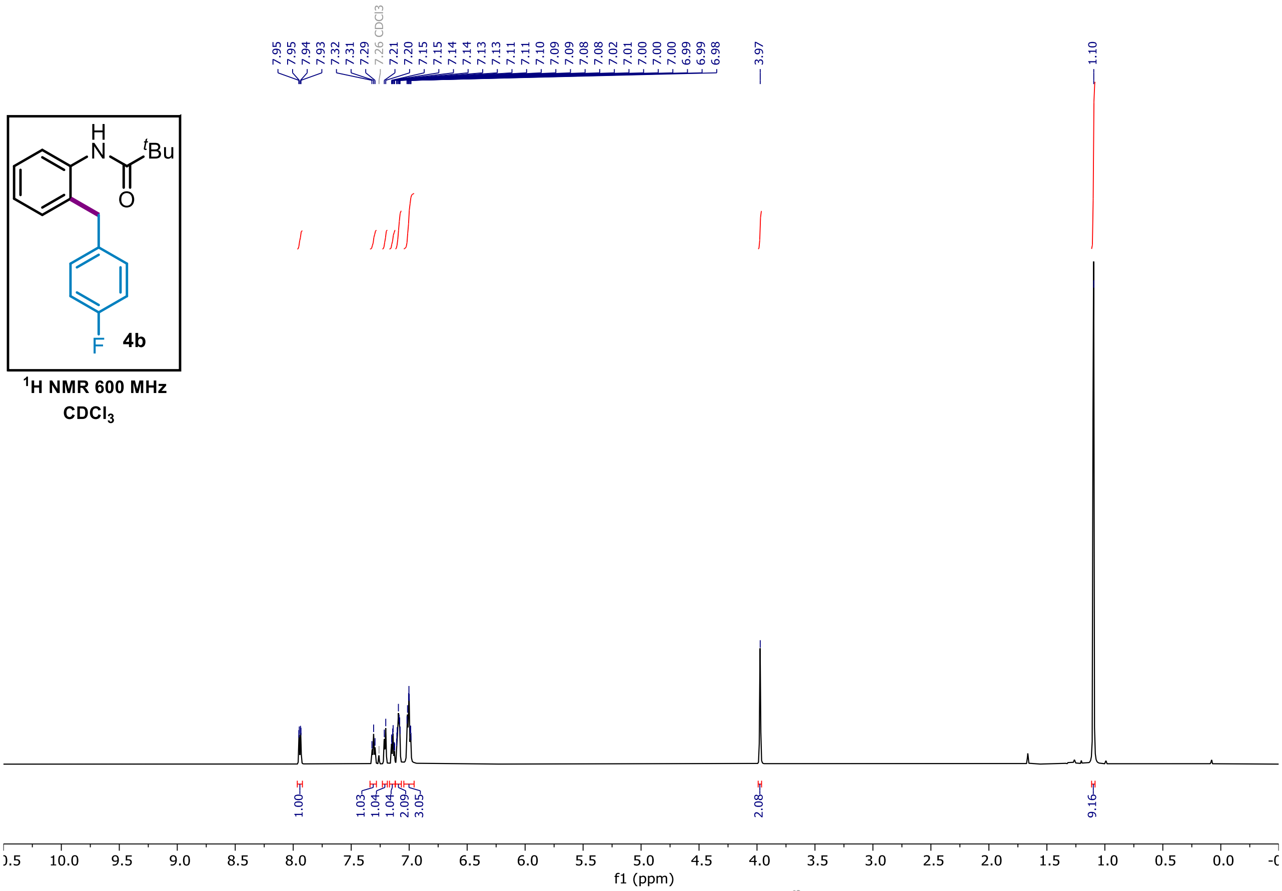

H
N
tBu
O
F
4b
1H NMR 600 MHz
CDCl3
7.95
7.95
7.94
7.93
7.32
7.31
7.29
7.26 CDCl3
7.21
7.20
7.15
7.15
7.14
7.14
7.13
7.13
7.11
7.11
7.10
7.09
7.09
7.08
7.08
7.02
7.01
7.00
7.00
7.00
6.99
6.99
6.98
3.97
1.10
1.00
1.03
1.04
1.04
2.09
3.05
2.08
9.16
f1 (ppm)

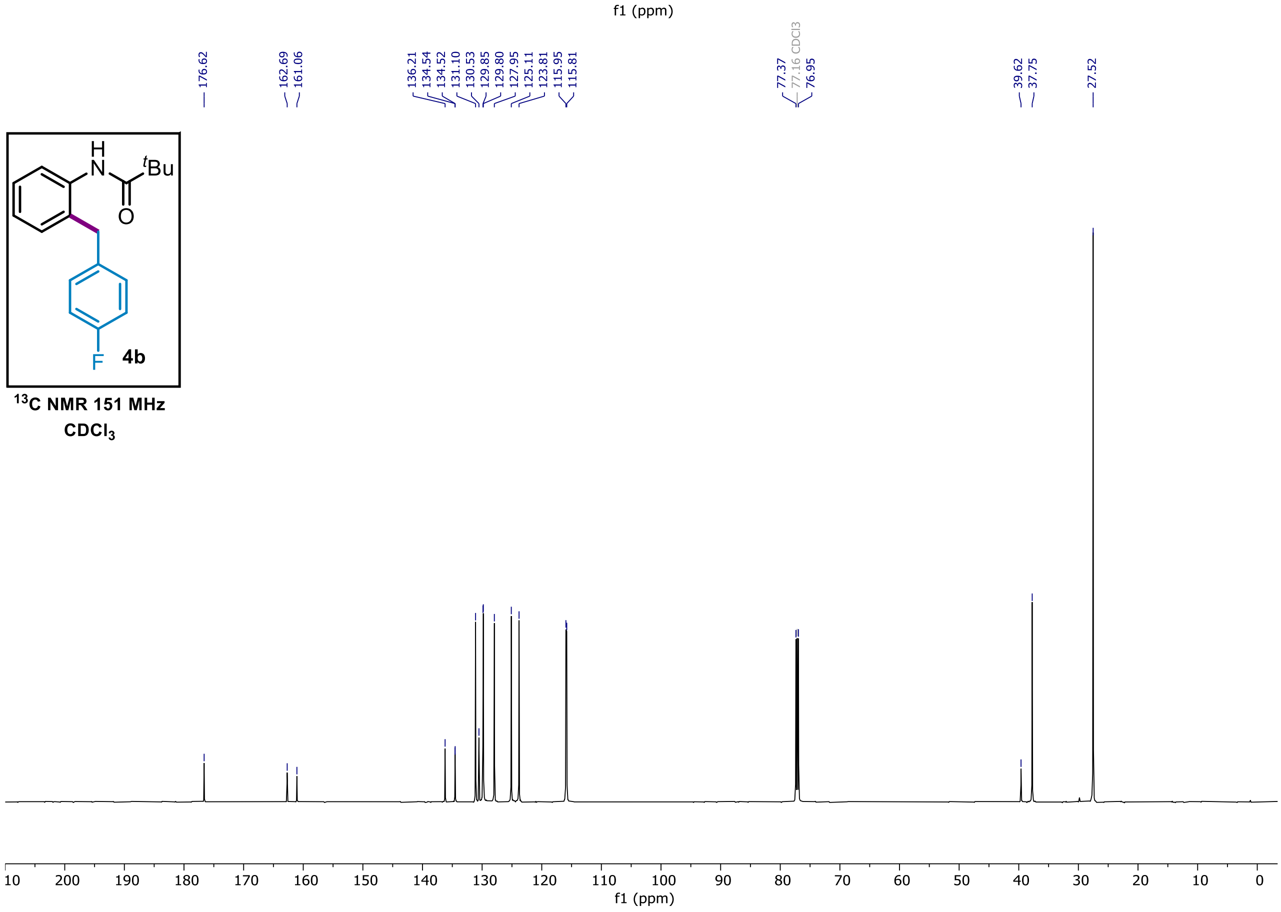

H
N
tBu
O
F
4b
13C NMR 151 MHz
CDCl3
176.62
162.69
161.06
136.21
134.54
134.52
131.10
130.53
129.85
129.80
127.95
125.11
123.81
115.95
115.81
77.37
77.16 CDCl3
76.95
39.62
37.75
27.52
f1 (ppm)

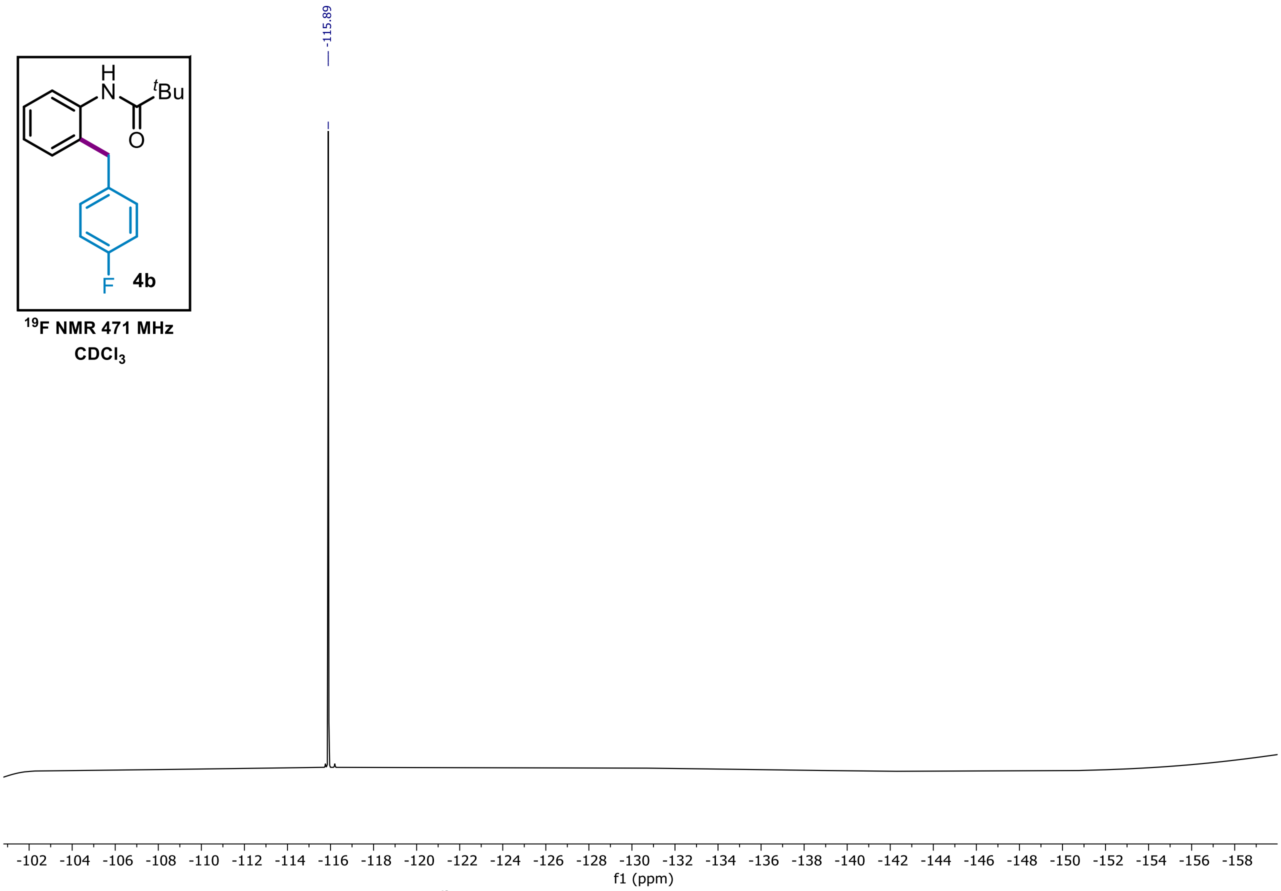

H
N
tBu
O
F
4b
19F NMR 471 MHz
CDCl3
-115.89
-102 -104 -106 -108 -110 -112 -114 -116 -118 -120 -122 -124 -126 -128 -130 -132 -134 -136 -138 -140 -142 -144 -146 -148 -150 -152 -154 -156 -158
f1 (ppm)

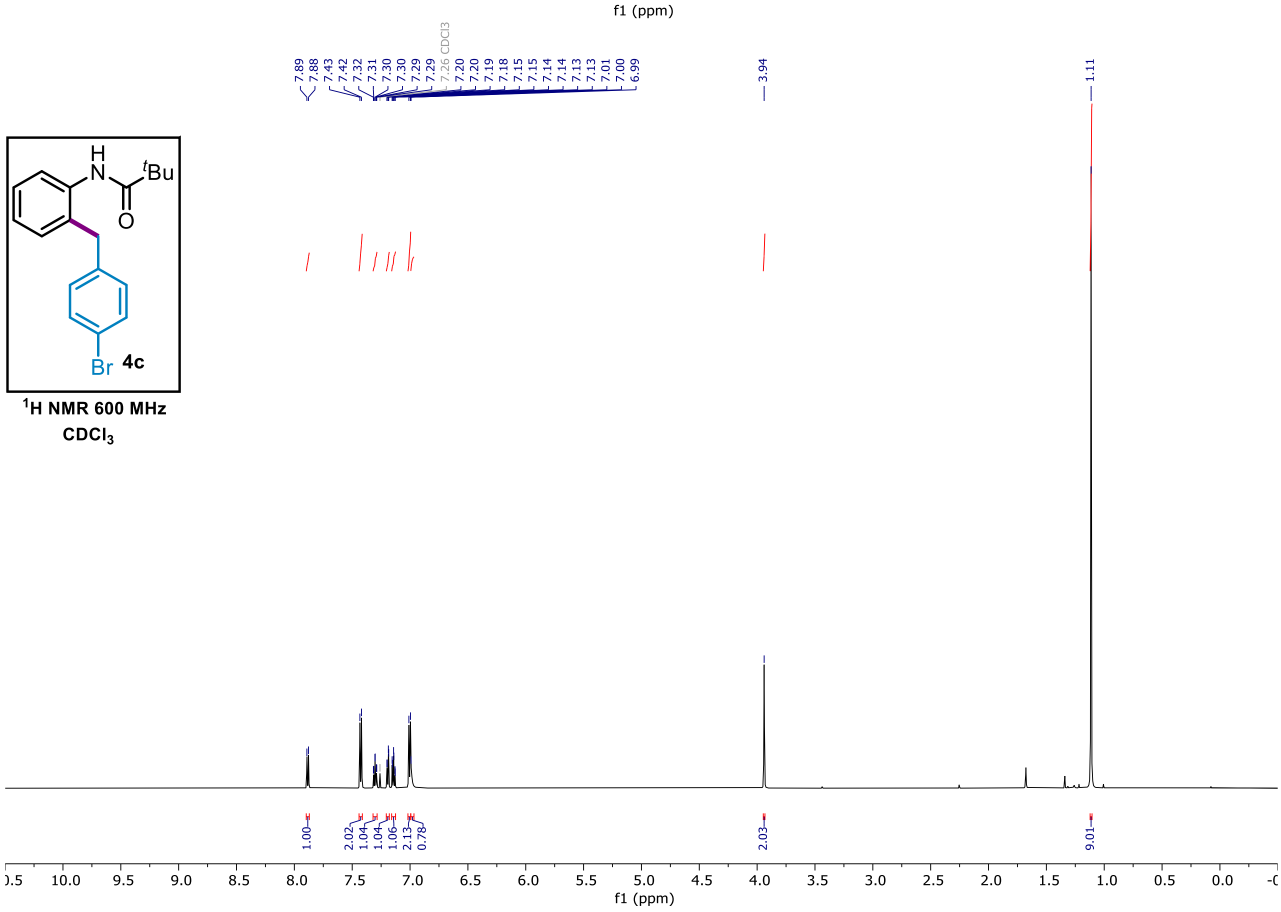

H
N
tBu
O
Br
4c
1H NMR 600 MHz
CDCl3
7.89 7.88 7.43 7.42 7.32 7.31 7.30 7.30 7.29 7.29 7.26 CDCl3 7.20 7.20 7.19 7.18 7.15 7.15 7.14 7.14 7.13 7.13 7.01 7.00 6.99
3.94
1.11
1.00 2.02 1.04 1.04 1.06 2.13 0.78 2.03 9.01
10.0 9.5 9.0 8.5 8.0 7.5 7.0 6.5 6.0 5.5 5.0 4.5 4.0 3.5 3.0 2.5 2.0 1.5 1.0 0.5 0.0
f1 (ppm)

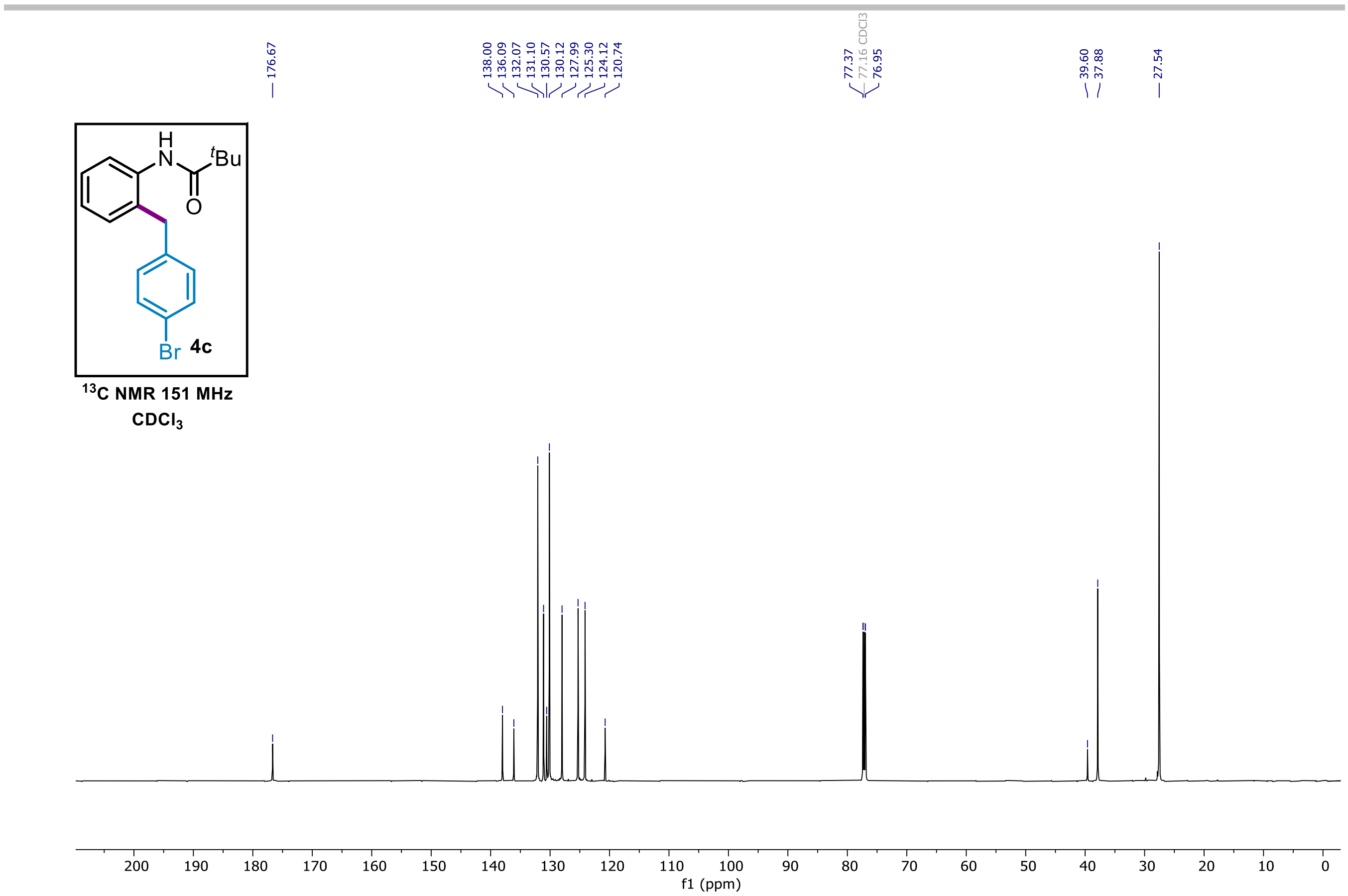

4c
13C NMR 151 MHz
CDCl3
176.67
138.00
136.09
132.07
131.10
130.57
130.12
127.99
125.30
124.12
120.74
77.37
77.16 CDCl3
76.95
39.60
37.88
27.54
f1 (ppm)

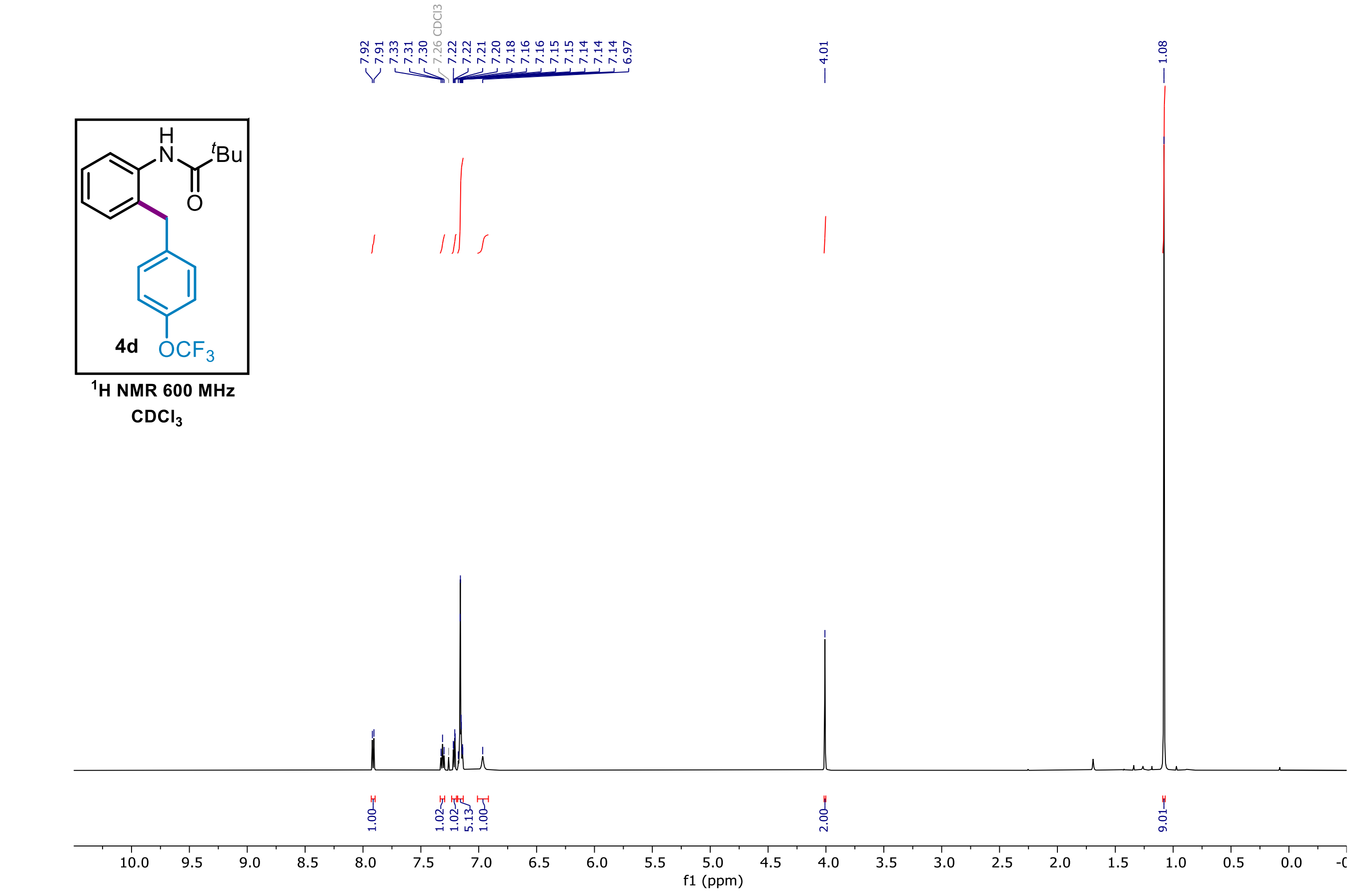

4d
1H NMR 600 MHz
CDCl3
7.92
7.91
7.33
7.31
7.30
7.26 CDCl3
7.22
7.22
7.21
7.20
7.18
7.16
7.16
7.15
7.15
7.14
7.14
7.14
6.97
4.01
1.08
1.00
1.02
1.02
5.13
1.00
2.00
9.01
f1 (ppm)

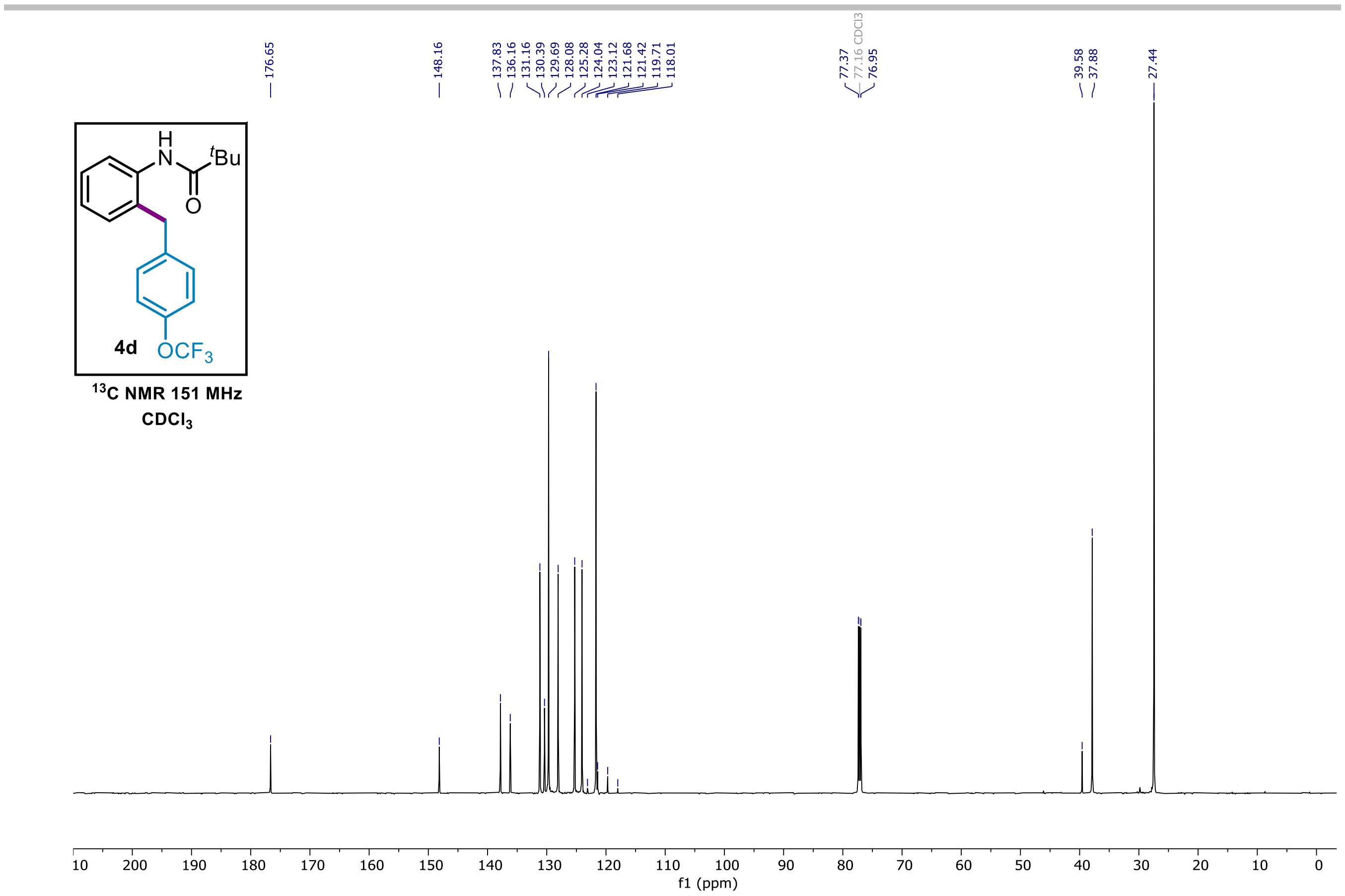

176.65
148.16
137.83
136.16
131.16
130.39
129.69
128.08
125.28
124.04
123.12
121.68
121.42
119.71
118.01
77.37
77.16 CDCl3
76.95
39.58
37.88
27.44
H
N
tBu
O
4d
OCF3
13C NMR 151 MHz
CDCl3
f1 (ppm)

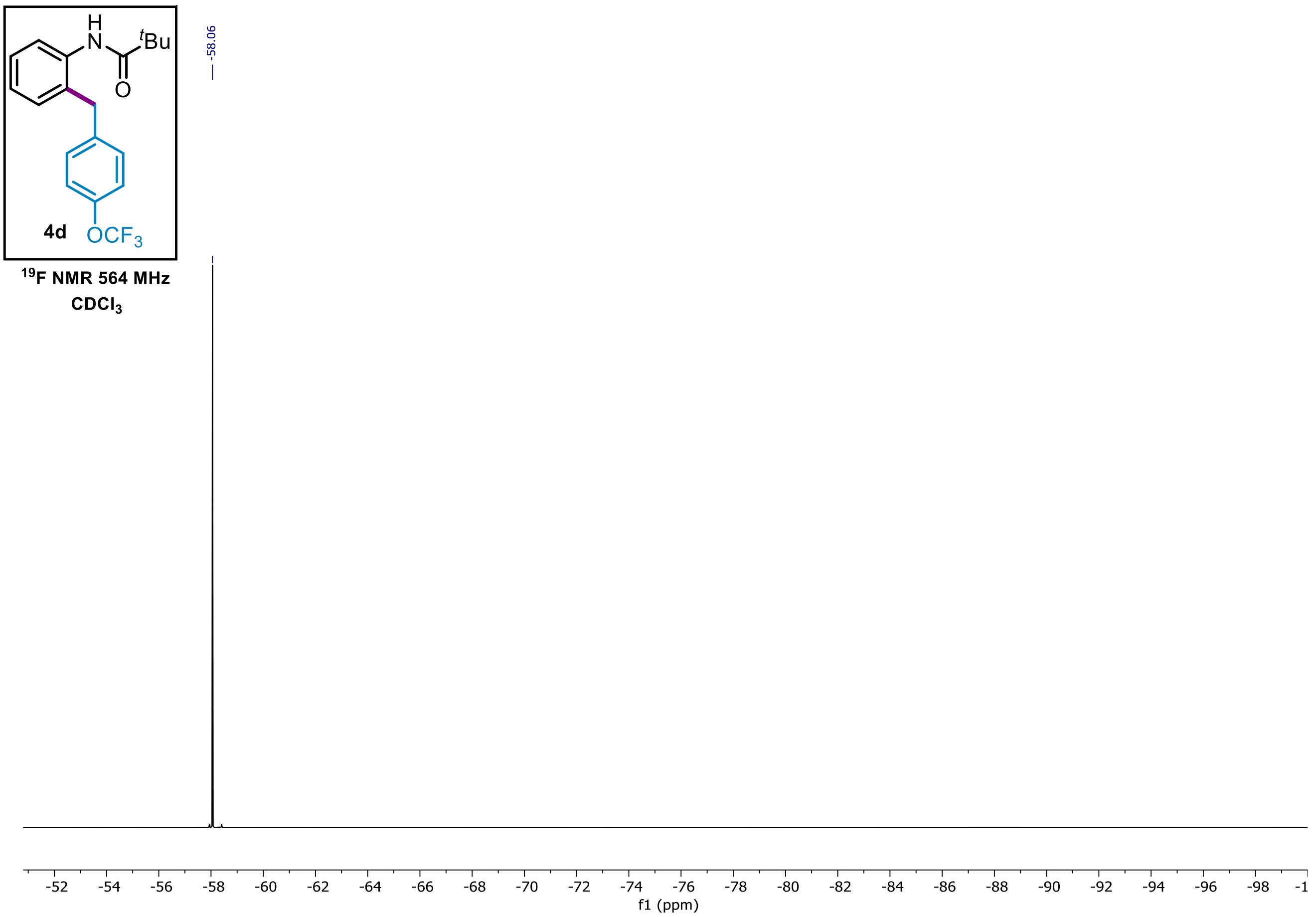

-58.06
H
N
tBu
O
4d
OCF3
19F NMR 564 MHz
CDCl3
f1 (ppm)

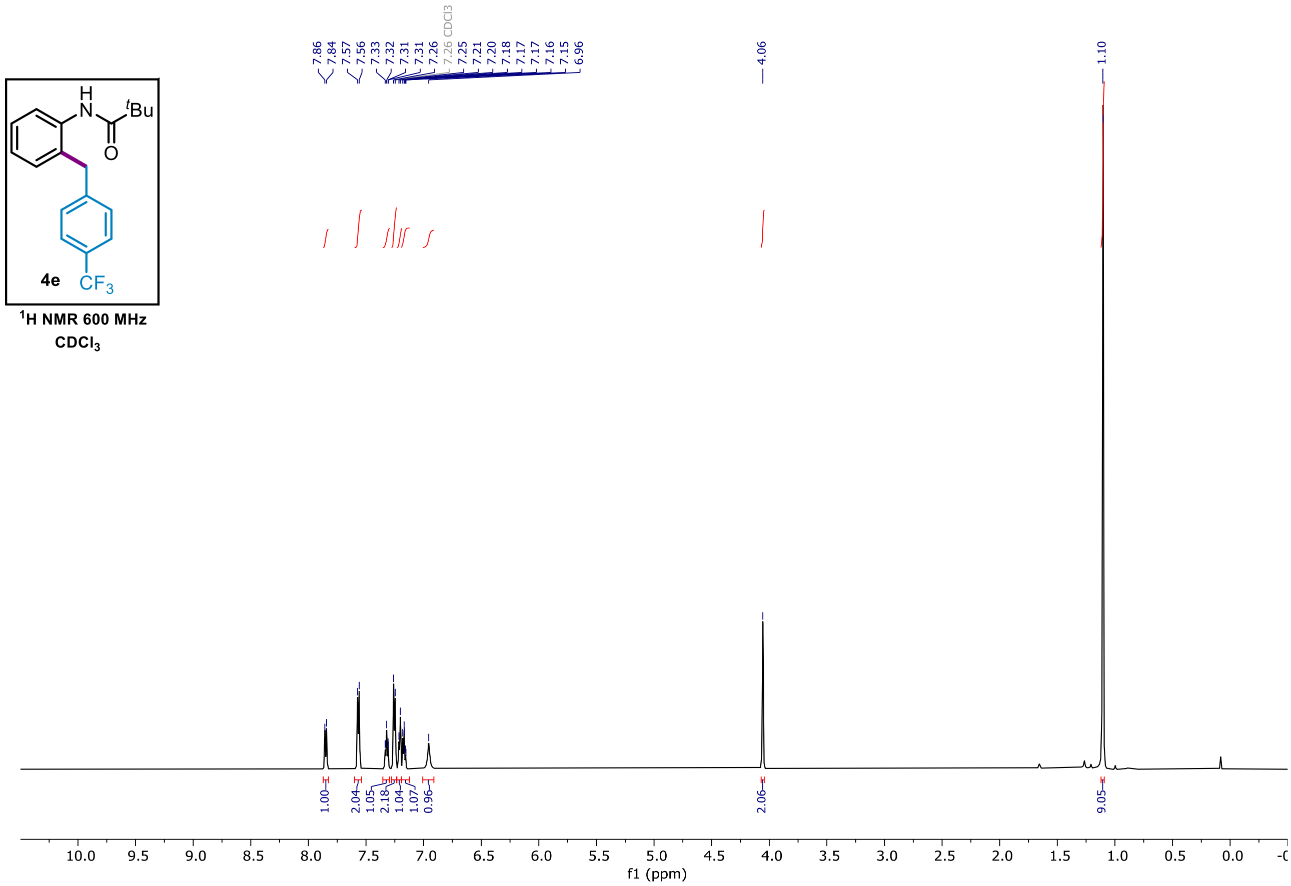

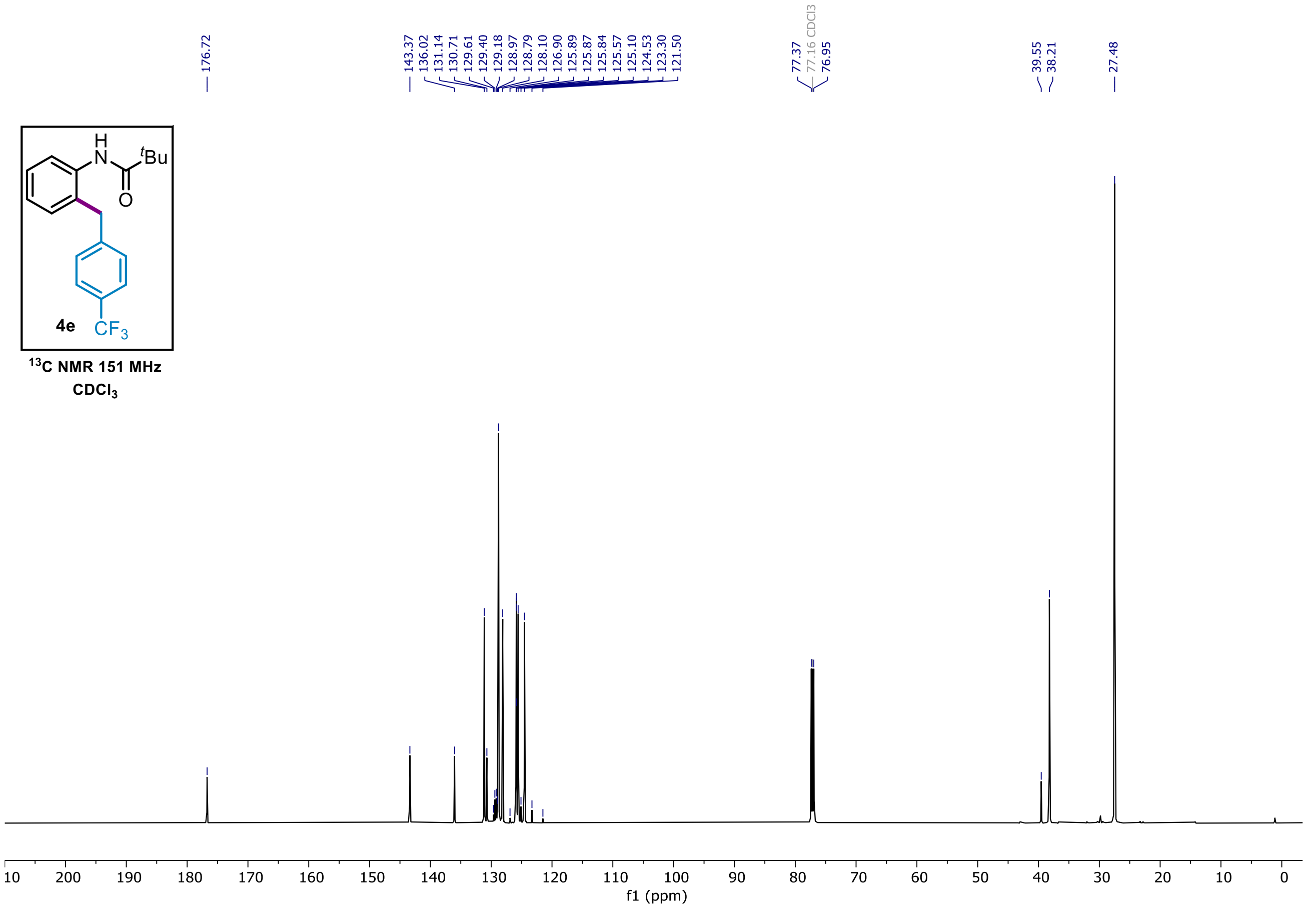

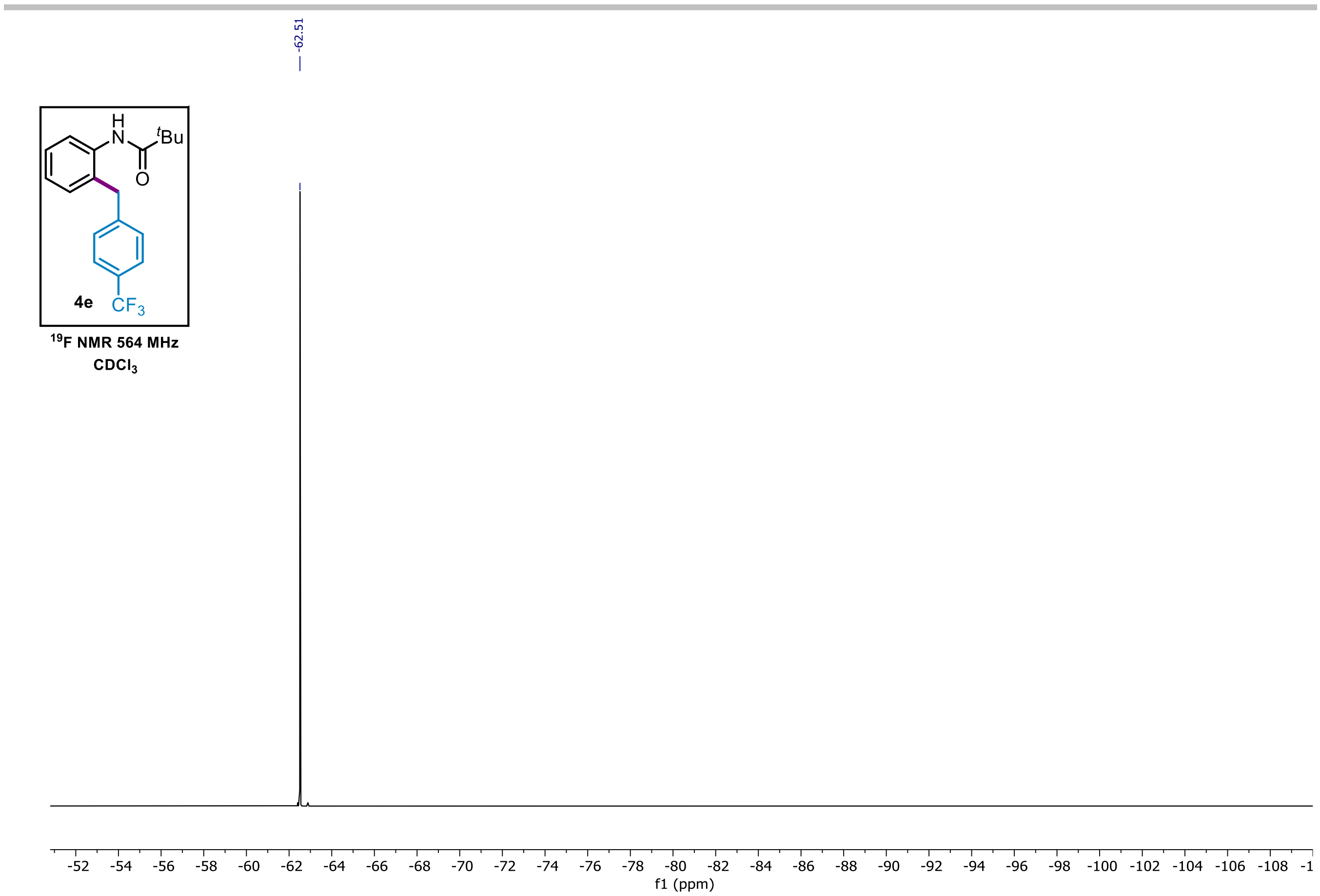
-62.51
4e
^{t}Bu
CF3
19F NMR 564 MHz
CDCl3
-52 -54 -56 -58 -60 -62 -64 -66 -68 -70 -72 -74 -76 -78 -80 -82 -84 -86 -88 -90 -92 -94 -96 -98 -100 -102 -104 -106 -108
f1 (ppm)

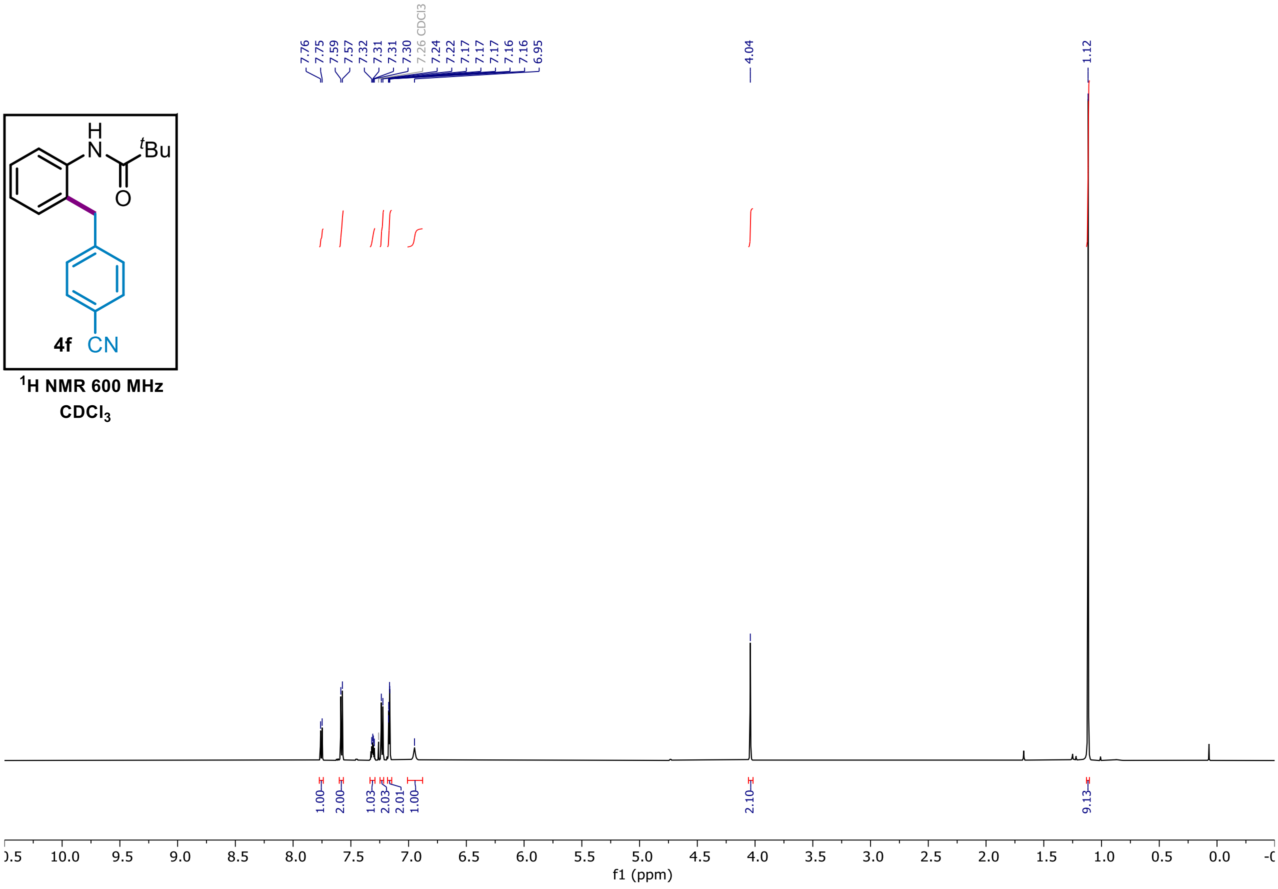
7.76 7.75 7.59 7.57 7.32 7.31 7.31 7.30 7.26 CDCl3 7.24 7.22 7.17 7.17 7.17 7.16 6.95
4.04
1.12
4f
^{t}Bu
CN
1H NMR 600 MHz
CDCl3
1.00 2.00 1.03 2.03 2.01 1.00
2.10
9.13
10.0 9.5 9.0 8.5 8.0 7.5 7.0 6.5 6.0 5.5 5.0 4.5 4.0 3.5 3.0 2.5 2.0 1.5 1.0 0.5 0.0
f1 (ppm)

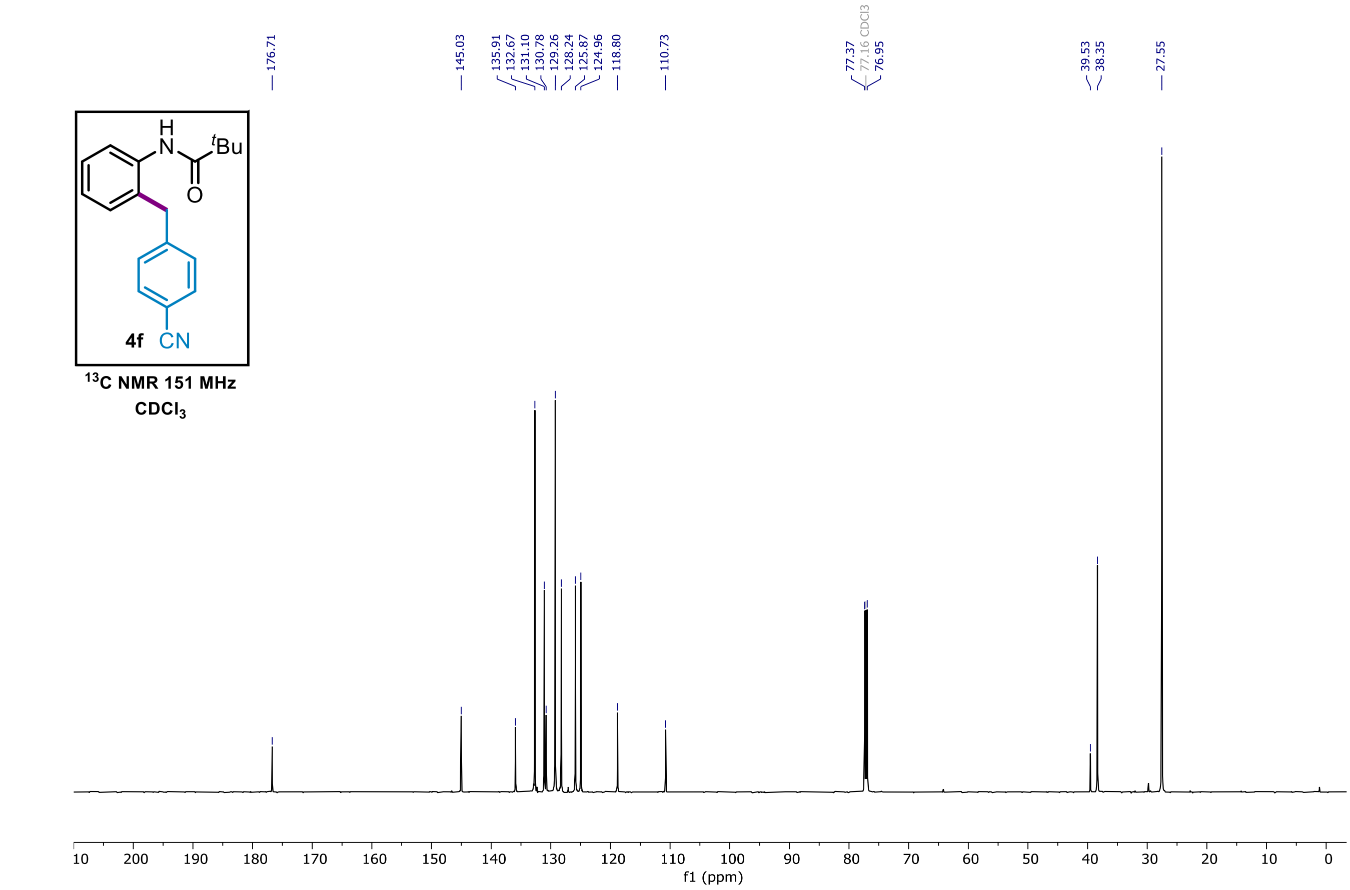

^{13}C NMR 151 MHz
$CDCl_3$

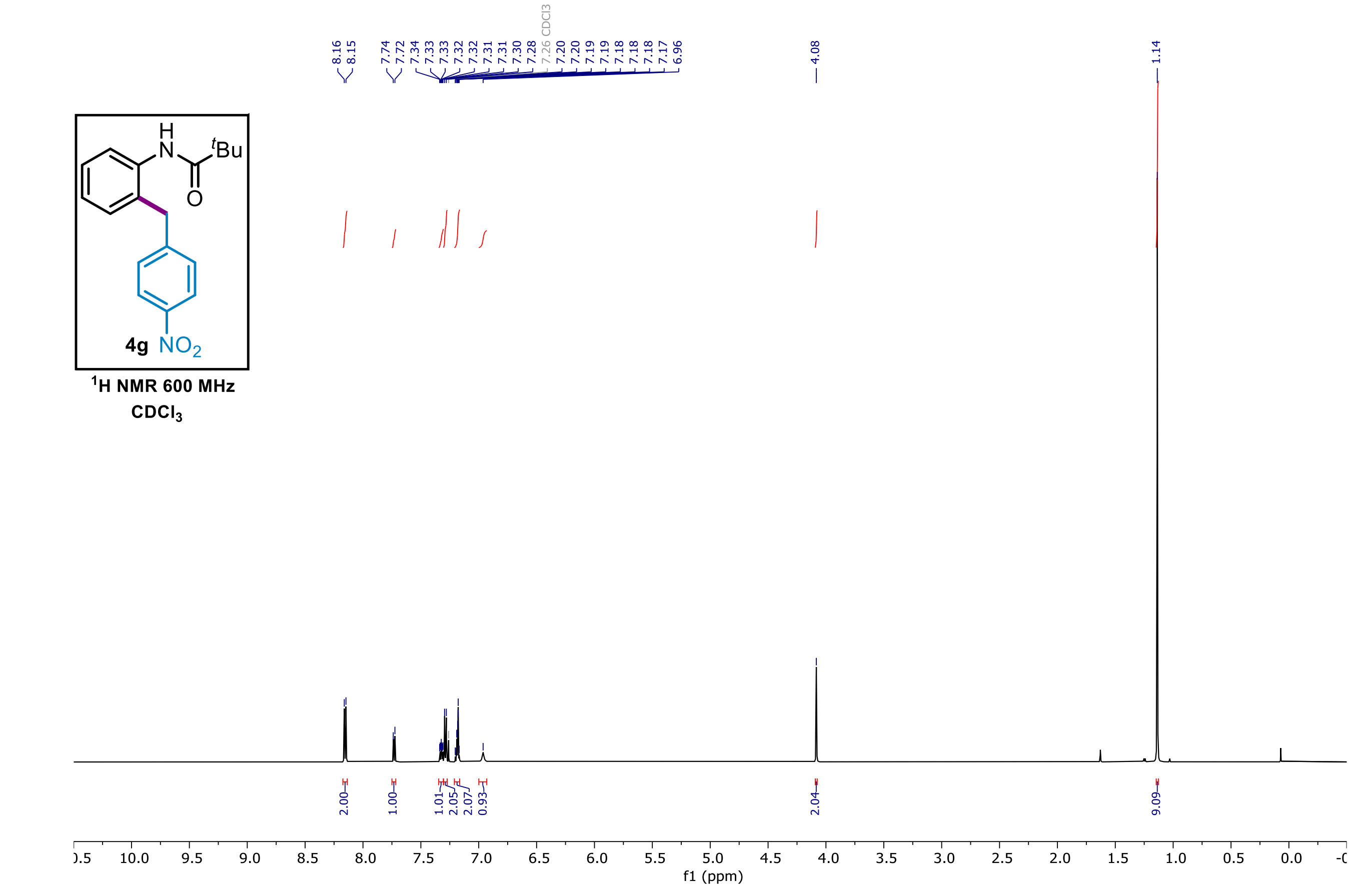

^{1}H NMR 600 MHz
$CDCl_3$

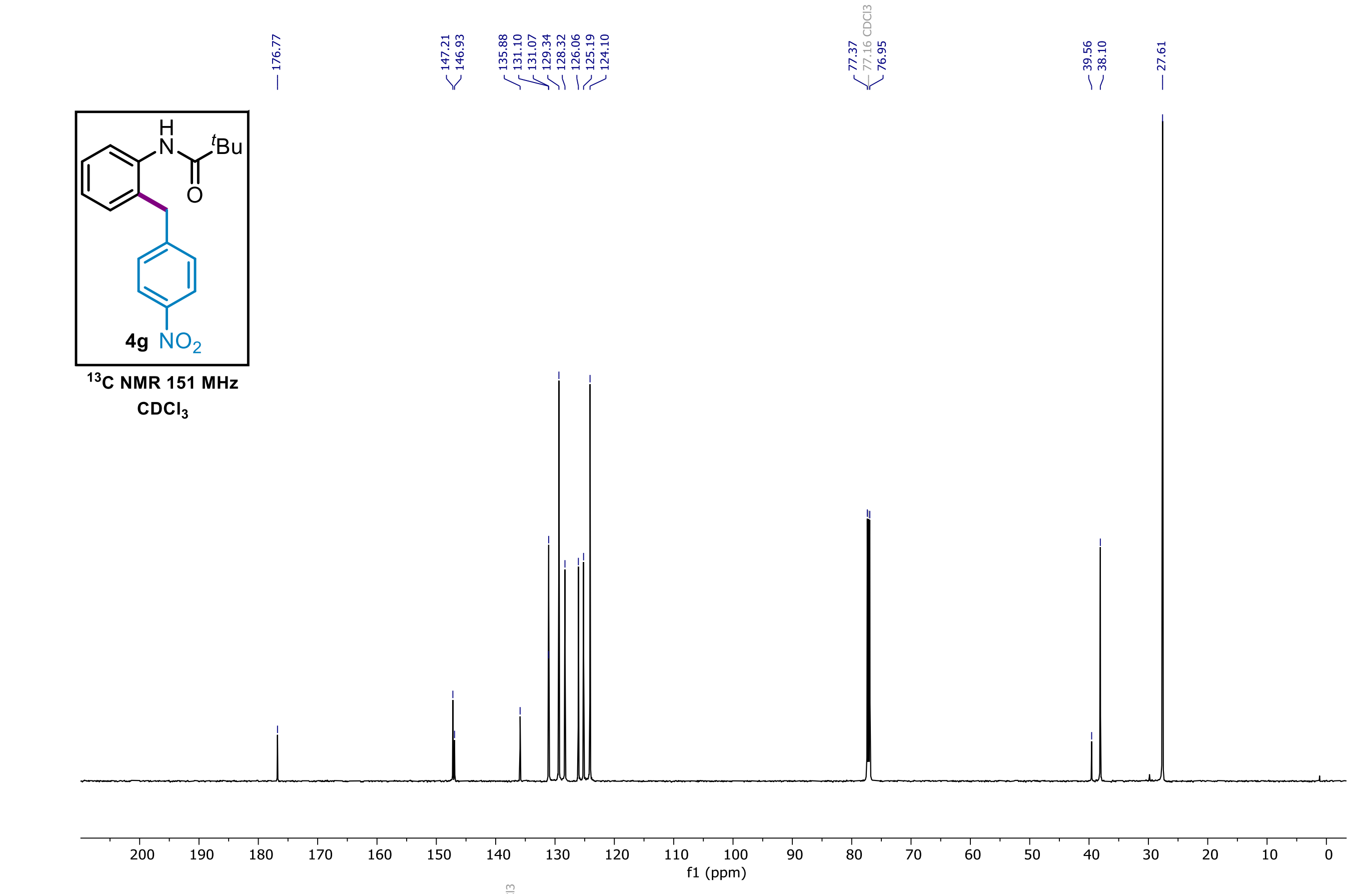
4g NO2
13C NMR 151 MHz
CDCl3
f1 (ppm)

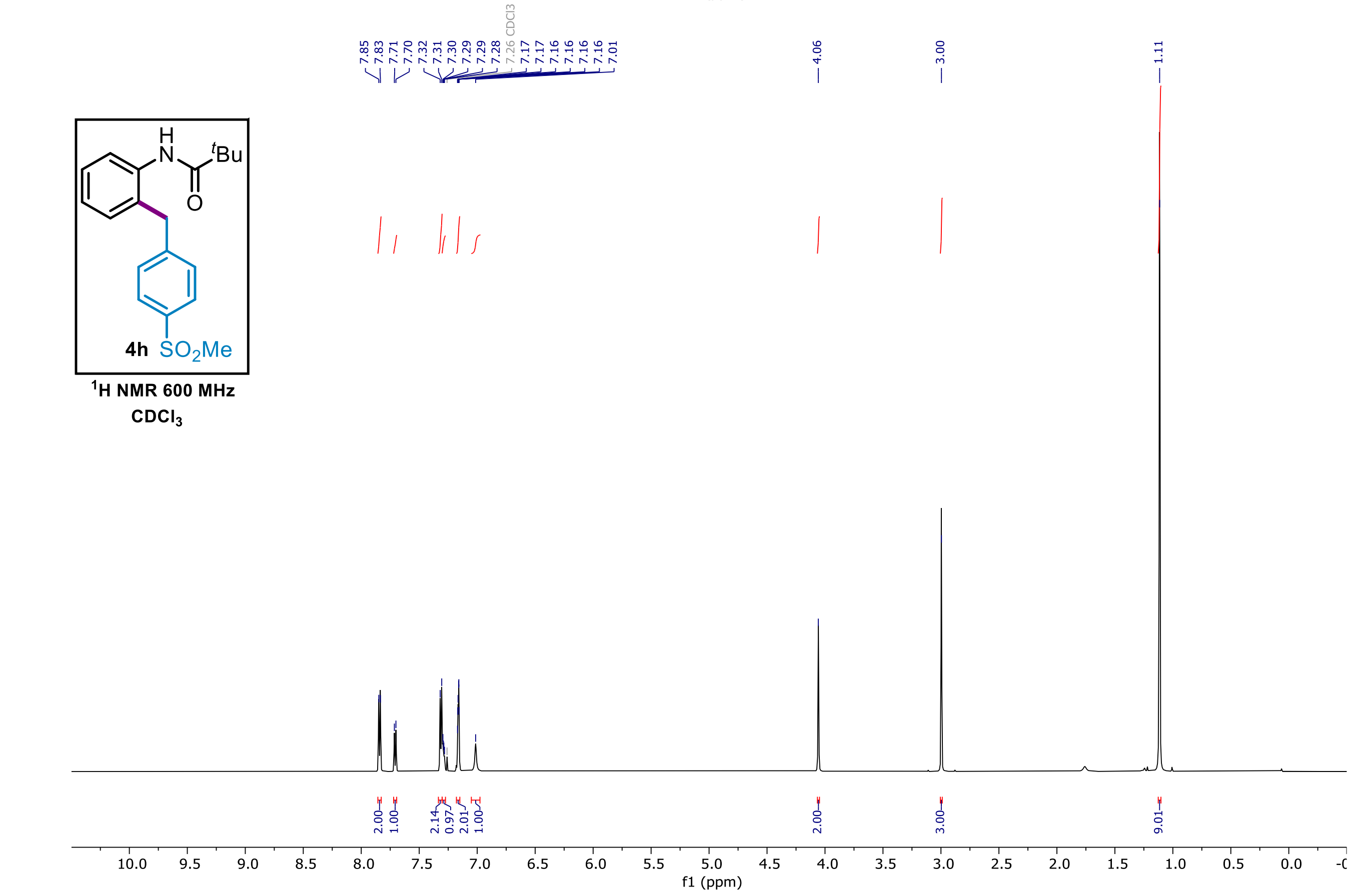
4h SO2Me
1H NMR 600 MHz
CDCl3
f1 (ppm)

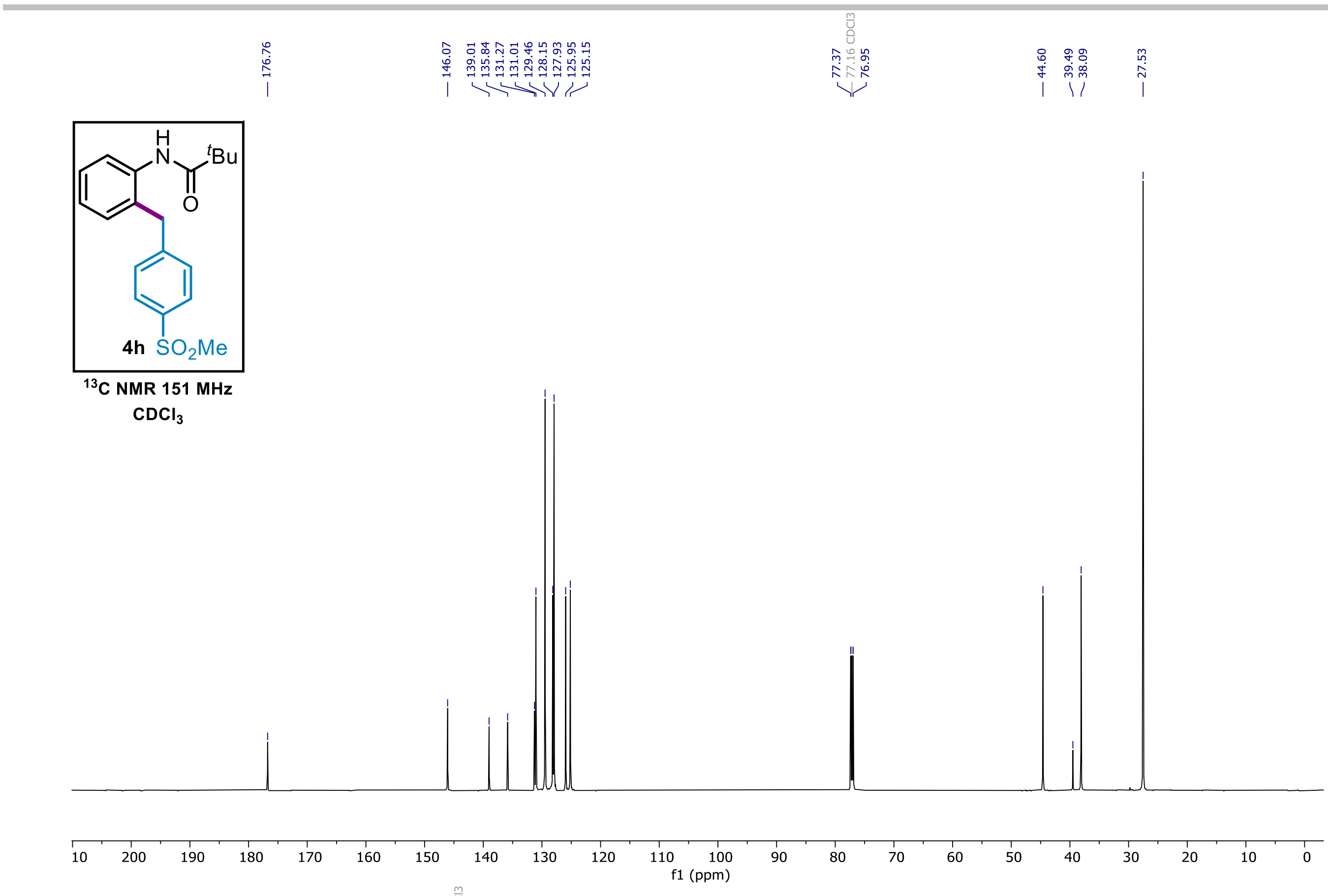
176.76
146.07
139.01
135.84
131.27
131.01
129.46
128.15
127.93
125.95
125.15
77.37
77.16 CDCl3
76.95
44.60
39.49
38.09
27.53
H
N
tBu
O
4h
SO2Me
13C NMR 151 MHz
CDCl3
f1 (ppm)

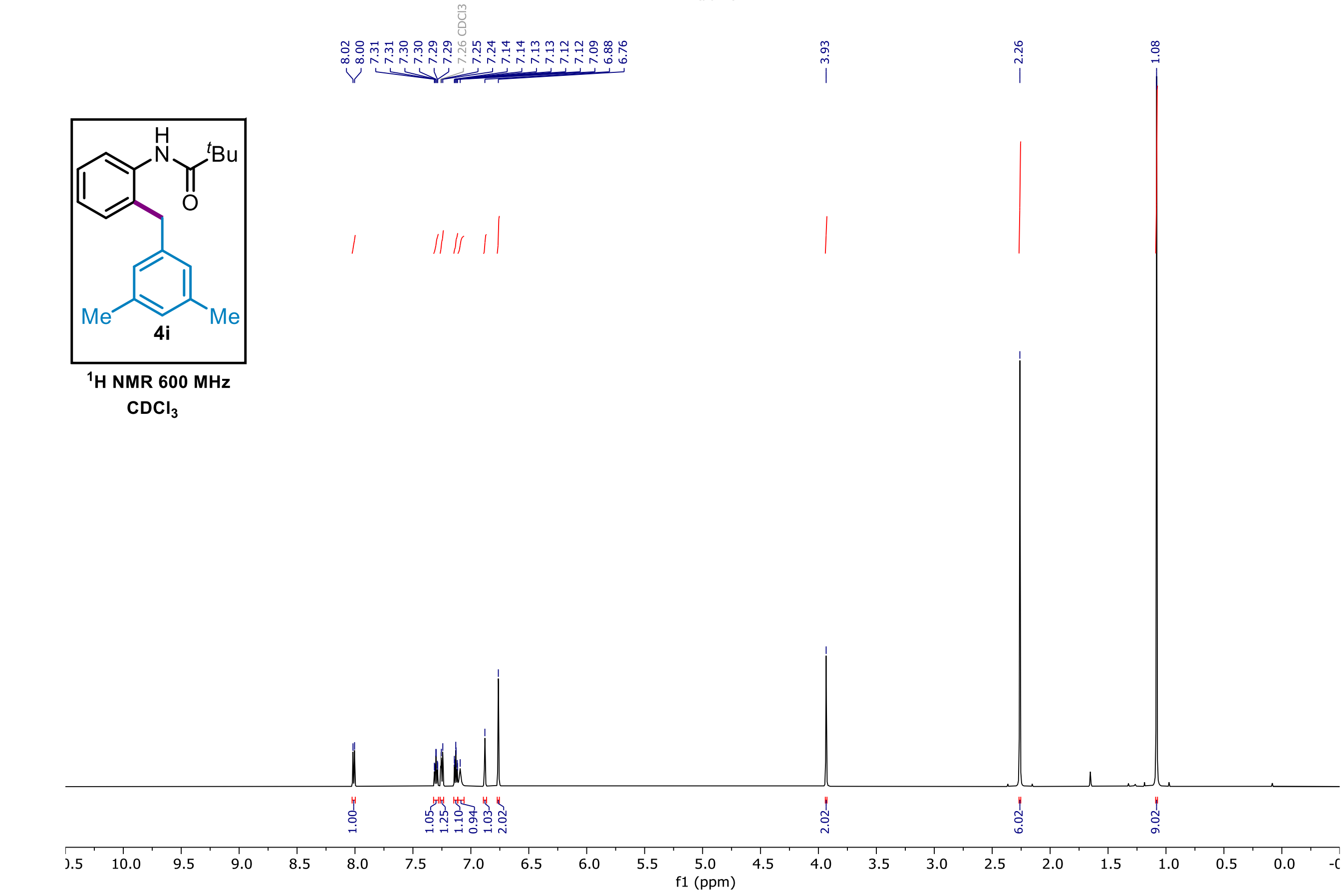
8.02
8.00
7.31
7.31
7.30
7.30
7.29
7.29
7.26 CDCl3
7.25
7.24
7.14
7.14
7.13
7.13
7.12
7.12
7.09
6.88
6.76
3.93
2.26
1.08
H
N
tBu
O
Me
Me
4i
1H NMR 600 MHz
CDCl3
1.00
1.05
1.25
1.10
0.94
1.03
2.02
2.02
6.02
9.02
f1 (ppm)

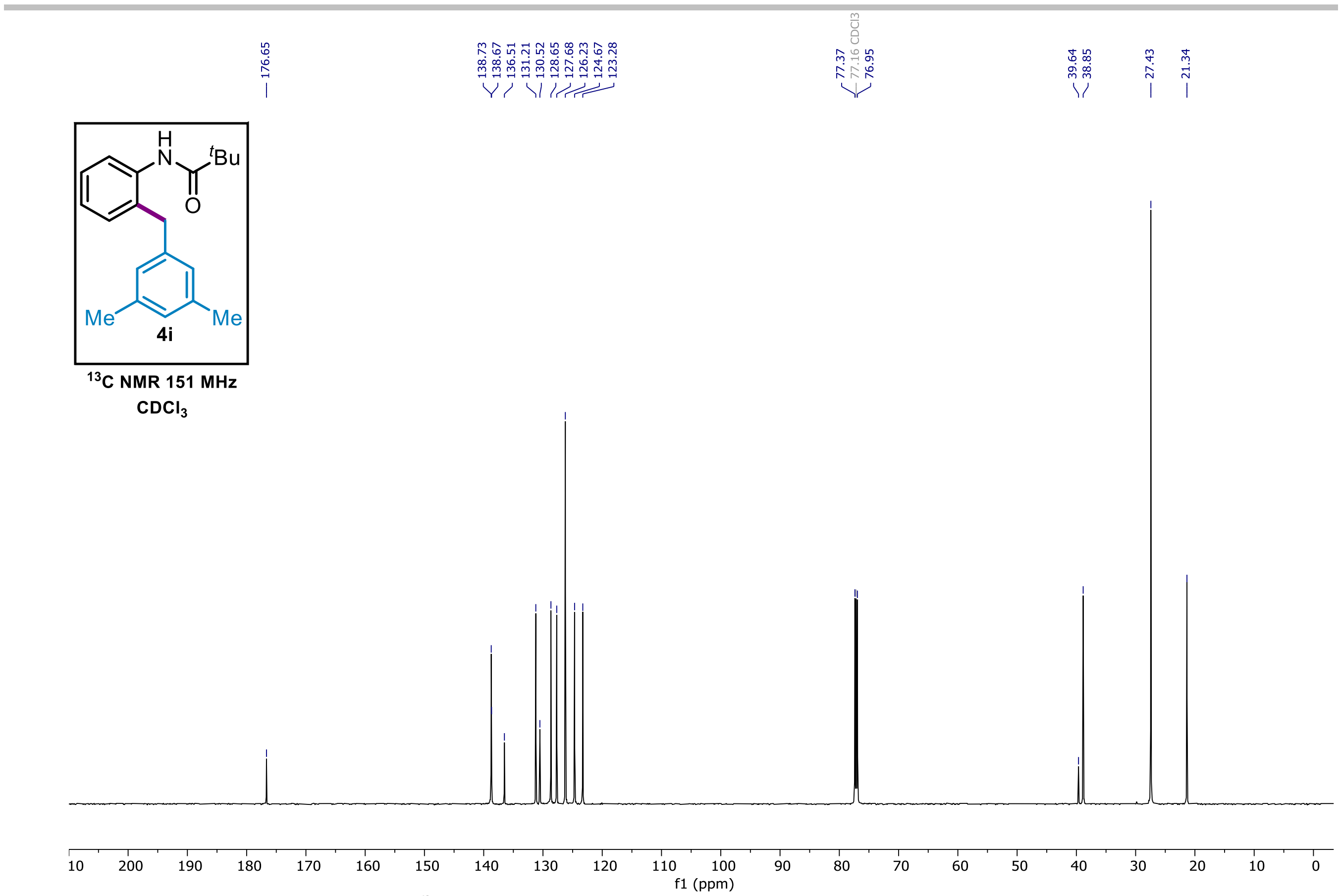

176.65
138.73
138.67
136.51
131.21
130.52
128.65
127.68
126.23
124.67
123.28
77.37
77.16 CDCl3
76.95
39.64
38.85
27.43
21.34
H
N
tBu
O
Me
Me
4i
13C NMR 151 MHz
CDCl3
f1 (ppm)

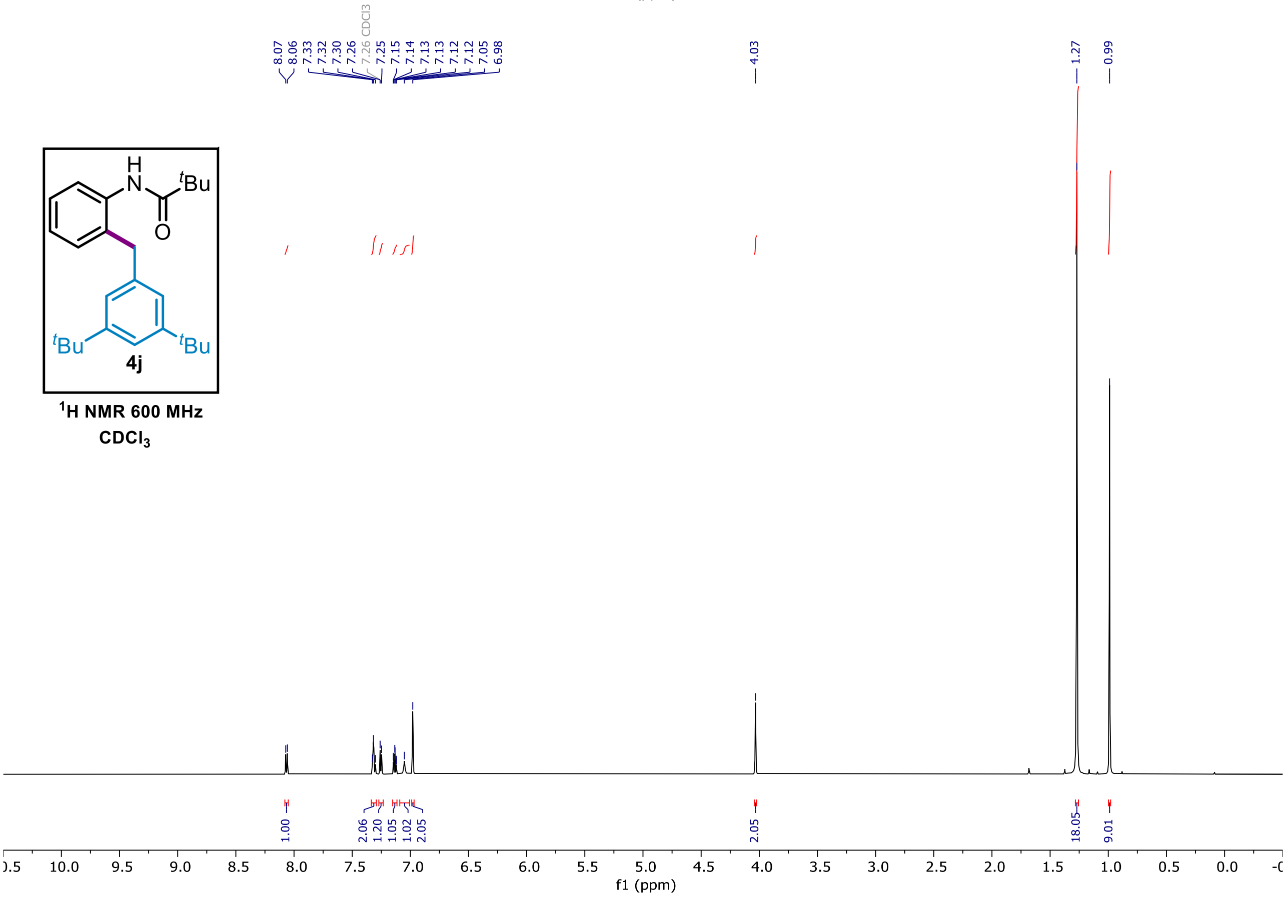

8.07
8.06
7.33
7.32
7.30
7.26
7.26 CDCl3
7.25
7.15
7.14
7.13
7.13
7.12
7.12
7.05
6.98
4.03
1.27
0.99
H
N
tBu
O
tBu
tBu
4j
1H NMR 600 MHz
CDCl3
1.00
2.06
1.20
1.05
1.02
2.05
2.05
18.05
9.01
f1 (ppm)

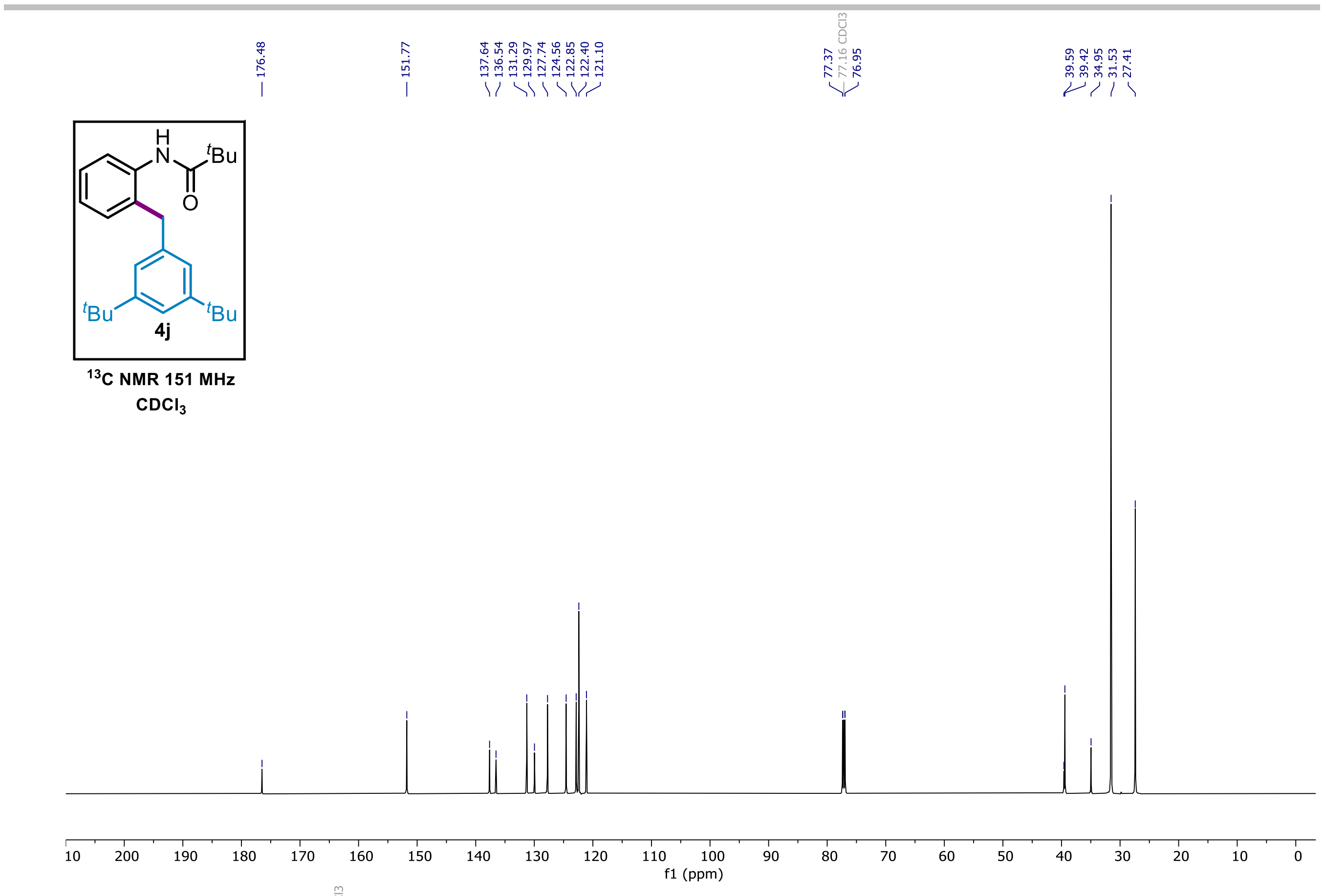

176.48
151.77
137.64
136.54
131.29
129.97
127.74
124.56
122.85
122.40
121.10
77.37
77.16 CDCl3
76.95
39.59
39.42
34.95
31.53
27.41
H
N
tBu
O
tBu
tBu
4j
13C NMR 151 MHz
CDCl3
f1 (ppm)

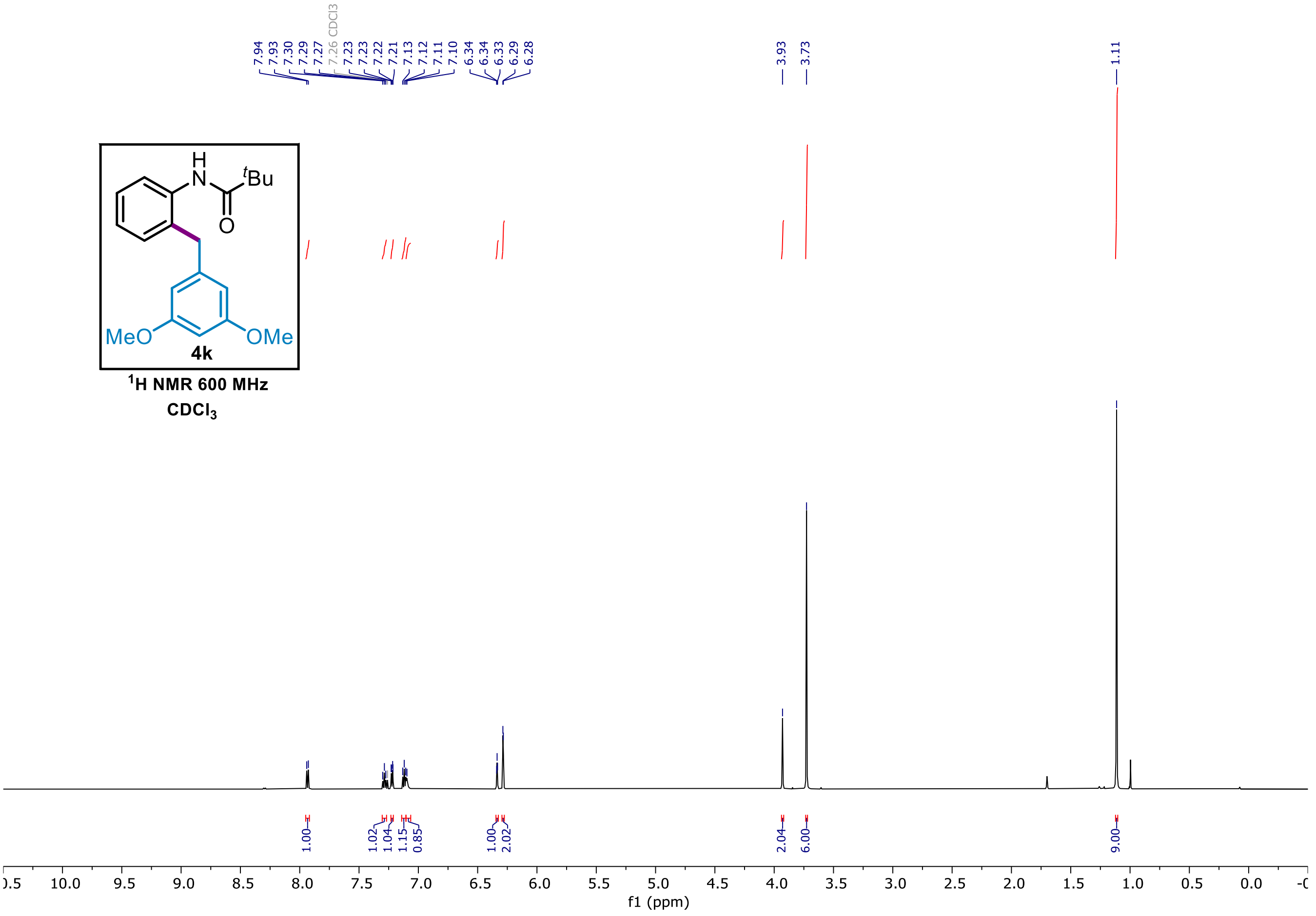

7.94
7.93
7.30
7.29
7.27
7.26 CDCl3
7.23
7.23
7.22
7.21
7.13
7.12
7.11
7.10
6.34
6.34
6.33
6.29
6.28
3.93
3.73
1.11
H
N
tBu
O
MeO
OMe
4k
1H NMR 600 MHz
CDCl3
1.00
1.02
1.04
1.15
0.85
1.00
2.02
2.04
6.00
9.00
f1 (ppm)

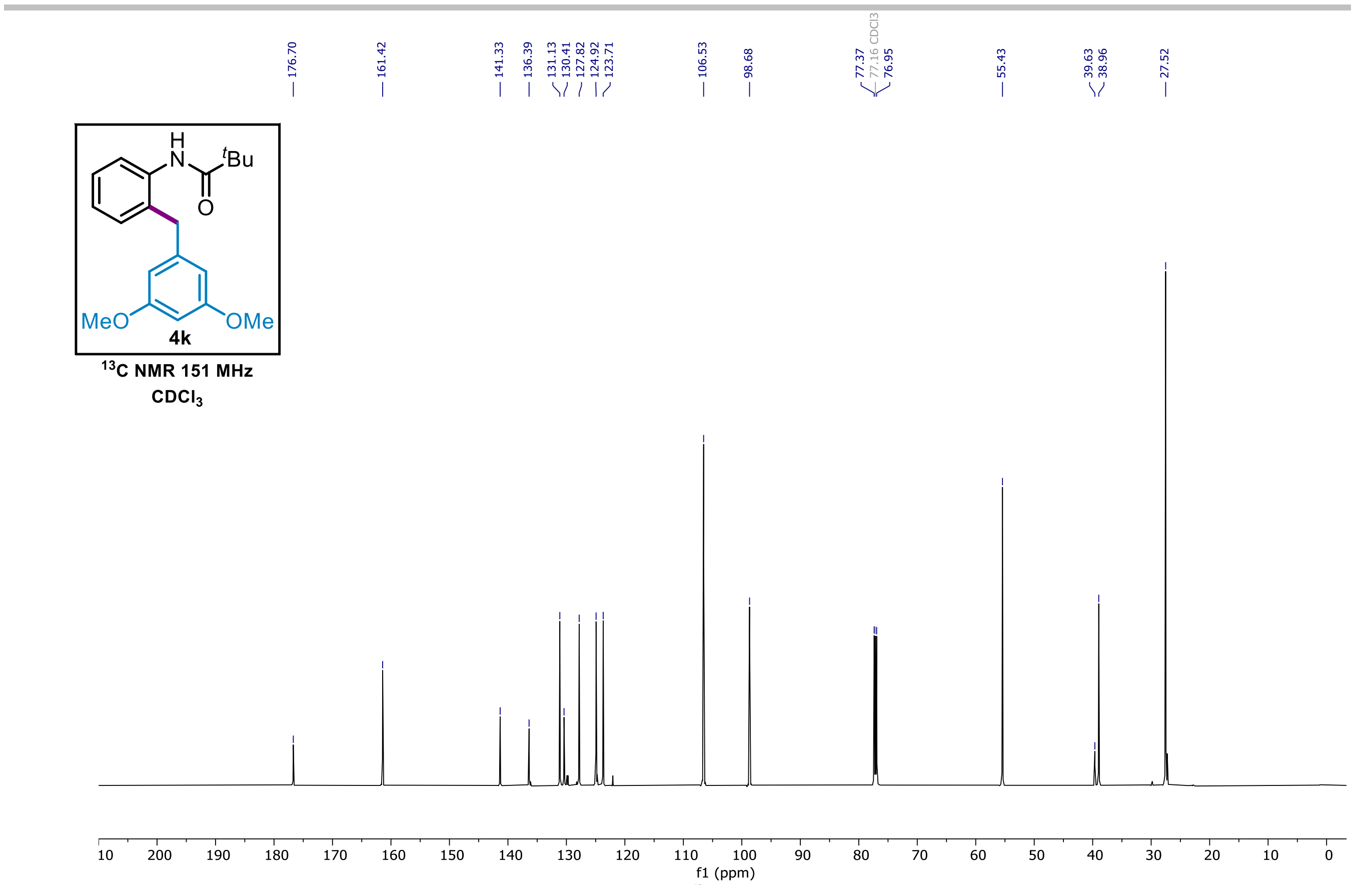
176.70
161.42
141.33
136.39
131.13
130.41
127.82
124.92
123.71
106.53
98.68
77.37
77.16 CDCl3
76.95
55.43
39.63
38.96
27.52
MeO
OMe
4k
^{13}C NMR 151 MHz
$CDCl_3$
f1 (ppm)

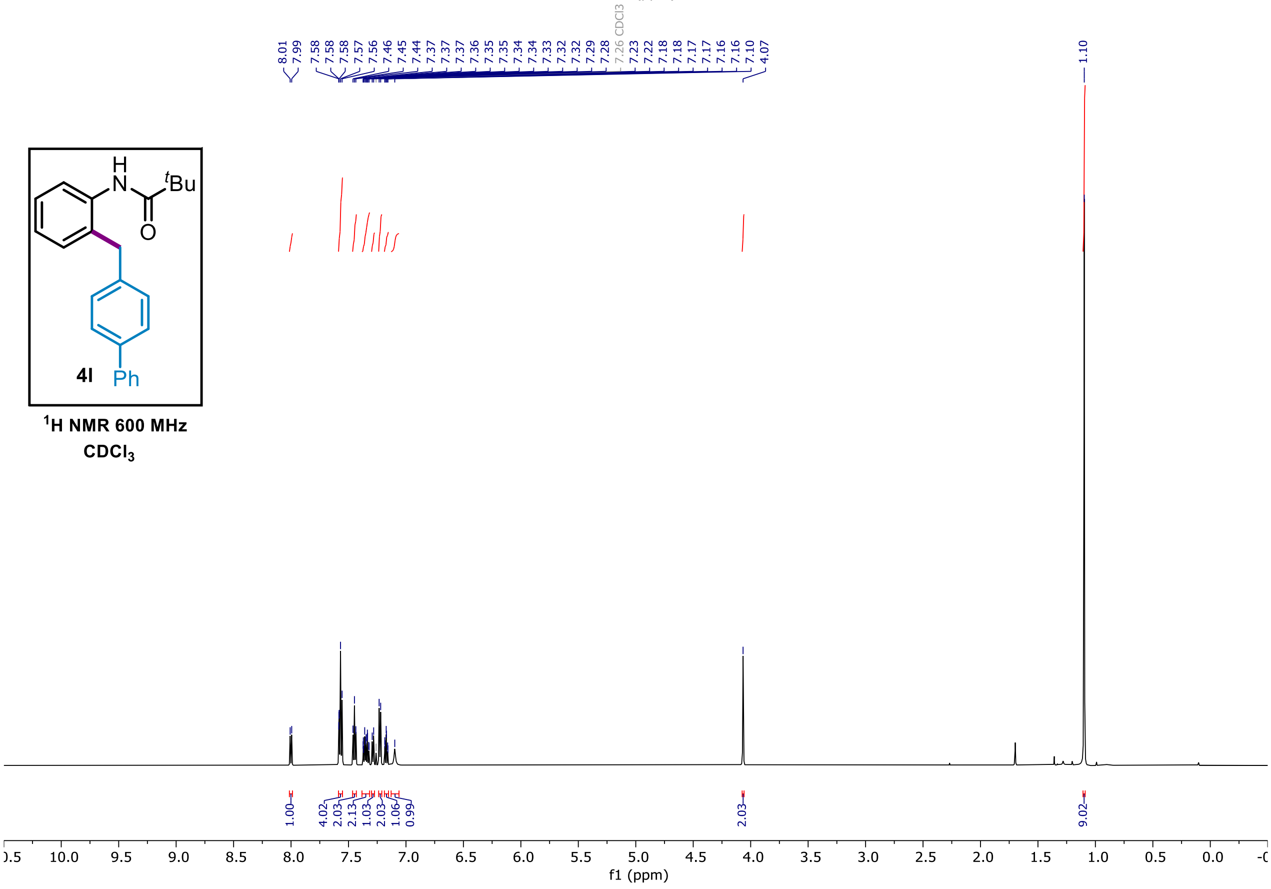
8.01
7.99
7.58
7.58
7.57
7.56
7.46
7.45
7.44
7.37
7.37
7.36
7.35
7.35
7.34
7.34
7.33
7.32
7.32
7.29
7.28
7.26 CDCl3
7.23
7.22
7.18
7.18
7.17
7.17
7.16
7.16
7.10
4.07
1.10
4l
Ph
^{1}H NMR 600 MHz
$CDCl_3$
1.00
4.02
2.03
2.13
1.03
2.03
1.06
0.99
2.03
9.02
f1 (ppm)

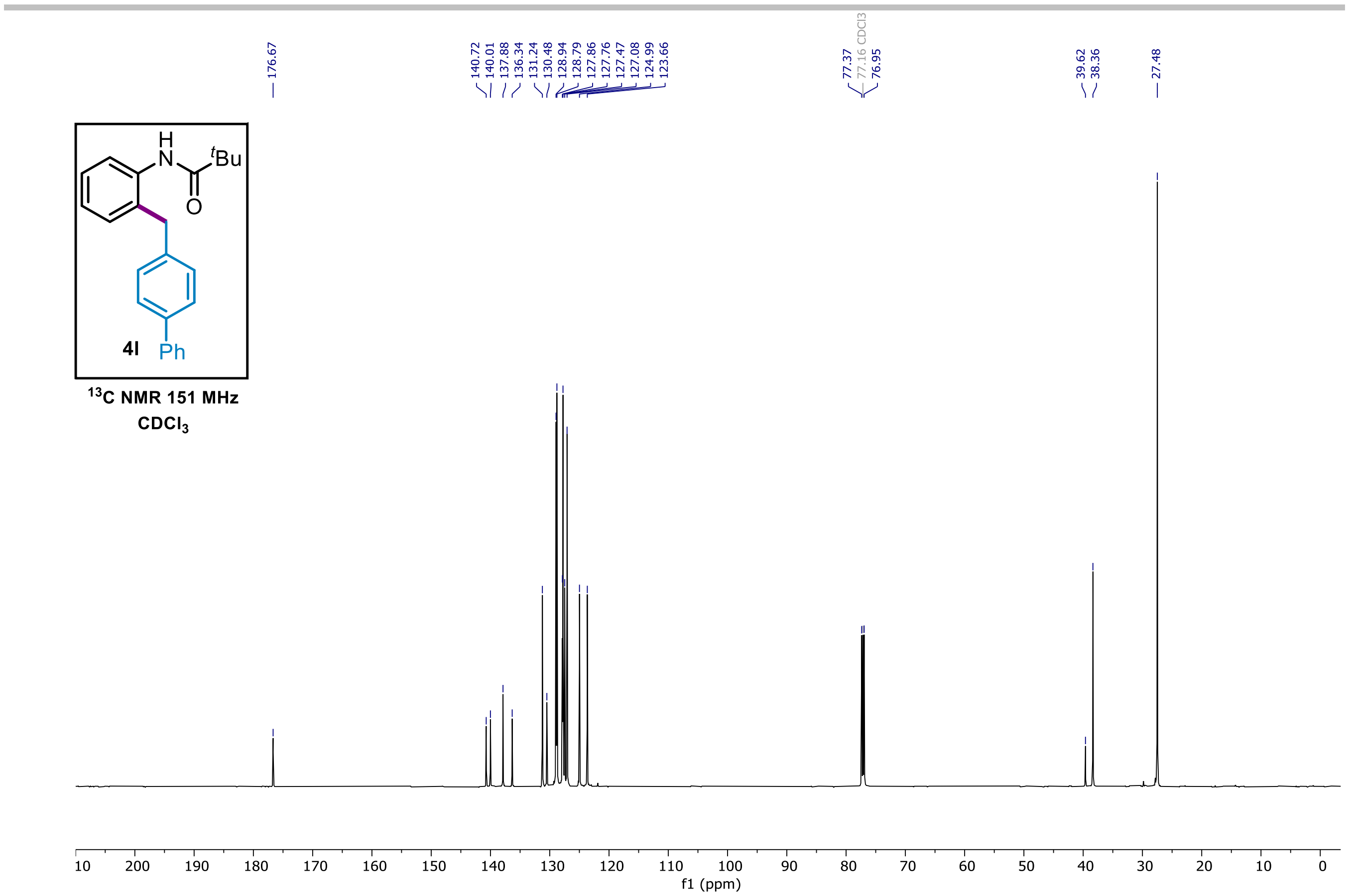

^{13}C NMR 151 MHz
$CDCl_3$

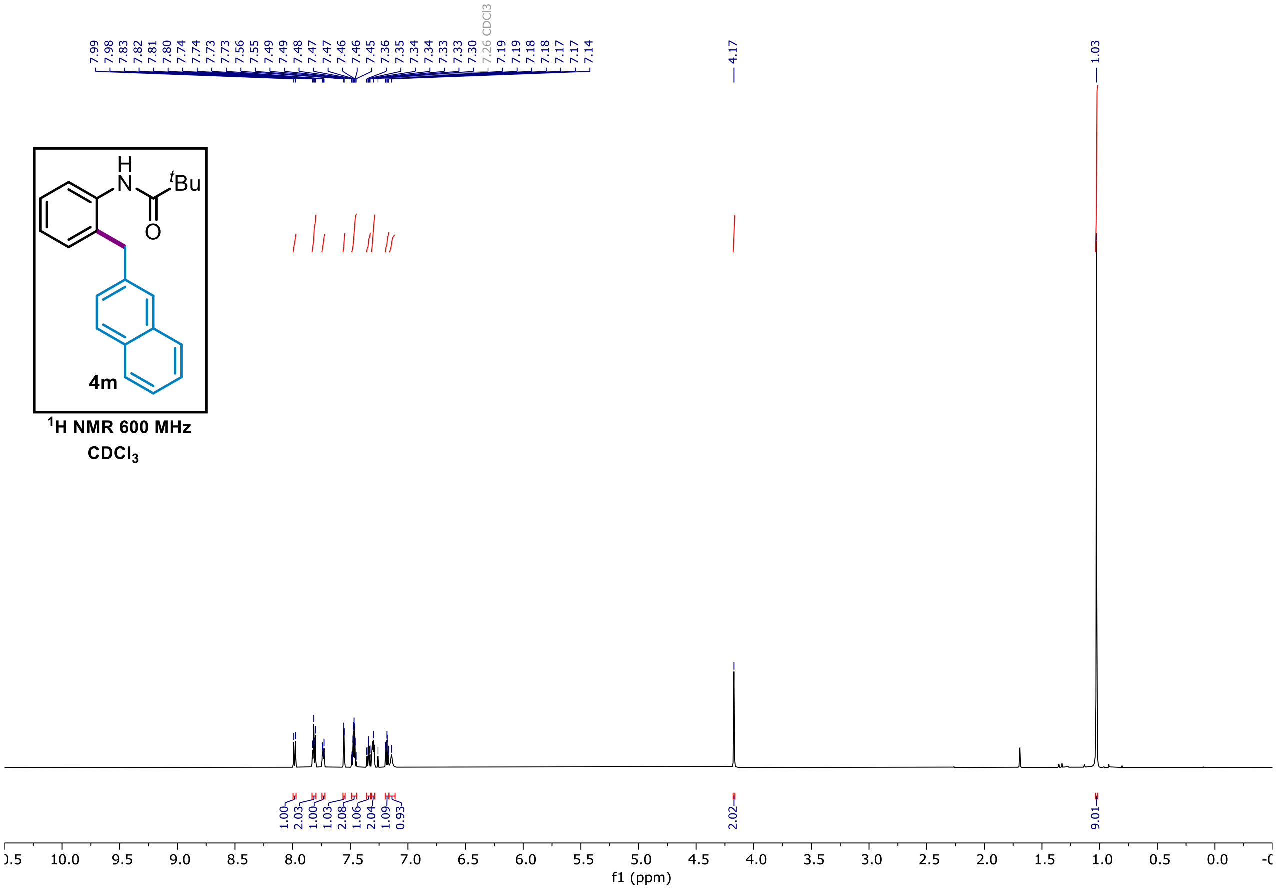

^{1}H NMR 600 MHz
$CDCl_3$

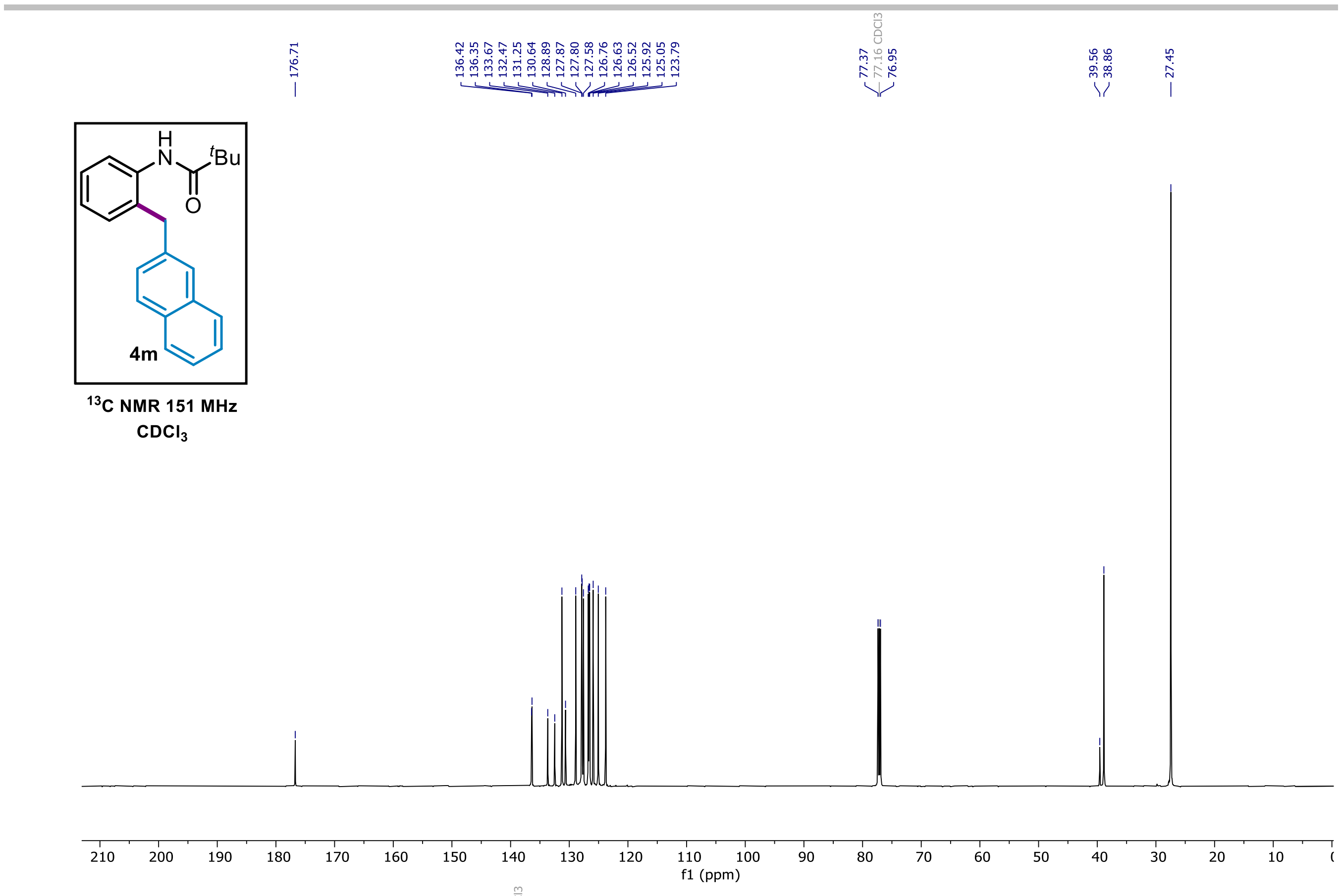

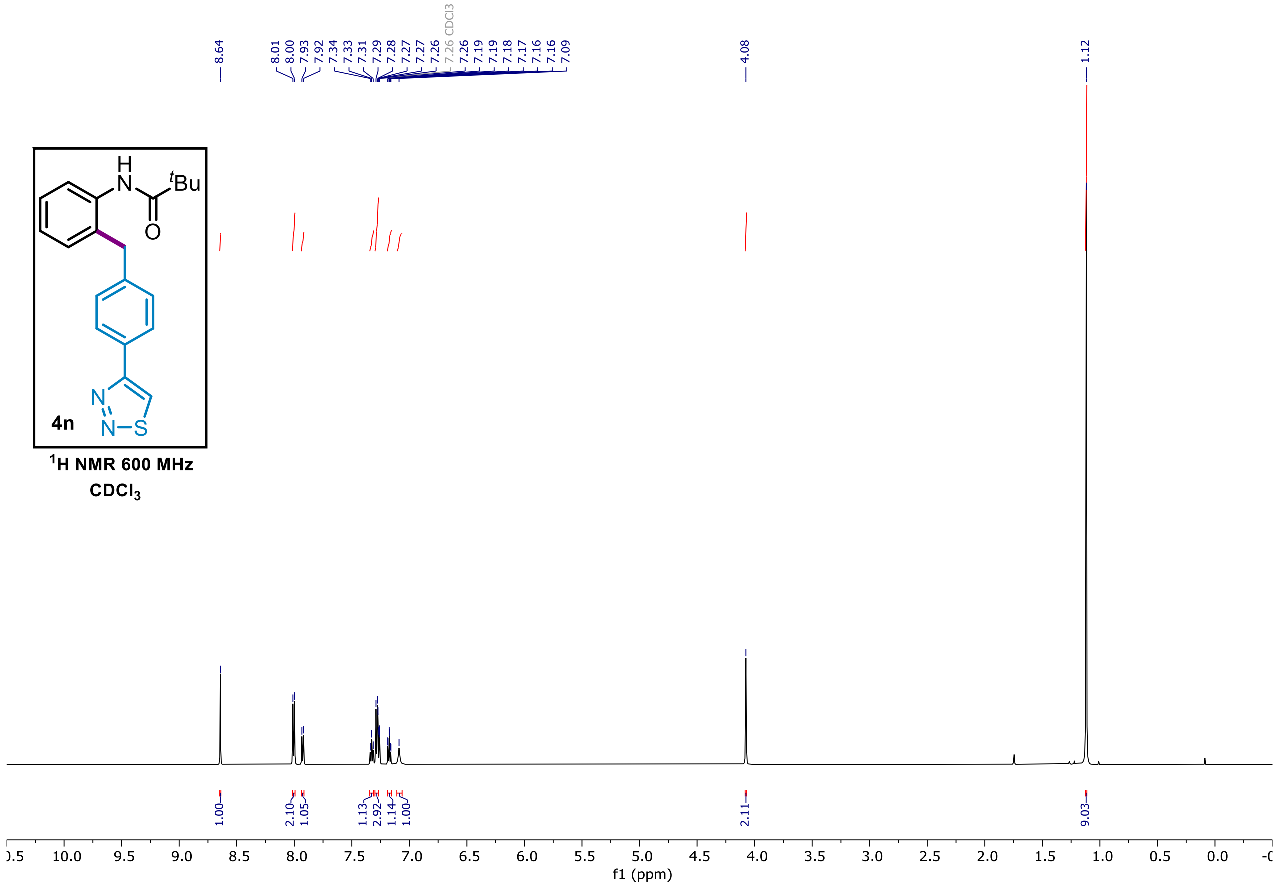

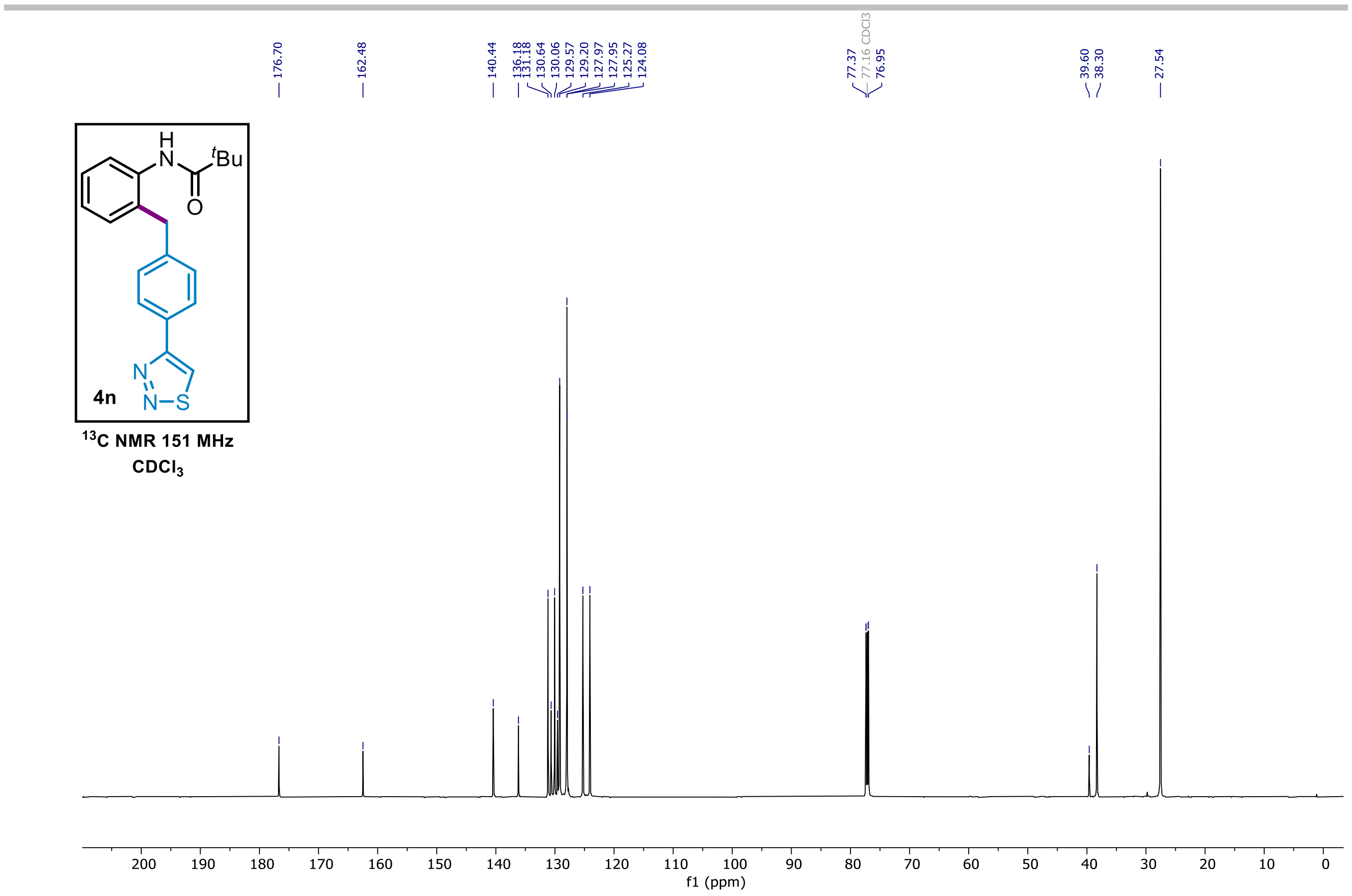

176.70
162.48
140.44
136.18
131.18
130.64
130.06
129.57
129.20
127.97
127.95
125.27
124.08
77.37
77.16 CDCl3
76.95
39.60
38.30
27.54
4n
13C NMR 151 MHz
CDCl3
f1 (ppm)

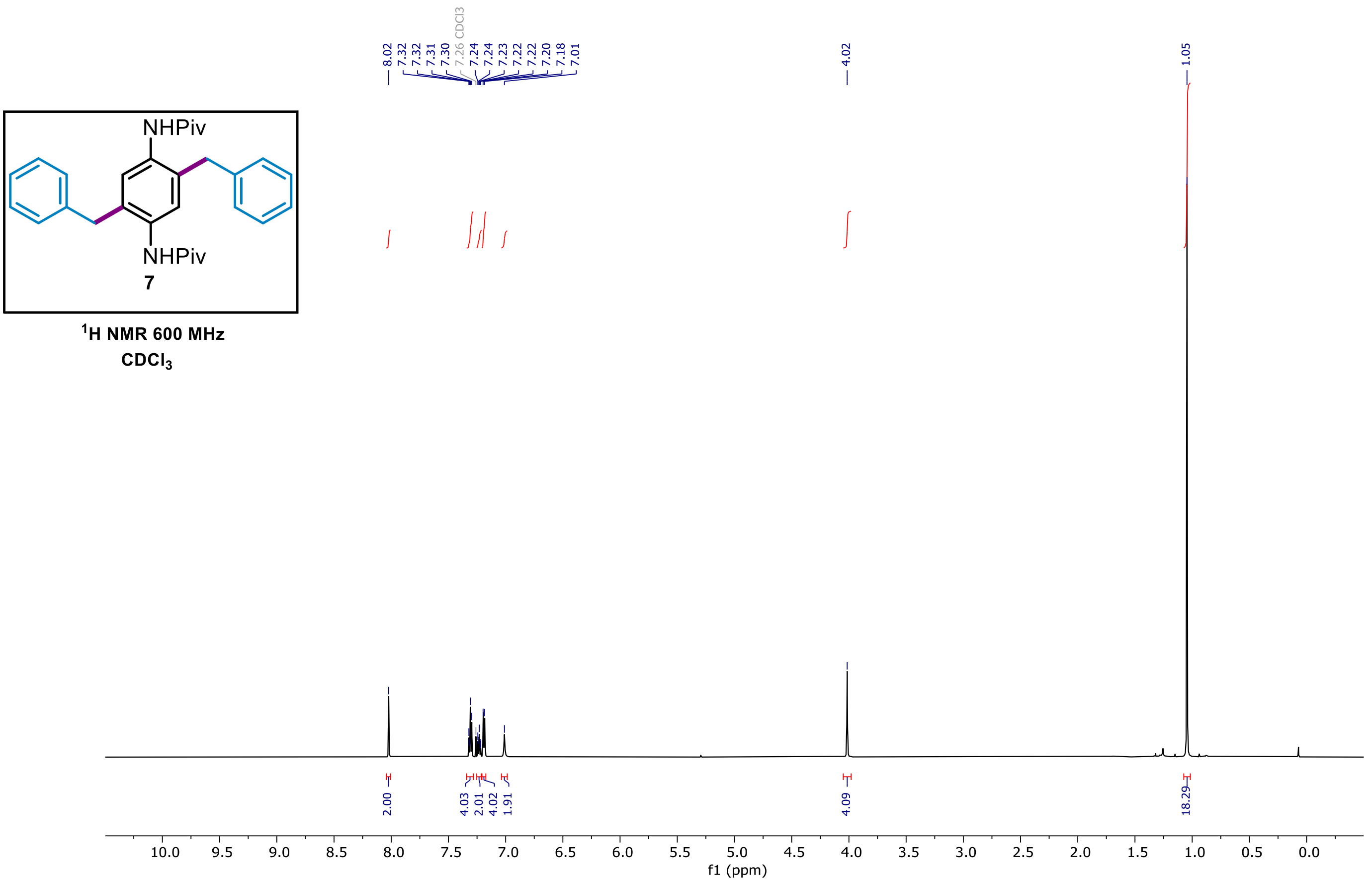

8.02
7.32
7.32
7.31
7.30
7.26 CDCl3
7.24
7.24
7.23
7.22
7.22
7.20
7.18
7.01
4.02
1.05
NHPiv
NHPiv
7
1H NMR 600 MHz
CDCl3
2.00
4.03
2.01
4.02
1.91
4.09
18.29
f1 (ppm)

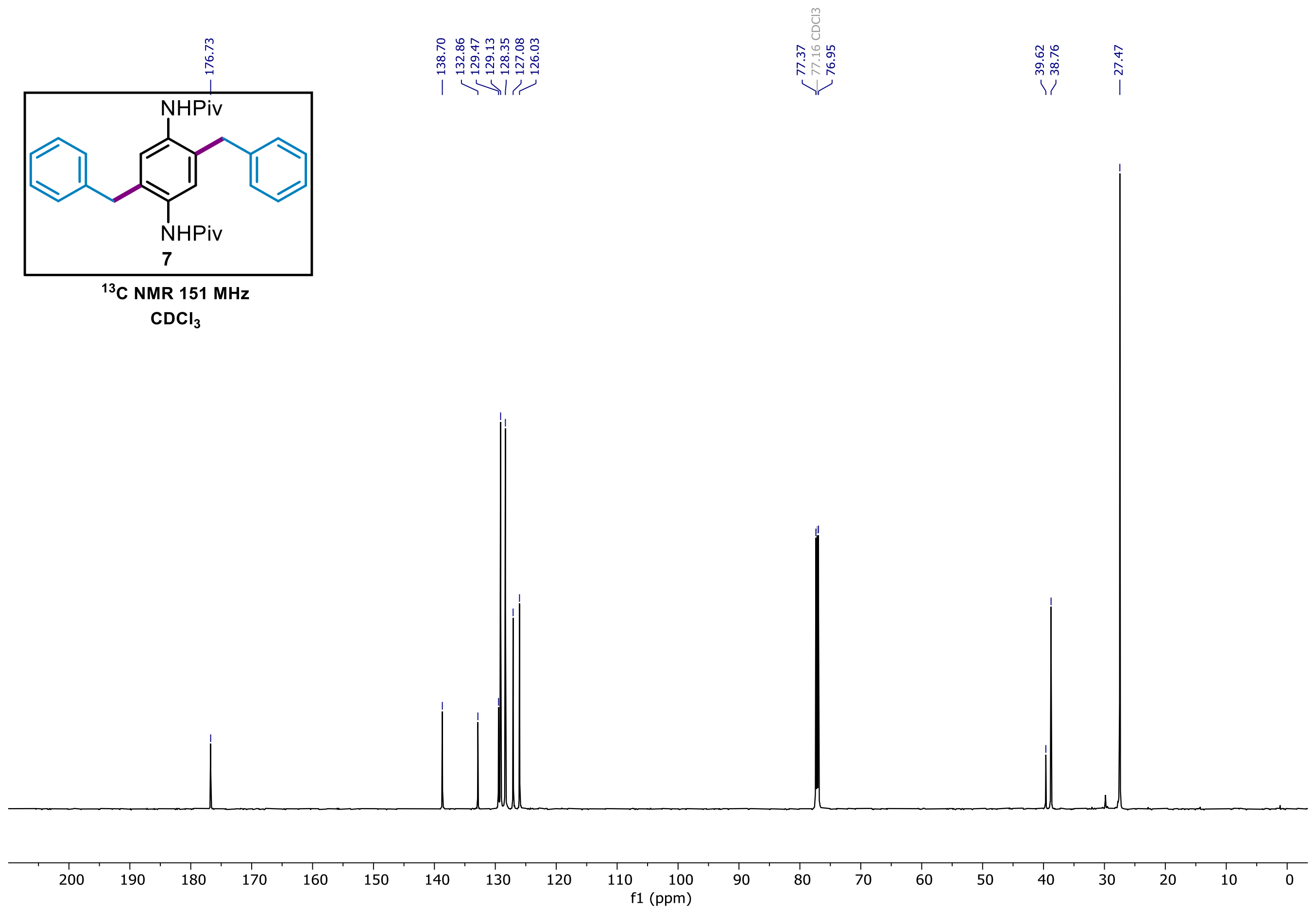

NHPiv
NHPiv
7
^{13}C NMR 151 MHz
$CDCl_3$
176.73
138.70
132.86
129.47
129.13
128.35
127.08
126.03
77.37
77.16 CDCl3
76.95
39.62
38.76
27.47
f1 (ppm)

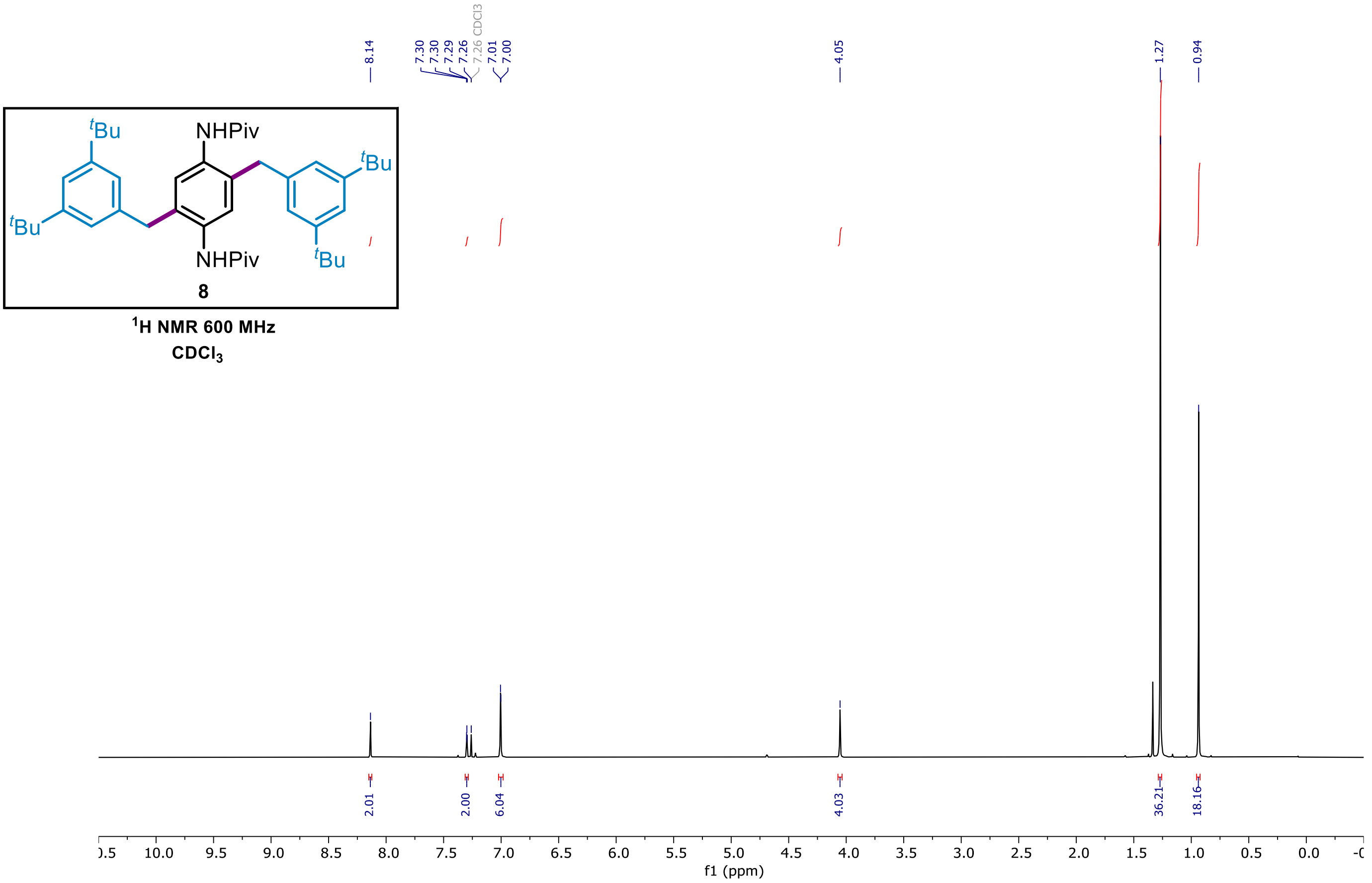

tBu
NHPiv
tBu
tBu
NHPiv
tBu
8
^{1}H NMR 600 MHz
$CDCl_3$
8.14
7.30
7.30
7.29
7.26
7.26 CDCl3
7.01
7.00
4.05
1.27
0.94
2.01
2.00
6.04
4.03
36.21
18.16
f1 (ppm)

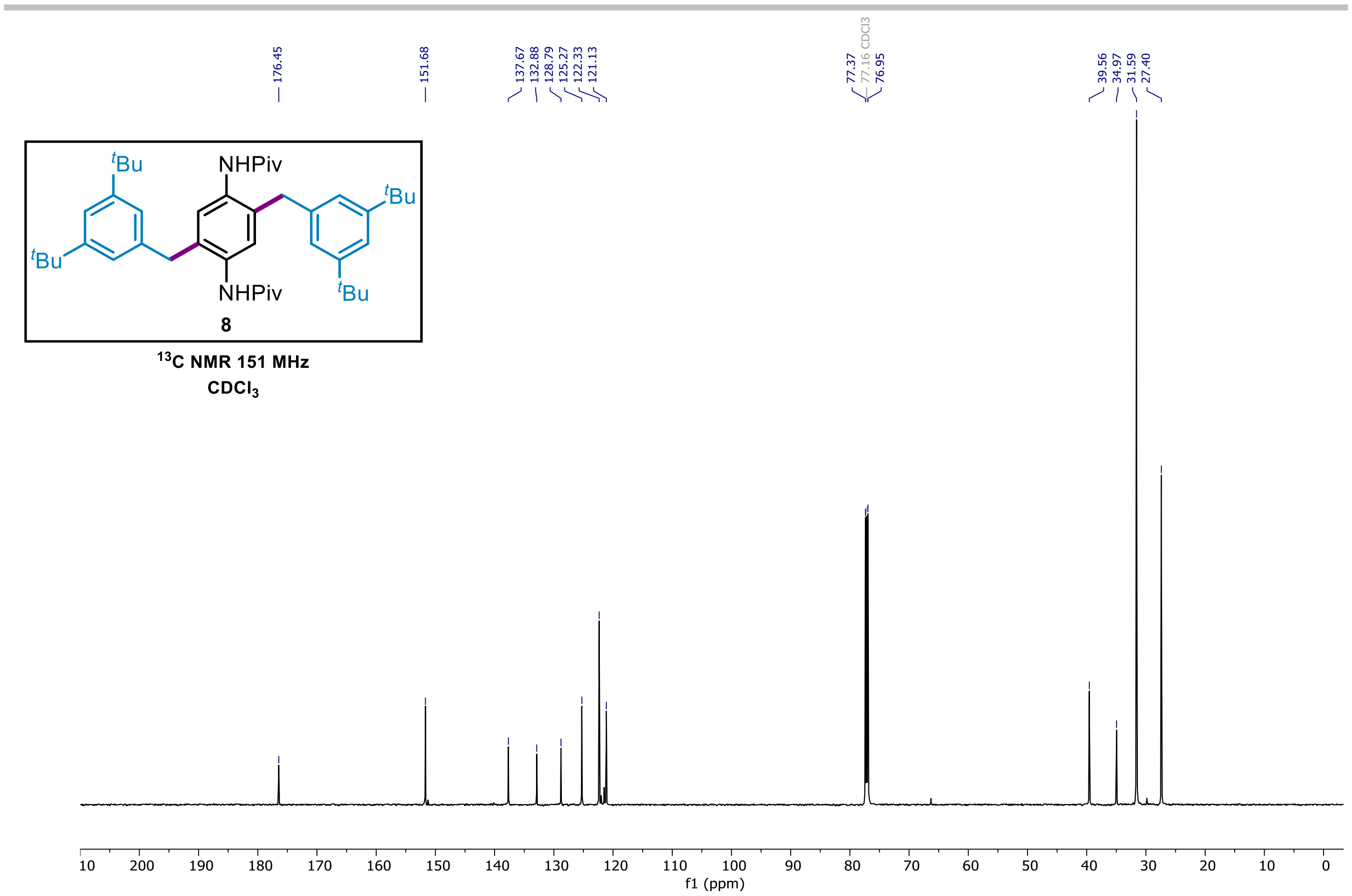

176.45
151.68
137.67
132.88
128.79
125.27
122.33
121.13
77.37
77.16 CDCl3
76.95
39.56
34.97
31.59
27.40
tBu
NHPiv
tBu
tBu
NHPiv
tBu
8
13C NMR 151 MHz
CDCl3
f1 (ppm)

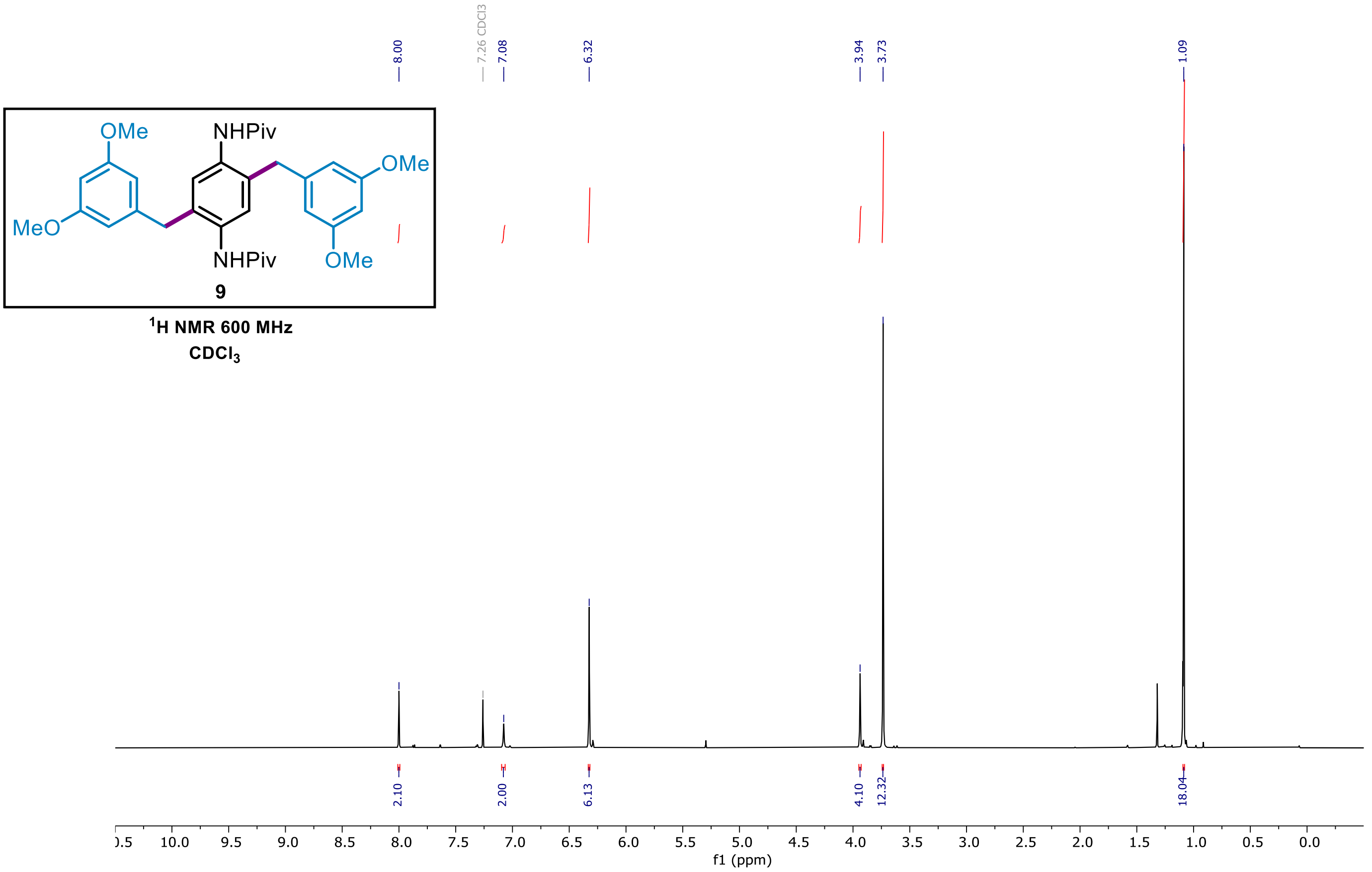

8.00
7.26 CDCl3
7.08
6.32
3.94
3.73
1.09
OMe
NHPiv
OMe
MeO
NHPiv
OMe
9
1H NMR 600 MHz
CDCl3
2.10
2.00
6.13
4.10
12.32
18.04
f1 (ppm)

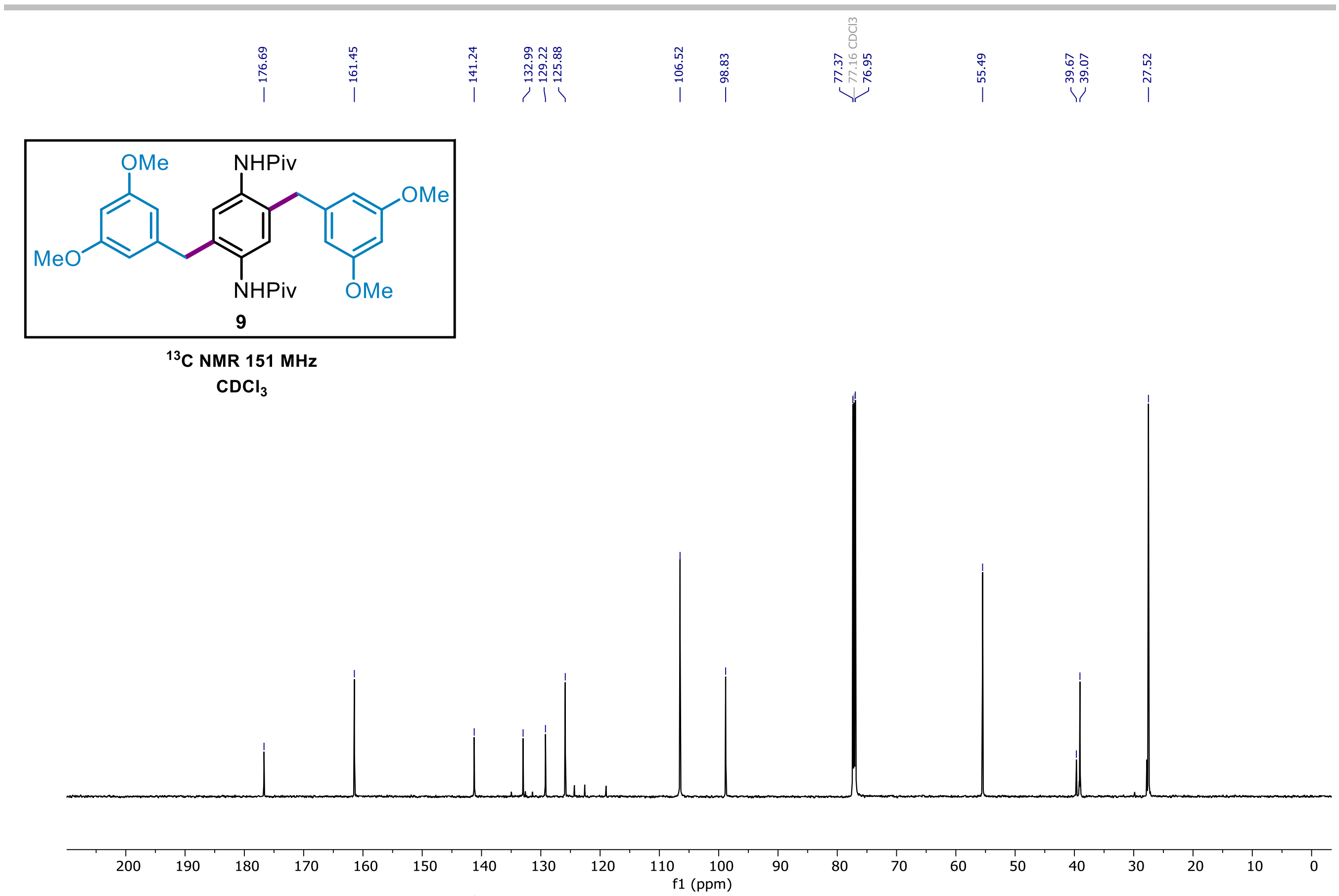
176.69
161.45
141.24
132.99
129.22
125.88
106.52
98.83
77.37
77.16 CDCl3
76.95
55.49
39.67
39.07
27.52
OMe
NHPiv
OMe
MeO
NHPiv
OMe
9
^{13}C NMR 151 MHz
$CDCl_3$
f1 (ppm)

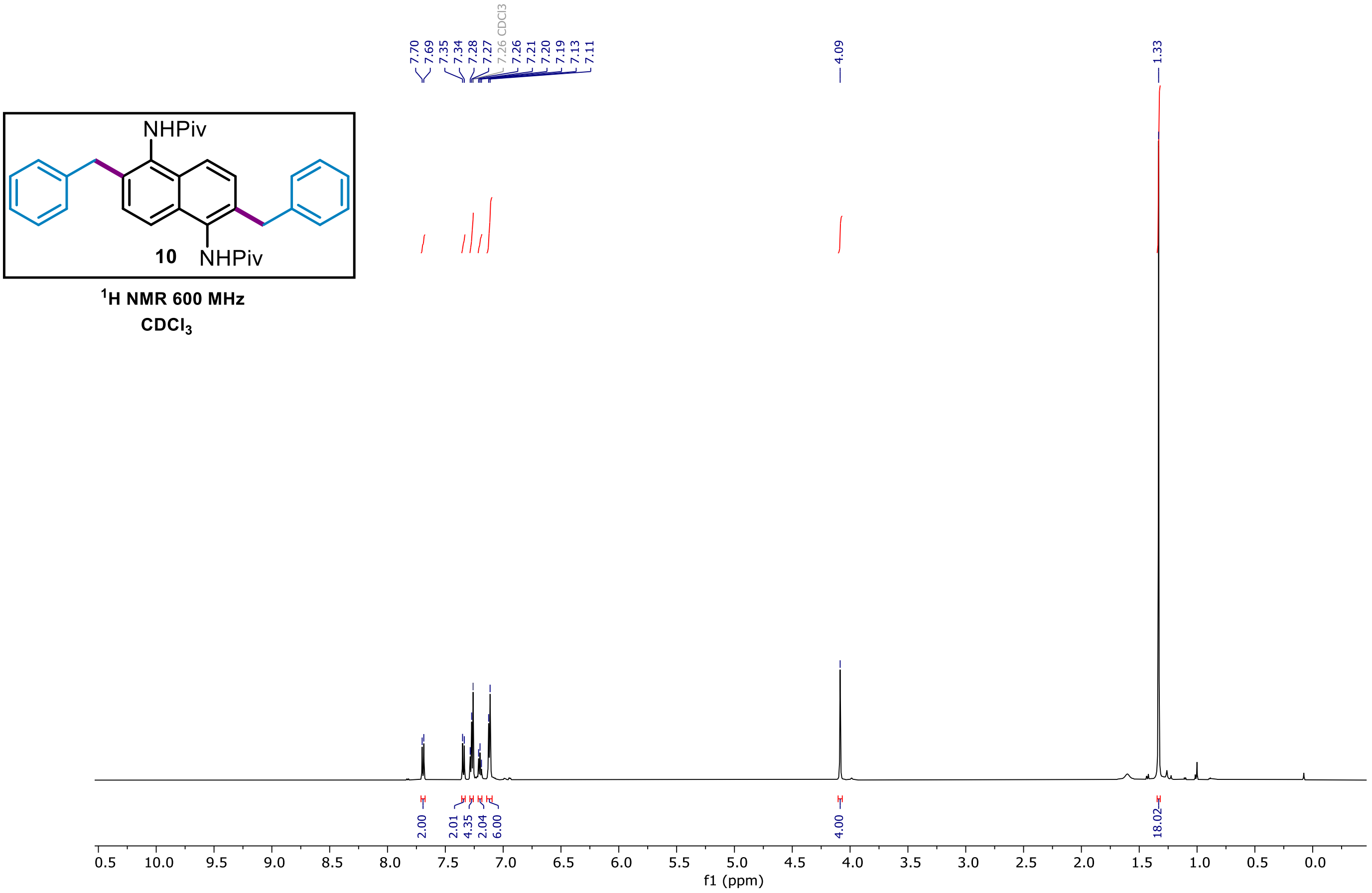
7.70
7.69
7.35
7.34
7.28
7.27
7.26 CDCl3
7.26
7.21
7.20
7.19
7.13
7.11
4.09
1.33
NHPiv
10
NHPiv
^{1}H NMR 600 MHz
$CDCl_3$
2.00
2.01
4.35
2.04
6.00
4.00
18.02
f1 (ppm)

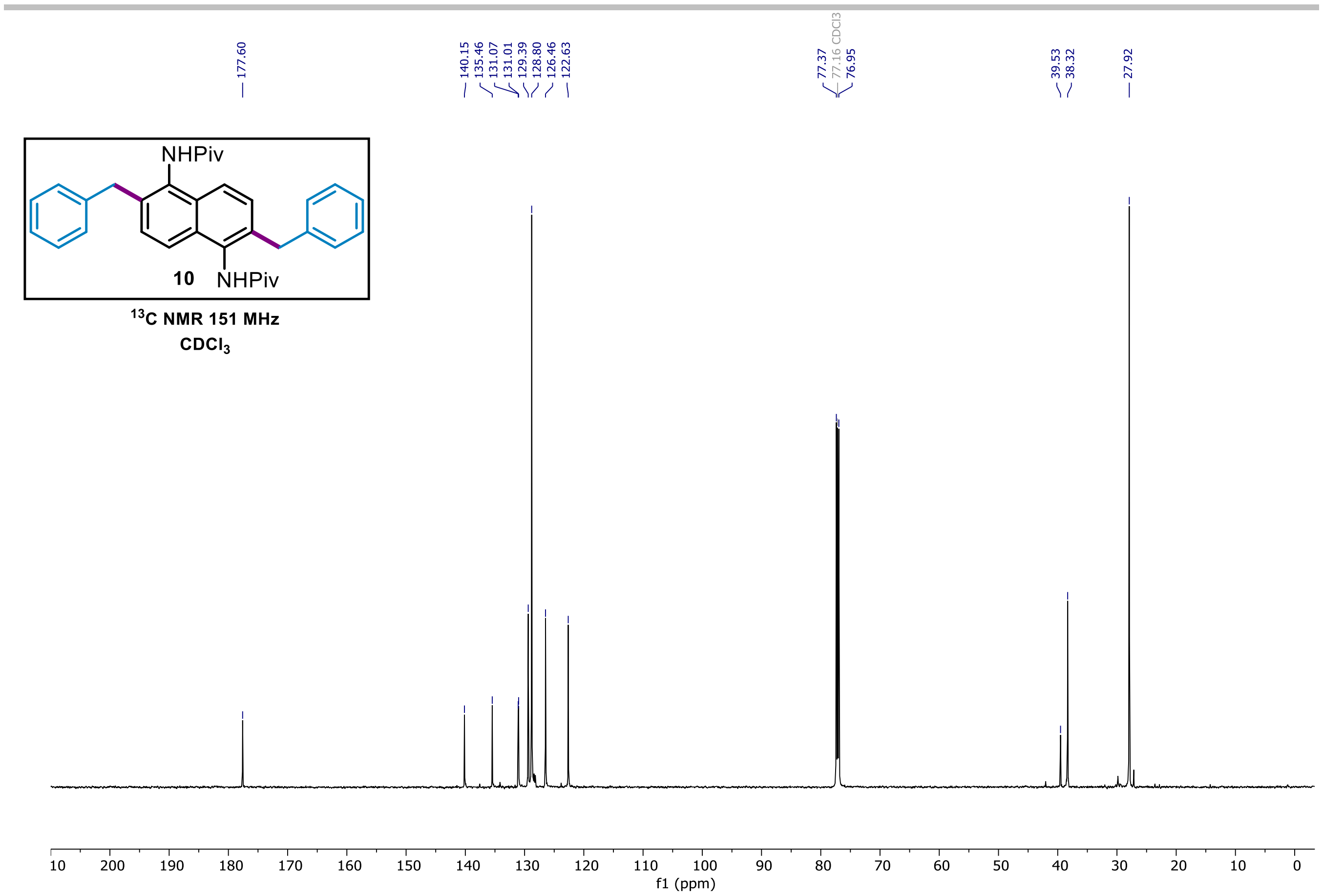
NHPiv
10
NHPiv
^{13}C NMR 151 MHz
$CDCl_3$
177.60
140.15
135.46
131.07
131.01
129.39
128.80
126.46
122.63
77.37
77.16 CDCl3
76.95
39.53
38.32
27.92
f1 (ppm)

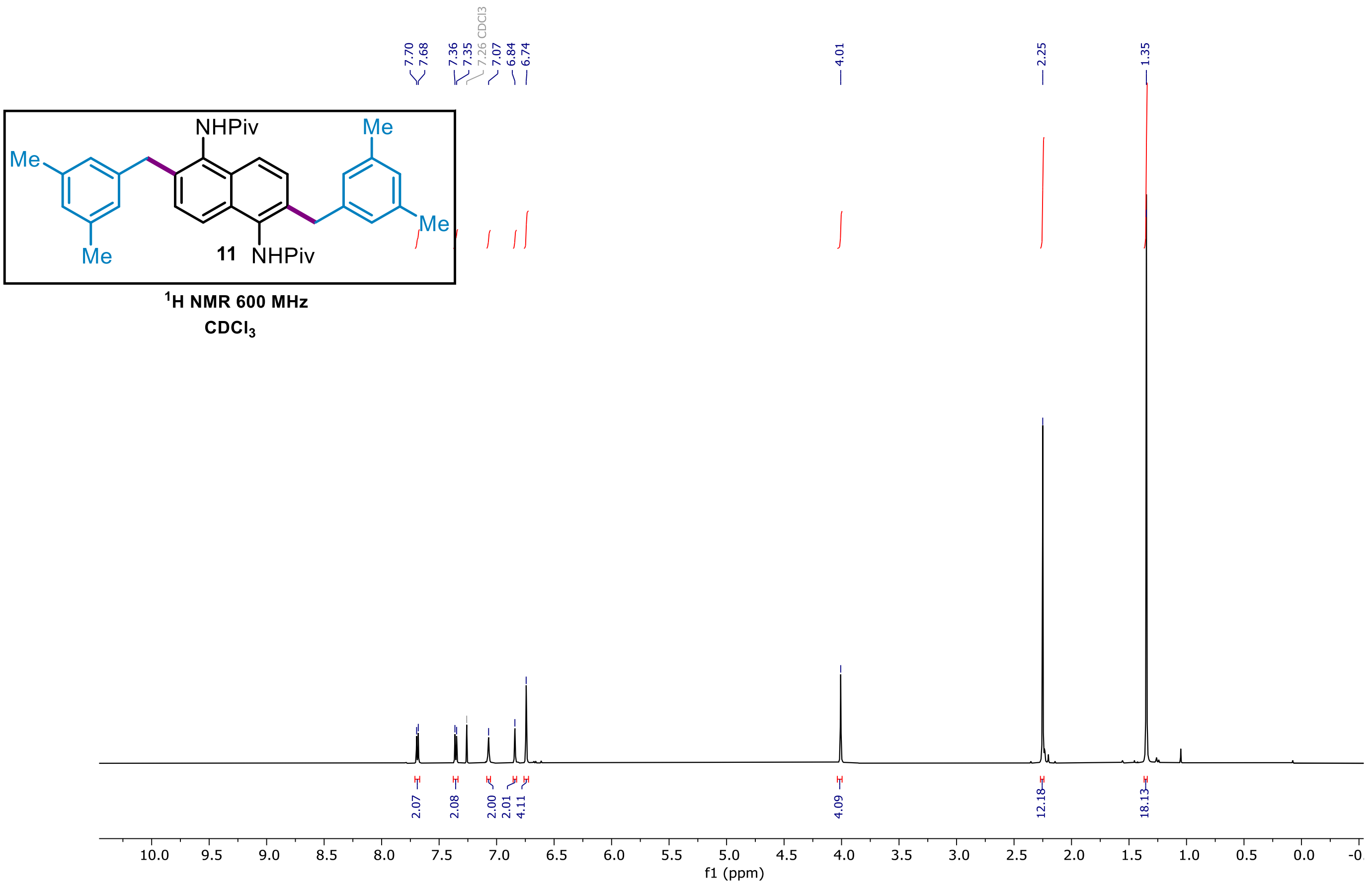
NHPiv
Me
Me
Me
Me
11
NHPiv
^{1}H NMR 600 MHz
$CDCl_3$
7.70
7.68
7.36
7.35
7.26 CDCl3
7.07
6.84
6.74
4.01
2.25
1.35
2.07
2.08
2.00
2.01
4.11
4.09
12.18
18.13
f1 (ppm)

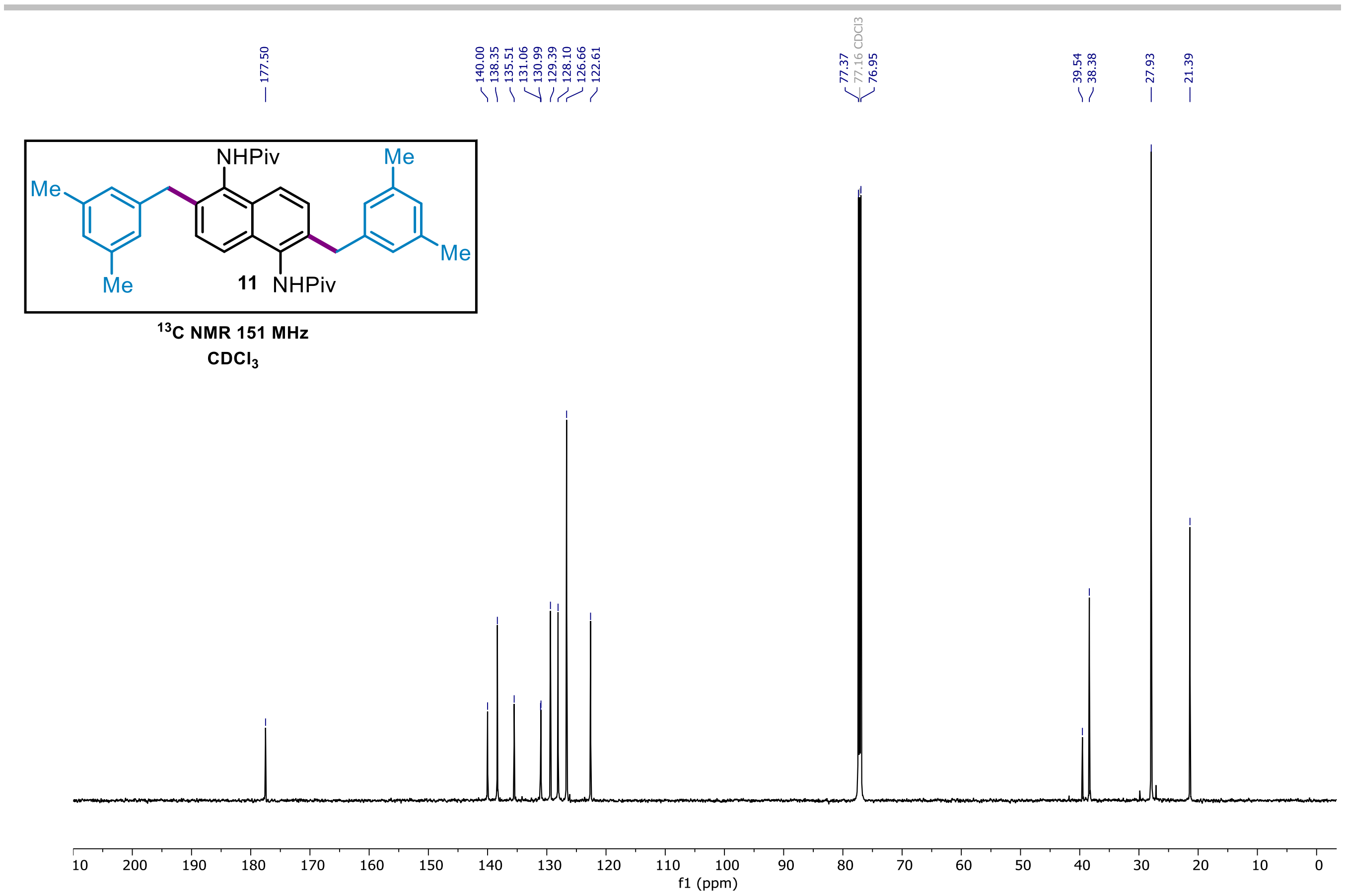
177.50
140.00
138.35
135.51
131.06
130.99
129.39
128.10
126.66
122.61
77.37
77.16 CDCl3
76.95
39.54
38.38
27.93
21.39
NHPiv
Me
Me
Me
Me
11
NHPiv
^{13}C NMR 151 MHz
$CDCl_3$
f1 (ppm)

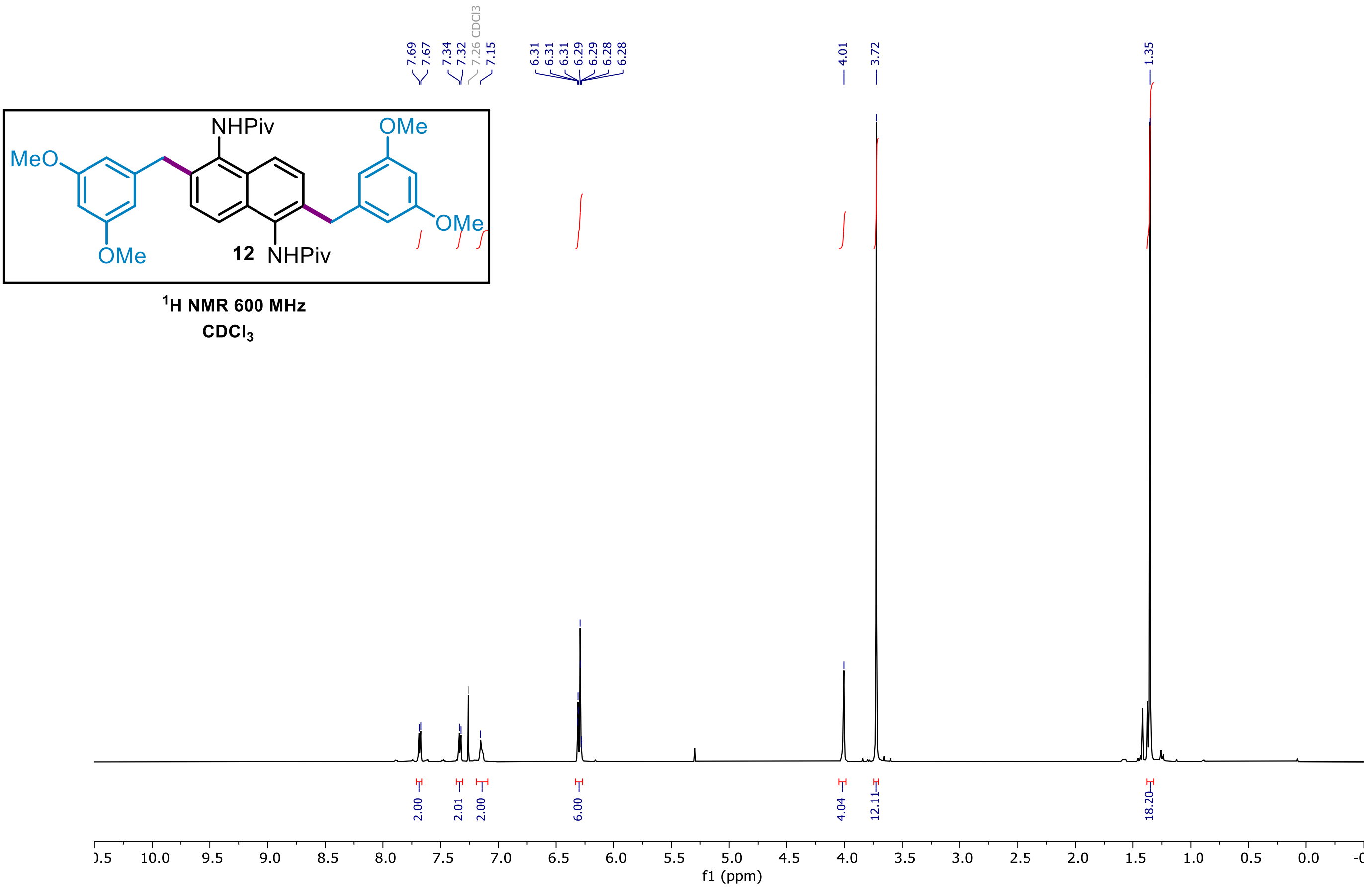
7.69
7.67
7.34
7.32
7.26 CDCl3
7.15
6.31
6.31
6.31
6.29
6.29
6.28
6.28
4.01
3.72
1.35
NHPiv
MeO
OMe
OMe
OMe
12
NHPiv
^{1}H NMR 600 MHz
$CDCl_3$
2.00
2.01
2.00
6.00
4.04
12.11
18.20
f1 (ppm)

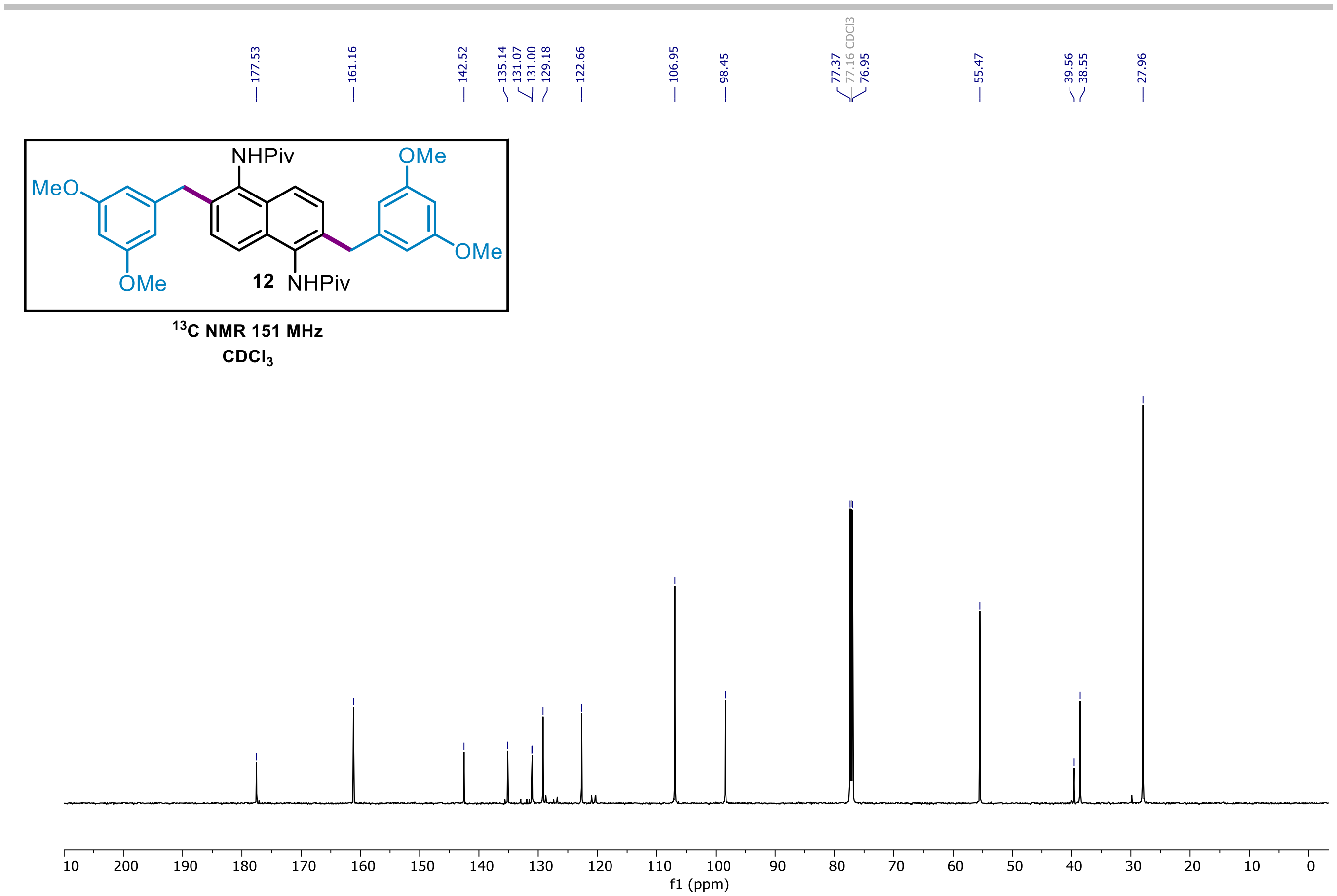

177.53
161.16
142.52
135.14
131.07
131.00
129.18
122.66
106.95
98.45
77.37
77.16 CDCl3
76.95
55.47
39.56
38.55
27.96
NHPiv
OMe
MeO
OMe
OMe
12 NHPiv
^{13}C NMR 151 MHz
$CDCl_3$
f1 (ppm)

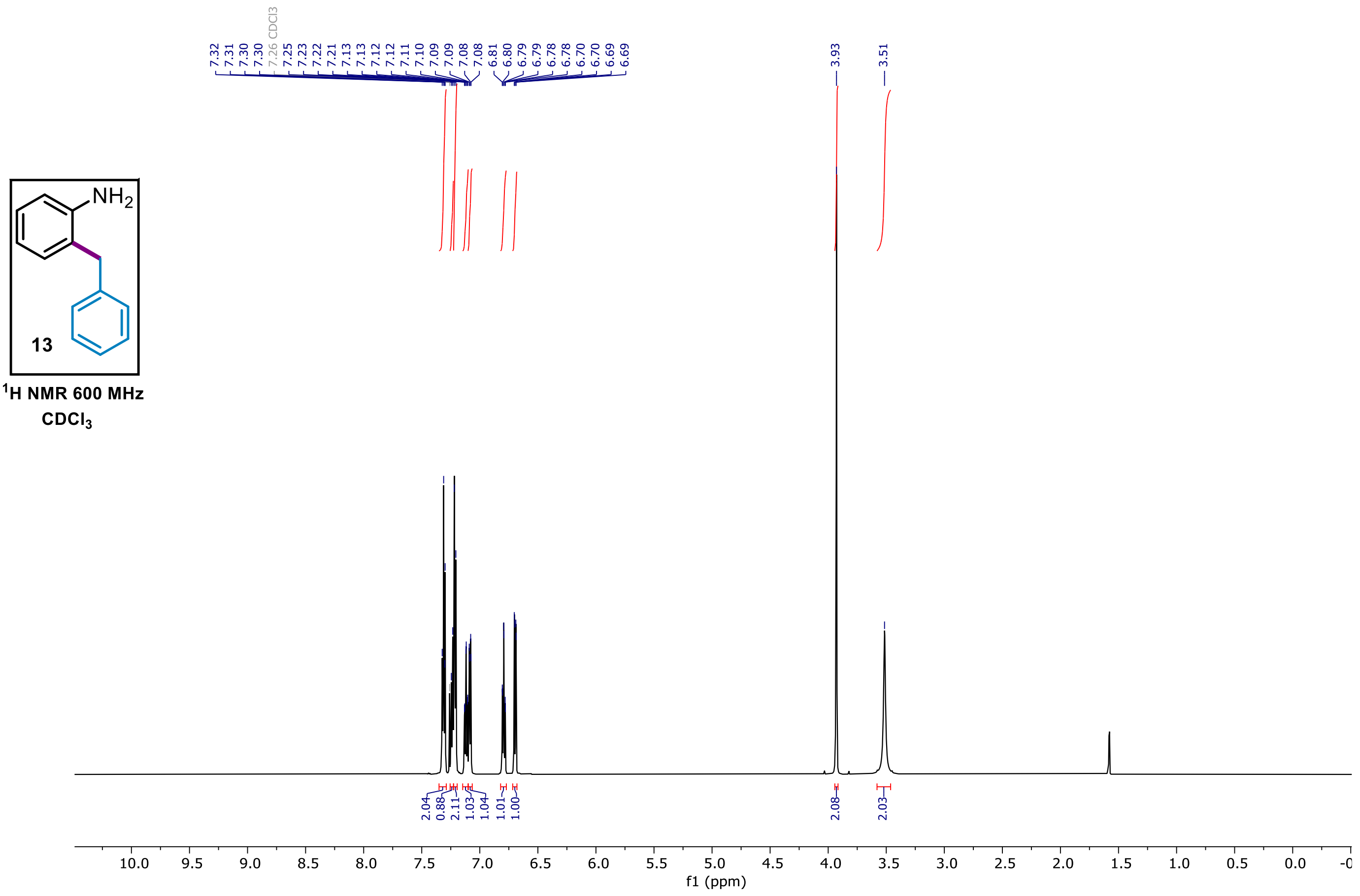

7.32
7.31
7.30
7.26 CDCl3
7.25
7.23
7.22
7.21
7.13
7.13
7.12
7.12
7.11
7.10
7.09
7.09
7.08
7.08
6.81
6.80
6.79
6.79
6.78
6.78
6.70
6.70
6.69
6.69
3.93
3.51
NH_2
13
^{1}H NMR 600 MHz
$CDCl_3$
2.04
0.88
2.11
1.03
1.04
1.01
1.00
2.08
2.03
f1 (ppm)

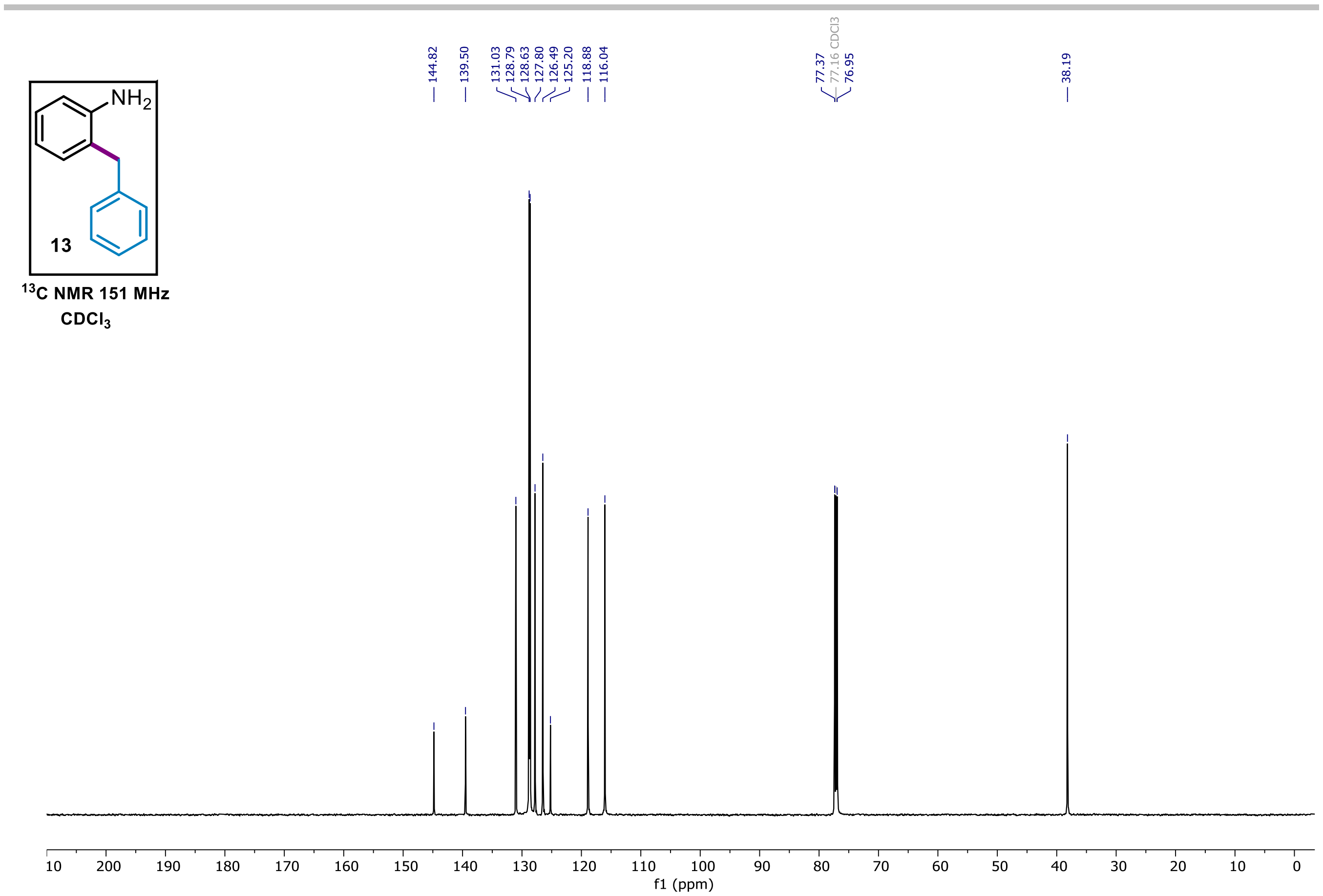
NH2
13
13C NMR 151 MHz
CDCl3
144.82
139.50
131.03
128.79
128.63
127.80
126.49
125.20
118.88
116.04
77.37
77.16 CDCl3
76.95
38.19
f1 (ppm)

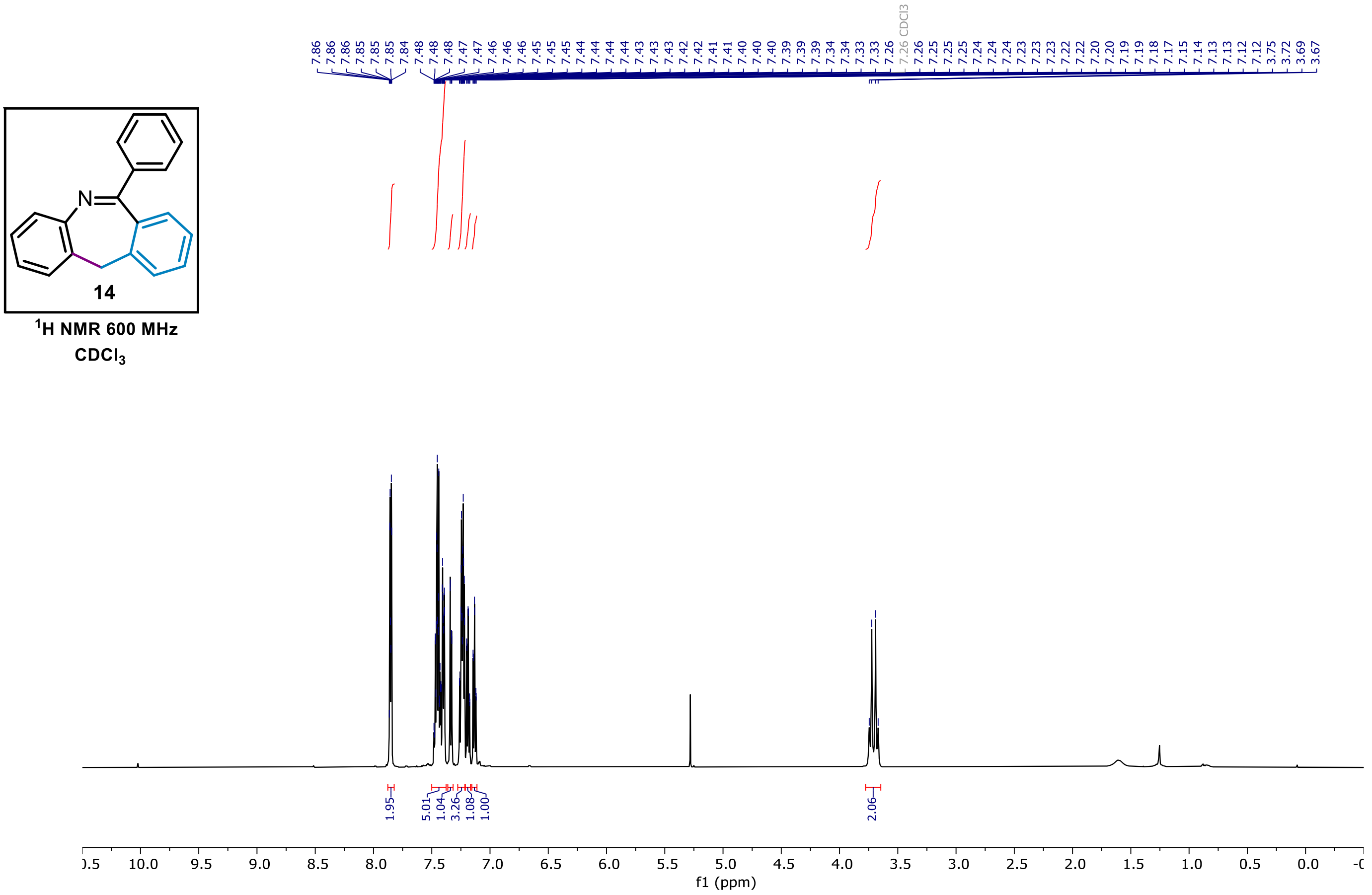
N
14
1H NMR 600 MHz
CDCl3
7.86 7.86 7.86 7.85 7.85 7.84 7.48 7.48 7.47 7.47 7.46 7.46 7.45 7.45 7.45 7.44 7.44 7.44 7.44 7.43 7.43 7.43 7.42 7.42 7.41 7.41 7.40 7.40 7.40 7.39 7.39 7.39 7.34 7.33 7.33 7.26 CDCl3 7.26 7.25 7.25 7.25 7.24 7.24 7.24 7.23 7.23 7.23 7.22 7.22 7.22 7.20 7.20 7.19 7.19 7.18 7.17 7.15 7.14 7.13 7.13 7.12 7.12 3.75 3.72 3.69 3.67
1.95
5.01
1.04
3.26
1.08
1.00
2.06
f1 (ppm)

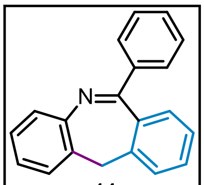

14

^{13}C NMR 151 MHz
$CDCl_3$

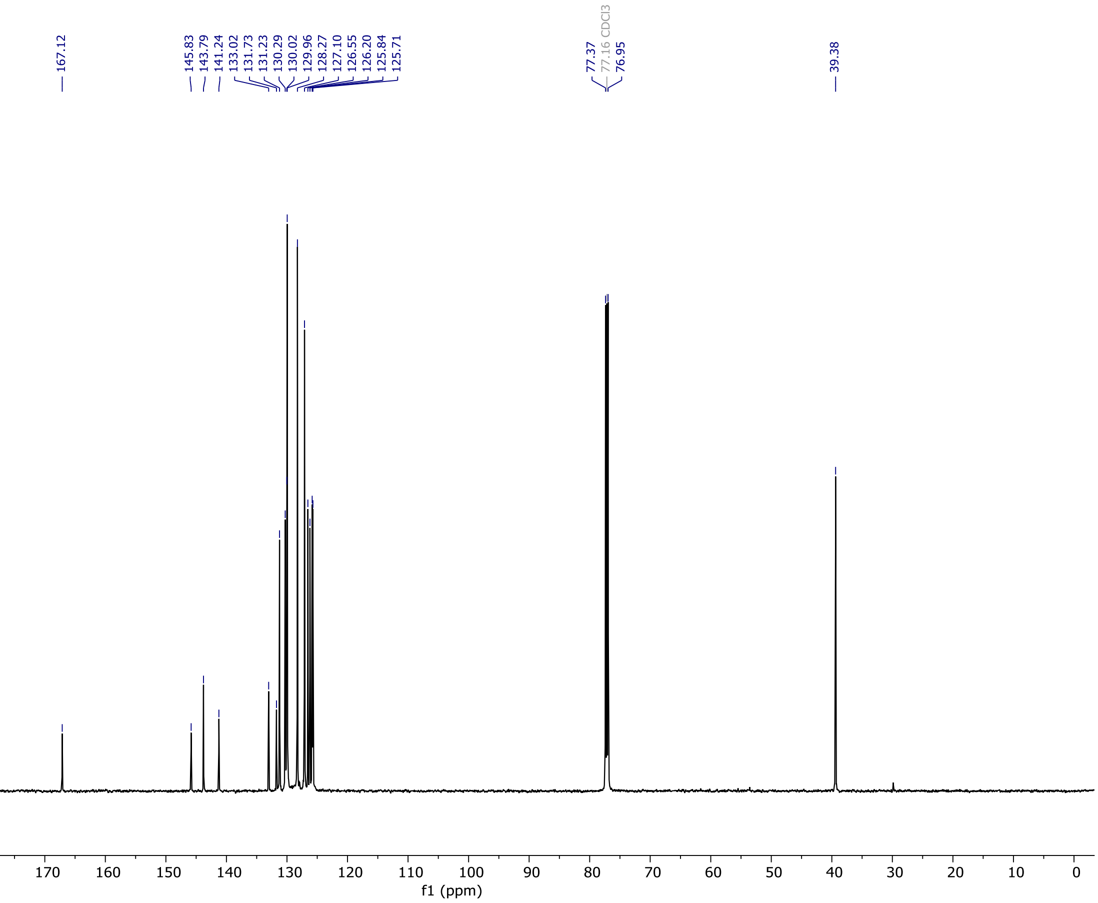

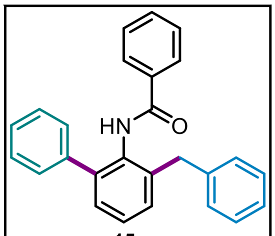

15

^{1}H NMR 600 MHz
$CDCl_3$

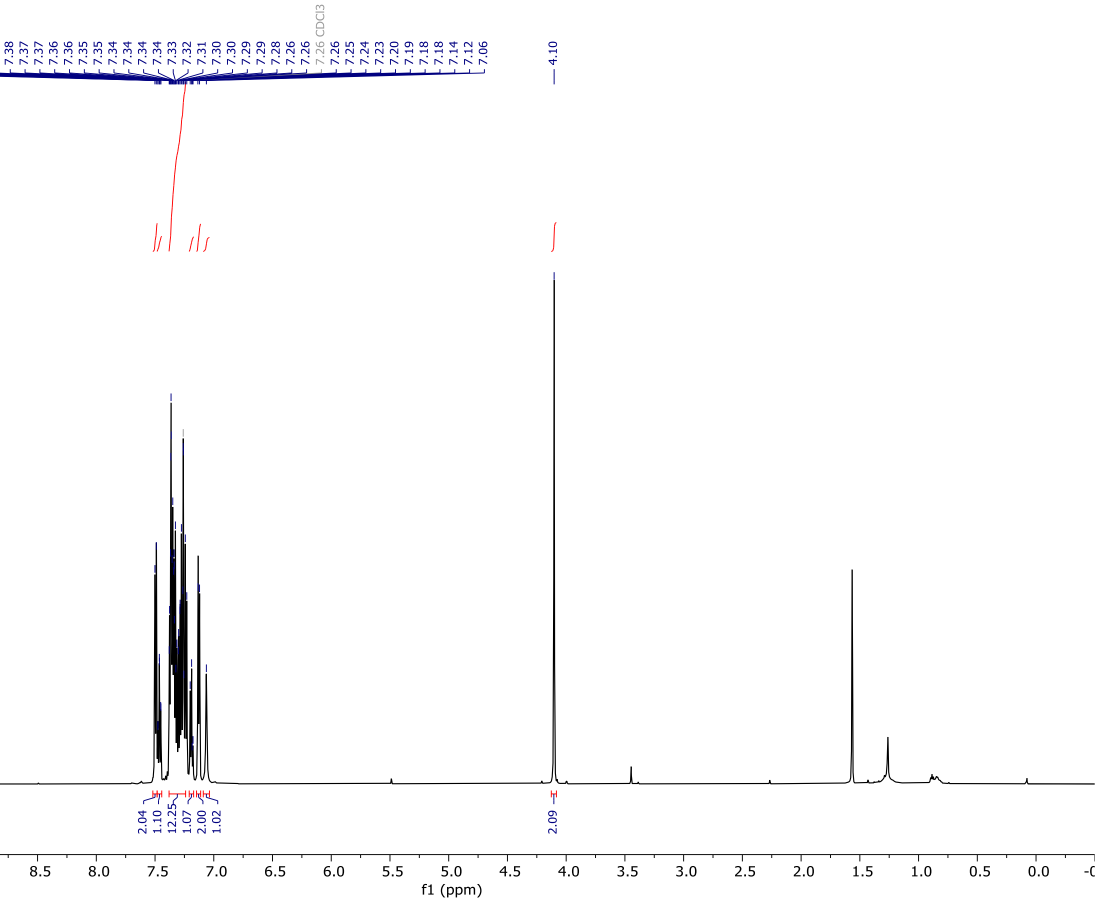

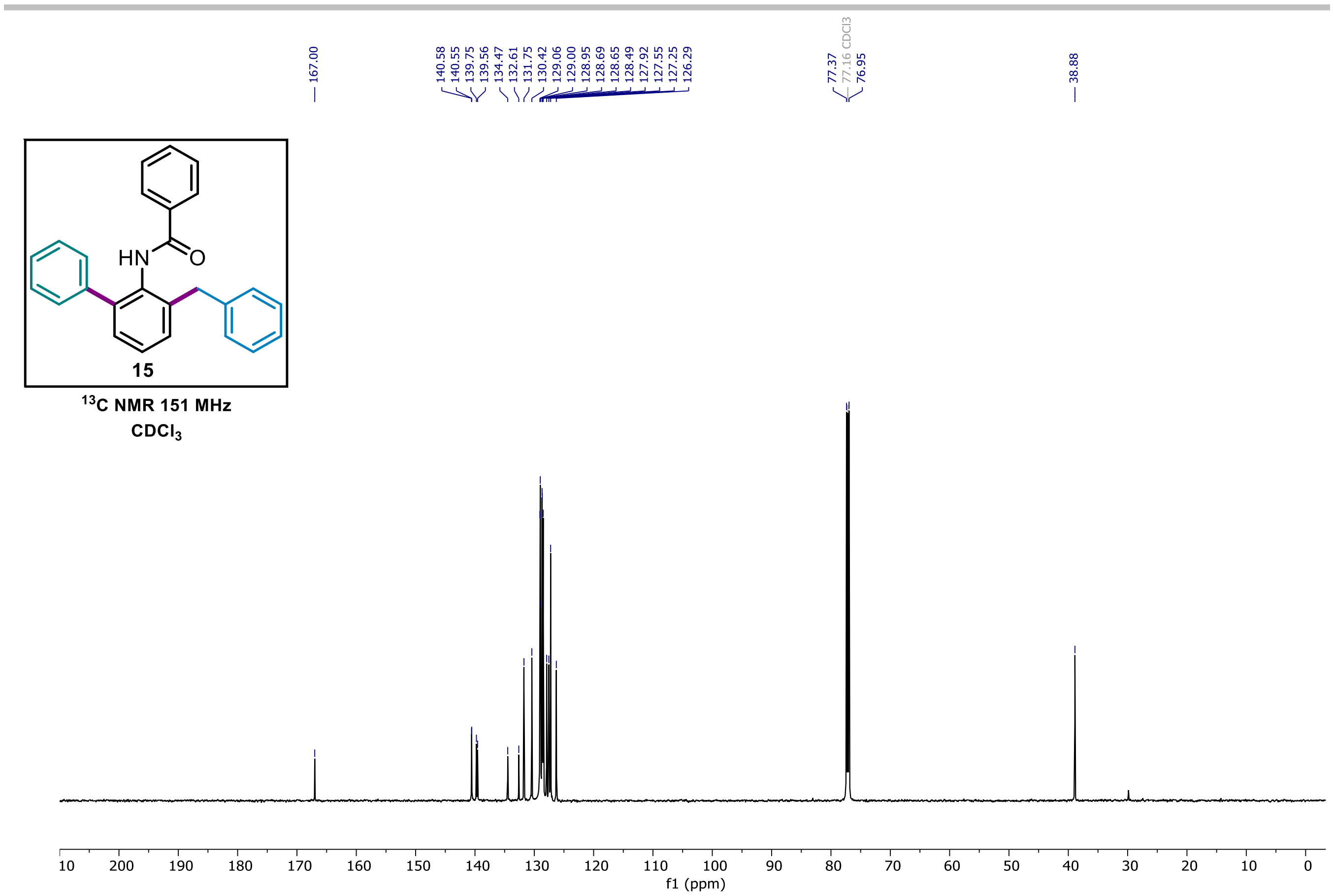
167.00
140.58
140.55
139.75
139.56
134.47
132.61
131.75
130.42
129.06
129.00
128.95
128.69
128.65
128.49
127.92
127.55
127.25
126.29
77.37
77.16 CDCl3
76.95
38.88
HN
O
15
13C NMR 151 MHz
CDCl3
f1 (ppm)

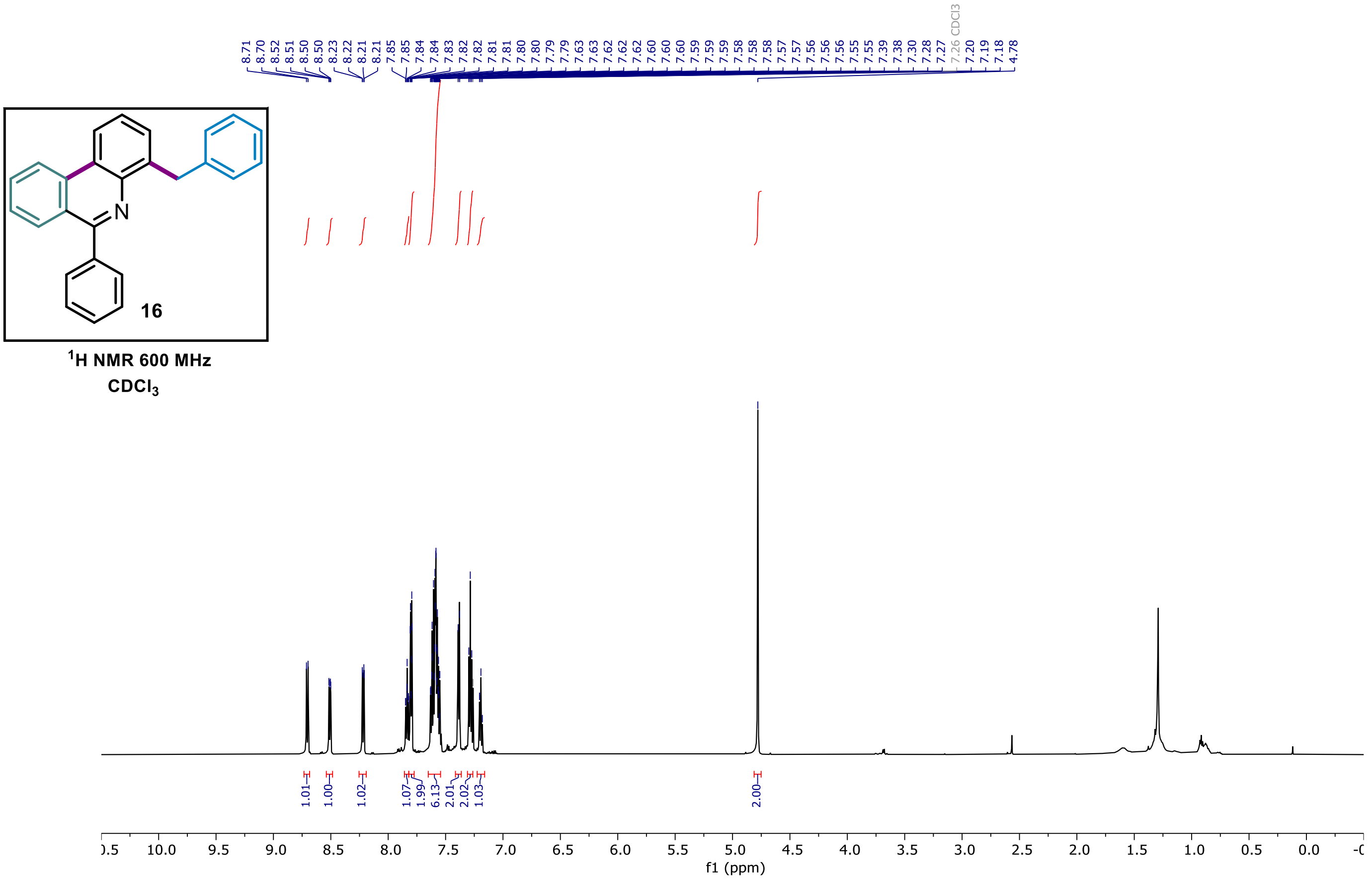
8.71
8.70
8.52
8.51
8.50
8.50
8.23
8.22
8.21
8.21
7.85
7.84
7.84
7.83
7.82
7.82
7.81
7.80
7.80
7.79
7.79
7.63
7.63
7.62
7.62
7.62
7.60
7.60
7.59
7.59
7.59
7.58
7.58
7.58
7.57
7.57
7.56
7.56
7.55
7.55
7.39
7.38
7.30
7.28
7.27
7.26 CDCl3
7.20
7.19
7.18
4.78
N
16
1H NMR 600 MHz
CDCl3
1.01
1.00
1.02
1.07
1.99
6.13
2.01
2.02
1.03
2.00
f1 (ppm)

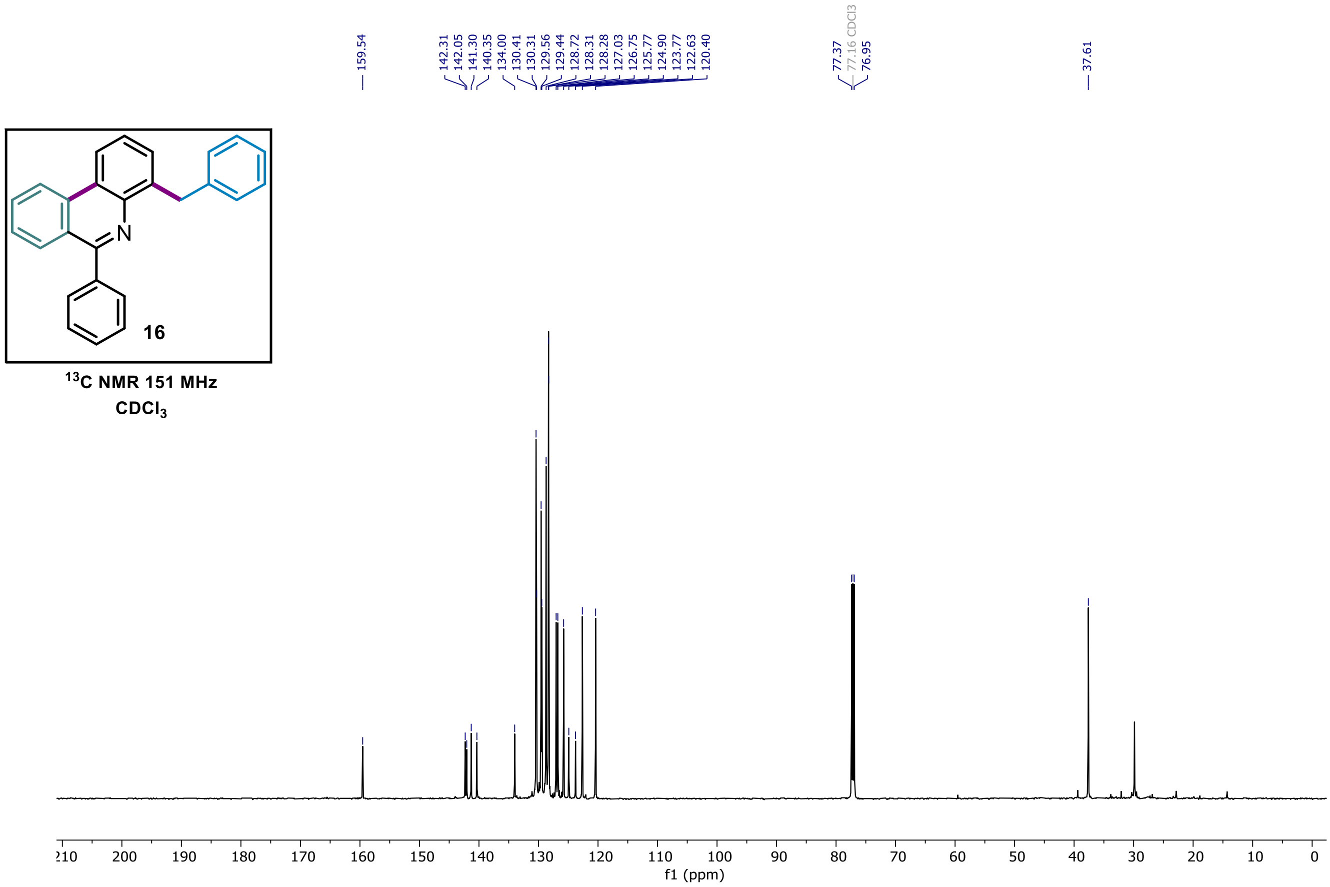

159.54
142.31
142.05
141.30
140.35
134.00
130.41
130.31
129.56
129.44
128.72
128.31
128.28
127.03
126.75
125.77
124.90
123.77
122.63
120.40
77.37
77.16 CDCl3
76.95
37.61
16
^{13}C NMR 151 MHz
$CDCl_3$
f1 (ppm)
